# Development and validation of an autonomous artificial intelligence agent for clinical decision-making in oncology

**DOI:** 10.1038/s43018-025-00991-6

**Published:** 2025-06-06

**Authors:** Dyke Ferber, Omar S. M. El Nahhas, Georg Wölflein, Isabella C. Wiest, Jan Clusmann, Marie-Elisabeth Leßmann, Sebastian Foersch, Jacqueline Lammert, Maximilian Tschochohei, Dirk Jäger, Manuel Salto-Tellez, Nikolaus Schultz, Daniel Truhn, Jakob Nikolas Kather

**Affiliations:** 1 Department of Medical Oncology, National Center for Tumor Diseases (NCT), Heidelberg University Hospital, Heidelberg, Germany; 2 Else Kroener Fresenius Center for Digital Health, Technical University Dresden, Dresden, Germany; 3 School of Computer Science, University of St Andrews, St Andrews, UK; 4 Department of Medicine II, Medical Faculty Mannheim, Heidelberg University, Mannheim, Germany; 5 Department of Medicine III, University Hospital RWTH Aachen, Aachen, Germany; 6 Department of Medicine I, University Hospital Dresden, Dresden, Germany; 7 Institute of Pathology, University Medical Center Mainz, Mainz, Germany; 8 Department of Gynecology and Center for Hereditary Breast and Ovarian Cancer, University Hospital rechts der Isar, Technical University of Munich (TUM), Munich, Germany; 9 Center for Personalized Medicine (ZPM), University Hospital rechts der Isar, Technical University of Munich (TUM), Munich, Germany; 10 German Cancer Consortium (DKTK), Munich, Germany; 11 European Network for Rare Cancers (EURACAN) Initiative, Munich, Germany; 12 Google Cloud, Munich, Germany; 13 Integrated Pathology Unit, Institute for Cancer Research and Royal Marsden Hospital, London, UK; 14 Department of Epidemiology and Biostatistics, Memorial Sloan Kettering Cancer Center, New York, NY USA; 15 Department of Diagnostic and Interventional Radiology, University Hospital Aachen, Aachen, Germany

**Keywords:** Cancer therapy, Data integration, Cancer, Information technology

## Abstract

Clinical decision-making in oncology is complex, requiring the integration of multimodal data and multidomain expertise. We developed and evaluated an autonomous clinical artificial intelligence (AI) agent leveraging GPT-4 with multimodal precision oncology tools to support personalized clinical decision-making. The system incorporates vision transformers for detecting microsatellite instability and *KRAS* and *BRAF* mutations from histopathology slides, MedSAM for radiological image segmentation and web-based search tools such as OncoKB, PubMed and Google. Evaluated on 20 realistic multimodal patient cases, the AI agent autonomously used appropriate tools with 87.5% accuracy, reached correct clinical conclusions in 91.0% of cases and accurately cited relevant oncology guidelines 75.5% of the time. Compared to GPT-4 alone, the integrated AI agent drastically improved decision-making accuracy from 30.3% to 87.2%. These findings demonstrate that integrating language models with precision oncology and search tools substantially enhances clinical accuracy, establishing a robust foundation for deploying AI-driven personalized oncology support systems.

## Abstract (web summary)

Ferber et al. present an autonomous artificial intelligence agent system for deployment of specialized medical oncology computational tools, validating their system across various clinical scenarios representative of typical patient care workflows.

## Main

The field of large language models (LLMs)^1^ has witnessed remarkable advancements in recent years. Models such as GPT-4 (ref. ^2^) have demonstrated capabilities that closely mimic human reasoning and problem-solving abilities and have shown knowledge across various professional disciplines. In the medical field, for instance, GPT-4 has achieved a passing score on the United States Medical Licensing Examination and is able to provide detailed explanations for its responses^3^. In oncology, where clinical decision-making is increasingly complex, LLMs can serve as a rapid and reliable reference tool, for instance, by providing medical recommendation suggestions from official medical guidelines^4^. This capability could not only aid in daily decision-making processes but also educate oncologists to stay updated with the latest treatment recommendations.

However, similar to the medical domain itself, where doctors rely on information through speech, written text and imaging, the future of medical artificial intelligence (AI) is multimodal^5^. Recently, several such AI systems have been introduced^6^. Examples include models that analyze radiology images together with clinical data^7^ or integrate information from histopathology with genomic^8^ or text-based information^9^. These advancements have fueled anticipations for the advent of generalist multimodal AI systems^10,11^, characterized by their ability to concurrently analyze and reason across any dimension of medical information.

However, it remains to be investigated whether such generalist multipurpose AI models alone are suitable for medical applications. The distribution of human diseases is wide and complex, which is not captured in current performance benchmarks, where these models are predominantly evaluated on a single specific task at a time. In contrast, real-world clinical decision-making often requires multistep reasoning, planning and repeated interactions with data to uncover new insights to make informed and personalized decisions.

Despite advances with models such as Med-PaLM M^11^ or Med-Gemini^12^, the complexities to develop a generalist foundation LLM that truly performs on par with precision medicine tools remain a substantial challenge. Additionally, at present, regulatory policies in the United States and the European Union restrict the approval of a universal multipurpose AI model, given the philosophy that medical devices should fulfill a singular purpose^13^.

Previous work has shown that some of these limitations can partially be overcome by enriching LLMs with domain-specific information. This can be achieved through fine-tuning^14^ or retrieval-augmented generation (RAG)^15^, a process that temporarily enhances an LLM’s knowledge by incorporating relevant text excerpts from authoritative sources into the model, such as medical guidelines^16^ or textbooks. Yet, this strategy, concentrating on augmenting the knowledge base of the models, positions LLMs as mere information extraction tools only, rather than serving as true clinical assistants. Ideally, such a system would engage in reasoning, strategizing and performing actions on patient records and retrieve or synthesize new information to enable customized decision-making. Outside of the medical field, several such autonomous AI systems, also termed agents, have been proposed. Equipping an LLM with a suite of tools, such as calculators or web search, has proven superiority in tasks that require multistep reasoning and planning^17,18^. Similarly, in biomedical research, Arasteh et al. used the integrated data analysis tools of an LLM to analyze scientific data, achieving results on par with human researchers^19^. Such an approach would facilitate the opportunity of accessing the information repositories that currently exist in hospital systems, allowing for a true model for integrated patient care^20^.

In this study, we build and evaluate an AI agent tailored to interact with and draw conclusions from multimodal patient data through tools in oncology. Contrarily to the philosophy of an all-encompassing multimodal generalist foundation model, we see the achievements that specialist unimodal deep learning models have brought to precision medicine^21^ as a viable template even in the future by equipping an LLM, specifically GPT-4, with additional functions and resources. These could be precision oncology deep learning models or the ability to perform web search, all referred to herein as tools. Specifically, this study includes the vision model application programming interface (API) dedicated to generating radiology reports from magnetic resonance imaging (MRI) and computed tomography (CT) scans, MedSAM^22^ for medical image segmentation and in-house developed vision transformer models trained to detect the presence of genetic alterations directly from routine histopathology slides^23^, in particular, to distinguish between tumors with microsatellite instability (MSI) and microsatellite stability (MSS)^24^ and to detect the presence or absence of mutations in *KRAS* and *BRAF*. Additionally, the system encompasses a basic calculator, capabilities for conducting web searches through Google and PubMed, as well as access to the precision oncology database OncoKB^25^. To ground the model’s reasoning on medical evidence, we compile a repository of roughly 6,800 medical documents and clinical scores from a collection of six different official sources, specifically tailored to oncology.

**Fig. 1:**
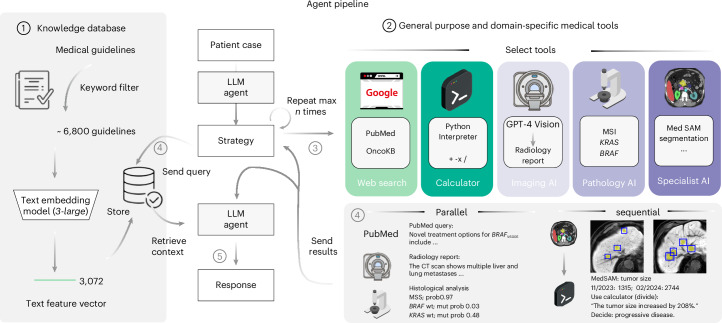
High-level overview of the LLM agent framework. A schematic overview of our LLM agent pipeline. At its core, our system accesses a curated knowledge database comprising medical documents, clinical guidelines and scoring tools. This database is refined from a broader collection through keyword-based search, with the selected documents undergoing text embeddings for efficient storage and retrieval (1). The framework is further augmented with a suite of medical tools, including specialized web search capabilities through platforms such as Google, PubMed and access to the OncoKB API. The agent’s capabilities are further expanded through the integration of a vision model tailored for generating detailed reports from CT and MRI scans, alongside MedSAM, a state-of-the-art medical image segmentation model and access to a simple calculator. Additionally, the system uses vision transformers specifically developed for the prediction of MSI versus MSS and the detection of *KRAS* and *BRAF* mutations in microscopic tumor samples (2). Given a simulated patient case, all tools are selected autonomously by the agent (3) with a maximum of ten per invocation and can be used either in parallel or in a sequential chain (4). This way, the agent can generate relevant patient information on demand and use this knowledge to query relevant documents within its database (4). This enables it to generate a highly specific and patient-focused response that integrates the initial clinical data with newly acquired insights, all while being substantiated by authoritative medical documentation (5). Source data

To quantitatively test the performance of our proposed system, we devise a benchmark strategy on realistic, simulated patient case journeys. Existing biomedical benchmarks and evaluation datasets are designed for one or two data modalities^26^ and are restricted to closed question-and-answer formats. Recent advancements have been made with the introduction of new datasets by Zakka et al.^16^, targeting the enhancement of open-ended responses, and LongHealth^27^, focusing on patient-related content. Yet, these datasets are limited to text and do not capture multimodal data, such as the combination of CT or MRI images with microscopic and genetic data, alongside textual reports. Therefore, in the present study, we develop and assess our agent using a dataset comprising 20 realistic and multidimensional patient cases, which we generate with a focus on gastrointestinal oncology. For each patient case, the agent follows a two-stage process. Upon receiving the clinical vignette and corresponding questions, it autonomously selects and applies relevant tools to derive supplementary insights about the patient’s condition, which is followed by the document retrieval step to base its responses on substantiated medical evidence, duly citing the relevant sources. To evaluate the results, we designed a blinded manual evaluation by four human experts, focusing on three areas: the agent’s use of tools, the quality and completeness of the textual outputs and the precision in providing relevant citations. For effective tool application, the agent must first recognize the utility of a tool, comprehend the necessary inputs and then extract these inputs from the provided patient information. We provide an overview of our entire pipeline in Fig. 1. A detailed description of our methodology is provided in the Methods.

## Results

### Tool use and retrieval improve LLM responses

**Fig. 2:**
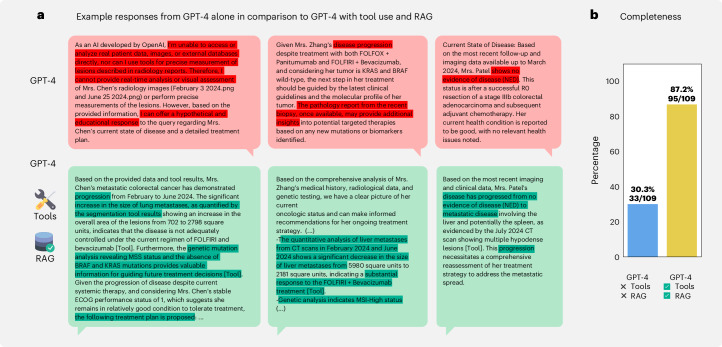
Tool use and RAG improves LLM performance. **a**, Top, to demonstrate the superiority of our approach compared to a standard LLM, we highlight three cases where GPT-4 without tool use either fails to detect the current state of the disease for a given patient or provides very generic responses. Bottom, in contrast, tool access and retrieval enable the model to provide detailed information, such as measuring tumor surface and making appropriate decisions. **b**, The performance comparison shows a higher fraction of responses being evaluated as complete on our completeness benchmark for the agent with tool use and RAG versus GPT-4 alone. Source data

To first demonstrate the superiority of combining medical tools and retrieval with an LLM, we compared our agent to GPT-4 alone. As an illustration, in Fig. 2a, we highlight three examples where tools and retrieval enabled the LLM to accurately solve cases, whereas, without these enhancements, GPT-4 either stated that it could not solve the patient case and provided hypothetical answers instead or drew incorrect conclusions, such as falsely assuming ‘disease progression’ or ‘no evidence of disease’ (in red). In contrast, the agent, by using tools, correctly identified treatment response and the presence of disease progression (in green). To quantify this, we assessed the model’s ability to develop a comprehensive treatment plan for each patient, specifying appropriate therapies on the basis of recognizing disease progression, response or stability, mutational profile and all other relevant information, much like an oncologist would. Therefore, we compiled a set of 109 statements (completeness) for 20 different patient cases. We show that GPT-4 alone only provided 30.3% of the expected answers. However, our agent’s responses achieved a 87.2% success rate, with only 14 instances not being covered (Fig. 2b). Overall, our results demonstrate that enhancing LLMs with tools drastically improves their ability to generate precise solutions for complex, realistic medical cases instead of providing only generic or even wrong responses when using LLMs in an out-of-the-box manner.

### GPT-4 handles complex chains of tool use

**Fig. 3:**
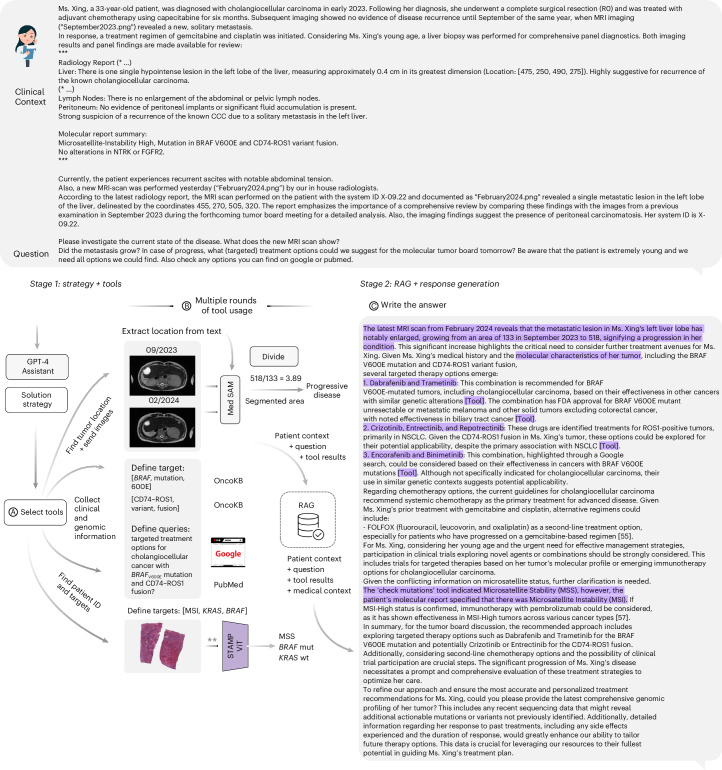
Details of the agent’s pipeline in patient case evaluation. The full agent’s pipeline for the simulated patient X, showcasing the complete input process and the collection of tools deployed by the agent. We abridge the patient description for readability (* …). The complete text is available in Supplementary Note 1. **a**,**b**, In the initial ‘tools’ phase, the model identifies tumor localization from patient data and uses MedSAM for the generation of segmentation masks. Measuring the area of the segmented region enables the calculation of tumor progression over time as the model calculates an increase by a factor of 3.89. The agent also references the OncoKB database for mutation information from the patient’s context (*BRAF*^V600E^ and *CD74–ROS1*) and performs literature searches through PubMed and Google. For histological modeling, we must note here that we streamlined the processing. The original STAMP pipeline consists of two steps, where the first is the timely and computationally intensive calculation of feature vectors, which we performed beforehand for convenience. The second step is performed by the agent by selecting targets of interest and the location of the patient’s data and executing the respective vision transformer (**). **c**, The subsequent phase involves data retrieval through RAG and the production of the final response. Source data

We next investigated the overall capabilities and limitations of our agent across a panel of benchmarks in greater depth. To provide better guidance, we present one complete, simulated patient case and detail every step, from input over tool usage to the model’s final output in Fig. 3. All tasks for the model and all answers are shown in Supplementary Note 1.

**Fig. 4:**
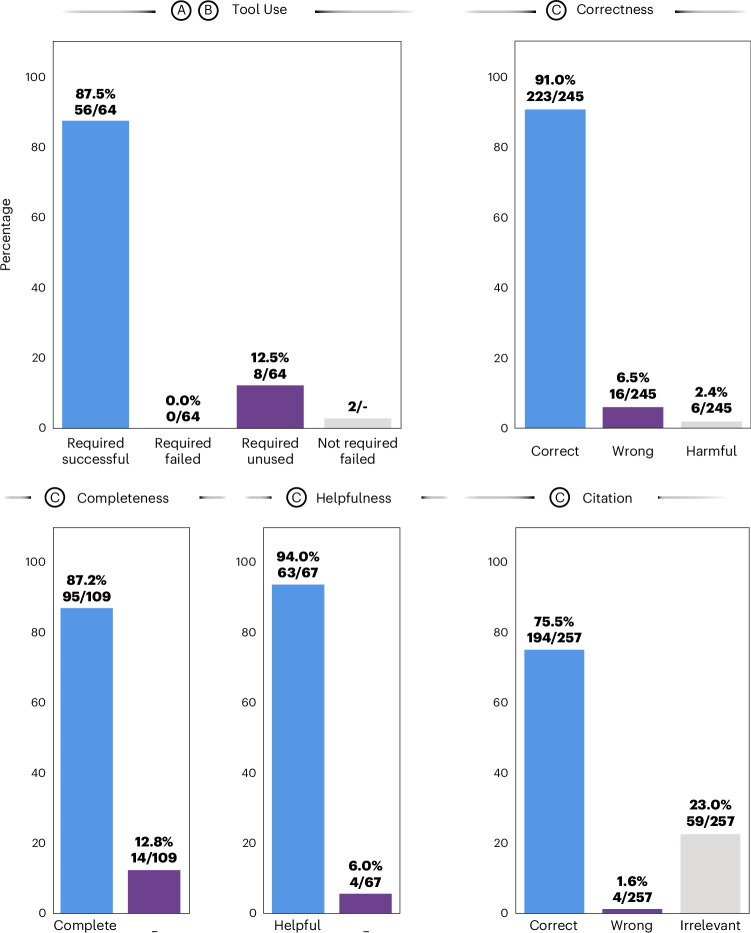
Performance of the agent’s pipeline in patient case evaluation. Results from benchmarking the LLM agent through manual evaluation conducted by a panel of four medical experts. **a**–**c**, Steps in the agent’s workflow as outlined in Fig. 3. For the metric ‘tool use’, we report four ratios: represents the proportion of tools that were expected to be used to solve a patient case and that ran successfully (56/64), with no failures among the required tools. Required/unused (8/64) are tools that the LLM agent did not use despite being considered necessary. Additionally, there are two instances where a tool that was not required was used, resulting in failures. ‘Correctness’ (223/245), ‘wrongness’ (16/245) and ‘harmfulness’ (6/245) represent the respective ratios of accurate, incorrect (yet not detrimental) and damaging (harmful) responses relative to the total number of responses. Here, a response is constituted by individual paragraphs per answer. ‘Completeness’ (95/109) measures the proportion of experts’ expected answers, as predetermined by keywords, that the model accurately identifies or proposes. ‘Helpfulness’ quantifies the ratio of subquestions the model actually answers to all questions or instructions given by the user (63/67). Lastly, we measure whether a provided reference is correct (194/257), irrelevant (59/257, where the reference’s content does not mirror the model’s statement) or wrong (4/257). Results shown here are obtained from the majority vote across all observers, selecting the least favorable response in cases of a tie. Source data

First, we evaluated the agent’s ability to recognize and successfully use tools (Fig. 4). Of 64 required tool invocations to fully solve all given patient cases, the agent correctly used 56, achieving an overall success rate of 87.5%, with no failures among the required tools. The remaining 12.5% of tools were required but missed by the model. There were two instances where the model tried to call superfluous tools without the necessary data available, which resulted in failures (Fig. 4, ‘tool use’). We provide an overview of each tool’s invocation status in Supplementary Table 1. To provide an illustrative example, in the patient case from Fig. 3, GPT-4 used its tools to evaluate the patient’s conditions by first identifying tumor localization from patient data and generating segmentation masks with MedSAM. It calculated tumor progression by measuring the segmented area’s growth and referenced the OncoKB database for mutation information. The model then performed literature searches and histological modeling to select targets of interest, ultimately producing the final response through data retrieval with RAG.

**Fig. 5:**
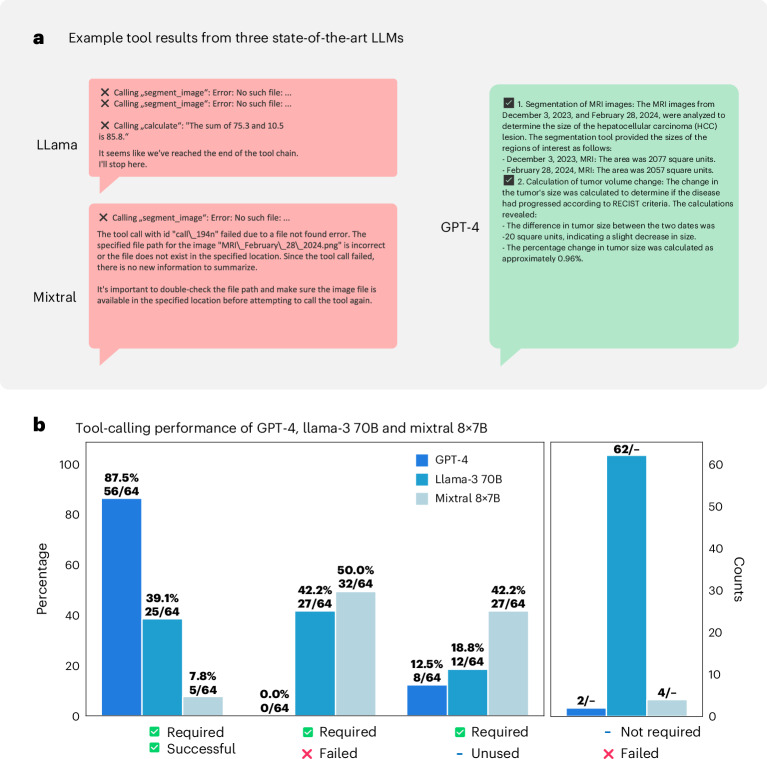
Benchmarking of tool use for Llama-3 70B, Mixtral 8x7B (both open-weight models) and GPT-4 (proprietary). **a**, Example tool results from three state-of-the-art LLMs (Llama-3, Mixtral and GPT-4). While the former two demonstrate failures in calling tools (or performing meaningless superfluous calculations in the case of Llama), GPT-4 successfully uses image segmentation on the MRI images and uses the calculator to calculate tumor changes in size. **b**, Tool benchmarking calling performance for these three models in a similar fashion to Fig. 4. Overall, our findings reveal that both open-weight models demonstrate only extremely poor function-calling performance. First, both models struggle to identify necessary tools for a given patient context (18.8% of required tools remain unused by Llama and even 42.2% for Mistral). Next, even in instances where the correct tool was identified, the model frequently failed to supply the necessary and accurate function arguments (‘required, failed’). This deficiency results in invalid requests that disrupt program functionality (Llama, 42.2%; Mixtral, 50.0%), ultimately leading to crashes. We saw none of these cases for GPT-4. Consequently, for Llama and Mixtral, the overall success rates were low, registering only 39.1% (Llama) and 7.8% (Mixtral) (‘required, successful’). Moreover, we saw that the Llama model frequently used superfluous tools, for example, performing random calculations on nonsense values or hallucinating (inventing) tumor locations during imaging analysis that did not exist. This led to 62 unnecessary tool calls and failures (‘not required, failed’) across all 20 patient cases evaluated. The major shortcoming of the Mixtral model was its frequent disregard for tool use, resulting in fewer than one in ten tools running successfully. Source data

We additionally benchmarked GPT-4 against two state-of-the-art open-weights models, Llama-3 70B (Meta)^28^ and Mixtral 8x7B (Mistral)^29^, for which we show exemplary results in Fig. 5a and quantitative evaluations in Fig. 5b. We provide results for each patient in Supplementary Tables 2 and 3. Given substantial shortcomings in the latter two, we decided to only focus on GPT-4 as it demonstrated reliable and highly effective performance in identifying relevant tools and applying them correctly to patient cases.

Additionally, we observed that GPT-4 is capable of chaining sequential tool calls, using the output from one tool as input for the next. For example, in the tool results for patient G in Supplementary Note 2, GPT-4 invoked the MedSAM tool twice to obtain segmentation masks from two images taken at different time points. Subsequently, GPT-4 used the calculator function with the appropriate values from MedSAM to determine that the tumor had grown by a factor of 2.14. As another example, for patient W (Supplementary Note 2), the model used vision transformer models to assess the patient’s mutational status, confirming the presence of a suspected *BRAF* mutation. The model then queried the OncoKB database to retrieve medical information on appropriate management regarding the mutation.

**Extended Data Fig. 1:**
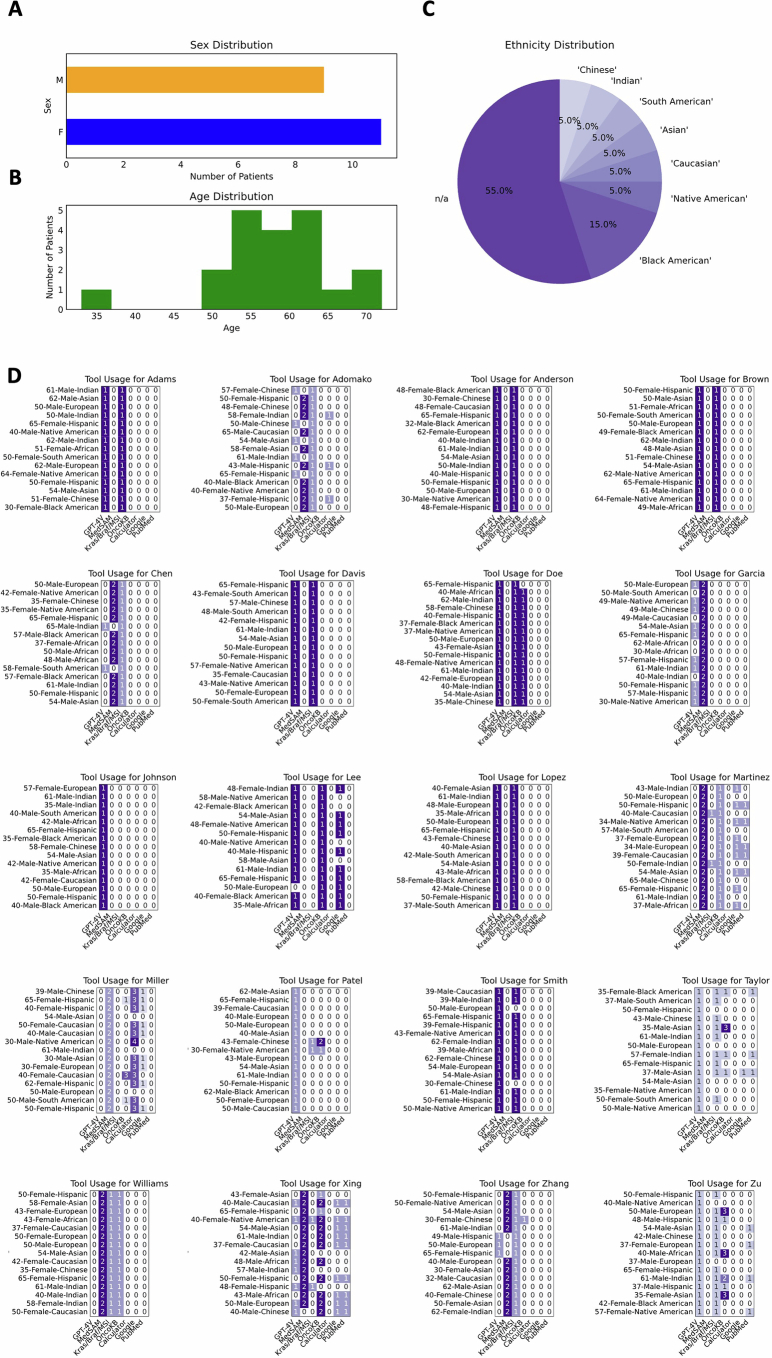
Patient case population and bias investigation. We show more details on the simulated patient cases from our benchmarking experiments including the sex (**A**) and age (**B**) distribution for all 20 cases. The pie chart in **C** shows the proportions of patient origins by country and ethnicity. For 55% of the patients, we did not provide this information in the patient case vignette (n/a), while the remaining 45% included diverse information on patient origin. (**D**) To investigate whether gender, age, and origin influence the models’ tool-calling behavior, we conducted an additional experiment with 15 random permutations on all 20 patient cases (300 in total). Notably, we observed that in contrary to patient cases requiring relatively fewer tools (for example, patients Adams, Lopez and Williams), there was higher variability in tool-calling behavior in situations requiring more tools (for example, patient Ms Xing), regardless of the combinations of age, sex, and ethnicity/origin. Heatmaps are annotated on the x-axis as ‘age-sex-ethnicity/country of origin’.

Additionally, we assessed the robustness of the agent across multiple repetitions and diverse patient populations, specifically considering different combinations in sex (Extended Data Fig. 1a), age (Extended Data Fig. 1b) and origin (Extended Data Fig. 1c). Our evaluation showed that the primary source of performance variation was the number of tools required for each patient case, rather than intrinsic patient factors (Extended Data Fig. 1d).

In summary, we can demonstrate that GPT-4 can effectively manage complex scenarios by sequentially using multiple tools, integrating their results and drawing informed conclusions on subsequent tool usage on the basis of prior information.

### Radiology tools improve GPT-4’s treatment accuracy

Second, we investigated the use of radiology processing tools by the agent. In our scenario, GPT-4 had two options: using the GPT-4 Vision (GPT-4V) API to generate comprehensive radiology reports (useful when no radiology report was provided for a patient) or using MedSAM for image segmentation to generate surface segmentation masks of the described tumor reference lesions when localizations of reference lesions were included in the patient case vignette. For both options, the model first had to identify the location where the images were stored on the filesystem, extract the order in which the images were taken (by date, indicated in the image file names) and send them to the appropriate tool. In our evaluations, we found that, despite occasional omissions, extraneous details, lack of information or making mistakes (highlighted in red in Supplementary Note 2), GPT-4V could nonetheless effectively guide the agent’s decision.

Similarly, for MedSAM as a tool, the expected workflow for GPT-4 involved first locating the relevant patient images on the system, identifying their chronological order and receiving the locations of described reference lesions from the patient case vignette. After sending a request to MedSAM and receiving the results (tumor segmentation sizes), the model was usually expected to use the calculator to determine the percentage change according to RECIST criteria. This process is schematically illustrated in Fig. 3, with a detailed example provided in Supplementary Note 2 for patient G. We show that, in all instances, MedSAM received information on all relevant tumor locations and returned results that were helpful to GPT-4 to determine the appropriate next steps on the basis of calculating whether the patient showed progressive disease or remained in a stable condition.

In summary, we show that our pipeline is able to autonomously handle multiple steps, such as determining the need for specific tools, locating the relevant data, understanding their chronological order, sending requests to the appropriate tool, receiving results and integrating these into the next steps of its decision-making, for instance, using a calculator to compare tumor sizes over time. All of these steps are managed fully autonomously, without human supervision, by the LLM agent.

### Evaluations show accurate, helpful and reliable responses

Thirdly, a central point in our analysis was the assessment of response accuracy (correctness). To evaluate this, we segmented the responses into smaller, evaluable items on the basis of the appearance of citations or transitions in topic within subsequent sentences, resulting in a total of 245 assessable statements. Our evaluations found 223 (91.0%) of these to be factually correct and 16 (6.5%) to be incorrect, while 6 (2.4%) were flagged as potentially harmful. Detailed instances of erroneous and harmful responses are provided in Supplementary Table 4 for review.

Remarkably, the agent was capable of resolving issues, even in instances where contradictory information was provided in a patient’s description, such as discrepancies in reported mutations versus results of testing such mutations using tools. In such cases, the agent pointed out these inconsistencies, recommended further genetic confirmation and outlined potential treatment options based on the results (patients D and X). An illustrative example of the model’s response is presented in Fig. 3.

Next, we assessed the degree of helpfulness of the agent by evaluating the proportion of subquestions it answered sophistically according to the human evaluators. Among the aggregate of 67 queries, 63 (94.0%) were categorized as having been effectively addressed.

Fourth, aiming to ensure transparency in the decision-making process, we instigated its adherence to citing relevant sources. Through manual review, we determined that, of the 257 citations provided in the models’ responses, 194 (75.5%) were accurately aligned with the model’s assertions, while 59 (23.0%) were found to be unrelated and merely 4 (1.6%) were found to be in conflict with the model’s statement. These findings are promising, highlighting that instances of erroneous extrapolation (so-termed hallucinations) by the model are limited. Supplementary Notes 2 and 3 respectively display the unprocessed complete results from using the tools and the entire model outputs. Detailed evaluation results from each human observer are elaborated on in Supplementary Tables 4–6. Evaluation results for GPT-4 without tools and retrieval that we benchmarked for completeness can be found in Supplementary Table 7 and Supplementary Note 3.

Overall, our results show that GPT-4 with tools and RAG can deliver highly accurate, helpful and well-cited responses, which enhances its ability to handle complex medical scenarios and provide support for clinical decision-making.

## Discussion

Our results demonstrate that combining precision medicine tools with an LLM enhances its capabilities in problem solving, aligning with the concept of using LLMs as ‘reasoning engines’ (ref. ^30^). As we have shown, GPT-4 alone only generates very generic or wrong responses. However, integrating three core elements (a reasoning engine, a knowledge database and tools) enables us to effectively address this limitation, greatly improving accuracy and reliability. Additionally, using tools has several further advantages. Despite the potential future development of a generalist medical multimodal foundation model^10^, its efficacy in addressing very specialized medical queries, such as predicting rare mutations or measuring disease development on the millimeter scale compared to narrower, domain-specific models, remains uncertain. Moreover, maintaining the alignment of such a generalist model with the evolving medical knowledge and updates in treatment guidelines is challenging, as it requires retraining model components on new data. Our tool-based approach, however, addresses all of these issues. It allows for the rapid update of medical knowledge by simply replacing documents in the database or searches through Google or PubMed, eliminating the need for direct modifications to the core model itself. Similarly, state-of-the-art medical devices that are approved by regulatory authorities can be included in our setup. Additionally, by using RAG, our system effectively addresses a major limitation in current LLMs, which often tend to hallucinate by providing wrong but seemingly correct or plausible answers to questions beyond their knowledge^4^. Mitigating this issue is particularly critical in sensitive domains such as healthcare. We show that our approach demonstrates a high capability in delivering accurate answers, supported by relevant citations from authoritative sources, which also facilitates quick fact-checking as the agent returns the exact original text from the guideline documents.

Our work has several limitations. The most notable is the small sample size for evaluation. Constructing these cases from real-world data requires careful manual crafting and evaluation while adhering to data protection standards, especially for cloud-based processing. We, therefore, consider our work as a proof-of-concept study for agentic AI workflows in oncology and encourage future efforts to develop larger-scale benchmark cases.

An important aspect beyond the scope of our study, which should be addressed in future research, is the selection of medical tools. While the core focus of our work lies in the tool-using abilities of language model agents, it is essential to recognize that individual tools require better independent optimization and validation. For example, we do not possess annotated ground-truth segmentation masks for comparison to MedSAM but use clinical endpoints such as progressive disease or tumor shrinkage as our primary metric to measure outcomes, which even more closely reflects the clinical workflow. As another example, vision transformers in our study were trained and tested on data from The Cancer Genome Atlas (TCGA) and some radiology images available online may have been included in the pretraining of GPT-4V. In a production environment, however, such tools would simply be replaced with better-validated and extensively tested alternatives, such as the clinical-grade MSIntuit model^31^. Overall, we believe that, by prioritizing clinical endpoints to validate our agent pipeline, we can offer new, directly relevant evidence and metrics to address the current scarcity of objective measures for AI agents in healthcare.

Regarding the agent, although equipped with a broad array of tools compared to other frameworks^17^, it remains in an experimental stage, thus limiting clinical applicability. One notable restriction for instance lies in the provision of only a singular slice of radiology images and the yet limited capabilities of GPT-4V (ref. ^32^) in interpreting medical images. We anticipate great progress in generalist foundation models with greatly enhanced capabilities in interpreting three-dimensional (3D) images, including medical images. In this regard, we expect the development of more advanced, specialized medical image–text models, similar to the recently introduced Merlin model for 3D CT imaging^33^, which would allow adding these models as additional tools instead of simple ones such as MedSAM into our pipeline. Furthermore, an image–text model for radiology could also incorporate information on patient history and previous therapies to evaluate the current state of the disease like a radiologist would, instead of relying on lesion size changes only.

Moreover, our current agent architecture is a static design choice. Medical professionals, however, have access to an extensive array of complex tools and can alternate between tool usage and knowledge retrieval. For AI agents, integrating RAG and tool use concurrently could also be complementary, as RAG could assist in guiding the agent through complicated steps of tool application for specific diagnostic or treatment decisions. This synergy might help improve complex workflows with more and more challenging tools, which remains an area of further exploration.

Additionally, despite being implemented as a multiturn chat agent, our evaluation is currently confined to a single interaction. As a next step, we aim to incorporate multiturn conversations, including human feedback for refinement, akin to a ‘human-in-the-loop’ concept^34^.

Furthermore, we restricted our patient scenarios to oncological use cases only, yet it is important to note that the underlying framework could be adapted to virtually any medical specialty, given the appropriate tools and data.

Next, regarding data protection in real-world settings, current regulatory restrictions make GPT-4 unsuitable because of its cloud-based nature, which necessitates transferring sensitive patient data to proprietary commercial servers. Consequently, we regard GPT-4 as a best-in-class model for proof-of-concept purposes and aim to explore open-weight models that can be deployed on local servers in the near future. Newer and better models, especially Llama-3 405B (ref. ^35^), might be promising candidates.

Moreover, medically fine-tuned models or models broadly optimized for tool use, such as Hermes-3-Llama-3.1 (ref. ^36^), also show considerable potential as local solutions.

Additionally, while our model receives relevant context from the RAG pipeline to provide accurate citations, there are several modifications and potential improvements to the retrieval process that we suggest exploring in future work. For instance, we used generalist embedding, retrieval and reranking models, whereas Xiong et al.^37^ demonstrated that domain-specific models can enhance retrieval performance. Moreover, for rare terms (such as rare diseases), exact match keyword searches can outperform similarity searches in embedding space and both can even be combined (also termed hybrid search^38^).

Moreover, modeling long context dependencies could be further improved by using models with larger context windows, such as Gemini 1.5 (ref. ^39^). This capability is especially crucial when scaling our approach to settings where patient information is not restricted to a single one-page case vignette but distributed across hundreds of documents.

Lastly, from a production setting perspective, another critical aspect is to consider how the LLM handles temporal dependencies in treatment recommendations. For instance, in lung cancer, molecular targeted treatments are subject to rapid changes and official guidelines are not always updated at the same pace as the latest trial results. In such a setting, our multitool agent could cross-reference information from official medical guidelines with more up-to-date information received through internet and PubMed searches. Previous work already showed that LLMs can reliably identify temporal differences in medical documents^4^.

Looking ahead, we anticipate more progress in the development of AI agents with even improved capabilities through further scaling. As a parallel step in this direction, Zhou et al.^40^ developed MedVersa, an AI system for medical image interpretation that leverages an LLM as a central ‘orchestrator’. This model is fully trainable to determine whether tasks can be completed independently or should be delegated to a specialist vision model and has demonstrated superior performance across multiple medical imaging benchmarks, often surpassing state-of-the-art models. The training of such a system holds immense potential, as it might allow models to learn critical concepts such as uncertainty, enabling them to recognize their limitations and delegate tasks to specialist models whenever appropriate. Although our model currently shows a strong ability to use given tools, task-specific fine-tuning and few-shot prompting^41^ (providing examples to the model) could further improve performance, particularly when increasing complexity with the addition of more and more complex tools. Additionally, we could enhance the system by incorporating human feedback on edge cases where the initial model performed poorly, similar to a human-in-the-loop approach. In the medical context, this method was already shown to improve prediction accuracy, as demonstrated in the context of molecular tumor board recommendations^42^.

To summarize, in the near future, we envision a framework that embodies characteristics akin to the GMAI model with the added ability to access precision medicine tools tailored to answer any kind of specialized clinical questions. This approach has multiple benefits. It enables medical AI models to assist clinicians in solving real-world patient scenarios using precision medicine tools, each tailored to specific tasks. Such a strategy facilitates circumventing data availability constraints inherent in the medical domain, where data are not uniformly accessible across all disciplines, preventing a singular entity from developing an all-encompassing foundational model. Instead, entities can leverage smaller, specialized models developed by those with direct access to the respective data, which could greatly improve discovering therapeutic options for personalized treatments. Moreover, this modular approach allows for individual validation, updating and regulatory compliance of each tool. In cases where existing tools are unsatisfactory or completely absent, the agent could rely on its internal strong medical domain knowledge and either refine^43^ or innovate entirely new tools from scratch. Crucially, this modular approach also offers far superior explainability compared to a large, generalist black-box model, as physicians can investigate the output from each individual tool separately. Herein, our study could serve as a blueprint, providing evidence that agent-based generalist medical AI is within reach.

To fully leverage the potential of AI agents in the near future, it will be crucial to integrate them as deeply into routine clinical practice as possible, ideally through direct incorporation into existing clinical information systems for live access to patient data with only minimal workflow disruption for clinicians. However, this first necessitates addressing many deployment challenges, including concerns regarding interoperability with existing systems, data privacy laws such as GDPR and HIPAA (in case of cloud-based models), liability and accountability issues and the need for regulatory approvals as medical devices^44^, which will also require much broader validation studies to show safety and benefits in actual clinical workflows. Lastly, there will be a critical need to educate medical professionals to effectively collaborate with AI agents while maintaining full authority over the final clinical decision-making.

## Methods

This study did not include confidential patient information. All research procedures were conducted exclusively on publicly accessible, anonymized patient data and in accordance with the Declaration of Helsinki, maintaining all relevant ethical standards. The overall analysis was approved by the Ethics Commission of the Medical Faculty of the Technical University Dresden (BO-EK-444102022).

### Dataset composition and data collection

The pipeline’s primary goal is to compile a comprehensive dataset from high-quality medical sources, ensuring three main components: correctness, up-to-dateness and contextual relevance, with a particular emphasis on including knowledge across all medical domains while additionally encompassing information specifically tailored to oncology. We restricted our data access to six sources: MDCalc (https://www.mdcalc.com/) for clinical scores, UpToDate and MEDITRON^14^ for general-purpose medical recommendations and the Clinical Practice Guidelines from the American Society of Clinical Oncology^45^, the European Society of Medical Oncology and the German and English subset of the Onkopedia guidelines from the German Society for Hematology and Medical Oncology. We retrieved and downloaded the relevant documents as either HTML extracted text or raw PDF files. To reduce the number of documents for the embedding step, we applied a keyword-based filtering of the document contents, targeting terms relevant to our specific use case. Medical guidelines that were obtained from the MEDITRON project were directly accessible as preprocessed jsonlines files.

### Information extraction and data curation from PDF files

The critical challenge in text extraction from PDF documents arises from the inherent nature of PDF files, which are organized primarily for the user’s ease of reading and display while not adhering to a consistent hierarchical structure, thus complicating the extraction process. For instance, upon text mining with conventional tools such as PyPDF2 or PyMuPDF, headers, subheaders and key information from the main text may be irregularly placed, with titles occasionally embedded within paragraphs and critical data abruptly interspersed within unrelated text. However, maintaining the integrity of the original document’s structure is crucial in the medical field to ensure that extracted information remains contextually coherent, preventing any conflation or misinterpretation. To overcome these limitations, we used GROBID (generation of bibliographic data), a Java application and machine learning library specifically developed for the conversion of unstructured PDF data into a standardized TEI^46^ format. Through its particular training on scientific and technical articles, GROBID enables the effective parsing of medical documents, preserving text hierarchy and generating essential metadata such as document and journal titles, authorship, pagination, publication dates and download URLs.

We next programmatically retrieved the raw document text from the generated XML fields in the TEI files, concurrently implementing data cleansing. This process encompasses the removal of extraneous and irrelevant information such as hyperlinks, graphical elements and tabular data that was corrupted during the extraction with GROBID, as well as any malformed characters or data such as inadvertently extracted IP addresses. The diversity of source materials presented a further challenge because of their varied formatting schemes. To address this, we meticulously reformatted and standardized the text from all sources, denoting headers with a preceding hash symbol (#) and inserting blank lines for the separation of paragraphs. The purified text, along with its corresponding metadata, was archived in jsonlines format for subsequent processing.

### Agent composition: RAG

Below, we delineate the detailed architecture of our agent in a two-step process, beginning with the creation of our RAG^15^ database, followed by an overview of the agent’s tool use and concluding with an examination of the final retrieval and response generation modules. Additionally, we highlight the structure of our model in detail in Supplementary Note 4.

### Embedding creation and indexing

We leveraged RAG to synergize the generative capabilities of LLMs with document retrieval to provide domain-specific medical knowledge (context) to a model. The RAG framework has greatly evolved in complexity recently; thus, we break down its architecture into three major components (embeddings, indexing and retrieval) and outline the implementation details of the first two in the next section. In RAG, we begin with the conversion of raw text data into numerical (vector) representations, also termed embeddings, which are consequently stored in a vector database alongside metadata and the corresponding original text (indexing). In more detail, we compute vector embeddings using OpenAI’s ‘text-embedding-3-large’ model from text segments of varying lengths (512, 256 and 128 tokens), each featuring a 50-token overlap, from the curated guideline cleaned main texts in our dataset, alongside their associated metadata for potential filtering operations. For storage, we use an instance of a local vector database (Chroma) that also facilitates highly efficient lookup operations using vector-based similarity measures such as cosine similarity (dense retrieval). We store documents from different sources in the same collection.

### Agent composition: tools

To endow the LLM with agentic capabilities, we equipped it with an array of tools, including the ability to conduct web searches through the Google custom search API and formulate custom PubMed queries. Information retrieved through Google search underwent text extraction and purification and was integrated directly as context within the model, while responses from PubMed were processed akin to the above-described RAG procedure in a separate database.

For the interpretation of visual data, such as CT or MRI scans, the LLM agent has access to two different tools. It can either call the GPT-4 Vision (GPT-4V) model, which is instructed to generate a comprehensive, detailed and structured report, or use MedSAM, which we explain in detail below. For both tasks, we primarily use representative slices from in-house CT and MRI chest and abdomen series, although a few cases are from public datasets, as highlighted in Supplementary Note 1. Our in-house slices were selected by an experienced radiologist from our team, who was blinded to the rest of the study at the time of image selection. In scenarios involving multiple images to model disease development over time, the model first investigates and reports on each image separately before synthesizing a comparative analysis. Because of the stringent adherence of OpenAI to ethical guidelines, particularly concerning the management of medical image data, we framed our patient cases as hypothetical scenarios when presenting them to the model. However, instances of refusal still arose, prompting us to discard the respective run entirely and initiate a new one from the beginning. Additionally, tasks that require the ability of the model to precisely measure the size of lesions and compare them over time can be solved using MedSAM^22^. MedSAM produces a segmentation mask for a given region of interest on the basis of an image and pixel-wise bounding box coordinates, enabling the calculation of the overall surface area. Herein, MedSAM processes each image independently; however, GPT-4 can track the results from MedSAM back to the original image date, as it has access to image file names that are based on date. This capability ensures accurate decision-making of lesion development over time. An example of this is demonstrated in Supplementary Note 2 under tool results for patient G. A potential limitation of the vision tool approach is that, for both scenarios, we currently restrict the use to single-slice images that additionally need to be taken at the same magnification.

Moreover, we provide access to a simplified calculation tool that allows elementary arithmetic operations such as addition, subtraction, multiplication and division by executing code locally using the Python interpreter.

To facilitate addressing queries related to precision oncology, the LLM leverages the OncoKB^25^ database to access critical information on medical evidence for treating a vast panel of genetic anomalies, including mutations, copy-number alterations and structural rearrangements. More specifically, GPT-4 can send the HUGO symbol, the change of interest (mutation, amplification or variant) and a specific alteration of interest (such as *BRAF*^V600E^) if applicable to the OncoKB API that returns a structured JSON object containing potential Food and Drug Administration-approved or investigational drug options including evidence levels. This enables querying any type of genetic arrangement, given it is listed on OncoKB and an appropriate license is acknowledged by the user.

Lastly, GPT-4 is also equipped to engage specialized vision transformer models for the histopathological analysis of phenotypic alterations underlying MSI^24^ or *KRAS* and *BRAF* mutations. For the *KRAS* and *BRAF* mutation prediction models, we used a setup similar to Wagner et al.^23^. These models were obtained by training a vision classifier model using histopathology features extracted from images of colorectal cancer tissue from TCGA. For feature extraction, we used CTranspath^47^, a state-of-the-art transformer model trained through self-supervised learning. To optimize time and computational resources, features were pre-extracted with CTranspath, which is a common procedure in computational pathology pipelines^48^. Consequently, instead of the original images, GPT-4 transmits these pre-extracted features to the MSI, KRAS, and BRAF vision transformers; however, it is important to note that this implementation detail is hidden from GPT-4 and, thus, has no influence on our overall pipeline. It, however, allows us to reduce the time and hardware requirements for each run in a research setting. For deployment purposes, one could easily, without making changes to the LLM agent itself, hide additional logic to extract features on the fly from the original images.

During each invocation, GPT-4 has to determine the availability of histopathology images for a patient, locate them within the system and select the targets (one, two or all three) for testing. It then receives a binary prediction (MSI versus MSS, *KRAS* mutant versus wild type or *BRAF* mutant versus wild type) along with the mutation probability in percentage.

All necessary information for calling the designated tools is derivable or producible from the given patient context. Unlike the retrieval phase, which we manually enforced at each invocation, the decision regarding the use and timing of tools is dependent only on the agent’s reasoning. However, manual intervention to prompt tool usage is possible, as demonstrated in patients D and X. The specifications for all tools are delineated in JSON format, which is provided to the model and encompasses a brief textual description of each tool’s function along with the required input parameters. From a procedural point of view, given an input comprising a variable-length textual patient context and a text query, the agent generates an initial action plan, followed by a series of iterative tool applications. The deployment of these tools can be executed either independently in parallel or sequentially, wherein the output from one tool serves as the input for another in subsequent rounds; for instance, the size of the segmentation areas obtained from two images through MedSAM can be used to compute a ratio and, thus, define disease progression, stability or response, as shown schematically in Fig. 1 and the patient case from Fig. 3.

### Agent composition: combine, retrieve and generate responses

The final retrieval and response generation pipeline is implemented using DSPy^49^, a library that allows for a modular composition of LLM calls. All instructions to the model can be found in our official GitHub repository. Firstly, the model receives the original patient context, the posed question and the outcomes from the tool applications as input. In a method similar to that described by Xiong et al.^50^, we used chain-of-thought reasoning^51^ to let the model decompose the initial user query into up to 12 more granular subqueries derived from both the initial patient context and the outcomes from tool applications. This facilitates the retrieval of documents from the vector database that more closely align with each aspect of a multifaceted user query. Precisely, for each generated subquery we extract the top *k* most analogous document passages from the collection. Subsequently, these data are combined, deduplicated, reranked and finally forwarded to the LLM. We highlight this process in more detail as pseudocode in Supplementary Note 4. Each request sent by the LLM to the RAG pipeline is transformed into a numerical representation using the same embedding model applied to the medical guidelines. We next use cosine distance (lower is better) to compare the query with any embedded chunk from the medical guidelines in the vector database, sorting in ascending order to retrieve the top 40 vectors, each mapped to their respective original text passage. Then, we use Cohere’s reranking model (Cohere Rerank 3 English) to reorder the retrieved text passages on the basis of their semantic similarity to the LLM’s query. This step filters out passages that exhibit falsely high similarity (low distance) in embedding space but are contextually irrelevant. For example, a query such as ‘Which drug is approved for NSCLC?’ and text from a guideline saying ‘Drug A is not approved for non-small cell lung cancer’ may show high cosine similarity (low distance), yet the former is irrelevant to the query. The reranking step helps to rank such passages lower. From the reranked results, we keep only the top ten relevant passages and repeat this entire process for each subquery (subtopic) generated by the model on the basis of the provided patient information. To reduce token usage, we remove duplicates from the entire collection of guideline text chunks, prefixed each with an enumeration of ‘Source [x]: …’ to allow for accurate citations and then sent the data back to the LLM.

Before generating the final answer, we instruct the LLM to generate a step-by-step strategy to build a structured response including identifying missing information that could help refine and personalize the recommendations. The resulting model output is then synthesized on the basis of all available information, strictly following the strategy as a hierarchical blueprint. To enhance the system’s reliability and enable thorough fact-checking, both of which are fundamental in real-world medical applications, the model was programmatically configured to incorporate citations for each statement (as defined as a maximum of two consecutive sentences) using DSPy suggestions^49^. On the implementation level, the LLM performs a self-evaluation step, wherein it compares its own output to the respective context from our database in a window of one to two sentences. We perform a single iteration over this procedure. All prompts are implemented using DSPy’s signatures.

### LLM tool use benchmarking: Llama-3 70B and Mixtral 8x7B

We additionally performed comparisons of GPT-4 with two state-of-the-art open-weight LLMs, namely Llama-3 70B from Meta AI^28^ and Mixtral 8x7B from Mistral^29^. On the basis of initial evidence from testing, we slightly simplified and shortened the original prompt from GPT-4 but left all other parameters with respect to the original tool composition unaltered. We evaluated the performance of function calling against GPT-4 on the 20 patient cases, reporting the following metrics: the fraction of required tool calls that were completed successfully (required/successful) and those that were required but failed (required/failed). Additionally, we assessed the fraction of necessary tool calls that the model failed to invoke (required/unused). Lastly, we measured the ratio of superfluous tool calls that the models invoked and that failed (not required/failed).

### Model specifications

In our study, we consistently used the following models through the official OpenAI Python API for all experiments. The core framework for the agent and all tools involving an LLM is the gpt-4-0125-preview model (GPT-4). For tasks requiring visual processing, the gpt-4-vision-preview (GPT-4V) model was used through the chat completions endpoint. The temperature value for both models was empirically set to 0.2 for the agent phase and 0.1 during RAG upon initial experimentation and no further modifications of model hyperparameters were performed. Additionally, for generating text embeddings, we used the latest version of OpenAI’s embedding models, specifically the text-embedding-3-large model, which produces embeddings with a dimensionality of 3,072. To establish a baseline for comparison without tools and retrieval, we also evaluated GPT-4 with identical hyperparameters using a chain-of-thought reasoning module. Additionally, for model benchmarking, we used the Meta Llama-3 70B model (llama3-70b-8192) and Mistral’s Mixtral 8x7B model (mixtral-8x7b-32768) through the Groq API, setting temperature values to 0.2 and the maximum number of output tokens to 4096.

### Clinical case generation

To address the limitations in current biomedical benchmarks, we compiled a collection of 20 distinct multimodal patient cases, primarily focusing on gastrointestinal oncology, including colorectal, pancreatic, cholangiocellular and hepatocellular cancers. Each case provided a comprehensive but entirely fictional patient profile, which includes a concise medical history overview encompassing diagnoses, notable medical events and previous treatments. We paired each patient with either a single or two slices of CT or MRI imaging that served as either sequential follow-up staging scans of the liver or lungs or simultaneous staging scans of both the liver and the lungs at a single point in time. Images were obtained predominantly from internal sources from the Department of Diagnostic and Interventional Radiology, University Hospital Aachen, but we included a few samples from the web and the Cancer Imaging Archive^52,53^. We highlight these cases in Supplementary Note 1. Histology images were obtained from TCGA. We additionally included information into genomic variations (mutations and gene fusions) in several patient descriptions. To evaluate our model’s proficiency in handling complex information, we decided to not pose a single straightforward question but instead structured each query with multiple subtasks, subquestions and instructions, necessitating the model to handle an average of three to four subtasks in each round. To evaluate the robustness of the model in using tools across various patient factors (Extended Data Fig. 1), we generated 15 random combinations of age, sex and origin for each of the 20 base cases, resulting in a total of 300 combinations.

### Human results evaluation

To enhance the assessment of free-text output, we developed a structured evaluation framework, drawing inspiration from the methodology of Singhal et al.^54^. Our evaluation focused on three primary aspects: the use of tools by the agent, the quality of the text output produced by the model and the adherence in providing accurate citations.

In reference to the former, we established a manual baseline for the expected use of tools necessary for generating additional patient information that is crucial for resolving the patient’s task. We measured this by the ratio of actual versus expected (required) tool uses. The requirement of tool use was defined as either the model is directly instructed to use a certain tool or the output of a tool is essential to proceed in answering the question, which is the default setting in almost all situations. Additionally, we assessed the helpfulness of the model, quantified by the proportion of user instructions and subquestions directly addressed and resolved by the model.

In assessing the textual outputs, our evaluation first encompassed factual correctness, defined by the proportion of correct replies relative to all model outputs. To segment answers into more manageable units, we split each reply according to statement (where a statement is considered a segment that concludes with either a reference to literature or is followed by a shift in topic in the subsequent sentence). Correspondingly, we distinguished between incorrectness and harmfulness in responses. Incorrect responses may include suggestions for superfluous diagnostic procedures or contain requests for irrelevant patient information. Conversely, harmful responses, while also incorrect, were determined by clinical judgment as potentially deleterious, such as advising suboptimal or contraindicated treatments. Furthermore, we assessed the completeness of the responses. For this purpose, we identified multiple specific keywords and terms for each unique medical scenario. These keywords represent expected interventions, such as treatments or diagnostic procedures and were carefully selected for their case relevance and crafted to be as specific as possible (for example, precise treatment combinations such as ‘FOLFOX and bevacizumab’ instead of ‘chemotherapy and antiangiogenic drugs’). This criterion, which we term ‘completeness’, was supposed to measure the extent to which the agent’s response aligns with the essential information that oncologists would anticipate in a human-generated answer under similar conditions.

Lastly, we evaluated the alignment of the responses with the original document segments used by the model through RAG. For each reference in the model’s output, we investigated the corresponding reference by its source identifier. Our evaluation encompassed three critical dimensions: citation correctness, ensuring the model’s statements faithfully mirror the content of the original document, irrelevance, identifying instances where the model’s assertions are not substantiated by the source material, and incorrect citation, detecting discrepancies where the information attributed to a source diverges from its actual content.

All evaluations described here were performed independently by four certified clinicians with expertise in oncology. For all benchmarks, we reported the majority vote. In cases of a tie, we selected the most adverse outcome, adhering to a hierarchical schema: correct, irrelevant and wrong for citations and correct, wrong and harmful for the correctness evaluation.

Data were analyzed using the pandas library and visualized with matplotlib in Python.

### Statistics and reproducibility

Data distribution was assumed to be normal but this was not formally tested. Sample sizes were set at *n* = 20. No statistical methods were used to predetermine sample sizes. Experiments were not randomized, as only one test group (the AI agent) existed in this proof-of-concept study. The investigators were not blinded to allocation during experiments and outcome assessment; however, they were blinded to the model responses while establishing the ground truth for the necessity of tool use and the completeness of the responses. Experiments were limited to *n* = 1 per case because of stringent rate and access limitations at the time of experimentation, when access to GPT-4V was available only as a preview. No data were excluded in the analysis. Reproducibility of the study’s findings may be affected by silent changes implemented to the model by its developers, which may not always be publicly disclosed, as well as by potential future model deprecations. Despite these factors, the results are expected to remain reproducible when using other current state-of-the-art models. Additionally, built-in guardrails designed to prevent potentially harmful content occasionally led to refusals and to inadequately addressing medical queries. In these instances, we reran each sample in newly instantiated settings until no such refusals occurred.

### Reporting summary

Further information on research design is available in the Nature Portfolio Reporting Summary linked to this article.

## Supplementary information

Supplementary Information Supplementary Notes 1–4.

Reporting Summary

Software Checklist

Supplementary Tables 1–7 Supplementary Tables 1–7.

## Source data

Source Data Figs. 2, 4 and 5 Model performance with and without tools.

Source Data Figs. 1 and 3 Image source data.

## Data Availability

All radiology images used in this study are available from the respective URLs as indicated in the Supplementary Information. The histopathology related results in our study are based upon data generated by TCGA Research Network (https://www.cancer.gov/tcga). Source data are provided with this paper.

## Code availability

An implementation of our source codes for researchers to extend on our work is available from GitHub (https://github.com/Dyke-F/LLM_RAG_Agent). Instructions for using the repository are detailed in the respective ReadMe file.
