# Supplementary material for: Development and validation of an autonomous artificial intelligence agent for clinical decision-making in oncology
Source: Nat Cancer. 2025 Jun 6;6(8):1337–49. doi: 10.1038/s43018-025-00991-6 (PMC12380607; doi:10.1038/s43018-025-00991-6)
Supplement: Supplementary file 1 — Supplementary Notes 1–4. [file 43018_2025_991_MOESM1_ESM.pdf]

nature cancer

Supplementary information

https://doi.org/10.1038/s43018-025-00991-6

Development and validation of an autonomous artificial intelligence agent for clinical decision-making in oncology

In the format provided by the authors and unedited

Supplementary Note 1

No.	Patient Case Description
A	Mrs. Adams (DOB: 03/05/1969, ID: A-CC-38), a 55-year-old female, has been battling left-sided colorectal cancer for an extended period. Four years after her initial diagnosis, in November 2023, a relapse was detected, characterized by multiple metastases to the liver, as evidenced by the imaging study dated 11/11/2023 (CTA_11_11_2023.png). Subsequently, she commenced a chemotherapy regimen comprising FOLFOX and bevacizumab at an external hospital in Berlin. The latest imaging, captured on 02/24/2024 (CTA_02_24_2024.png), is stored in the patient's folder within the Images path of the system. To date, there are no additional metastatic sites identified beyond the liver. Mrs. Adams has generally tolerated the chemotherapy well. However, she has recently reported an increase in blood pressure, with readings consistently in the range of 170-190 mmHg. Apart from elevated blood pressure, she has not experienced any other symptomatic manifestations of her condition.

Doctor: How did the disease develop? What are the next steps in her cancer treatment plan?

Appendix[1]:

CT-A, 11/11/2023

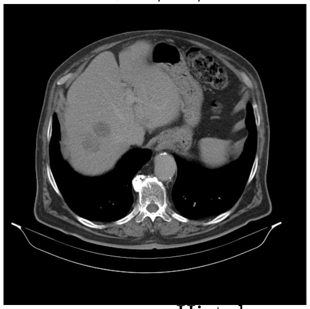

CT-A, 02/24/2024

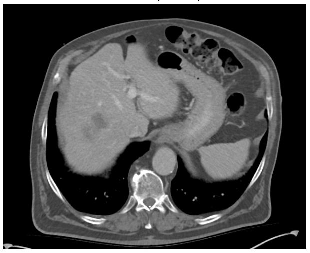

Histology

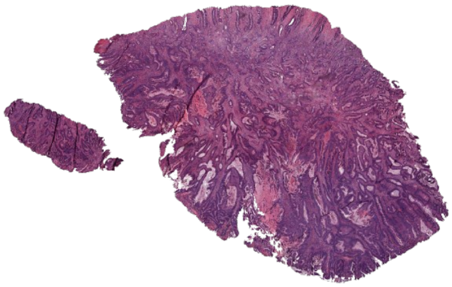

Model Response[2]:
Given Mrs. Adams' stable liver metastases as evidenced by the comparison of CTA_11_11_2023.png and CTA_02_24_2024.png [Tool], alongside her genetic profile indicating MSS, and wild-type status for both BRAF and KRAS [Tool], her disease appears to be in a controlled state under the current regimen of FOLFOX and bevacizumab. The stability of liver lesions suggests that the chemotherapy, in conjunction with bevacizumab, is effective in managing her metastatic colorectal cancer (mCRC) [Tool].

No.	Patient Case Description
	The development of hypertension, a known side effect of bevacizumab [11][12][13], necessitates a multidisciplinary approach to manage this condition without compromising her cancer treatment efficacy. Given the stable disease and the absence of BRAF and KRAS mutations [Tool], Mrs. Adams remains a candidate for continued anti-EGFR therapy, considering her left-sided mCRC, which is typically more responsive to such treatments [2][8].<br><br>**Next Steps in Treatment Plan:**<br>**1. Management of Hypertension:** Immediate consultation with a cardiologist is recommended to manage her newly developed hypertension, potentially related to bevacizumab [22][23][26][27]. Adjustments to her antihypertensive therapy may be necessary, with continuous monitoring of blood pressure to prevent cardiovascular complications [68][69][70][71].<br>**2. Ongoing Cancer Treatment:** Given the stable disease, continuation of the current regimen is advisable. However, the patient's tolerance, especially concerning the development of hypertension, should be closely monitored [1][4]. If hypertension cannot be controlled, a dose adjustment or a switch to an alternative antiangiogenic agent with a potentially different side effect profile, such as aflibercept or ramucirumab, could be considered [9][86].<br>**3. Re-evaluation for Surgical Intervention:** Despite the stable metastatic disease, re-evaluation for the possibility of liver metastasectomy or ablation should be considered, as these could offer a chance for prolonged survival or even cure in selected cases [33][34][35].<br>**4. Maintenance Therapy:** If surgery is not feasible and once a maximum response to the current regimen is achieved, transitioning to maintenance therapy with a fluoropyrimidine (e.g., capecitabine) with or without bevacizumab could be considered to reduce toxicity while maintaining disease control [4][85].<br>**5. Genetic and Molecular Marker Re-evaluation:** Although initial genetic predictions indicate MSS, and wild-type BRAF and KRAS, a broader panel re-evaluation might uncover other actionable mutations or markers that could open up additional targeted therapy options [33][42][46].<br>**6. Clinical Trials and Novel Therapies:** Exploration of clinical trials focusing on novel therapies or combinations that could be beneficial for her specific genetic and molecular profile is recommended [6][33].<br>**7. Palliative Care Integration:** Early integration of palliative care to manage symptoms, improve quality of life, and support Mrs. Adams and her family through treatment decisions is crucial [126][127][129].<br>**8. Regular Monitoring:** Continuous assessment of her disease with imaging studies and tumor markers is essential to evaluate the response to treatment and adjust the treatment plan as necessary [38][80].<br>This comprehensive approach, tailored to Mrs. Adams' specific medical history, current condition, and potential treatment pathways, aims to optimize her quality of life while effectively managing her mCRC. Regular reassessment of her condition and treatment tolerance will guide any necessary adjustments to her treatment plan.<br>To further refine Mrs. Adams' treatment plan, it would be helpful to know the specifics of her blood pressure readings over time, to assess the severity and progression of her hypertension. Utilizing my array of resources, I can further assist by identifying optimal blood pressure management strategies that align with her ongoing cancer treatment. Additionally, detailed information regarding her response to previous treatments and any side effects experienced would be invaluable. This would enable a more personalized approach to considering the integration of anti-EGFR therapy, ensuring it complements her current regimen while minimizing potential adverse effects.

No.	Patient Case Description

B Mrs. Brown, identified in our system as X11-01A, is a 49-year-old female patient who has been navigating her journey with metastatic colorectal cancer since 2019. Her treatment history includes chemotherapy followed by surgical interventions, specifically a total mesorectal excision and a right-sided hemi-hepatectomy. In the previous year, a follow-up CT scan revealed the presence of a single metastasis in the liver, prompting the initiation of a chemotherapy regimen that combined FOLFOX with Panitumumab. Currently, Mrs. Brown returns with a new CT scan dated February 10, 2024, ordered by her oncologist. Additionally, she provides microscopic images from a recent biopsy of the tumor. All relevant imaging data have been uploaded to our system.

Doctor: How do we best proceed with the oncologic treatment of the patient? Is a continuation of chemotherapy plus Panitumumab recommended? What other options do we have?

Appendix:

CT-A, 11/01/2023

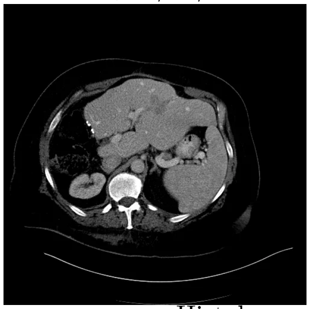

CT-A, 02/10/2024

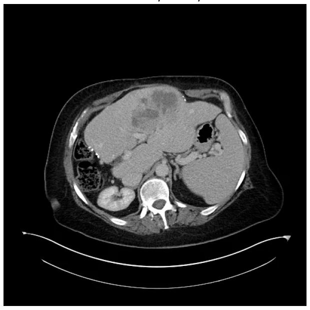

Histology

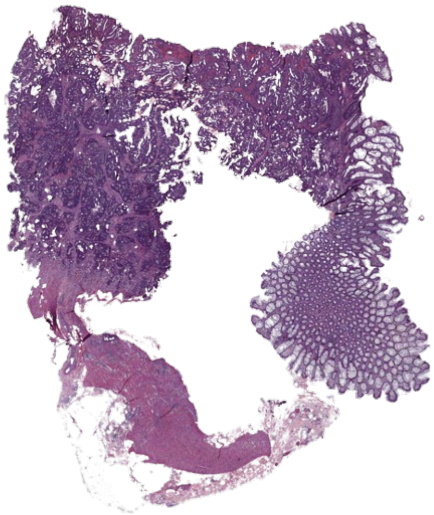

Model Response:
Given Mrs. Brown's current clinical scenario, including the recent CT findings and genetic profile of her tumor, a strategic re-evaluation of her treatment plan is warranted. The presence of a new liver lesion suggests disease progression, and the genetic analysis revealing a KRAS mutation significantly impacts the choice of targeted therapy. Specifically, the efficacy of Panitumumab, an EGFR inhibitor, is compromised in the context of KRAS mutations [25], [26]. Therefore, continuing chemotherapy plus Panitumumab is not recommended based on the current evidence.

No.	Patient Case Description
	**1. Discontinue Panitumumab:** Given the KRAS mutation, discontinuing Panitumumab is advised. KRAS mutations are known to confer resistance to EGFR inhibitors, rendering treatments like Panitumumab ineffective [25], [26].<br>**2. Adjust Chemotherapy Regimen:** Considering the disease progression, adjusting the chemotherapy regimen might be necessary. Options include switching to a different chemotherapy backbone such as FOLFIRI (folinic acid, fluorouracil, and irinotecan) if not previously used, or exploring the addition of bevacizumab, an anti-VEGF therapy, which is not affected by KRAS mutation status and could offer a benefit in this setting [10], [11], [12].<br>**3. Explore Additional Targeted Therapies:** Given the KRAS mutation, targeted therapies that are effective against KRAS-mutated tumors should be considered. While direct KRAS inhibitors like sotorasib are currently approved for lung cancer with KRAS G12C mutations, ongoing trials are exploring their efficacy in colorectal cancer. They may offer potential benefits, although their applicability depends on the specific KRAS mutation present. Participation in a clinical trial targeting KRAS mutations could be an option [39], [40].<br>**4. Consider Local Treatments for Liver Metastasis:** Depending on the size, location, and number of liver metastases, local treatments such as radiofrequency ablation (RFA) or stereotactic body radiation therapy (SBRT) could be considered to manage the liver disease [6], [16].<br>**5. Clinical Trials:** Enrolling Mrs. Brown in clinical trials focusing on novel therapies for KRAS-mutated mCRC or new combinations of existing drugs could provide access to cutting-edge treatments and potentially improve outcomes [4].<br>**6. Supportive Care and Monitoring:** Regardless of the treatment adjustments, ensuring supportive care to manage symptoms and side effects, along with regular monitoring of disease status through imaging and biomarkers, is crucial [6].<br>**7. Patient Education and Involvement:** Discussing the rationale behind changing the treatment plan, potential benefits and side effects of the new regimen, and the importance of close monitoring with Mrs. Brown is essential. Her preferences and concerns should be taken into account in shared decision-making [7].<br>In summary, the treatment plan for Mrs. Brown should involve discontinuing Panitumumab due to the KRAS mutation, considering an alternative chemotherapy regimen possibly combined with bevacizumab, exploring eligibility for clinical trials, and evaluating local treatment options for liver metastasis. Regular follow-up and supportive care remain integral components of her care plan.<br>To refine our approach based on Mrs. Brown's unique genetic profile and the progression of her disease, it would be beneficial to gather more detailed information on any additional genetic alterations that have not yet been explored. Utilizing my array of resources, I can further assist by identifying the most promising treatment avenues tailored to her specific genetic landscape. This includes pinpointing novel or underutilized targeted therapies that may bypass the resistance mechanisms posed by the KRAS mutation. Additionally, understanding the full scope of her disease through comprehensive genetic profiling could unveil new opportunities for clinical trial participation, potentially offering access to innovative treatments not yet available in standard care.

No.	Patient Case Description
D	Mr. Doe, a 53-year-old male patient with the identification X22-70C, has presented to the oncology department with a recent diagnosis of colorectal cancer. He has provided CT images, which have been stored in the 'Imaging' directory under a subfolder bearing his family name. Unfortunately, a corresponding radiology report is not available at this time. Additionally, Mr. Doe mentions a biopsy conducted last week, the slides of which have been uploaded by the pathology department into the 'Histology' folder. His medical history and physical examination have not revealed any cancer-specific findings. During his consultation, Mr. Doe repeatedly expresses his refusal to undergo chemotherapy. He also mentions undergoing molecular testing at another facility, which, to the best of his recollection, identified a BRAF V600E mutation.

Doctor: Is the patient rather suitable for surgery or systemic therapy? Are there options for targeted therapies? Check available drugs for the known mutation in databases and online. Are there other mutations?

Appendix:

CT Liver

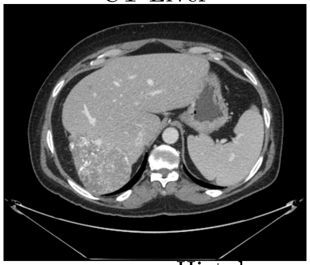

CT Lung

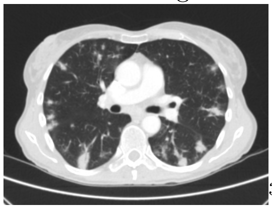

3

Histology

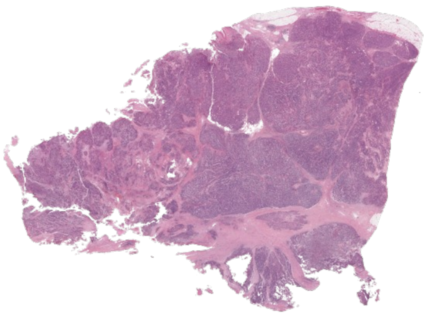

Model Response:
Given Mr. Doe's advanced metastatic colorectal cancer with evidence of metastases in both the lungs and liver, as indicated by the **gen_radiology_report** tool analysis of **CT_Lung.png** and **CT_Liver.png** [Tool], he is more suitable for systemic therapy rather than surgery. The presence of multiple well-defined nodules in both lung fields and multiple hypodense lesions in both lobes of the liver strongly suggests a widespread metastatic disease, making surgical intervention less feasible and unlikely to provide curative outcomes [Tool].
The **check_mutations** tool's prediction of MSI-High status with a probability of 0.95 [Tool] opens up possibilities for immunotherapy, particularly given Mr. Doe's refusal to undergo chemotherapy. Pembrolizumab, an immune checkpoint inhibitor, is recommended for patients with MSI-High or dMMR metastatic colorectal cancer (mCRC) as a first-line treatment [25][30]. This aligns with Mr. Doe's treatment preferences and the current medical guidelines suggesting pembrolizumab for MSI-High mCRC [81][84].

No.	Patient Case Description
	The discrepancy regarding the BRAF V600E mutation status, with Mr. Doe's recollection of a positive result and the **check_mutations** tool predicting wild-type BRAF with a probability of 0.48 [Tool], necessitates a confirmatory molecular test. If the BRAF V600E mutation is confirmed, targeted therapy with a combination of encorafenib and cetuximab is recommended in the second line, based on the BEACON study results and FDA approval for mCRC with BRAF V600E mutation after prior therapy [45][49]. This combination has shown efficacy in increasing overall survival compared with standard chemotherapy [72][73].<br>Furthermore, the slight likelihood of a KRAS mutation, as indicated by the **check_mutations** tool with a probability of 0.52 [Tool], suggests that anti-EGFR therapies such as cetuximab or panitumumab, typically used in RAS wild-type mCRC, may not be as effective [42][43]. This further emphasizes the importance of confirming the BRAF mutation status, as the presence of a KRAS mutation limits the use of certain targeted therapies.<br>Given the advanced nature of Mr. Doe's disease and his preference against chemotherapy, exploring clinical trials and newer treatment modalities is also a prudent step. This could include trials focusing on novel immunotherapies, targeted therapies based on other actionable mutations, or combination therapies that have not yet been standardly approved but show promise in early-phase trials.<br>In summary, Mr. Doe's treatment strategy should involve:<br>1. Confirming the BRAF mutation status through a reliable molecular testing method. If BRAF V600E mutation is confirmed, proceed with targeted therapy using encorafenib plus cetuximab in future lines of treatment [45][49].<br>2. Considering immunotherapy with pembrolizumab due to the MSI-High status, which has shown efficacy in MSI-High or dMMR mCRC as a first-line treatment [25][30].<br>3. Exploring clinical trials and newer treatment modalities, given the advanced nature of Mr. Doe's disease and his preference against chemotherapy.<br>4. Involving a multidisciplinary team including medical oncologists, genetic counselors, and possibly palliative care specialists to address Mr. Doe's treatment preferences, manage symptoms, and ensure the best possible quality of life.<br>Further investigations should include a repeat or confirmatory genetic test to resolve the discrepancy regarding the BRAF mutation status and comprehensive molecular profiling to identify any other actionable mutations or biomarkers that could guide therapy selection. This approach ensures a comprehensive and patient-centered care plan that aligns with the latest clinical evidence and Mr. Doe's treatment preferences.<br>To further refine our analysis and recommendations, could you please provide the coordinates of the nodules and lesions highlighted in the CT scans? This information is crucial for a detailed segmentation analysis, which will help in accurately assessing the extent of metastatic spread. Additionally, if you could supply the measurements of these lesions over time, we can perform a progression analysis to better understand the disease's trajectory. Lastly, sharing specific search terms or questions you have regarding colorectal cancer, particularly those focusing on KRAS mutations and targeted therapy options, would enable a targeted literature review, ensuring we gather the most relevant and up-to-date information to guide treatment planning.
G	Mr. Garcia, a 61-year-old male, has been under treatment for metastatic colorectal cancer. He previously underwent six cycles of FOLFOX therapy at an external hospital. Mr. Garcia has provided a CT scan of the abdomen from November 8, 2023, taken before the initiation of his treatment, now uploaded under the filename '08_11_23.png.' We also have an corresponding old radiology report:<br>****<br>**Radiology Report from November, 8 2023:**<br>Abdominal MRI-scan.

No.	Patient Case Description
	Clinical Information: Biospy-confirmed colorctal cancer with potential liver metastases according to ultra-sound.<br>Technique: Multi-phase imaging of the abdomen with intravenous contrast.<br>Findings:<br>Primary Tumor: The primary colorectal tumor is identified within the upper 1/3 of the rectum without evidence of intestinal occlusion<br>Liver: There are multiple hepatic lesions consistent with metastases. We highlight three target lesions: The largest lesion is located in position 190, 195, 225, 225. Two more lesions cand be found at [230, 100, 260, 130] and [140, 245, 170, 270]. No evidence of biliary dilation.<br>Kidneys, Adrenals, Pancreas, Spleen: No abnormalities detected. Organs appear normal in size, shape, and attenuation without evidence of lesions or masses.<br>Lymph Nodes: No enlarged abdominal or pelvic lymph nodes are identified.<br>Vasculature: The abdominal aorta and its major branches are patent without evidence of aneurysm or significant stenosis.<br>Bowel and Mesentery: No evidence of bowel obstruction, focal bowel wall thickening, or mesenteric masses.<br>Peritoneum: No ascites or peritoneal nodules are evident.<br>Other Findings: There are no other significant abnormalities detected in the abdomen.<br>****<br>A recent control imaging conducted by our in-house radiologists is saved as '02_10_24.png'. All related imaging data are accessible on the server under 'Imaging/family_name.' The preliminary report states "... multiple hepatic lesions consistent with metastases. The largest lesion is located in position: [120, 260, 165, 305], more are found in positions [240, 220, 280, 255], [195, 185, 235, 220] and 145, 235, 180, 275. No comparison to previous imaging available."

Doctor: How do we best proceed with the treatment of this patient?

Appendix:

MRI-A, 11/01/2023

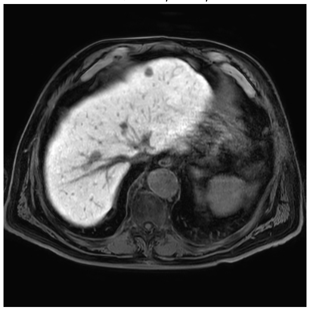

MRI-A, 02/10/2024

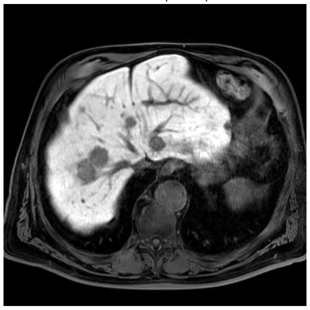

Model Response:

Given Mr. Garcia's progression of metastatic colorectal cancer, as evidenced by the significant increase in the overall area of hepatic lesions from 1,315 square units to 2,809 square units [Tool], which indicates a growth by a factor of 2.14 [Tool], a comprehensive and multidisciplinary approach is warranted. The strategy for Mr. Garcia should include the following steps, aligned with the structured approach and medical guidelines:

No.	Patient Case Description
	**1. Re-evaluation of the Molecular Profile:** Given the progression of disease, it's crucial to reassess the molecular characteristics of Mr. Garcia's tumor. This includes testing for RAS, BRAF mutations, and MSI status, as these can significantly influence the choice of targeted therapies [1], [2]. If not previously done, testing for HER2 amplification and NTRK fusions should also be considered due to their potential actionability [10], [124].<br>**2. Assessment for Second-Line Chemotherapy Options:** Based on the molecular profile and prior treatment with FOLFOX, if Mr. Garcia's tumor is RAS wild-type and without BRAF V600E mutation, options may include anti-EGFR therapy (cetuximab or panitumumab) combined with irinotecan-based chemotherapy [2], [11]. For RAS or BRAF mutant tumors, or if anti-EGFR therapy is not suitable, consideration of FOLFIRI with bevacizumab or aflibercept could be appropriate [2], [7].<br>**3. Consideration of Immunotherapy:** For tumors that are MSI-H or dMMR, immunotherapy with pembrolizumab or nivolumab, with or without ipilimumab, should be considered [3], [16], [26], [27]. This is based on the significant efficacy of immune checkpoint inhibitors in this subset of patients.<br>**4. Liver-Directed Therapies:** Given the liver-dominant nature of Mr. Garcia's metastatic disease, exploring liver-directed therapies such as radioembolization (Y-90), transarterial chemoembolization (TACE), or ablative therapies could provide local control and potentially palliate symptoms [38], [40], [48], [49]. These options should be discussed in a multidisciplinary team (MDT) setting, considering Mr. Garcia's overall health status and liver function.<br>**5. MDT Discussion and Supportive Care Optimization:** An MDT discussion involving medical oncology, surgical oncology, radiology, and palliative care is essential to integrate the above considerations into a personalized treatment plan for Mr. Garcia [Strategy]. Concurrently, optimization of supportive care measures to manage symptoms and maintain quality of life is paramount [121].<br>**6. Clinical Trial Enrollment:** Finally, given the refractory nature of Mr. Garcia's disease, exploration of eligibility for clinical trials testing novel agents or combinations should be a priority. This could offer access to emerging therapies and contribute to the advancement of treatment for metastatic colorectal cancer [136].<br>In summary, Mr. Garcia's treatment plan should be personalized based on the latest molecular profiling, consider second-line systemic therapy options tailored to his tumor's characteristics, evaluate the feasibility of liver-directed therapies, and ensure supportive care needs are met, all within the context of an MDT discussion. Participation in clinical trials should also be actively pursued.<br>To further refine our approach in managing Mr. Garcia's condition, I would need to analyze histologic images for genetic mutations that could guide targeted therapy selection. Could you provide the path to the folder containing Mr. Garcia's histologic images? This will enable me to perform genetic modeling to check for MSI, BRAF, and KRAS mutations. Additionally, knowing any specific genetic alterations identified would allow me to consult relevant databases for targeted treatment options, enhancing the precision of Mr. Garcia's treatment plan. Lastly, detailed information regarding Mr. Garcia's eligibility criteria for clinical trials would allow for a targeted search for novel therapeutic opportunities, potentially offering access to groundbreaking treatments.

No.	Patient Case Description
Le	Mr. Lee, a 72-year-old individual, was initially diagnosed with pancreatic cancer in December 2022. Following his diagnosis, he underwent a pancreatoduodenectomy (Whipple procedure), and completed five cycles of gemcitabine chemotherapy. Mr. Lee chose to conclude the chemotherapy sessions based on personal preference, although he was tolerating the treatment well. He has been under regular observation since then. In November of the previous year, a CT scan identified a small lung nodule, raising concerns about a potential recurrence of the cancer (The CT slides are in the 'Imaging' directory under his family subdirectory for evaluation). After consulting with Mr. Lee, a decision was made to adopt a watchful waiting approach. Recently, another CT scan was conducted and the results have been uploaded to the server. Despite these medical concerns, Mr. Lee reports feeling well and maintains an active lifestyle, engaging in sports activities twice weekly. He currently exhibits no clinical symptoms indicative of cancer progression. Molecular analysis of the tumor has revealed the presence of a KRAS G12C mutation.

Doctor: Please provide an overview of the standard chemotherapy protocols recommended for situations similar to Mr. Lee's, in alignment with current medical guidelines. Additionally, investigate and summarize the most recent online information regarding treatment options for KRAS G12C mutations, specifically for Mr. Lee's case.

Appendix:

CT Lung, November 2023

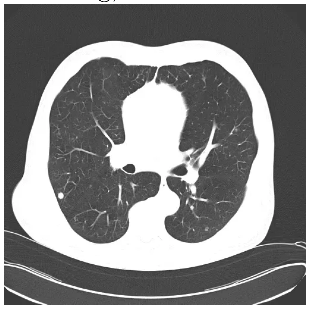

CT Lung, February 2024

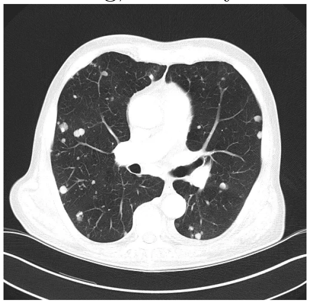

Model Response:

Given Mr. Lee's history of pancreatic cancer, post-Whipple procedure status, and completion of five cycles of gemcitabine chemotherapy, along with the recent CT scan findings indicating progression of lung nodules, a comprehensive approach is warranted. The presence of a KRAS G12C mutation in his tumor adds a significant layer to his treatment planning, as this mutation opens the door to targeted therapy options that have shown promise in recent studies [Tool].

Standard Chemotherapy Protocols

For patients like Mr. Lee, who have undergone surgical resection followed by adjuvant chemotherapy but show signs of disease recurrence or progression, the choice of further treatment depends on several factors including performance status, extent of disease, and molecular tumor profile. In the absence of specific contraindications, and considering Mr. Lee's good performance status and active lifestyle, a more aggressive approach could be considered.

No.	Patient Case Description
	**1. Modified FOLFIRINOX:** This regimen, consisting of oxaliplatin, irinotecan, fluorouracil (5-FU), and leucovorin, has been recommended for patients with a good performance status in the metastatic setting and could be considered here, despite its intensive nature [4][5]. The regimen's efficacy in the adjuvant setting post-surgery suggests its potential utility in cases of recurrence [13][20].<br>**2. Gemcitabine and Nab-Paclitaxel:** This combination has shown efficacy in the metastatic pancreatic cancer setting and could be an option for Mr. Lee, given his previous tolerance of gemcitabine [16][18]. This regimen might offer a balance between efficacy and tolerability.<br><br>**Targeted Therapy for KRAS G12C Mutation**<br>The discovery of the KRAS G12C mutation in Mr. Lee's tumor is pivotal. Recent advancements have led to the development of targeted therapies for this specific mutation, which is traditionally considered challenging to treat [Tool].<br>**1. Sotorasib:** As the first targeted agent with regulatory approval for KRAS G12C-mutated NSCLC, sotorasib represents a novel treatment option. Although primarily studied in lung cancer, its efficacy in KRAS G12C-positive tumors suggests potential applicability in Mr. Lee's case, given the lung nodules' progression [26][29][33]. The reported durable clinical responses and manageable safety profile make it a compelling option [Tool].<br>**2. Divarasib (GDC-6036):** This agent has shown durable responses across KRAS G12C-positive tumors, including low-grade adverse events, which could be advantageous for maintaining Mr. Lee's quality of life [Tool].<br>**3. Adagrasib:** Similar to sotorasib, adagrasib has shown efficacy in KRAS G12C-mutated tumors. Its use, alone or in combination with cetuximab, has been evaluated in colorectal cancer patients with this mutation, showing promising efficacy [Tool]. Given the cross-tumor activity of KRAS G12C inhibitors, adagrasib could be considered for Mr. Lee, especially in light of the lung nodules' progression.<br><br>**Conclusion and Next Steps**<br>For Mr. Lee, a dual approach incorporating systemic chemotherapy with consideration for targeted therapy against the KRAS G12C mutation appears warranted. Given the progression in lung nodules and the presence of the KRAS G12C mutation, exploring the use of sotorasib or adagrasib, in consultation with a multidisciplinary team, could provide a targeted approach to his treatment. Additionally, enrollment in clinical trials investigating these or new agents for KRAS G12C mutations should be considered, offering Mr. Lee access to cutting-edge treatments and contributing to the growing body of knowledge on managing this genetic alteration [Tool].<br>Regular monitoring and supportive care measures are essential to maintain Mr. Lee's quality of life during treatment, including managing any side effects from the targeted therapy and providing psychological support as needed. This approach aligns with current medical guidelines and reflects a personalized treatment strategy based on Mr. Lee's unique medical history, current condition, and molecular tumor profile [4][5][13][20][26][29][33].<br>To further refine our approach and ensure the most accurate and personalized treatment recommendations for Mr. Lee, I would need to delve deeper into specific aspects of his condition. If you could provide more details on the lung nodules, this would help in assessing the dynamics of the disease more accurately. Additionally, if histopathology images are available, providing the unique identifier for Mr. Lee along with specifying interest in the KRAS mutation would enable a precise genetic modeling to confirm the KRAS G12C mutation status. This detailed information is crucial for tailoring the most effective treatment strategy.

No.	Patient Case Description
Lo	Mr. Lopez, aged 54 (DOB: 03/01/1970, Patient ID: AA-21-C), visited our oncology department for a consultation following his recent diagnosis of left-sided colorectal cancer, which was discovered during a routine colonoscopy where a biopsy was taken. He had an appointment at the radiology department earlier today for a CT scan; the images have been uploaded for our review, although we are currently awaiting the radiologist's report. Mr. Lopez reports feeling generally well, with an ECOG performance status of 1, and he exhibits no specific symptoms related to the tumor. His medical history includes hypertension and a past humerus fracture in 2013. He is currently not on any medication.

Doctor: What is the optimal management approach for this patient? Develop a detailed treatment plan. Should we consider local treatment options such as surgical intervention or liver-directed therapies?

Appendix:

CT Abdomen

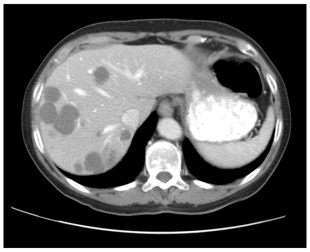

Histology

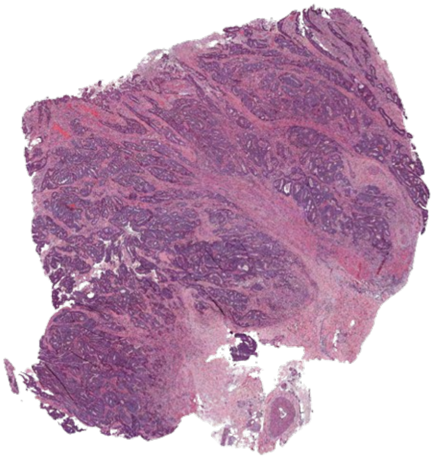

Model Response:
Given Mr. Lopez's diagnosis of left-sided colorectal cancer with potential liver metastases, MSS status, and wild-type BRAF and KRAS genes [Patient], [Tool], the optimal management approach should be multi-pronged, incorporating systemic chemotherapy, evaluation for surgical resection or liver-directed therapies, and supportive care measures.
1. Systemic Chemotherapy Initiation: Based on the NCCN guidelines [1], for Mr. Lopez, who has MSS, left-sided colorectal cancer without BRAF or KRAS mutations, a combination chemotherapy regimen such as FOLFOX (oxaliplatin plus fluorouracil and leucovorin) or FOLFIRI (irinotecan plus fluorouracil and leucovorin) should be considered as the first line of treatment. Given the absence of RAS mutations, the addition of an anti-EGFR agent, specifically cetuximab or panitumumab, is recommended to enhance treatment efficacy [1], [74].

No.	Patient Case Description
	**2. Further Imaging and Assessment for Liver Metastases:** Before deciding on local treatments, further characterization of the liver lesions with MRI or PET-CT is advised to confirm metastasis and assess resectability [7]. This step is crucial as it influences the decision regarding local versus systemic therapy and the potential for curative intervention [4].<br>**3. Evaluation for Surgical Resection or Liver-Directed Therapies:** If the liver metastases are confirmed and deemed limited and potentially resectable, Mr. Lopez should be evaluated by a multidisciplinary team including a surgical oncologist [4], [6]. Options such as radiofrequency ablation (RFA), microwave ablation (MWA), or stereotactic body radiotherapy (SBRT) could be considered if surgical resection is not feasible due to the location or number of lesions, or if Mr. Lopez's health status does not permit surgery [21], [23], [84].<br>**4. Maintenance Therapy and Regular Monitoring:** Following the initial treatment phase, transitioning Mr. Lopez to maintenance therapy with a less intensive regimen, such as fluoropyrimidine alone or in combination with bevacizumab, could be considered to manage the disease while minimizing side effects [32]. Regular monitoring through imaging studies and tumor markers will be crucial to adjust the treatment plan based on the disease's response and progression.<br>**5. Comprehensive Supportive Care:** Throughout the treatment process, it is essential to provide supportive care measures aimed at managing symptoms, improving quality of life, and addressing any treatment-related side effects. This includes nutritional support, pain management, psychological support, and management of chemotherapy or targeted therapy-related toxicities [5].<br>In summary, Mr. Lopez's treatment plan should start with systemic chemotherapy combined with an anti-EGFR agent, followed by a detailed assessment of liver metastases for potential resectability or suitability for liver-directed therapies. The treatment strategy should be flexible, with adjustments based on response to therapy and Mr. Lopez's overall health status. A multidisciplinary approach involving oncologists, radiologists, surgical oncologists, and supportive care specialists is essential for optimal management.<br>To further refine Mr. Lopez's treatment strategy with precision and up-to-date research findings, could you provide the exact genetic mutations beyond BRAF and KRAS wild-type status? This will enable a detailed OncoKB analysis to identify any additional targeted therapy options. Additionally, for a comprehensive assessment of the liver metastases, please supply the file path to the most recent CT scan images along with the coordinates for each visible lesion. This will assist in accurately determining the extent of liver involvement and the feasibility of local treatments.
S	Mrs. Smith, a 57-year-old patient with the System ID B13-22A, is under the care of our oncology department due to her diagnosis of metastatic colorectal cancer in September 2023. Today marks her initial visit to our department, and she has provided a brief summary of her medical journey thus far:<br><br>****<br>To our esteemed colleague,<br><br>This correspondence pertains to Mrs. Smith, born on March 31, 1977, who is receiving oncological treatment in our facility.<br><br>**Primary Diagnosis:**<br>Colorectal cancer, staged as T3, N2, M+ (HEP), identified in May 2023.<br>Genetic profiling of the liver biopsy from May 2023 confirmed KRAS wild type, BRAF wild type, and MSS status.<br>**Secondary Diagnoses:**

No.	Patient Case Description

Hypothyreosis
arterial Hypertension
Chemotherapy-induced polyneuropathy

Timeline:

05/2023: Diagnosis established for colorectal cancer with hepatic metastasis, substantiated by liver biopsy revealing KRAS wt, BRAF wt, MSS.
05-08/2023: Administered 6 cycles of FOLFOX + Panitumumab
08/2023: Imaging studies of the thorax and abdomen indicated a partial remission in the known hepatic metastases without the emergence of new metastases. The primary tumor remained stable.
08/2023-11/2023: Continued treatment with six cycles of FOLFOX and Panitumumab, with a reduction in Oxaliplatin dosage to 80% from the third cycle onwards (totaling nine cycles) due to the onset of polyneuropathy.
11/23 - 02 / 2024: 6 cycles 5-fluorouracil with Panitumumab
****
Mrs. Smith has also brought along an external CT scan[4]of the chest and abdomen dated February 2024 for our evaluation. Additionally, a recent tumor biopsy was conducted, though the reports are yet to be furnished.

Doctor: What is the current state of the patient's health, particularly with respect to the liver metastases previously identified? Are these metastases showing signs of stability? Based on the current condition, is it advisable to persist with the regimen of 5-Fluorouracil and Panitumumab?

Appendix:

MRI-A, 08/11/2023

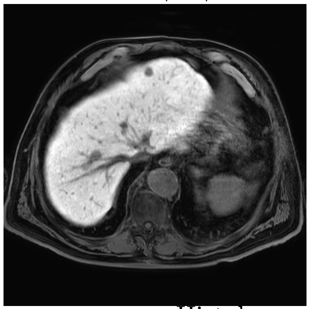

MRI-A, 02/10/2024

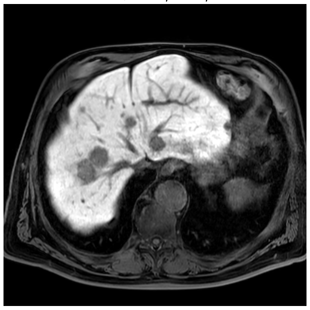

Histology

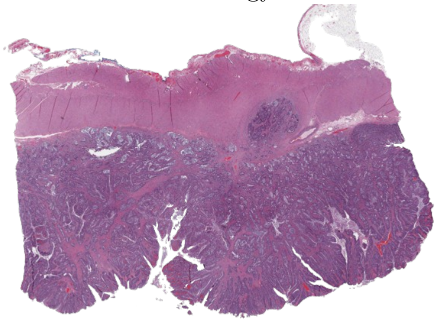

Model Response:

Given the comprehensive details provided about Mrs. Smith's medical history and the recent diagnostic findings, a thorough analysis reveals critical insights into her current health status, particularly concerning her liver metastases and the implications of the newly detected KRAS mutation on her treatment regimen.

Current Health Status and Liver Metastases:

No.	Patient Case Description
	The radiology report analysis comparing MRI scans from February 2024 and November 2023 indicates a notable change in the liver metastases. The increased brightness in the February 2024 scan compared to the November 2023 scan suggests a potential progression or alteration in the liver metastases [Tool]. However, without additional clinical context, such as liver function tests or symptoms, the clinical significance of this radiological change remains uncertain. It's crucial to correlate these imaging findings with clinical assessments and laboratory results including tumor markers to accurately determine the current state of the liver metastases.<br><br>**Implications of KRAS Mutation:**<br>The detection of a KRAS mutation (with a probability of 0.52) represents a significant change from the initial genetic profiling, which identified Mrs. Smith as KRAS wild type [Tool]. This finding is crucial as it impacts the efficacy of the current treatment regimen. Anti-EGFR therapies, such as Panitumumab, are known to be ineffective in the presence of KRAS mutations [117][118]. The presence of a KRAS mutation suggests that Mrs. Smith's cancer has acquired resistance to the anti-EGFR monoclonal antibody, thereby reducing the effectiveness of her current treatment regimen of 5-Fluorouracil and Panitumumab.<br>**Treatment Recommendations:**<br>Given the new genetic mutation analysis revealing a KRAS mutation, continuing the regimen of 5-Fluorouracil and Panitumumab is not advisable [117][118]. The detection of a KRAS mutation necessitates a reevaluation of Mrs. Smith's treatment plan. Alternative treatment options should be considered, focusing on regimens that do not rely on the efficacy of anti-EGFR therapies. Options may include chemotherapy combinations without anti-EGFR monoclonal antibodies or considering enrollment in clinical trials exploring novel agents targeting KRAS mutations or other pathways relevant to her tumor's genetic profile [120][126].<br><br>**Further Actions:**<br>**1. Clinical Correlation:** A multidisciplinary team discussion is recommended to integrate the new radiological and genetic findings with Mrs. Smith's clinical status, including any new symptoms or changes in her overall health.<br>**2. Additional Testing:** Further genetic testing to confirm the KRAS mutation subtype could provide insights into targeted therapy options, including KRAS G12C inhibitors if applicable [120].<br>**3. Treatment Adjustment:** Based on the KRAS mutation, switching to a chemotherapy regimen not reliant on EGFR inhibition, such as FOLFIRI (irinotecan, fluorouracil, and leucovorin), possibly in combination with bevacizumab if not contraindicated, should be considered [19][20].<br>**4. Clinical Trials:** Exploration of clinical trials targeting KRAS mutations or other novel therapies suitable for Mrs. Smith's genetic profile and disease state is highly recommended [126].<br>In conclusion, the new findings necessitate a significant adjustment in Mrs. Smith's treatment strategy, moving away from anti-EGFR therapies due to the detected KRAS mutation and considering alternative systemic therapies or clinical trial enrollment for novel treatments.

No.	Patient Case Description
	To enhance our understanding of the progression of Mrs. Smith's liver metastases and refine her treatment options, could you please supply the exact dates of the CT scans you're referring to? This will allow for a precise comparison of changes over time. Additionally, knowing the precise KRAS mutation subtype (e.g., G12C, G12D) will enable a more targeted search for applicable therapies and clinical trials. Lastly, for a comprehensive literature review on the efficacy of her current treatment in the context of her specific KRAS mutation, please provide any keywords or phrases that you believe are crucial for this search, such as "KRAS mutation colorectal cancer resistance." These details are vital for leveraging our resources to their fullest potential, ensuring a tailored approach to Mrs. Smith's care.
T	Mr Taylor, a 71-year-old male with a significant medical history of Hepatitis C Virus-induced liver cirrhosis, initially presented with small esophageal varices and has been under maintenance treatment with omeprazole and amlodipine for arterial hypertension. In July 2018, he was diagnosed with multifocal hepatocellular carcinoma (HCC), for which he received four sessions of Transarterial Chemoembolization (TACE). Despite these interventions, his HCC advanced, complicated by portal vein thrombosis as of February 2023. His liver function, as per the Child-Pugh classification, remains at A5, his ECOG PS is 0. AFP 90.4; CEA 19.9; Ca19-9 16.7.<br><br>**Doctor:** The progression of Mr. Taylors's HCC in the context of PVT requires a reassessment of therapeutic strategies to address the current complexity of his oncologic and hepatic status. Given his clinical information on the patient and a new CT image that was performed and uploaded, can you suggest the next steps in the management of his disease?<br><br>**Appendix:**<br>CT Abdomen, March 7th, 2024<br>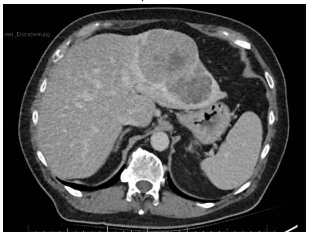<br><br>**Model Response:**<br>Given Mr. Taylor's advanced HCC with portal vein thrombosis (PVT), his preserved liver function (Child-Pugh A5), and his good performance status (ECOG PS 0), a multidisciplinary approach is crucial for optimizing his treatment strategy [1], [5], [8]. The presence of PVT categorizes his HCC as advanced or BCLC stage C, for which systemic therapy is recommended over locoregional therapies [3], [4], [59].<br>First-line systemic therapy with atezolizumab plus bevacizumab is recommended for patients like Mr. Taylor, who have advanced HCC, no contraindications to bevacizumab, and preserved liver function [60], [93]. This recommendation is based on the IMbrave150 trial, which demonstrated a significant survival benefit of atezolizumab-bevacizumab over sorafenib [93]. However, given the presence of esophageal varices, it is imperative to ensure that these have been appropriately managed to minimize the risk of variceal bleeding, a known complication associated with bevacizumab due to its antiangiogenic effects [70], [71].

No.	Patient Case Description
	Considering the progression of his disease despite previous TACE sessions, further locoregional therapies such as additional TACE or radioembolization (TARE) are unlikely to provide significant benefit and may not be appropriate given the extent of his PVT [10], [63]. However, if systemic therapy is contraindicated or not tolerated, and Mr. Taylor's PVT is classified as Vp1 or Vp2 (involving segmental or subsegmental branches of the portal vein), locoregional therapies could be reconsidered in a highly selective manner [7], [63].<br>Clinical trial enrollment should be explored for Mr. Taylor, given the rapidly evolving landscape of HCC treatment, which may offer access to novel therapeutic agents or combinations not yet available in standard practice [2], [22]. This approach is particularly relevant for advanced HCC, where emerging treatments continue to show promise.<br>Supportive care measures, including management of ascites and screening for esophageal varices, should be optimized. Therapeutic paracentesis may be required for symptomatic ascites, and beta-blockers or endoscopic variceal ligation (EVL) should be considered for variceal prophylaxis [80], [81], [82]. Given his portal hypertension, as evidenced by splenomegaly and ascites, careful monitoring for complications such as spontaneous bacterial peritonitis (SBP) is warranted [85].<br>Regular monitoring of Mr. Taylor's liver function, tumor markers, and imaging studies is essential to assess the response to any new treatment and adjust the management plan as necessary [6], [24]. Palliative care consultation should also be involved early to manage symptoms and support quality of life [6].<br>In summary, the next steps in managing Mr. Taylor's disease should include consideration of systemic therapy with atezolizumab plus bevacizumab, exploration of clinical trial enrollment, and supportive care measures tailored to his specific needs and disease status.<br>To further refine our approach and ensure the most accurate and personalized treatment recommendations for Mr. Taylor, could you please supply the path to the histologic or histopathology images of Mr. Taylor's liver tumor? This will enable a detailed analysis to identify potential genetic markers critical for his treatment planning. Additionally, if you could list any specific genetic alterations already identified in Mr. Taylor's case, it would greatly enhance our ability to query targeted treatment options and relevant clinical trials. For the most recent imaging studies, specifying the regions of interest would allow for precise measurements and better assessment of the tumor's response to treatment over time. Lastly, providing specific search terms related to Mr. Taylor's condition and any known mutations will facilitate a more focused literature search to support evidence-based decision-making.
W	Mrs. Williams, born on September 5, 1967, and identified as BC.33-2, is a 57-year-old woman who was diagnosed with colorectal cancer in 2020. Following her diagnosis, she underwent surgical removal of the tumor but experienced a prolonged hospital stay due to complications of postoperative pneumonia and sepsis. She was treated with adjuvant chemotherapy as part of her recovery process. Up until November of the previous year, Mrs. Williams was under consistent follow-up care. During one of these follow-up visits, imaging revealed the presence of a solitary metastasis in her liver, as detailed in the accompanying radiology report:<br>***<br>**Radiology Report, CT Abdomen November 2023**<br>Mrs Williams, *03/05/1966<br>Procedure: CT Abdomen with iv Contrast<br>Clinical Information: Post colorectal cancer, status post total mesorectal excision. Evaluation for metastasis.<br>Technique: Multi-phase imaging of the abdomen was performed following the administration of intravenous contrast material.<br>Findings:

No.	Patient Case Description
	Liver: A single hypodense lesion noted in the left lobe of the liver, measuring approximately 0.5 cm in its greatest dimension (Location: [250, 185, 295, 230]). This finding is suggestive for liver metastasis post colorectal cancer. No additional focal liver lesions are identified.<br>Biliary Tree: The intrahepatic and extrahepatic bile ducts are not dilated. The gallbladder is unremarkable.<br>Pancreas: The pancreas is normal in size, contour, and enhancement pattern. No mass, cyst, or ductal dilatation is seen.<br>Spleen: The spleen is normal in size and attenuation with no focal lesions.<br>Adrenal Glands: Both adrenal glands are normal in size and morphology.<br>Kidneys: Both kidneys are normal in size, shape, and function without evidence of mass, stone, or hydronephrosis. Small cyst on the left kidney, bosniak 1.<br>Bowel: There is no evidence of bowel obstruction or wall thickening. The visualized portions of the colon, including the anastomosis site, show no signs of local recurrence.<br>Vessels: The abdominal aorta and its major branches are patent without evidence of aneurysm or significant stenosis.<br>Lymph Nodes: No enlarged abdominal or pelvic lymph nodes are identified.<br>Peritoneum: There are no signs of peritoneal implants or significant ascites.<br>Impression:<br>1 small hypodense lesion in the left lobe of the liver, suggestive of liver metastasis. Given the clinical history, further evaluation with MRI or follow-up imaging may be warranted for characterization.<br>No evidence of local recurrence post total mesorectal excision for colorectal cancer.<br>No other significant abnormality detected in the abdomen.<br>Notes: Uncomplicated biopsy of a liver lesion and dispatch to pathology.<br>***<br>Following the discovery of liver metastasis, Mrs. Williams underwent six cycles of chemotherapy using the FOLFOX regimen and Panitumumab. Subsequent imaging (CTA_02_2024.png) confirmed the metastatic lesion remained stable in location (coordinates: 250, 185, 295, 230), with these findings documented in our system. Genetic analysis revealed the presence of a BRAF V600E mutation in her tumor.<br><br>**Doctor:** What is the optimal management approach for this patient? Develop a detailed treatment plan. Should we consider local treatment options such as surgical intervention or liver-directed therapies?<br><br>**Appendix:**

CT Abdomen, November 2023

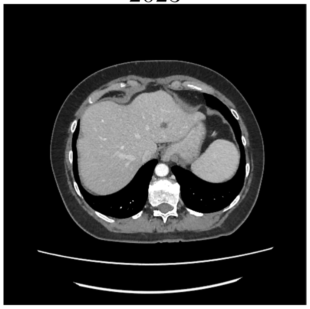

CT Abdomen, February 2024

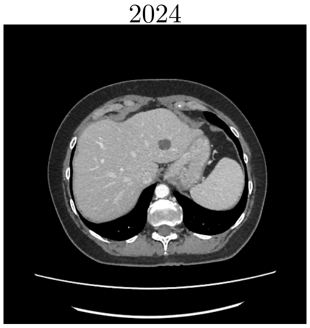

No.	Patient Case Description

Histology[5]

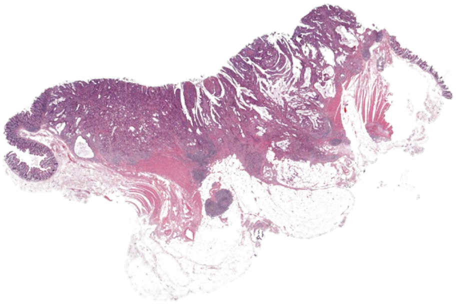

Model Response:
Given Mrs. Williams' progressive disease under the FOLFOX regimen and Panitumumab, as evidenced by the growth of her liver metastasis, the next steps involve a comprehensive reassessment of her treatment plan based on her genetic profile and current medical guidelines. The increase in the size of the metastasis, with a ratio of approximately 1.799, clearly indicates progressive disease [Tool].
1. Targeted Therapy for BRAF V600E Mutation: Considering the BRAF V600E mutation, the combination of encorafenib and cetuximab is recommended. This combination is FDA-approved for mCRC with a BRAF V600E mutation, after prior therapy [12], [13], [41]. This approach is supported by the efficacy shown in the BEACON CRC trial, which demonstrated a significant improvement in overall survival and response rate with this combination [3], [41].
2. Immunotherapy Consideration: The MSI-High status of the tumor suggests a potential benefit from immunotherapy. Pembrolizumab, recommended as first-line therapy for patients with dMMR mCRC by the ASCO 2022 guideline [20], could be considered given the patient's genetic profile. This option should be discussed with Mrs. Williams, considering the potential for a better response to immunotherapy in MSI-High status tumors [19], [20], [22].
3. Local Treatment Options: For the solitary liver metastasis, exploring local treatments such as radiofrequency ablation (RFA), stereotactic body radiotherapy (SBRT), or selective internal radiation therapy (SIRT) could be beneficial [46], [47], [48]. These options might offer localized control of the metastasis, especially if systemic therapy options are exhausted or if the metastasis becomes more localized.
4. Systemic Therapy Alternatives: If targeted therapy and immunotherapy are not immediately pursued or if further progression is noted, considering FOLFIRI as a second-line therapy could be an option [76]. However, given the genetic makeup of Mrs. Williams' tumor, targeted therapy options should be prioritized over switching to another chemotherapy regimen [5], [7].
5. Multidisciplinary Team Discussion: A multidisciplinary discussion involving medical oncologists, surgical oncologists, and radiologists is crucial to tailor the treatment plan to Mrs. Williams' specific condition, genetic makeup, and preferences [56], [57]. This team approach ensures that all potential treatment avenues are explored and that the chosen strategy aligns with the patient's goals and quality of life considerations.
6. Patient Consultation: It is essential to discuss all potential treatment options with Mrs. Williams, including the benefits, risks, and potential side effects of each treatment [6]. Her preferences, quality of life considerations, and any concerns should be central to the decision-making process.
In summary, the immediate next steps involve prioritizing targeted therapy with encorafenib and cetuximab, exploring immunotherapy options due to the MSI-High status, and considering local treatment options for the liver metastasis. A multidisciplinary approach and patient-centered discussion are paramount in deciding the future course of treatment for Mrs. Williams.

No.	Patient Case Description
	To enhance our exploration of second-line therapy options and local treatment possibilities for Mrs. Williams, it would be beneficial if you could provide the exact search terms or phrases you have in mind for investigating the effectiveness of FOLFIRI in BRAF V600E mutated colorectal cancer and the latest advancements in local treatment options for liver metastasis. Additionally, specifying the particular aspects of colorectal cancer treatment you're most interested in could greatly assist in tailoring the search to yield the most relevant and up-to-date guidelines and research findings. This focused approach will ensure we utilize our resources to gather the most pertinent information to aid in Mrs. Williams' treatment planning.
X	Ms. Xing, a 33-year-old patient, was diagnosed with cholangiocellular carcinoma in early 2023. Following her diagnosis, she underwent a complete surgical resection (R0) and was treated with adjuvant chemotherapy using capecitabine for six months. Subsequent imaging showed no evidence of disease recurrence until September of the same year, when MRI imaging ("September2023.png") revealed a new, solitary metastasis. In response, a treatment regimen of gemcitabine and cisplatin was initiated. Considering Ms. Xing's young age, a liver biopsy was performed for comprehensive panel diagnostics. Both imaging results and panel findings are made available for review:<br>***<br>**Radiology Report, MRI Abdomen September 2023**<br>Mrs Xing, *02/03/1987<br>Procedure: MRI Abdomen with iv Contrast<br>Clinical Question: Post Cholangiocellular carcinoma, new metastasis? Recurrence? Staging.<br>Technique: MRI Abdomen<br>Findings:<br>Liver: There is one single hypointense lesion in the left lobe of the liver, measuring approximately 0.4 cm in its greatest dimension (Location: [475, 250, 490, 275]). Highly suggestive for recurrence of the known cholangiocellular carcinoma.<br>Biliary Tree: The intrahepatic and extrahepatic bile ducts are not dilated.<br>Pancreas: The pancreas appears regular in size, shape, and enhancement, showing no abnormalities such as masses, cysts, or dilation of ducts.<br>Spleen: The spleen is within normal limits regarding size and density, with no detected lesions.<br>Adrenal Glands: Both adrenal glands present a normal appearance in terms of size and structure.<br>Kidneys: The kidneys are normal in all aspects of size, contour, and functionality, with no signs of masses, stones, or hydronephrosis.<br>Bowel: No obstructions or abnormal thickening is observed in the bowel.<br>Vessels: The abdominal aorta and its main branches are unobstructed, with no aneurysms or significant narrowing observed.<br>Lymph Nodes: There is no enlargement of the abdominal or pelvic lymph nodes.<br>Peritoneum: No evidence of peritoneal implants or significant fluid accumulation is present.<br>Impression:<br>Strong suspicion of a recurrence of the known CCC due to a solitary metastasis in the left liver.<br>**Molecular Report Summary:**<br>Microsatellite-Instability High, Mutation in BRAF V600E and CD74-ROS1 variant fusion. No alterations in NTRK or FGFR2.<br>***<br>Currently, the patient experiences recurrent ascites with notable abdominal tension.<br>Also, a new MRI-scan was performed yesterday ("February2024.png") by our in house radiologists.

No.	Patient Case Description
	According to the latest radiology report, the MRI scan performed on the patient with the system ID X-09.22 and documented as ”February2024.png” revealed a single metastatic lesion in the left lobe of the liver, delineated by the coordinates 455, 270, 505, 320. The report emphasizes the importance of a comprehensive review by comparing these findings with the images from a previous examination in September 2023 during the forthcoming tumor board meeting for a detailed analysis. Also, the imaging findings suggest the presence of peritoneal carcinomatosis. Her system ID is X-09.22.

Doctor: Please investigate the current state of the disease in detail. What does the new MRI scan show? Did the metastasis grow? In case of progress, what targeted or chemotherapy options could we suggest for the tumor board tomorrow? What treatment options does the patient still have according to the official guidelines for cholangiocellular cancer given her medical history. Be aware that the patient is extremely young and we need all options we could find. Also check any options you can find on google or pubmed.

Appendix:

MRI Abdomen, September 2023

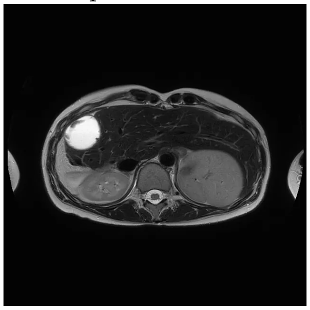

MRI Abdomen, February 2024

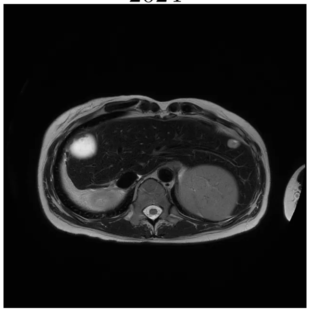

Histology

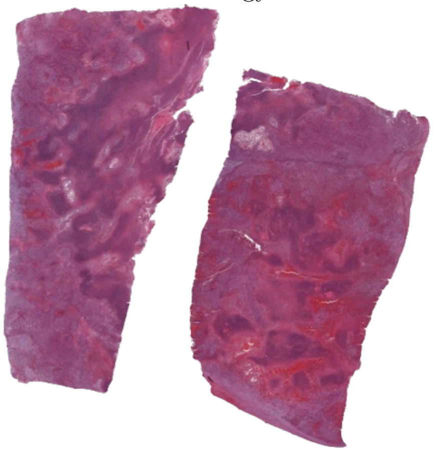

Model Response:

The latest MRI scan from February 2024 reveals that the metastatic lesion in Ms. Xing's left liver lobe has notably enlarged, growing from an area of 133 in September 2023 to 518, signifying a progression in her condition. This significant increase highlights the critical need to consider further treatment avenues for Ms. Xing.

Given Ms. Xing's medical history and the molecular characteristics of her tumor, including the BRAF V600E mutation and CD74-ROS1 variant fusion, several targeted therapy options emerge:

No.	Patient Case Description
	**1. Dabrafenib and Trametinib:** This combination is recommended for BRAF V600E-mutated tumors, including cholangiocellular carcinoma, based on their effectiveness in other cancers with similar genetic alterations [Tool]. The combination has FDA approval for BRAF V600E mutant unresectable or metastatic melanoma and other solid tumors excluding colorectal cancer, with noted effectiveness in biliary tract cancer [Tool].<br>**2. Crizotinib, Entrectinib, and Repotrectinib:** These drugs are identified treatments for ROS1-positive tumors, primarily in NSCLC. Given the CD74-ROS1 fusion in Ms. Xing's tumor, these options could be explored for their potential applicability, despite the primary association with NSCLC [Tool].<br>**3. Encorafenib and Binimetinib:** This combination, highlighted through a Google search, could be considered based on their effectiveness in cancers with BRAF V600E mutations [Tool]. Although not specifically indicated for cholangiocellular carcinoma, their use in similar genetic contexts suggests potential applicability.<br>Regarding chemotherapy options, the current guidelines for cholangiocellular carcinoma recommend systemic chemotherapy as the primary treatment for advanced disease. Given Ms. Xing's prior treatment with gemcitabine and cisplatin, alternative regimens could include:<br>- **FOLFOX** (fluorouracil, leucovorin, and oxaliplatin) as a second-line treatment option, especially for patients who have progressed on a gemcitabine-based regimen [55].<br>For Ms. Xing, considering her young age and the urgent need for effective management strategies, participation in clinical trials exploring novel agents or combinations should be strongly considered. This includes trials for targeted therapies based on her tumor's molecular profile or emerging immunotherapy options for cholangiocellular carcinoma.<br>Given the conflicting information on microsatellite status, further clarification is needed. The 'check_mutations' tool indicated Microsatellite Stability (MSS), however, the patient's molecular report specified that there was Microsatellite Instability (MSI). If MSI-High status is confirmed, immunotherapy with pembrolizumab could be considered, as it has shown effectiveness in MSI-High tumors across various cancer types [57].<br>In summary, for the tumor board discussion, the recommended approach includes exploring targeted therapy options such as Dabrafenib and Trametinib for the BRAF V600E mutation and potentially Crizotinib or Entrectinib for the CD74-ROS1 fusion. Additionally, considering second-line chemotherapy options and the possibility of clinical trial participation are crucial steps. The significant progression of Ms. Xing's disease necessitates a prompt and comprehensive evaluation of these treatment strategies to optimize her care.<br>To refine our approach and ensure the most accurate and personalized treatment recommendations for Ms. Xing, could you please provide the latest comprehensive genomic profiling of her tumor? This includes any recent sequencing data that might reveal additional actionable mutations or variants not previously identified. Additionally, detailed information regarding her response to past treatments, including any side effects experienced and the duration of response, would greatly enhance our ability to tailor future therapy options. This data is crucial for leveraging our resources to their fullest potential in guiding Ms. Xing's treatment plan.
Z	Mr. Zu, a 55-year-old individual, has been diagnosed with pancreatic adenocarcinoma based on histological evidence. Unfortunately, we don't have access to the imaging that led to this diagnosis (classified as T2N1MX). Just yesterday, he underwent comprehensive staging, which included an IV-contrast-enhanced CT scan of the abdomen. While the radiology report is still pending, the images are available for review. Clinically, Mr. Zu is in robust health and is deemed a suitable candidate for a range of therapeutic interventions.

No.	Patient Case Description

Doctor: Considering Mr. Zu's diagnosis and the current stage of his pancreatic adenocarcinoma, what specific treatment recommendations should we prepare for discussion at tomorrow's tomor board meeting?

Appendix:

CT Abdomen, February 2024[6]

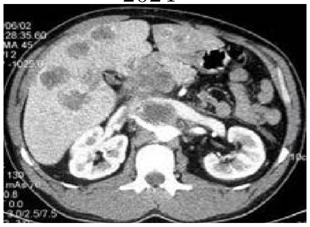

Model Response:

Given Mr. Zu's diagnosis of pancreatic adenocarcinoma with potential liver metastases as indicated by the presence of multiple hypodense areas within the liver [Tool], a comprehensive and multidisciplinary approach is warranted. The treatment strategy should balance aggressive management of metastatic disease with considerations for Mr. Zu's quality of life and preferences. Here's a detailed treatment plan based on current medical guidelines and the specific patient context at hand:

1. Confirm Metastatic Status: Before finalizing the treatment plan, it's crucial to confirm whether the hypodense liver areas represent metastatic disease. This can be achieved through further imaging, such as MRI with liver-specific contrast or PET-CT, and potentially a liver biopsy for histological confirmation [Tool]. The outcome of these investigations will significantly influence the treatment pathway.

2. Genetic Profiling: Although the mutation check encountered an error [Tool], pursuing comprehensive genetic testing is essential. This includes testing for MSI, BRAF, KRAS, and potentially other relevant markers like BRCA mutations, given the pancreatic origin of the cancer. The results could open up options for targeted therapies [21], [43].

3. Systemic Chemotherapy: If metastases are confirmed, systemic chemotherapy becomes the primary treatment approach. For a patient with a good performance status like Mr. Zu, a regimen like FOLFIRINOX (if tolerated) or gemcitabine plus nab-paclitaxel could be considered, as these are standard options for metastatic pancreatic adenocarcinoma [13], [19]. The choice between these regimens should consider Mr. Zu's overall health, comorbidity profile, and personal preferences.

4. Local Control Options: If the liver metastases are limited in number and the primary tumor is controlled, local therapies such as stereotactic body radiotherapy (SBRT) for liver metastases could be discussed. This approach could potentially improve quality of life and overall survival [23], [26].

5. Supportive Care: Integrating palliative care early in the treatment plan is crucial to manage symptoms and improve quality of life. This includes pain management, nutritional support, and addressing any psychological needs [111], [112].

6. Clinical Trials: Exploring the availability of clinical trials that Mr. Zu may be eligible for is important. This could include trials focusing on novel therapies or combinations that could offer benefit beyond standard care [154].

7. Multidisciplinary Evaluation: Ensure that Mr. Zu's treatment plan is discussed in a multidisciplinary tumor board meeting, incorporating input from medical oncology, surgical oncology, radiation oncology, radiology, and palliative care to formulate a comprehensive and individualized treatment plan [150].

8. Follow-Up and Monitoring: Plan for regular follow-up to assess treatment response, manage side effects, and adjust the treatment plan as necessary. This includes monitoring tumor markers and imaging studies [78].

No.	Patient Case Description
	Given the complexity of Mr. Zu's case, especially with the potential presence of liver metastases, it's imperative that the treatment plan is flexible and responsive to the evolving nature of his condition. Further, the limitations encountered in genetic profiling necessitate a reattempt or alternative methods to ensure a comprehensive understanding of the tumor's genetic landscape, which could significantly impact treatment decisions [Tool]. To further refine our approach and ensure we're leveraging the most accurate and personalized treatment options for Mr. Zu, I would kindly ask for the re-submission of histologic or histopathology images. Specifically, high-resolution images of the affected tissue would be invaluable. These images are crucial for a detailed genetic modeling analysis, aiming to identify MSI, BRAF, and KRAS mutations with precision. Additionally, if you could provide any available clinical data regarding Mr. Zu's health status, including any previous treatments and their outcomes, it would greatly assist in tailoring the query to the OncoKB database. This information will enable us to pinpoint targeted treatment options that are most likely to benefit Mr. Zu, based on his unique genetic profile and clinical scenario.
Ad	Mrs. Abena Adomako is a black African American, 58 years old, with metastatic (pulmonary and to the liver) left sided, colorectal cancer. She now consults our oncology department for a second opinion. She brings the following documents:<br>*** Patient Report: Diagnosis: Metastatic colorectal cancer with liver and lung metastases Mutation Status: KRAS wild-type (from previous biopsy) (other changes not identified due to lack of material) Disease Progression and Treatment History: The patient was initially diagnosed with metastatic colorectal cancer in June 2022. She received first-line chemotherapy with FOLFOX and panitumumab, resulting in partial disease control.<br>CT Abdomen, Report for Mrs Adomako Date: November 15, 2022 Hospital: Johns Hopkins Hospital CT Abdomen:<br>Liver: One metastasis in location 330, 440, 460 and 580. Vessels: No abnormalities noted in the aorta or its major branches. The venous structures, including the inferior vena cava, appear patent without signs of thrombosis or stenosis. Vertebrae: The alignment of the cervical, thoracic, and lumbar vertebrae appears normal. There are no signs of compression fractures, degenerative changes, or lesions evident in the marrow signal. Ribs: All visualized ribs are intact without evidence of fracture, deformity, or lytic lesions. Costovertebral joints are unremarkable. Stomach: The stomach shows normal contour and wall thickness. There is no evidence of distention, ulceration, or masses. The gastric folds are well-preserved. Other Organs: The spleen, pancreas, gallbladder, and adrenal glands are unremarkable with normal size and morphology. No significant lymphadenopathy or free fluid is apparent in the abdominal cavity.<br>Radiology Statement: One liver metastasis identified. Other organs and structures appear normal.<br>CT Lung: Multiple metastases.<br>***

No.	Patient Case Description
	*** CT Abdomen, Report for Mrs Adomako Date: June 10, 2024 Hospital: Moya Clinic CT Abdomen:<br>Liver: One metastasis in position: 330, 480, 440, 580. No clear progression noted due to lack of comparison with the previous scan. Vessels: The vascular structures exhibit usual caliber and wall integrity throughout the visible segments. Vertebrae: Bone density and cortical margins of the vertebrae are consistent with age and show no evidence of pathological alteration. Ribs: Cortical and trabecular patterns of the ribs are uniform, indicating normal bone architecture.<br>Stomach: Intraluminal contents are normal, and the gastric exit is clear without any obstructions or thickening. Other Organs: The liver, kidneys, and intestines display expected anatomical features with no alterations in size or texture suggestive of disease.<br>Lungs: There is no change in the previously noted metastatic deposits, indicating stability with no progression of the disease. No additional pulmonary nodules or pleural effusions are observed.<br>Radiology Statement: One liver metastasis observed. Comparison with the previous CT abdomen scan is required to assess progression. Lung metastases are stable. ***<br>Mrs. Adomako recently underwent a new liver biopsy for pathology workup. The latest CT scan was performed concurrently at the Moya Clinic. We await the pathology results to guide further treatment. The patient is registered on our system under the ID A1-002-YXA. The images are named "November15_2022.png" and "June10_2024.png".<br><br>**Doctor:** Could you please assess the current state of Mrs. Adomako disease based on the most recent imaging and clinical data? Can you measure precisely if the described metastasis has grown or responded? By which factor did it change? Does she have any molecular targets according to the histology? Could you develop a detailed treatment plan for Mrs. Adomako ongoing care and management, considering her current disease status and biopsy results?

Appendix:

CT, November 15, 2022

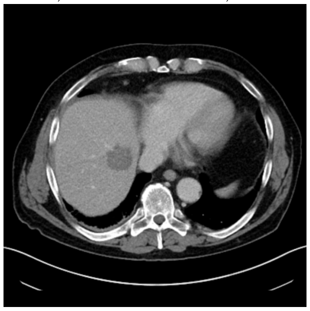

CT, June 10, 2024

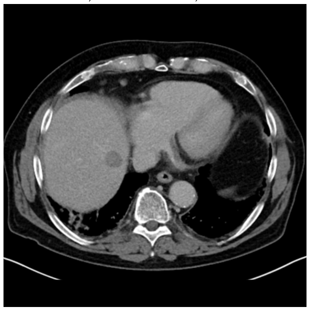

No.	Patient Case Description

Histology

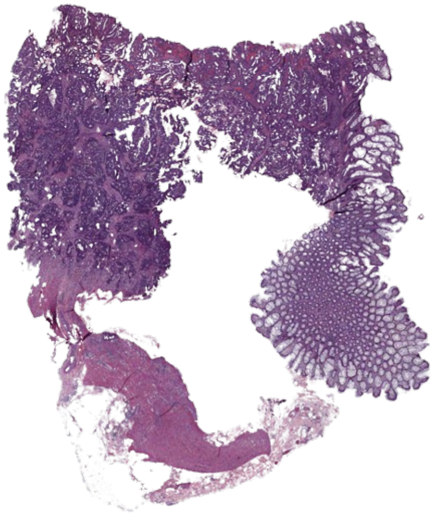

Model Response:
Based on the comprehensive analysis of Mrs. Abena Adomako's medical history, recent imaging, and tool results, we can conclude that her metastatic colorectal cancer has shown a significant positive response to the treatment regimen administered between November 2022 and June 2024. The liver metastasis has decreased in size by approximately 2.26 times, indicating a substantial reduction and suggesting effective disease control during this period [Tool]. The genetic predictions from the histopathology images reveal that Mrs. Adomako has Microsatellite Stability (MSS), a wild-type BRAF gene, and a mutated KRAS gene [Tool]. This genetic profile suggests that while she may not benefit from immunotherapies targeting MSI-high tumors or treatments targeting BRAF mutations, she could be eligible for targeted therapies aimed at KRAS mutations.
Given Mrs. Adomako's current disease status and biopsy results, the following detailed treatment plan is proposed for her ongoing care and management:
1. **Continue Monitoring**: Regular imaging studies should be continued to monitor the disease's response closely and detect any signs of progression [Patient].
2. **Targeted Therapy for KRAS Mutation**: Considering the presence of a KRAS mutation, targeted therapy options such as cetuximab or panitumumab could be considered. These agents have shown efficacy in KRAS wild-type colorectal cancer; however, emerging therapies targeting specific KRAS mutations (e.g., sotorasib for KRAS G12C mutation) may also be explored if the specific type of KRAS mutation is identified and matches available targeted therapies [3], [4].
3. **Chemotherapy Regimen Adjustment**: Based on the positive response to her previous treatment, maintaining a similar chemotherapy backbone (e.g., continuation of FOLFOX if tolerated) with adjustments to incorporate targeted therapy against KRAS mutation could be beneficial [Patient].
4. **Clinical Trials**: Enrollment in clinical trials exploring new treatments or targeted therapies for KRAS-mutated metastatic colorectal cancer should be considered, especially those focusing on novel KRAS inhibitors or combination therapies [Patient].
5. **Supportive Care**: Symptom management and supportive care remain crucial to maintaining Mrs. Adomako's quality of life. This includes managing any side effects from treatment, nutritional support, and psychological support [Patient].

No.	Patient Case Description
	6. **Multidisciplinary Team Review**: Mrs. Adomako's case should be regularly reviewed by a multidisciplinary team, including medical oncologists, surgical oncologists, radiologists, and genetic counselors, to adapt the treatment plan as her disease status and the landscape of colorectal cancer treatment evolve [Patient].<br>This treatment plan is based on the current understanding of Mrs. Adomako's disease status and available medical evidence. It is crucial to re-evaluate her treatment plan regularly, considering any new clinical findings, patient preferences, and emerging therapies in the field of metastatic colorectal cancer.<br>To enhance the precision of Mrs. Adomako's treatment strategy, could you supply the exact KRAS mutation subtype detected in her biopsy? This detail is pivotal for identifying the most effective targeted therapy options. Additionally, providing the latest imaging studies' results would allow for a more detailed analysis of the disease's progression or response to the current treatment, ensuring a tailored approach to her ongoing care.
An	Name: Mrs. Anderson<br>Age: 62<br>Ethnicity: Caucasian<br>Diagnosis: Liver metastatic colorectal cancer<br>Initial Diagnosis Date: January 2022<br>Medical History:<br>Hypertension, managed with lisinopril<br>Hyperlipidemia, managed with atorvastatin<br>Treatment Timeline:<br>Month-Year Event/Intervention<br>January 2022 Initial diagnosis of colorectal cancer with liver metastasis.<br>February 2022 - May 2022: First-line therapy with FOLFOX and panitumumab.<br>June 2022 CT scan abdomen: Stable disease.<br>July 2022 - October 2022 Continued FOLFOX and bevacizumab.<br>November 2022 CT scan abdomen: Progressive disease noted.<br>December 2022 - March 2023 Switched to second-line therapy with FOLFIRI and bevacizumab.<br>April 2023 CT scan abdomen: Stable disease.<br>May 2023 - August 2023 Continued FOLFIRI and bevacizumab.<br>September 2023 CT scan abdomen: Progressive disease noted.<br>October 2023 - January 2024 Switched to third-line therapy with regorafenib.<br>February 2024 CT scan abdomen: Stable disease.<br>March 2024 - June 2024 Continued regorafenib.<br>July 2024 Latest imaging: CT scan abdomen performed last week, report pending.<br>Current Status:<br>Mrs. Anderson is currently feeling well with an ECOG performance status of 1. Her liver function remains stable, and she continues to be active with minimal symptoms. Results from a recent tumor biopsy are pending. Her patient ID in the system is "XX-01-134".<br><br>**Doctor:** Could you please evaluate the current state of Mrs. Anderson's disease based on the most recent imaging, microscopic and clinical data? What treatment options should be recommended for Mrs. Anderson at this stage?

No.	Patient Case Description
	**Appendix:**

CT February 2024

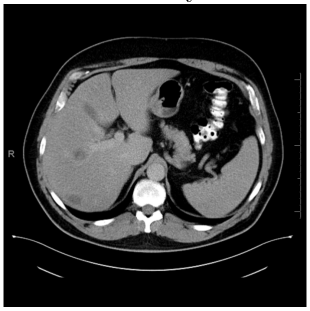

CT July 2024

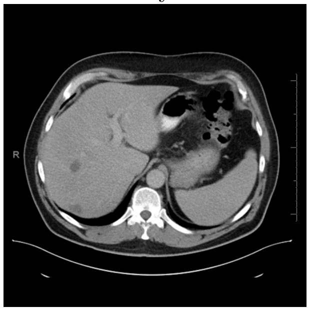

Histology

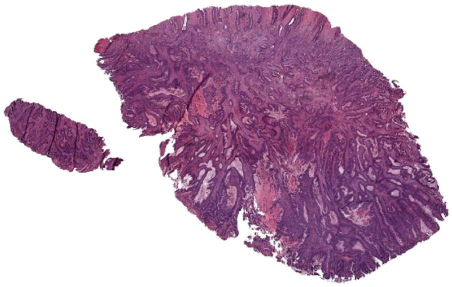

Model Response:
Based on the comprehensive analysis of Mrs. Anderson's current disease state, including her medical history, treatment timeline, recent imaging, and genetic mutation analysis, we can deduce that her metastatic colorectal cancer (mCRC) has shown progression despite previous lines of systemic therapy. The most recent imaging indicates a significant burden of liver metastases, with the largest lesion being 5 cm in diameter. However, there are no new metastases outside the liver, and the surrounding organs are stable with no significant abnormalities [Patient]. The genetic mutation analysis reveals that Mrs. Anderson's cancer is MSS, and both BRAF and KRAS are wild-type [Tool].
Given Mrs. Anderson's MSS status and wild-type BRAF and KRAS, along with the progression of liver metastases after multiple lines of systemic therapy, including regorafenib, the following treatment options should be considered:
1. **Local Therapies for Liver Metastases**: Considering the liver-dominant nature of her disease and the presence of a large 5 cm lesion, local therapies such as radiofrequency ablation (RFA), stereotactic body radiotherapy (SBRT), or transarterial chemoembolization (TACE) could be considered to control the liver disease and potentially improve her quality of life [25][26][27][28]. These options aim to target the liver metastases directly and could be particularly beneficial given the stable nature of disease outside the liver.
2. **Clinical Trials**: Mrs. Anderson should be evaluated for eligibility in clinical trials targeting MSS, BRAF, and KRAS wild-type metastatic colorectal cancer. Given the advancements in cancer therapy and ongoing research, participation in a clinical trial could provide access to novel treatments not yet available in the standard care setting [3].
3. **Immunotherapy Evaluation**: Although traditional immunotherapy options might have limited efficacy in MSS colorectal cancer, it's worth reviewing any new evidence or ongoing trials that Mrs. Anderson might be eligible for, especially those exploring novel immunotherapeutic approaches or combinations that could be effective in MSS contexts [13][14][15].

No.	Patient Case Description
	4. **Palliative Care Integration**: Palliative care should be integrated into Mrs. Anderson's treatment plan to manage symptoms, address quality of life concerns, and provide support for her and her family. This is crucial as the disease progresses and treatment becomes more focused on symptom management [5][6].<br>5. **Regular Follow-Up and Re-assessment**: Mrs. Anderson should have regular follow-up appointments to monitor her response to any new treatment, manage side effects, and adjust her treatment plan as needed. This includes regular imaging to monitor disease progression and response to treatment [Patient].<br>6. **Patient and Family Communication**: Ensure open and ongoing communication with Mrs. Anderson and her family about her treatment options, prognosis, and any decisions regarding her care. It's important that she participates in decision-making processes and that her preferences and values are considered [Strategy].<br>Given Mrs. Anderson's performance status of 1 and the stable liver function, she may still be a candidate for systemic therapy adjustments based on the outcomes of the discussed local therapy options and/or clinical trial participation. The absence of actionable mutations (BRAF, KRAS) and MSS status guide us away from certain targeted therapies but open the door for other innovative treatments through clinical trials.<br>To enhance our ability to tailor Mrs. Anderson's treatment plan with precision and explore the most promising avenues for her condition, it would be beneficial if you could supply the exact mutation details, including any minor mutations not previously mentioned. This information is crucial for a more targeted query that could reveal potential therapies specifically suited to her genetic profile. Additionally, if you could provide specific terms or keywords that you believe are most relevant to Mrs. Anderson's case, especially those pertaining to innovative treatments for liver metastatic colorectal cancer in the context of her genetic makeup, this would greatly assist in a focused literature search to uncover the latest and most relevant studies. Lastly, for a detailed analysis of the liver lesions, kindly provide the latest imaging files along with precise coordinates or descriptions for the lesions' locations. This will enable a thorough quantification and assessment of the disease's progression, which is vital for planning the next steps in her treatment.
Ch	Mrs. Chen, a 64-year-old Chinese native, was diagnosed with colorectal cancer in March 2022. Initially, she underwent surgical resection followed by adjuvant chemotherapy with FOLFOX. Despite this, follow-up imaging in March 2023 revealed small lung metastases, prompting the initiation of systemic therapy with FOLFIRI and bevacizumab.<br>Throughout 2023 Mrs. Chen continued with the systemic therapy, showing a partial response. She now consults our oncology department for a second opinion. She brings the latest two radiology reports:<br>***<br>Radiology Report for Mrs Chen:<br>Date: February 3, 2024<br>Hospital: Shanghai General Hospital<br>CT Chest:<br>Lungs: The lung parenchyma is clear with no signs of infection or large masses. There are multiple small metastases scattered throughout both lungs.<br>Heart: Normal size and contour.<br>Mediastinum: No lymphadenopathy or masses.<br>Pleura: No pleural effusion.<br>Radiology Statement: Multiple small lung metastases are present. These findings are consistent with known metastatic colorectal cancer. Reference lesions are in locations: 100, 650, 150, 750.<br>***<br>***<br>Radiology Report for Mrs Chen:

No.	Patient Case Description

Date: June 25, 2024
Hospital: SF Medical College Hospital
CT Chest:
Lungs: Clear lung parenchyma without signs of infection or large masses. Multiple small metastases are noted throughout both lungs. Exemplary lesions can be found in locations: 100, 650, 220, 780.
Heart: Normal size and contour.
Mediastinum: No significant lymphadenopathy or masses.
Pleura: No pleural effusion.
Radiology Statement: Small lung metastases are observed. We recommend comparing this imaging with the previous CT scans to determine any changes in the size and number of the metastatic lesions.
***
Mrs. Chen remains under close observation and continues her systemic therapy. Her overall condition is stable, with an ECOG performance status of 1. She reports feeling well, with manageable side effects from the ongoing treatment. Histopathology images should also be available on the system. The patient's ID is: "XX-03AY".

Doctor: Could you please assess the current state of Mrs. Chen's disease based on the most recent imaging and clinical data? Check the images (February_3_2024.png and June_25_2024.png). Also use your tools to measure precisely the described lesions, please. Additionally, could you develop a detailed treatment plan for Mrs. Chen's ongoing care and management?

Appendix:

CT February 3 2024

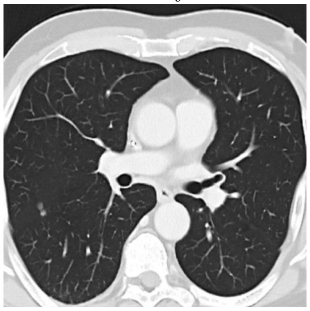

CT June 25 2024

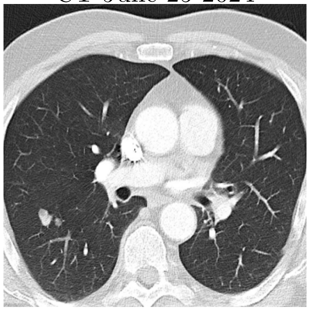

No.	Patient Case Description

Histology

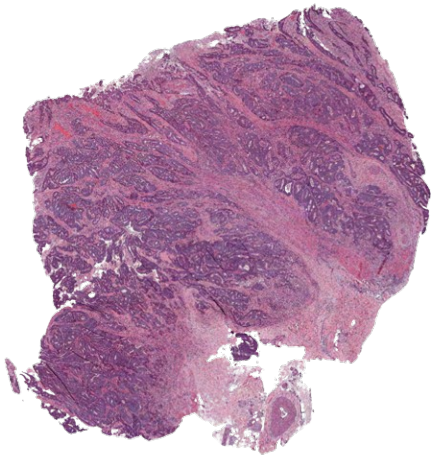

Model Response:
Based on the provided data and tool results, Mrs. Chen's metastatic colorectal cancer has demonstrated progression from February to June 2024. The significant increase in the size of lung metastases, as quantified by the segmentation tool results showing an increase in the overall area of the lesions from 702 to 2798 square units, indicates that the disease is not adequately controlled under the current regimen of FOLFIRI and bevacizumab [Tool]. Furthermore, the genetic mutation analysis revealing MSS status and the absence of BRAF and KRAS mutations provides valuable information for guiding future treatment decisions [Tool].
Given the progression of disease despite current systemic therapy, and considering Mrs. Chen's stable ECOG performance status of 1, which suggests she remains in relatively good condition to tolerate treatment, the following treatment plan is proposed:
1. **Re-evaluation of Systemic Therapy**: Given the disease progression, it is advisable to consider alternative systemic therapy options. Since Mrs. Chen's cancer is MSS and lacks BRAF and KRAS mutations, therapies that do not specifically target these mutations could be considered. Options may include:
- Switching to a different chemotherapy backbone, such as reintroducing oxaliplatin in a modified FOLFOX regimen if her neuropathy is manageable [1].
- Considering the addition of an alternative biologic agent. Given the absence of RAS mutations, anti-EGFR therapies such as cetuximab or panitumumab could be considered, despite the primary tumor's right-sided origin which typically shows less benefit from anti-EGFR therapy [1], [13], [14].
2. **Local Therapies for Lung Metastases**: Given the progression of lung metastases, exploring local treatment options such as stereotactic body radiation therapy (SBRT) for lung lesions could be considered, especially for lesions that are causing symptoms or have a significant growth [5], [57].
3. **Supportive Care**: Continue to manage side effects of therapy and provide supportive care to maintain quality of life. This includes managing any symptoms of chemotherapy-induced toxicity and providing psychological support [3].
4. **Clinical Trials**: Explore the availability of clinical trials that Mrs. Chen may be eligible for, focusing on new therapeutic agents or combinations that could be effective for her cancer's genetic and molecular profile [3].
5. **Regular Monitoring**: Continue with regular imaging every 2-3 months to monitor disease progression and response to the new treatment plan. Additionally, monitor blood work, including CEA levels, as an indicator of treatment response [3].

No.	Patient Case Description
	6. **Multidisciplinary Team (MDT) Review**: Mrs. Chen's case should be discussed in a multidisciplinary team setting, including medical oncologists, radiation oncologists, and surgical oncologists, to tailor the treatment plan based on her specific needs and preferences [3].<br>This structured strategy aims to address the progression of Mrs. Chen's metastatic colorectal cancer by considering alternative systemic therapies, potential local treatments for lung metastases, and the importance of supportive care, while also keeping an eye on new treatment opportunities through clinical trials.<br>To enhance our approach in tailoring Mrs. Chen's treatment plan, could you supply any further genetic mutation data that hasn't been previously analyzed? This information will be invaluable for a more comprehensive genetic profile assessment, potentially uncovering additional targeted therapy options. Furthermore, if you could specify particular aspects of metastatic colorectal cancer treatments you're interested in exploring˘2014especially those relevant to MSS status without BRAF and KRAS mutations˘2014this would enable a focused search for the most current and effective treatment strategies in the literature and clinical guidelines.
Da	Mr. Davis, a 60-year-old Black American, visited our oncology department for a consultation regarding his metastatic colorectal cancer, initially diagnosed in 2021. The primary tumor is located on the left side. He is EGFR wild-type and has been receiving routine therapy for his disease ever since. In 2022, due to progressive disease in the liver (the patient reports at least 3 known metastases), his treatment regimen was switched from first-line FOLFOX combined with panitumumab to second-line FOLFIRI-panitumumab. Mr Davis further explains that a CT abdomen scan performed in November 2023 showed a stable disease management, so his treatment was continued as previously. However, the most recent scan on February 22, 2024, has been conducted, but we are still awaiting the radiologist's report. Mr. Davis describes feeling generally well, with an ECOG performance status of 1, and he exhibits no specific symptoms related to the tumor. His medical history includes type 2 diabetes, managed with metformin, and a past hip replacement in 2018. During our conversation the patient further reports that a liver biopsy was performed two weeks ago, but he has no news regarding the pathologist's evaluation. The patient ID in our system is: Y-003-3.<br>**Doctor:** What is the optimal management approach for Mr. Davis at this stage? Should we consider local treatment options, such as surgical intervention or liver-directed therapies, for his condition? Could you develop a detailed treatment plan for his continued care<br><br>**Appendix:**<br>November 11, 2023 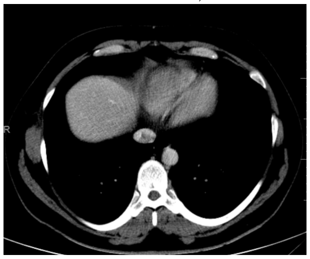 February 22, 2024 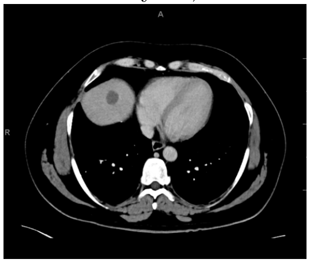

No.	Patient Case Description

Histology

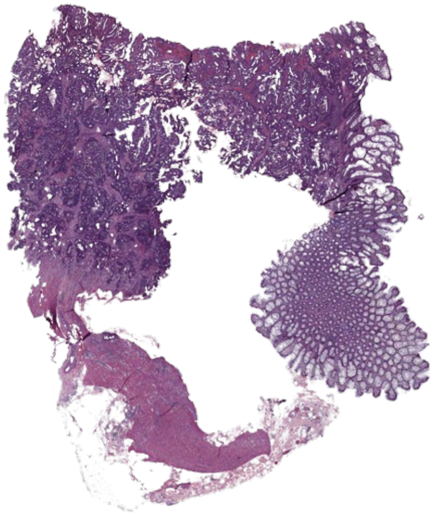

Model Response:
Given Mr. Davis's current clinical presentation, including the progression of liver metastases as indicated by the February 2024 CT scan and the presence of a KRAS mutation, a comprehensive and multidisciplinary approach is warranted for his continued care. The following strategy integrates insights from medical guidelines, the patient's diagnostic data, treatment history, and the recent tool results [Tool].
1. **Reassessment of Systemic Therapy**: Given the KRAS mutation and disease progression, Mr. Davis's current regimen of FOLFIRI-panitumumab is no longer optimal. KRAS mutations are known to confer resistance to anti-EGFR therapies such as panitumumab [1], [3], [29]. Considering his KRAS mutation status, switching to a chemotherapy regimen not previously administered, potentially combined with a VEGF inhibitor like bevacizumab, should be considered. Bevacizumab has shown efficacy in KRAS-mutated mCRC when combined with chemotherapy [23], [24].
2. **Evaluation for Liver-Directed Therapies**: Given the significant liver involvement, assessing the feasibility of liver-directed therapies is crucial. Options such as radiofrequency ablation (RFA), transarterial chemoembolization (TACE), or stereotactic body radiotherapy (SBRT) could be considered, depending on the lesion's size, location, and the patient's overall health status [11], [13], [15]. These modalities offer potential for local control and symptom management, especially in the context of systemic disease progression.
3. **Exploration of Clinical Trials**: Given the KRAS mutation, Mr. Davis may benefit from emerging therapies targeting this pathway. Clinical trials exploring novel KRAS inhibitors or combination therapies targeting other pathways in conjunction with KRAS could provide additional treatment options [3], [27].
4. **Supportive Care Measures**: Continuation of supportive care measures to manage symptoms, improve quality of life, and address any treatment-related side effects is essential. This includes pain management, nutritional support, and monitoring for potential complications related to liver metastases.
5. **Close Monitoring and Follow-Up**: Regular imaging and laboratory tests will be necessary to monitor the disease's response to the new treatment plan. This should include CT scans every 2-3 months and blood tests for tumor markers, liver function tests, and monitoring for treatment-related toxicities [35].

No.	Patient Case Description

6. **Consultation with a Multidisciplinary Team**: A multidisciplinary team discussion involving medical oncologists, hepatobiliary surgeons, interventional radiologists, and radiation oncologists is recommended to tailor the treatment plan to Mr. Davis's specific needs and to explore all viable treatment options [115], [116].

Given the complexity of Mr. Davis's case, including his medical comorbidities and the progression of his metastatic disease, this structured approach aims to optimize his treatment outcomes while maintaining his quality of life. It is imperative to have a detailed discussion with Mr. Davis regarding the potential benefits and risks of the proposed treatment options, ensuring that his preferences and values are central to the decision-making process.

To further refine our approach and ensure the most accurate and personalized treatment recommendations for Mr. Davis, could you please provide the exact path to the February 22, 2024, CT abdomen scan images? This will enable a detailed analysis to better understand the progression and characteristics of the liver metastases. Additionally, specifying the exact KRAS mutation variant Mr. Davis has will allow for a targeted search in the OncoKB database, ensuring we identify the most effective treatment options available for his specific genetic profile. Lastly, if you could share the precise search terms or queries you have in mind regarding the latest treatment options and outcomes for metastatic colorectal cancer with KRAS mutations and liver-directed therapies, it would greatly enhance our ability to retrieve the most relevant and up-to-date information to guide his treatment plan.

J

Mrs. Johnson, a 65-year-old Black American female, has a significant medical history of liver cirrhosis and progressively advancing hepatocellular carcinoma (HCC). Initially diagnosed at an early stage in March 2023, she received localized treatments, including radiofrequency ablation (RFA) and transarterial chemoembolization (TACE).

The last imaging study, taken in December 2023, showed stable disease, allowing her to take a therapy break to visit her daughter in Europe for a few weeks. She has now returned, and a follow-up imaging study was conducted in March 2024, but the radiology report is pending. Mrs. Johnson remains in good clinical condition. Her liver function, as per the Child-Pugh classification, is B7, and her Eastern Cooperative Oncology Group (ECOG) performance status is 1. Tumor markers show AFP levels at 120.5, CEA at 25.4, and Ca19-9 at 20.3.

Doctor: What are the findings of the new imaging study? Can the patient continue her treatment break based on these findings? What is the recommended treatment strategy moving forward?

Appendix:

CT December, 2023

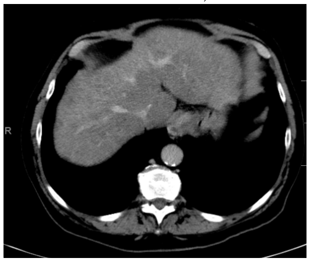

CT May, 2024

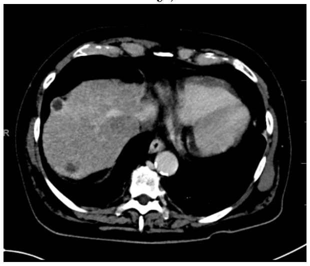

No.	Patient Case Description

Model Response:
Given the detailed analysis of Mrs. Johnson's imaging studies from December 2023 and May 2024, it is evident that there has been a significant change in her condition, suggesting disease progression. The increase in liver size and the emergence of a large, heterogeneous mass in the right lobe are indicative of aggressive tumor behavior or disease progression [Tool]. This contrasts with the stable disease observed in the December 2023 scan, marking a pivotal point in her treatment journey [Tool].
Mrs. Johnson's current clinical condition, characterized by a Child-Pugh classification of B7 and an ECOG performance status of 1, along with the tumor markers (AFP, CEA, and Ca19-9 levels), suggests that she remains in relatively good condition for potential treatment, despite the apparent disease progression [Patient]. However, the findings from the May 2024 imaging study necessitate a reassessment of her treatment plan rather than continuing the treatment break [Tool].
The recommended treatment strategy moving forward should include:
1. **Immediate Reassessment:** Convene a multidisciplinary team meeting involving hepatologists, oncologists, radiologists, and surgeons to discuss Mrs. Johnson's case in detail [1][3]. This team should review the imaging findings, her current clinical status, and liver function to understand the full scope of disease progression.
2. **Further Diagnostic Workup:** A liver biopsy of the newly identified lesion may be necessary to obtain histological confirmation of the suspected progression [3][4]. Molecular profiling of the biopsy sample could provide insights into potential targeted therapy options [5].
3. **Treatment Plan Revision:** Based on the multidisciplinary team's assessment and additional diagnostic findings, a revision of Mrs. Johnson's treatment plan is warranted. Considering the potential disease progression, systemic therapy options should be explored. Atezolizumab plus bevacizumab has shown efficacy in advanced HCC and could be considered if Mrs. Johnson's condition aligns with the eligibility criteria for this treatment [12][16]. Other molecularly targeted therapies like cabozantinib or ramucirumab, depending on the molecular profile and specific characteristics of her tumor, should also be considered [15][17].
4. **Supportive Care:** Optimize supportive care measures to manage symptoms and improve her quality of life. This includes management of any potential side effects from new systemic therapies [14].
5. **Regular Monitoring:** Establish a schedule for regular monitoring, including imaging studies and tumor marker assessments, to evaluate the response to the new treatment regimen and adjust as necessary [9].
Given the complexity of HCC management and the potential for rapid disease progression, it is crucial to act swiftly to reassess Mrs. Johnson's condition and adapt her treatment plan accordingly. Consulting with a liver transplant center may also be prudent, considering her liver function and the aggressive nature of her disease [83].
To enhance our understanding and tailor Mrs. Johnson's treatment plan more precisely, could you supply the histologic or histopathology images, specifically indicating the path to these images for genetic analysis? This will allow for a detailed exploration of potential genetic underpinnings of her disease progression. Furthermore, if you could list any known genetic mutations Mrs. Johnson has, it would enable a targeted search for the most effective treatment options. For a comprehensive evaluation of the hepatic lesion, please provide the most recent imaging files where the lesion is clearly visible, including specific coordinates if possible, to facilitate precise lesion analysis. Lastly, to ensure we are considering the most current and relevant treatment modalities, please specify any particular terms or keywords you believe should be included in our search of recent scientific literature on hepatocellular carcinoma treatment advancements.

No.	Patient Case Description
Ma	Mr Pedro Martinez, a 61-year-old South American immigrant from Argentina, presents to our oncology clinic for the first time. He is in good clinical condition and seeks continued management and evaluation of his metastatic colorectal adenocarcinoma which was diagnosed in Argentina in late 2022.<br>Initial Diagnosis:<br>Tumor Type: Colorectal adenocarcinoma<br>Stage: IV, T3N2M1 (Liver)<br>Size: Primary tumor 4.5 cm<br>Mutations: KRAS G12D, EGFR wild-type<br>Patient History:<br>December 2022 - August 2023: First Line Chemotherapy with FOLFOX + Bevacizumab in Buenos Aires<br>August 2023: CT scan Chest / Abdomen: Progression of Liver Metastases September 2023 - now: Second Line Chemotherapy with FOLFIRI and Bevacizumab<br>Here is the previous report from February 2024:<br>***<br>Liver: Three metastatic lesions identified. First one in position: 220, 620, 280, 690. The other two are located in 280, 305, 340, 380 and 440, 200, 510, 280.<br>Vessels: Hepatic and portal veins are patent with no evidence of thrombosis or other abnormalities.<br>Aorta: The aorta appears normal in caliber and contour, with no signs of aneurysm, dissection, or other pathology.<br>Ribs: Ribs are intact, without evidence of fractures, lytic lesions, or other abnormalities.<br>Lungs: There are no new pulmonary lesions observed; the lungs remain clear with no signs of metastasis or other pathology.<br>Vertebrae: Vertebral alignment and signal characteristics are normal, with no evidence of metastatic involvement or pathological fractures.<br>***<br>Here is the latest radiology report:<br>***<br>CT Chest / Abdomen Report, May 2024<br>Liver: Three metastases observed in the following locations: [230, 630, 300, 710], [260, 350, 340, 420] and [410, 230, 480, 320]. Vessels: No abnormalities noted in the hepatic or portal veins.<br>Aorta: Normal appearance with no evidence of aneurysm or dissection.<br>Ribs: Intact without any fractures or lytic lesions.<br>Lungs: No new lesions detected; no evidence of pulmonary metastases.<br>Vertebrae: Normal alignment and signal, with no evidence of metastatic disease or pathological fractures.<br>Radiology Statement: The patient did not provide previous imaging studies; therefore, therefore a direct comparison to assess disease progression is not possible.<br>***<br>The radiology images related to the reports can be found as February2024.png and May2024.png on the system.<br>**Doctor:** Please assess the current state of Mr. Martinez disease based on the most recent imaging and clinical data? Could you develop a detailed treatment plan for his ongoing care and management? Could you please check for options for targeted therapies in the future also on web sources?<br><br>**Appendix:**

No.	Patient Case Description

February 2024 May 2024

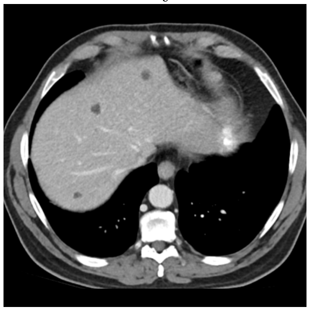

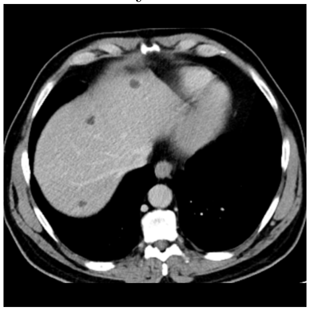

Model Response:
Given the detailed case of Mr. Pedro Martinez, a 61-year-old patient with metastatic colorectal adenocarcinoma, we have analyzed his disease progression and potential treatment pathways based on the latest imaging data and his KRAS G12D mutation status. The analysis of liver metastases from February 2024 to May 2024 shows an increase in the overall area of liver metastases from 3519 square units to 3841 square units, suggesting disease progression [Tool]. This progression is despite ongoing second-line chemotherapy with FOLFIRI and Bevacizumab, indicating the need for a reassessment of the treatment strategy.
RECIST Evaluation:
Based on the RECIST criteria, which primarily focus on changes in the longest diameter of target lesions to categorize response to treatment, the provided data indicate progression. However, precise linear measurements are necessary to formally apply RECIST criteria. The reported increase in overall area suggests progression, but without specific linear dimensions, a precise RECIST categorization cannot be provided. For a formal RECIST evaluation, the increase in the longest diameter of the lesions should be quantified. Given the data limitations, we estimate disease progression but recommend obtaining exact measurements for formal RECIST categorization [Tool].
Molecular Targets and Treatment Plan:
The presence of a KRAS G12D mutation in Mr. Martinez's tumor profile suggests specific considerations for targeted therapy. EGFR-targeted therapies such as cetuximab and panitumumab are generally ineffective in the presence of KRAS mutations, including G12D [OncoKB Analysis]. This aligns with current guidelines recommending against the use of anti-EGFR monoclonal antibodies in KRAS-mutated mCRC [6], [7].
Given the KRAS G12D mutation and the disease progression, the following treatment plan is proposed:
1. **Discontinue Current Regimen**: Given the evidence of disease progression, discontinuing the current regimen of FOLFIRI and Bevacizumab is recommended.
2. **Initiate MEK Inhibitor Therapy**: Consider starting a MEK inhibitor, such as cobimetinib or trametinib, which has shown effectiveness in cancers with KRAS mutations [OncoKB Analysis]. The choice between these agents will depend on Mr. Martinez's overall health status, potential side effects, and other clinical considerations.
3. **Explore Clinical Trials for Novel KRAS Inhibitors**: Actively seek out clinical trials for novel KRAS inhibitors (RMC-6236, MRTX-1133, ASP3082) that are specifically designed for KRAS G12D mutations. Participation in such trials could provide access to cutting-edge treatments and potentially improve outcomes [OncoKB Analysis].
4. **Supportive Care Measures**: Continue comprehensive supportive care to manage symptoms and maintain quality of life. This includes pain management, nutritional support, and addressing any psychosocial needs.

No.	Patient Case Description
	5. **Further Molecular Testing**: Perform comprehensive genomic profiling to uncover additional actionable mutations or biomarkers that could guide the selection of other targeted therapies or immunotherapies.<br>6. **Multidisciplinary Team Review**: Convene a multidisciplinary team meeting, including medical oncologists, surgical oncologists, and radiation oncologists, to review Mr. Martinez's case and explore all possible treatment options, including the feasibility of surgical resection or localized treatments for liver metastases.<br>7. **Regular Monitoring**: Implement a schedule for regular monitoring of disease progression through imaging and tumor markers to assess the effectiveness of the new treatment regimen and adjust as necessary.<br>This structured approach aims to leverage the latest in molecular targeting based on Mr. Martinez's specific genetic profile while ensuring his quality of life is prioritized through supportive care and comprehensive disease management.<br>To enhance our understanding of Mr. Martinez's condition and tailor the treatment plan more precisely, could you please provide the exact dates and file paths for the most recent CT or MRI scans? This information is crucial for a detailed analysis of disease progression and to accurately measure the response of metastases over time. Additionally, if you have access to histologic or histopathology images, kindly specify the file paths for these images as well. This will enable us to perform genetic modeling to identify potential molecular targets. Lastly, please list any known genetic mutations or alterations in Mr. Martinez's case, as this will allow us to consult the OncoKB database for the most effective targeted treatment options.
Mi	Mr. Miller, a 55-year-old Native American, was diagnosed with hepatocellular carcinoma (HCC) in January 2021. Initially, he received localized treatments, including radiofrequency ablation (RFA) and transarterial chemoembolization (TACE). Despite these efforts, the disease has required ongoing monitoring and intervention.<br>We have the following information available that the patient brings to his consultation:<br>***<br>MRI Report for Mr Miller, born April 7, 1969:<br>Date: December 3, 2023<br>Hospital: LA Medical Center<br>MRI Abdomen:<br>Liver: There is a contrast-enhanced lesion in location 480, 350, 550, 450.<br>Spleen: Normal size and appearance, no focal lesions observed.<br>Vertebrae: Normal alignment and bone marrow signal, no evidence of metastasis.<br>Ribs: Intact without lytic or sclerotic lesions.<br>Stomach: Normal wall thickness, no masses or abnormal enhancement.<br>Aorta: Normal caliber and course, no aneurysms or dissection.<br>***<br>***<br>MRI Report for Mr Miller, born April 7, 1969:<br>Date: February 28, 2024<br>Hospital: Cancer Health Care Center<br>MRI Abdomen:<br>Liver: The previously identified HCC lesion in similar location as previously described, currently at position [510, 330, 570, 400].<br>Spleen: Normal size and appearance, no focal lesions observed.<br>Vertebrae: Normal alignment and bone marrow signal, no evidence of metastasis.<br>Ribs: Intact without lytic or sclerotic lesions.<br>Stomach: Normal wall thickness, no masses or abnormal enhancement.<br>Aorta: Normal caliber and course, no aneurysms or dissection.

No. Patient Case Description

Conclusion: The HCC lesion in location [510, 330, 570, 400] is present. A comparison with the December 3, 2023, MRI is necessary to assess the state of the disease.
***
Mr. Miller is currently under regular monitoring as per patient wish. He reports feeling generally well with minimal symptoms. His liver function, assessed using the Child-Pugh classification, is at A5. His ECOG performance status is 1, recent lab results include AFP at 75.3, CEA at 10.5, and Ca19-9 at 12.4. Mr. Miller has a history of hypertension, managed with amlodipine.
His images (MRI_December_3_2023.png and MRI_February_28_2024.png) are available in the system for review.
Doctor: Could you evaluate the current state of Mr. Miller's disease based on the latest imaging and clinical data? Did it progress according to RECIST criteria? Please have a look at the images MRI_December_3_2023.png and MRI_February_28_2024.png. By how much percent did the tumor volume change precisely? What treatment options are available for Mr. Miller at this stage of his disease?

Appendix:

MRI December 3, 2023

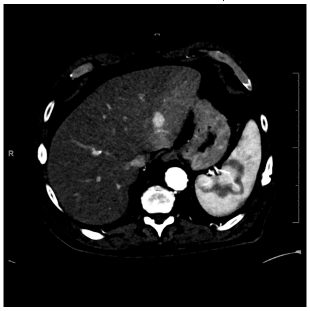

MRI February 28, 2024

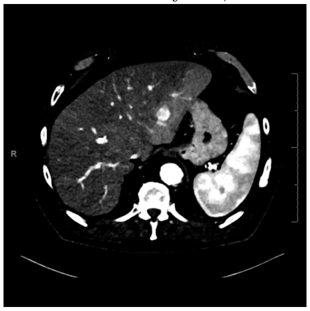

Model Response:
Based on the latest imaging and clinical data provided for Mr. Miller, his hepatocellular carcinoma (HCC) has shown a slight decrease in size, with a calculated change in tumor volume of approximately -0.96% between the MRI scans from December 3, 2023, and February 28, 2024 [Tool]. According to RECIST criteria, this does not qualify as disease progression, which requires at least a 20% increase in the sum of diameters of target lesions [3]. Therefore, Mr. Miller's disease is considered stable or slightly improved.
Given Mr. Miller's stable disease state, minimal symptoms, well-preserved liver function (Child-Pugh A5), and an ECOG performance status of 1, several treatment options could be considered [Patient]. His current management strategy of regular monitoring is appropriate given the stable nature of his disease. However, should there be signs of progression in the future, systemic therapies could be explored. These include:
1. **Targeted Therapies**: Sorafenib or lenvatinib, which are tyrosine kinase inhibitors, have been established as first-line treatments for advanced HCC in patients with well-preserved liver function and without significant portal hypertension [12], [15].
2. **Immunotherapy**: The combination of atezolizumab and bevacizumab has shown superiority over sorafenib in terms of overall survival and is recommended as a first-line treatment for patients with advanced HCC, Child-Pugh class A liver disease, and ECOG PS 0-1 [8], [29]. Mr. Miller's history of hypertension, managed with amlodipine, should be carefully considered due to the potential risk of bleeding associated with bevacizumab [29].
3. **Clinical Trials**: Participation in clinical trials could provide access to new therapies or combinations of therapies that are not yet widely available. This option should always be considered for patients with HCC, especially those with advanced disease [Patient].

No.	Patient Case Description
	4. **Continued Monitoring**: Given the stability of Mr. Miller's disease, continued regular monitoring with imaging and tumor markers could be considered to promptly identify any signs of progression [Patient].<br>5. **Local Ablative Therapies**: Although Mr. Miller has previously received RFA and TACE, should new lesions appear that are amenable to local treatments, options such as repeat RFA or TACE could be revisited [Patient].<br>In conclusion, Mr. Miller's HCC has not progressed according to RECIST criteria, and his disease appears to be stable with a slight decrease in tumor size. The choice of further treatment should be personalized, taking into account his preferences, potential benefits and risks of each treatment, and the goal of maintaining quality of life. A multidisciplinary team approach involving hepatologists, oncologists, and interventional radiologists is crucial for optimal decision-making [Strategy].<br>To enhance our understanding of Mr. Miller's hepatocellular carcinoma and tailor treatment options more precisely, could you supply histologic or histopathology images? These images are crucial for a detailed genetic analysis. Additionally, if there are any known genetic alterations within Mr. Miller's tumor, such as MSI, BRAF, or KRAS mutations, providing these specifics would enable a more targeted search for suitable treatment options. The exact mutation details, including the gene symbol (hugo_symbol), the nature of the change, and the type of alteration, are essential for this analysis. This information will allow us to leverage our resources effectively to propose the most appropriate therapeutic strategies.
P	Mrs Patel, Priya is a 52-year old female Indian Patient who presents to her recent follow up appointment after resection of locally advanced colorectal adenocarcinoma in late 2022. Here is the relevant patient information:<br>Initial Diagnosis:<br>Tumor Type: Colorectal adenocarcinoma<br>Stage: IIIb<br>Details: T3N1M0<br>Size: 5 cm<br>Mutations: EGFR T790M, PIK3CA mut, KRAS wt, BRAF wt<br>Previous Treatment:<br>Surgery: R0 - resection of the primary tumor on December 1, 2022 Pathology: R0, T3N1M0 L0 V0<br>Tumor board from December 8 2022, adjuvant chemotherapy was initiated<br>January 2023 - February 2023: Adjuvant 5-fu based chemotherapy.<br>March 2023: CT scan of the abdomen and pelvis showed no evidence of disease.<br>June 2023: Follow-up CT scan confirmed NED.<br>September 2023: Routine CT scan: NED.<br>December 2023: Continued surveillance with CT scan showing NED.<br>March 2024: Follow-up CT scan revealed NED.<br>The patient received a novel CT abdomen scan yesterday - we are currently awaiting the radiologist's report for further evaluation. Mrs Patel is in good condition, she does not report any currently relevant health issues.<br><br>**Doctor:** Could you please assess the current state of Mrs. Patel's disease based on the most recent imaging and clinical data? Could you develop a detailed treatment plan for Mrs. Patel's ongoing care and management. If necessary, also include local therapy options considering her current disease status. Would her mutational status allow for targeted therapy options?<br>**Appendix:**

No.	Patient Case Description

March 2024

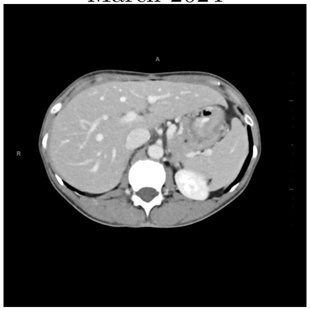

July 2024

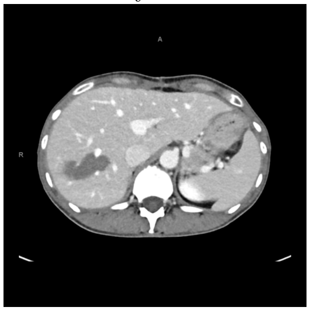

Model Response:
Based on the most recent imaging and clinical data, Mrs. Patel's disease has progressed from no evidence of disease (NED) to metastatic disease involving the liver and potentially the spleen, as evidenced by the July 2024 CT scan showing multiple hypodense lesions [Tool]. This progression necessitates a comprehensive reassessment of her treatment strategy to address the metastatic spread.
Treatment Plan for Mrs. Patel's Ongoing Care and Management:
1. **Systemic Therapy Adjustment:**
- **Targeted Therapy Consideration:** Given Mrs. Patel's EGFR T790M mutation, the use of osimertinib, a third-generation EGFR TKI, could be beneficial. Although primarily approved for non-small cell lung cancer, its efficacy in colorectal cancer harboring this mutation warrants discussion in a multidisciplinary team setting. A trial of osimertinib or consultation with a clinical trial offering targeted therapy for colorectal cancer patients with this mutation should be considered [14], [15], [17], [18].
- **Chemotherapy Regimen Reevaluation:** Based on the progression, a second-line chemotherapy regimen should be considered. Options may include FOLFIRI (irinotecan, fluorouracil, and leucovorin) if not previously used, potentially combined with a biologic agent like bevacizumab, depending on her VEGF status and previous exposure [3], [4], [5].
2. **Local Therapy Options:**
- **Liver-Directed Therapies:** For liver metastases, options such as radiofrequency ablation (RFA), microwave ablation (MWA), or stereotactic body radiotherapy (SBRT) could be considered, especially for lesions that are causing symptoms or are well-defined and accessible for such interventions [1], [2], [59], [60].
- **Splenectomy:** If the spleen's involvement is significant and symptomatic, and if systemic therapy controls the disease elsewhere, splenectomy could be discussed as a palliative measure to improve quality of life [Patient].
3. **Further Genetic Testing:**
- Due to the error in mutation checking and the unknown significance of the PIK3CA mutation, comprehensive genomic profiling should be performed to identify any additional actionable mutations that could open up further targeted therapy options [Tool].
4. **Supportive Care:**
- Ensure supportive care measures are in place, including pain management, nutritional support, and psychological support to address the physical and emotional aspects of metastatic cancer [Patient].
5. **Clinical Trial Enrollment:**
- Explore enrollment in clinical trials that are investigating new targeted therapies or combination therapies for metastatic colorectal cancer, especially those focusing on her specific mutational profile [Patient].
6. **Multidisciplinary Team Consultation:**

No.	Patient Case Description
	- A multidisciplinary team approach involving medical oncologists, surgical oncologists, radiologists, and genetic counselors is crucial to tailor the treatment plan to Mrs. Patel's evolving disease status and to explore all available therapeutic options [Patient].<br>In summary, Mrs. Patel's treatment plan should be adjusted to address the progression of her disease with a focus on systemic therapy adjustments, consideration of local therapy options for symptom management, further genetic testing to guide targeted therapy decisions, and supportive care measures. Regular follow-up and reassessment will be essential to monitor the effectiveness of the treatment plan and make adjustments as needed.<br>To enhance our understanding and tailor Mrs. Patel's treatment plan more precisely, could you please provide the histopathology images of the liver and spleen lesions? This will allow for a detailed mutation analysis, specifically targeting MSI, BRAF, and KRAS mutations, which are crucial for determining the most effective targeted therapy options. Additionally, the exact dimensions of the metastatic lesions observed in the most recent CT scans would be invaluable. This information will enable the quantification of these lesions' size, assisting in the evaluation of disease progression and the potential efficacy of local therapy options.
Zh	Mrs. Li Zhang, a 61-year-old Chinese immigrant to the USA, consults our oncology clinic for the first time today, in the company of her husband. She brings with her relevant medical reports and imaging studies for further evaluation.<br>Here is the patient report from her previous oncologist:<br>===<br>Dear Sir or Madam,<br>We report on Mrs. Li Zhang, who presents with metastatic colorectal adenocarcinoma. Below is a detailed timeline of her diagnosis and treatment history:<br>Initial Diagnosis:<br>Date: Early 2023<br>Tumor Type: Metastatic colorectal adenocarcinoma<br>Stage: III, T4N2M1 (Liver)<br>Tumor Size: Primary tumor 5.0 cm<br>Mutations: KRAS wild-type, BRAF wild-type<br>Treatment Timeline:<br>January 2023: Initiation of FOLFOX + Panitumumab<br>March 2023: CT Scan – Stable Disease (SD)<br>April-June 2023: FOLFOX and Panitumumab, 6 cycles<br>June 2023: CT Chest / Abdomen: Liver lesions stable, no new metastases<br>June-September 2023: FOLFOX (Oxaliplatin 80%) and Panitumumab, 6 cycles<br>September 2023: Progressive disease. Multiple small metastases to the lungs, one new liver metastasis, and growth of existing metastases.<br>September 2023: Change to FOLFIRI + Bevacizumab.<br>December 2023: Stable disease in CT Chest / Abdomen. No new metastases. Slight decrease in size of the liver metastases.<br>December 2023 - March 2024: Continuation of FOLFIRI + Bevacizumab.<br>February 2024: CT scan: Stable disease.<br>Planned: Continuation of chemotherapy with FOLFIRI and Bevacizumab on March, 6th 2024.<br>===<br>Here is her radiologists report from February this year:<br>===<br>CT Chest / Abdomen, February 2024:<br>Patient: Zhang, Li

No.	Patient Case Description
	Liver: The scan identifies a tumor in the liver, exemplary lesions here: [280, 400, 400, 550]. Overall, lesions appear stable compared to the imaging from December 2023. No new metastases.<br>Stomach: The stomach displays a normal appearance without any abnormal masses or lesions.<br>Spleen: The spleen is within normal limits, with no abnormalities in size or appearance.<br>Hepatic and Portal Veins: Both the hepatic and portal veins are patent, showing no evidence of thrombosis or other abnormalities. Aorta: The aorta is normal in caliber and contour, with no signs of aneurysm, dissection, or other pathology.<br>Ribs: The ribs are intact, without any fractures, lytic lesions, or other abnormalities.<br>Lungs: The lungs are clear, with no new pulmonary lesions and no signs of metastasis.<br>Vertebrae: The vertebral alignment and signal characteristics are normal, showing no evidence of metastatic involvement or pathological fractures.<br>===<br>She ultimately received another 4 cycles of chemotherapy plus bevacizumab after the CT scan from February. Next is the most recent radiology report that was performed externally:<br>===<br>CT Chest / Abdomen, July, 4, 2024:<br>Patient: Zhang, Li<br>The CT scan of the chest and abdomen reveals two large metastases in the liver located at coordinates [280, 380, 380, 490]. The stomach appears normal, with no evidence of abnormal masses or lesions. The spleen is within normal limits, showing no abnormalities in size or appearance. The hepatic and portal veins are patent, with no evidence of thrombosis or other abnormalities. The aorta appears normal in both caliber and contour, with no signs of aneurysm, dissection, or other pathology. The ribs are intact, with no evidence of fractures, lytic lesions, or other abnormalities. The lungs show no new pulmonary lesions and remain clear, with no signs of metastasis. Vertebral alignment and signal characteristics are normal, with no evidence of metastatic involvement or pathological fractures.<br>Note: The patient provided no previous imaging studies, making a direct comparison to assess disease development over time not possible.<br>===<br>Mrs Chen additionally reports that a new CT-guided liver biopsy was performed a few days ago, but the pathology report is still pending. Her images including CT scans (CT_February2024.png and CT_June2024.png) and the biopsy slides have been uploaded to the system.<br>Her system ID is: AA-02-xx2.<br><br>**Doctor:**<br>Please assess the current state of Mrs. Zhang's oncologic disease? Please measure if the described liver metastases have progressed or responded to therapy? Can you calculate in detail how much her tumor has changed? What treatment should we suggest to her according to current guidelines?

No.	Patient Case Description

Appendix:

CT February 2024

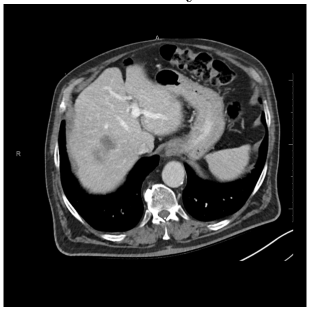

CT June 2024

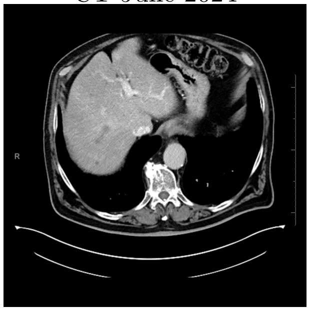

Histology

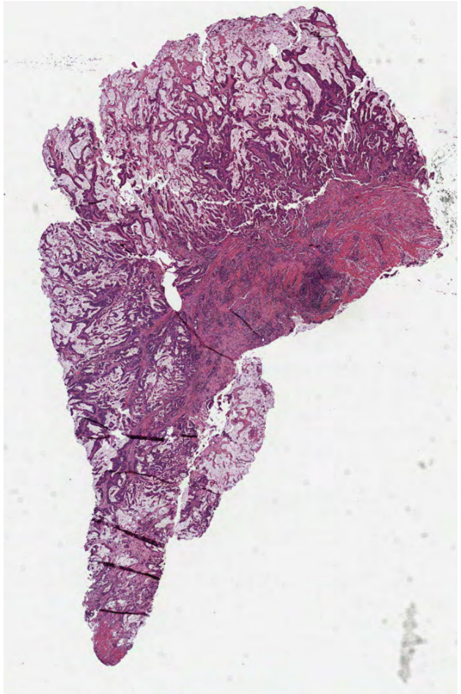

Model Response:

Based on the comprehensive analysis of Mrs. Zhang's medical history, radiological data, and genetic testing, we have a clear picture of her current oncologic status and can make informed recommendations for her ongoing treatment strategy.

Summary of Patient Details and Test Outcomes:

- Mrs. Zhang has metastatic colorectal adenocarcinoma, initially treated with FOLFOX + Panitumumab, showing stable disease until September 2023 when progression was noted, leading to a switch to FOLFIRI + Bevacizumab [Patient].
- The quantitative analysis of liver metastases from CT scans in February 2024 and June 2024 shows a significant decrease in the size of liver metastases from 5980 square units to 2181 square units, indicating a substantial response to the FOLFIRI + Bevacizumab treatment [Tool].
- Genetic analysis indicates MSI-High status, with no mutations in BRAF and KRAS, which is crucial for determining future treatment options [Tool].

Treatment Recommendations:

No.	Patient Case Description
	1. **Continuation of Current Regimen**: Given the significant response to the FOLFIRI + Bevacizumab regimen, it is advisable to continue this treatment. The substantial reduction in liver metastases size suggests effective disease control with this therapy [3][4][Tool].<br>2. **Consideration for Immunotherapy**: Mrs. Zhang's MSI-High status makes her a candidate for immunotherapy, which has shown effectiveness in MSI-High colorectal cancer cases. Pembrolizumab or nivolumab, with or without ipilimumab, could be considered as potential treatment options, especially if there is further progression on the current regimen [8][9][11][23][27][28][32].<br>3. **Genetic and Molecular Profiling**: The absence of BRAF and KRAS mutations, combined with MSI-High status, supports the consideration of immunotherapy. Continuous monitoring of her genetic profile may provide insights into resistance mechanisms or further tailor her treatment [9][11].<br>4. **Regular Monitoring and Reassessment**: Ongoing monitoring through imaging and tumor marker assessments will be crucial to adapt the treatment plan as needed. This should include regular CT scans and CEA levels, with adjustments to the treatment regimen based on disease progression or response [65][73][75].<br>5. **Multidisciplinary Team Management**: Given the complexity of Mrs. Zhang's case, management by a multidisciplinary team including medical oncologists, surgical oncologists, radiologists, and genetic counselors is recommended to ensure a comprehensive approach to her care [40][43][59][60].<br>6. **Patient and Family Education**: Discussing the potential benefits and side effects of the current treatment and possible future options, including immunotherapy, will be important. Mrs. Zhang and her family should be informed about the goals of therapy, expected outcomes, and signs of disease progression [110][111].<br>**Further Information Required**:<br>- **Pathology Report of Recent Biopsy**: The pathology report from the recent CT-guided liver biopsy will provide additional information that could influence treatment decisions, particularly if there are any changes in the tumor's molecular or genetic profile [Patient].<br>In conclusion, Mrs. Zhang's treatment should continue with FOLFIRI + Bevacizumab while preparing for a potential transition to immunotherapy, considering her MSI-High status. This approach aligns with current guidelines and her positive response to the current treatment, with a detailed plan for regular monitoring and reassessment to ensure the best possible outcomes.<br>To enhance our understanding and tailor Mrs. Zhang's treatment plan even more precisely, could you supply the directory path where her latest imaging studies are stored? This will enable a detailed analysis to monitor the progression or regression of her disease with high accuracy. Furthermore, if there are particular genetic alterations you suspect or are concerned about that haven't been explored yet, listing these would allow for a targeted search in relevant databases to uncover any potential treatment avenues or resistance mechanisms. Lastly, providing specific terms or questions you have regarding MSI-High colorectal cancer treatments could guide a focused literature review to ensure we're considering all viable treatment options based on the latest research findings.

No.	Patient Case Description

[1]Note that the Appendix is only for illustration purposes here. Any data shown is not directly provided to the agent, however is stored on the filesystem and can be accessed autonomously on demand through retrieving the necessary information where to find it from the main text.

[2]It should be noted that the outputs generated by the GPT-4 model are presented in their original format and structure, with only markdown elements such as bold text and subsection titles being translated into LaTeX, and adding color highlighting.

[3]Case courtesy of Guillermo Ropero Luis, *Radiopaedia.org*. From the case https://radiopaedia.org/cases/30460?lang=usrID: 30460

[4]We are aware that the actual images are MRI images, which is correctly identified by the model. Additionally, the model asks the user about the mentioned CT images.

[5]We acknowledge that the image in question depicts material from a primary tumor site, rather than from a metastatic liver site as proposed in this hypothetical scenario. This distinction has no bearing on the outcomes or conclusions of our study.

[6]This image is obtained from the following publication: Souguir, A. et al. CT-guided celiac plexus neurolysis in the management of pain related to unresectable pancreatic cancer: About 2 cases. Open J. Gastroenterol. 05, 37–41 (2015) under the Creative Commons Attribution 4.0 International License (CC BY 4.0).

Supplementary Note 2

No.	Tool Results
A	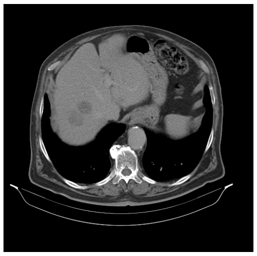 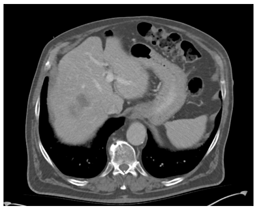 **1. Radiology Report: CTA_11_11_2023.png**: The initial imaging from November 11, 2023, showed a large area of low attenuation in the liver, which could suggest a liver lesion or cyst, and evidence of free fluid in the abdomen, indicating conditions such as ascites or acute pathology.<br>**CTA_02_24_2024.png**: The latest imaging from February 24, 2024, revealed several hypodense areas in the liver, highly suspicious for metastatic lesions, particularly in the right lobe. The liver parenchyma surrounding these lesions appeared normal. The spleen, pancreas, adrenal glands, and kidneys were unremarkable, with no signs of bowel obstruction or acute abdomen. The comparison between the two images suggested little to no change in the liver lesion's size and location but a reduction in the amount of free fluid in the abdomen, which could indicate a response to treatment or a change in the patient's condition. **2. Genetic Predictions:**<br>- **Microsatellite Instability (MSI):** The prediction is Microsatellite Stable (MSS) with a high probability of 0.97, indicating that the cancer is not likely to have high levels of MSI, which is associated with a better response to certain types of immunotherapy.<br>- **BRAF Mutation:** The prediction is wild-type with a very low probability of 0.03, suggesting that there is no BRAF mutation present.<br>- **KRAS Mutation:** The prediction is wild-type with a probability of 0.48, indicating that there is no KRAS mutation present. **Summary:** The radiology reports and genetic predictions provide a comprehensive overview of Mrs. Adams' current condition. The imaging studies show multiple liver lesions consistent with metastases and a reduction in abdominal free fluid, suggesting a potential response to the chemotherapy regimen. The genetic analysis indicates that the cancer is MSS and lacks mutations in BRAF and KRAS, which could influence the choice of targeted therapies. This information is crucial for planning the next steps in Mrs. Adams' cancer treatment, potentially guiding the selection of targeted therapies based on the genetic makeup of her cancer.

No.	Tool Results
B	

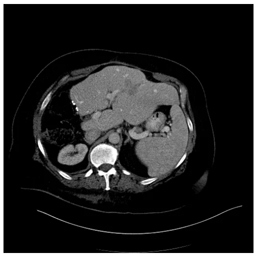 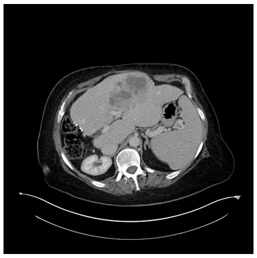

Based on the tools used and the information received, here is a detailed summary of the new information regarding Mrs. Brown's condition and potential implications for her oncologic treatment:

1. Radiology Report Analysis:
- Two CT images were analyzed: CTA_10_02_24.png and CTA_01_11_23.png.
- **CTA_10_02_24.png**: This image shows a cross-sectional view of the human abdomen, particularly focusing on the liver and kidneys. A notable difference in density within the liver suggests the presence of a lesion or abnormality. The kidneys and other anatomical structures are visible, with the right kidney partially obscured by the liver.
- **CTA_01_11_23.png**: Similar to the first image, this scan shows the liver, kidneys, spine, and abdominal wall. However, the liver appears to have a more uniform density without the prominent lesion or abnormality observed in the first image.
- **Comparison**: The most significant finding when comparing the two images is the appearance of a heterogeneous area within the liver in CTA_10_02_24.png, which is not present in CTA_01_11_23.png. This could indicate a pathological change, possibly the development of a new liver metastasis.

2. Genetic Predictions from Histopathology Images:
- **MSI (Microsatellite Instability)**: The prediction is MSS (Microsatellite Stable) with a high probability of 0.97. This suggests that the cancer is not characterized by high levels of microsatellite instability, which can influence the response to certain types of immunotherapy.
- **BRAF Mutation**: The prediction is wild-type with a very low probability of 0.03, indicating that a BRAF mutation is unlikely. This has implications for targeted therapy options, as BRAF mutations can sometimes be targeted with specific drugs.
- **KRAS Mutation**: The prediction is mutated with a probability of 0.68. This suggests the presence of a KRAS mutation, which is significant as it can impact the effectiveness of certain treatments, including EGFR inhibitors like Panitumumab.

Implications for Oncologic Treatment:
- The presence of a new lesion or abnormality in the liver, as indicated by the radiology report, suggests that the disease may have progressed. This needs to be considered when planning further treatment.

No.	Tool Results
	- The genetic analysis indicates that the cancer is MSS and KRAS mutated, with no BRAF mutation. This genetic profile can influence the choice of chemotherapy and targeted therapy options. Specifically, the presence of a KRAS mutation may limit the effectiveness of EGFR inhibitors like Panitumumab.<br>- Given these findings, a continuation of chemotherapy plus Panitumumab may need to be re-evaluated in light of the KRAS mutation. Other treatment options, possibly including other targeted therapies or immunotherapies, may need to be considered depending on the full clinical context and additional factors such as the patient's overall health, preferences, and other genetic markers not analyzed here.<br>This summary provides a comprehensive overview of the new information obtained from the tools used, highlighting key findings and their potential implications for Mrs. Brown's oncologic treatment.

No.	Tool Results
D	

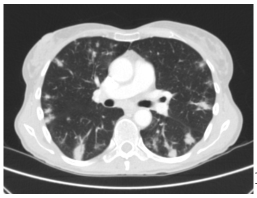[1] 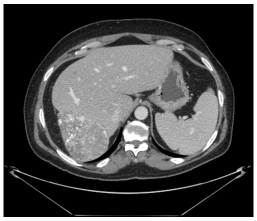

The strategy to determine the suitability of Mr. Doe for surgery or systemic therapy, and to explore options for targeted therapies, involved the use of two tools: **gen_radiology_report** and **check_mutations**. Here's a summary of the new information received from these tools and what they mean:

Radiology Report Analysis:
Two CT images, **CT_Lung.png** and **CT_Liver.png**, were analyzed to assess the extent of Mr. Doe's colorectal cancer.
CT_Lung.png findings:
- Multiple well-defined nodules scattered throughout both lung fields, suggestive of metastatic disease.
- No significant consolidation, pleural effusion, or lymphadenopathy.
- The nodules are hyperdense, non-calcified, and without cavitation.
- The report concludes that these findings raise concern for metastatic disease and recommends further evaluation with a tissue biopsy.
- **CT_Liver.png** findings:
- Multiple hypodense lesions in both lobes of the liver, consistent with hepatic metastases.
- The largest lesion in the right lobe has irregular margins, indicating an aggressive growth pattern.
- No signs of cirrhosis, fatty infiltration, thrombosis, or encasement of vascular structures.
- No evidence of ascites or visible lymphadenopathy in the provided slice.
- The report concludes that the findings are concerning for metastatic spread of colorectal cancer and recommends a full review of the entire CT scan series for a complete assessment.

Genetic Predictions from Histopathology Images The **check_mutations** tool provided predictions on three genetic targets: MSI, BRAF, and KRAS.
- **MSI (Microsatellite Instability)**: The prediction is MSI-High with a probability of 0.95. This indicates a high likelihood of the cancer being MSI-High, which is relevant for determining suitability for certain immunotherapies.
- **BRAF**: The prediction is wild-type with a probability of 0.48. This suggests that, contrary to Mr. Doe's recollection, the BRAF V600E mutation might not be present.
- **KRAS**: The prediction is mutated with a probability of 0.52. This indicates a slight likelihood of a KRAS mutation, which has implications for targeted therapy options.

Targeted Treatment Options for BRAF V600E
The **onko_kb** tool provided results on BRAF V600E.

No.	Tool Results
	- We received a comprehensive report on the BRAF V600E mutation, highlighting its oncogenic nature, known effect as a gain-of-function mutation, and its role in activating the downstream MAPK pathway independent of RAS. It also provided information on the sensitivity of this mutation to RAF monomer inhibitors such as vemurafenib and dabrafenib. The report detailed various treatments, including dabrafenib alone or in combination with trametinib, and their FDA-approved indications for different cancer types. Notably, it mentioned the combination of encorafenib and cetuximab as FDA-approved for the treatment of adult patients with metastatic colorectal cancer with a BRAF V600E mutation after prior therapy, based on the Phase III BEACON study.<br>**Web Search for BRAF V600E treatment options**<br>- The tool returned information on several drugs and treatment regimens available for colorectal cancer with the BRAF V600E mutation. These included Encorafenib in combination with Cetuximab, a targeted drug trio (Encorafenib, Cetuximab, and Binimetinib), Pembrolizumab for MSI mCRC harboring BRAFV600E mutation, and the feasibility of BRAF inhibitors in combination with MEK inhibitors to overcome drug resistance. It also mentioned ongoing trials and recent approvals for anti-BRAF/EGFR therapy in metastatic BRAFV600E colorectal cancer.<br>**Implications**<br>- The presence of metastatic disease in both the lungs and liver significantly impacts the treatment strategy, potentially making Mr. Doe less suitable for surgery and more reliant on systemic therapy.<br>- The genetic predictions suggest that Mr. Doe's cancer is MSI-High and has a KRAS mutation, which opens up possibilities for targeted therapies and immunotherapies, particularly given his refusal to undergo chemotherapy.<br>- The discrepancy regarding the BRAF mutation status highlights the importance of confirming genetic alterations through reliable molecular testing, as they directly influence the selection of targeted therapies.<br>This comprehensive analysis combining radiological and genetic data provides a clearer picture of Mr. Doe's disease status and the potential therapeutic avenues, emphasizing the need for a multidisciplinary approach to his treatment planning.

No.	Tool Results
G	

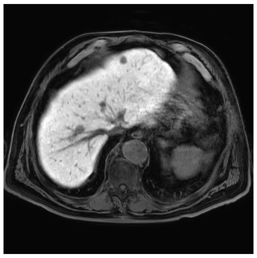

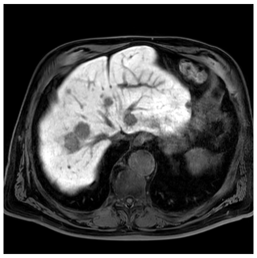

To develop a strategy for the best treatment approach for Mr. Garcia, who has metastatic colorectal cancer, the following steps were taken:

1. Segmentation of Hepatic Lesions in Imaging Data: Utilizing the ‘segment_image‘ function, the areas of hepatic lesions identified in the MRI scans were measured for both the initial scan taken on November 8, 2023 (’08_11_23.png’), and the recent control imaging conducted on February 10, 2024 (’02_10_24.png’). The bounding box coordinates for the lesions were provided based on the radiology reports.

- For the initial scan (’08_11_23.png’), the overall area of the regions of interest (three lesions) was calculated to be **1315** square units.
- For the recent control scan (’02_10_24.png’), the overall area of the regions of interest (four lesions) was calculated to be **2809** square units.

2. Calculation of Lesion Growth Ratio: Using the ‘calculate‘ function, the ratio of the lesion areas between the recent control imaging and the initial scan was determined to be approximately **2.14**. This indicates that the overall area of the hepatic lesions has more than doubled over the period between the two scans.

Summary of New Information Received:

- The segmentation of hepatic lesions from the MRI scans provided precise measurements of the lesion areas at two different time points.
- The calculation of the lesion growth ratio revealed a significant increase in the overall area of the hepatic lesions, suggesting progression of the metastatic disease.

This information is crucial for understanding the progression of Mr. Garcia’s metastatic colorectal cancer and will inform the next steps in developing a treatment strategy, including the consideration of additional diagnostic tests, genetic profiling, and potential treatment options.

Med-SAM Outputs:

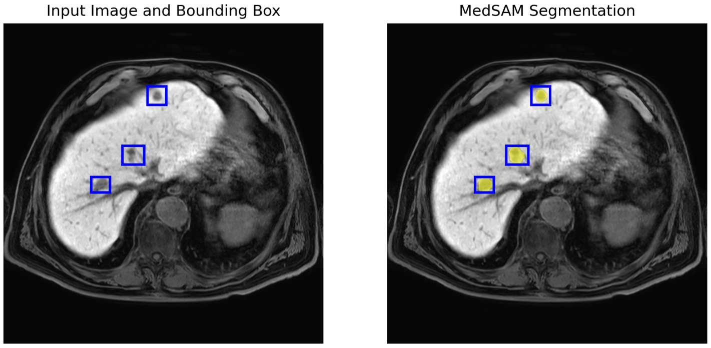

No.	Tool Results
	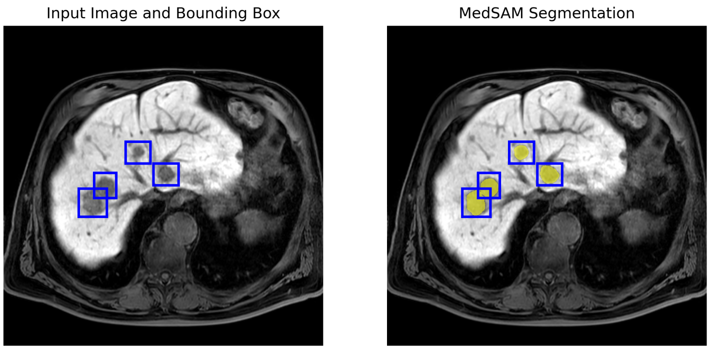

No.	Tool Results
Le	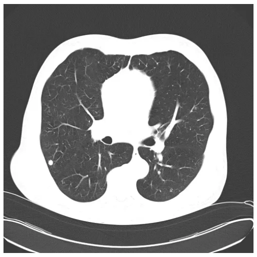 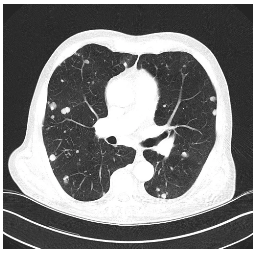 Based on the tools used and the information received, here is a detailed summary of the new information: **1. Radiology Report Analysis:** - Two CT scans were evaluated: 'CT_Lung_Feb_2024.png' and 'CT_Lung_Nov2023.png'. - The report for 'CT_Lung_Nov2023.png' described normal thoracic anatomy without obvious abnormalities in the lung fields, mediastinum, or bony structures. - The comparison between the two scans showed that the 'CT_Lung_Feb_2024.png' image had more and denser opacities throughout the lung fields compared to the 'CT_Lung_Nov2023.png' image, suggesting a more advanced or different pathological process in the February 2024 scan. - The findings indicate a progression in the lung nodules from November 2023 to February 2024, which could be consistent with a recurrence or progression of the pancreatic cancer. **2. Google Search on KRAS G12C Mutation Treatment in 2023: -** The search revealed significant advancements in the treatment of KRAS G12C mutation in cancer, with the development and approval of targeted therapies. - Key findings include: - **Sotorasib** has been compared to Docetaxel in patients with non-small-cell lung cancer (NSCLC) harboring the KRAS G12C mutation, showing promising results. - **Divarasib (GDC-6036)** reported durable clinical responses across KRAS G12C-positive tumors with mostly low-grade adverse events. - **Adagrasib**, alone or in combination with Cetuximab, evaluated in colorectal cancer patients with the KRAS G12C mutation, showing efficacy. - These studies and publications highlight the progress in the development of targeted therapies for cancers with the KRAS G12C mutation, offering new hope for patients with this genetic alteration. **Conclusion:** The radiology report comparison suggests a progression in Mr. Lee's lung nodules, which could be indicative of a recurrence or progression of his pancreatic cancer. The Google search on KRAS G12C mutation treatment in 2023 provided valuable insights into the latest advancements in targeted therapies for this mutation, including Sotorasib, Divarasib, and Adagrasib, which could be considered in the treatment strategy for Mr. Lee's condition.

No.	Tool Results
Lo	

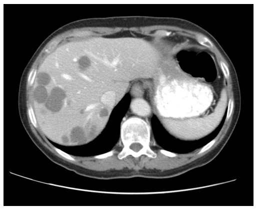

Based on the tools
used and the information received, we have gathered significant insights into Mr. Lopez's condition:
1. Radiology Report Analysis (CT Scan):
- **Tool Used**: 'functions.gen_radiology_report'
- **Input**: Path to the CT scan folder "Imaging/Lopez" with a query focusing on the extent of the primary tumor, lymph node involvement, and signs of metastasis, especially in the liver.
- **Output**: The CT scan (staging_CTA.png) showed multiple hypodense areas in the liver, which could indicate cysts, lesions, or areas of lower density compared to the normal liver parenchyma. Other organs such as the spleen, kidneys, stomach, spine, abdominal aorta, and bowel loops were visualized and appeared normal. The presence of multiple hypodense areas in the liver is of clinical significance and warrants further investigation.
- **Interpretation**: The findings suggest potential liver involvement, which could indicate metastasis. This is crucial for staging the cancer and determining the appropriate treatment strategy.
2. Genetic Mutation Analysis:
- **Tool Used**: 'functions.check_mutations'
- **Input**: Patient ID "AA-21-C" with targets for MSI, BRAF, and KRAS mutations.
- **Output**: The genetic analysis revealed that the patient is MSS (Microsatellite Stable) with a high probability of 0.97, indicating that the cancer is not MSI-high which often has implications for certain treatment options. Both BRAF and KRAS genes are wild-type with probabilities of 0.02 and 0.27, respectively.
- **Interpretation**: The absence of mutations in BRAF and KRAS genes suggests that treatments specifically targeting these mutations may not be applicable. The MSS status also influences the treatment approach, as MSI-high status can sometimes make patients eligible for immunotherapy.
Summary: The combination of the CT scan findings and genetic mutation analysis provides a comprehensive overview of Mr. Lopez's condition. The presence of multiple hypodense areas in the liver suggests potential metastasis, which is critical for staging the cancer and could influence the decision between local therapies and systemic treatments. The genetic analysis indicates that the cancer is MSS and lacks mutations in BRAF and KRAS, which guides us away from certain targeted therapies. This information is essential for developing a tailored treatment strategy for Mr. Lopez, considering both the extent of the disease and the molecular characteristics of the tumor. Further investigations and multidisciplinary discussions are necessary to finalize the treatment plan.

No.	Tool Results

S

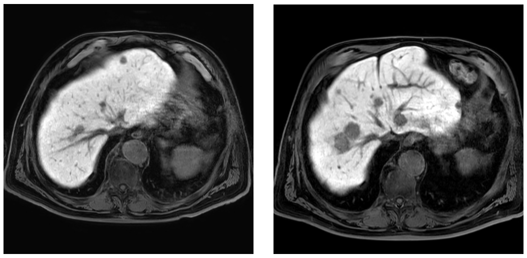

Based on the utilization of multiple tools, we have gathered new information regarding Mrs. Smith's current health status, particularly focusing on her liver metastases and genetic mutations.

1. Radiology Report Analysis:
- The comparison between two MRI scans, one from February 2024 (02_10_24.png) and the other from November 2023 (08_11_23.png), revealed the following:
- Both images show a similar cross-section of the human abdomen.
- A notable bright area on the left side of the images, indicative of a region of high signal intensity, was observed. This area was more pronounced in the February 2024 image compared to the November 2023 image.
- The contrast between different tissues was slightly more distinct in the November 2023 image.
- The decrease in intensity of the bright area from February 2024 to November 2023 might suggest a change in the liver metastases, but without further clinical context, a definitive assessment cannot be made.

2. Genetic Mutation Analysis:
- The genetic predictions from the histopathology images of patient B13-22A (Mrs. Smith) indicated:
- **KRAS Mutation**: Detected with a probability of 0.52. This suggests a mutation in the KRAS gene, which is a significant finding given that Mrs. Smith was previously reported as KRAS wild type in May 2023.
- **BRAF Status**: Confirmed as wild type with a high confidence (probability of 0.03), consistent with the initial genetic profiling.
- **MSI Status**: Predicted as MSS (Microsatellite Stable) with a high probability of 0.96, aligning with the initial diagnosis.

Summary:
The radiology report comparison suggests a potential change in the liver metastases, indicated by the variation in signal intensity between the two MRI scans. However, without additional clinical context, the significance of this change remains uncertain. The genetic mutation analysis revealed a new finding of a KRAS mutation, which differs from the initial genetic profiling that identified Mrs. Smith as KRAS wild type. The BRAF wild type and MSS status remain consistent with the initial diagnosis. This new information, particularly the KRAS mutation, could have implications for Mrs. Smith's treatment plan and may warrant a reevaluation of the current regimen of 5-Fluorouracil and Panitumumab.

No.	Tool Results
T	

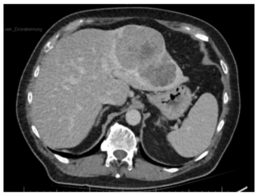

The radiology report generated from the CT scan image labeled "Staging_03_07_2024.png" for Mr. Taylor provides several key findings relevant to his current clinical status and the progression of his hepatocellular carcinoma (HCC) in the context of portal vein thrombosis (PVT) and underlying liver cirrhosis:

1. Liver Morphology and Lesions: The liver shows irregular contours and heterogeneous density, consistent with cirrhosis and previous interventions. Multiple hypodense areas within the liver parenchyma are observed, suggestive of multifocal HCC. The report emphasizes the need for comparison with previous imaging to assess for progression.

2. Portal Vein Thrombosis: A filling defect within the portal vein is noted, aligning with the known PVT. The report suggests further evaluation of the thrombosis's extent and its impact on portal circulation.

3. Vascular Structures: The aorta and inferior vena cava appear patent without obvious abnormalities.

4. Adjacent Organs and Ascites: The spleen is enlarged, likely secondary to portal hypertension. Ascites is also present, indicating a common complication of liver cirrhosis and portal hypertension.

5. Conclusion: The CT image shows features consistent with cirrhotic liver, multifocal HCC, portal vein thrombosis, ascites, and splenomegaly due to portal hypertension. The report recommends a comprehensive comparison with previous imaging to determine the progression of HCC and the status of portal vein thrombosis, as well as to identify any new findings that could influence the management of Mr. Taylor's disease.

This detailed radiology report provides crucial information for reassessing therapeutic strategies for Mr. Taylor, highlighting the importance of comparing current findings with previous imaging to understand the disease's progression and adjust the management plan accordingly.

No.	Tool Results
W	

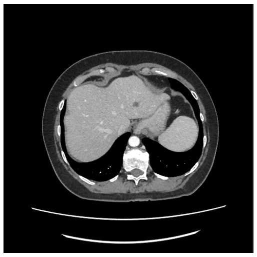
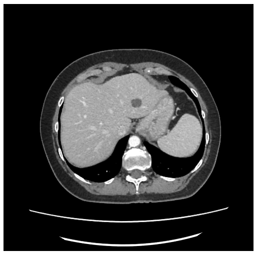

Here's a summary of the new information we received from the tools used: **1. Segmentation of Liver Metastasis:**
- The area of the liver metastasis in the November 2023 CT scan (Imaging/Williams/CTA_11_2023.png) was measured to be 204 square units.
- The area of the liver metastasis in the February 2024 CT scan (Imaging/Williams/CTA_02_2024.png) was measured to be 367 square units.
- The ratio between the February 2024 and November 2023 areas is approximately 1.799, indicating an increase in the size of the metastasis. **2. Genetic Predictions:**
- The genetic test revealed the tumor is MSI-High with a probability of 0.68.
- BRAF mutation was confirmed with a probability of 0.7.
- The tumor is KRAS wild-type with a probability of 0.44.

3. OncoKB Database Information:
- For the BRAF V600E mutation, it is described as oncogenic with a known effect of "Gain-of-function". It activates the downstream MAPK pathway independent of RAS and retains sensitivity to RAF monomer inhibitors such as vemurafenib and dabrafenib. There are several FDA-approved treatments for BRAF V600E mutations in different cancers, including dabrafenib and trametinib for melanoma, lung cancer, and others. Encorafenib and cetuximab are FDA-approved for metastatic colorectal cancer with a BRAF V600E mutation after prior therapy. **Interpretation:**
- The increase in the size of the liver metastasis under chemotherapy indicates progressive disease rather than a response to treatment.
- The described BRAF mutation was confirmed.
- The presence of a BRAF V600E mutation and MSI-High status suggests potential targeted therapy options. Specifically, the BRAF V600E mutation opens up several treatment avenues with FDA-approved drugs such as dabrafenib, trametinib, encorafenib, and cetuximab.
- The MSI-High status, although not yielding specific treatment options from OncoKB[2], is generally associated with a better response to immunotherapy in colorectal cancer.

Next Steps:
- Considering the progressive disease and the genetic makeup of the tumor, a discussion about switching to a targeted therapy regimen that includes BRAF inhibitors (e.g., dabrafenib or encorafenib) and possibly combining with an EGFR inhibitor (e.g., cetuximab) or a MEK inhibitor (e.g., trametinib) could be warranted.
- Further exploration of immunotherapy options, given the MSI-High status, could also be considered.

Med-SAM Outputs:

No.	Tool Results

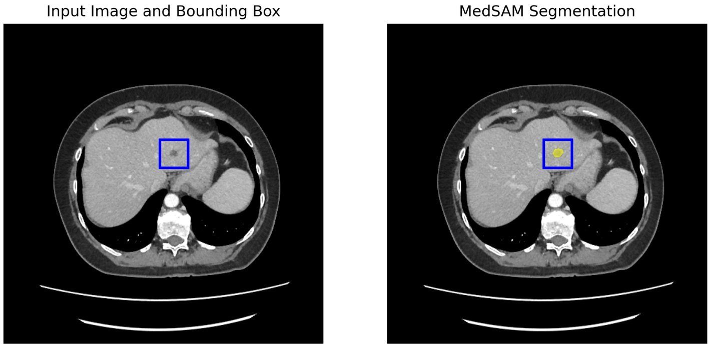

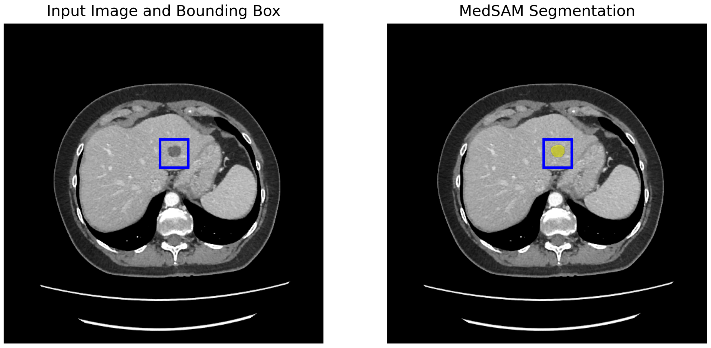

No.	Tool Results
X	

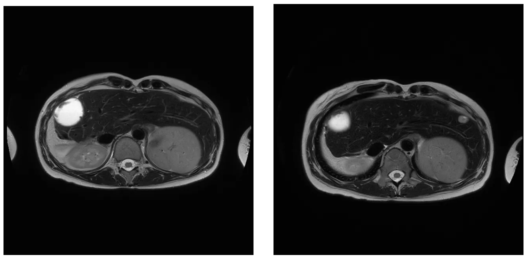

The strategy to investigate the current state of Ms. Xing's disease and explore targeted treatment options involved multiple steps, utilizing various tools to gather comprehensive information. Here's a summary of the new information received from the tools used, along with their implications: **1. Segmentation of Metastasis in Imaging:**
- **Tool Used**: 'segment_image' for both September 2023 and February 2024 images.
- **Inputs**: September 2023 image with coordinates [475, 250, 490, 275] and February 2024 image with coordinates [455, 270, 505, 320].
- **Outputs**: The area of the metastasis in September 2023 was 133, and in February 2024, it was 518.
- **Implication**: The significant increase in the area of the metastasis (approximately 3.89 times larger) indicates disease progression.

2. Investigation of Targeted Treatment Options for BRAF V600E Mutation:
- **Tool Used**: 'onco_kb'.
- **Inputs**: Query for BRAF V600E mutation.
- **Outputs**: Identified treatments including Dabrafenib and Trametinib, with FDA approval for BRAF V600E mutant unresectable or metastatic melanoma, and other solid tumors excluding colorectal cancer. Also noted was the effectiveness of these drugs in biliary tract cancer.
- **Implication**: Given Ms. Xing's BRAF V600E mutation, Dabrafenib and Trametinib could be considered for her treatment, noting their approval and effectiveness in similar cases.

3. Investigation of Targeted Treatment Options for CD74-ROS1 Variant Fusion:
- **Tool Used**: 'onco_kb'.
- **Inputs**: Query for CD74-ROS1 variant fusion.
- **Outputs**: Identified treatments for ROS1-positive tumors, including Crizotinib, Entrectinib, and Repotrectinib, with FDA approval for metastatic non-small cell lung cancer (NSCLC) whose tumors are ROS1-positive. Ceritinib and Lorlatinib were also mentioned as treatment options.
- **Implication**: Although the CD74-ROS1 fusion is primarily associated with NSCLC, the identified treatments could be explored for their potential applicability in Ms. Xing's case, given the oncogenic nature of this fusion.

4. Google Search for Targeted Treatment Options:
- **Tool Used**: 'google_search'
- **Inputs**: Query for targeted treatment options for cholangiocellular cancer with BRAF V600E mutation and CD74-ROS1 fusion
- **Output**: The Google search provided insights into potential targeted treatment options for cholangiocellular cancer with BRAF V600E mutation and CD74-ROS1 fusion. It highlighted the use of encorafenib and binimetinib, dabrafenib, and the combination of dabrafenib and trametinib as potential options based on their effectiveness in other cancers with similar genetic alterations.

No.	Tool Results

5. PubMed Query for Latest Targeted Treatment Options:
- **Tool Used: ‘query_pubmed‘**
- **Inputs:** Query for ’targeted treatment options for cholangiocellular cancer with BRAF V600E mutation and CD74-ROS1 fusion?’
- **Outputs:** The PubMed search did not yield specific results for targeted treatment options for cholangiocellular cancer with BRAF V600E mutation and CD74-ROS1 fusion. However, it emphasized the importance of targeting specific gene fusions in cancer treatment and mentioned the potential of NTRK gene fusions as novel targets, suggesting the relevance of molecular assays in identifying specific gene fusions for targeted therapy.

6. Genetic Predictions from Histopathology Images:
- **Tool Used**: ‘check_mutations‘
- **Inputs**: Patient ID ”X-09.22” and targets for MSI, KRAS, BRAF.
- **Microsatellite Instability (MSI)**: The prediction indicated Microsatellite Stability (MSS) with a high probability of 0.89, suggesting that the tumor is not characterized by a high level of microsatellite instability.
- **BRAF Mutation**: The analysis predicted a BRAF mutation with a probability of 0.74. This is consistent with the molecular report summary provided, which identified a mutation in BRAF V600E.
- **KRAS Mutation**: The prediction indicated a wild-type KRAS with a low probability of 0.29, suggesting that no KRAS mutation is present in the tumor.

This comprehensive approach, utilizing segmentation to quantify disease progression and querying the OncoKB database for targeted treatment options, provides a solid foundation for discussing Ms. Xing’s treatment in the molecular tumor board. The significant growth of the metastasis underscores the urgency of considering advanced targeted therapies, specifically those targeting the BRAF V600E mutation and CD74-ROS1 variant fusion, to potentially manage the disease progression as also shown by the provided web search results. The genetic predictions confirmed the presence of a BRAF mutation and showed the absence of a KRAS mutation and MSS status, which is contradictory to the described finding of microsatellite instability.
Med-SAM Outputs:

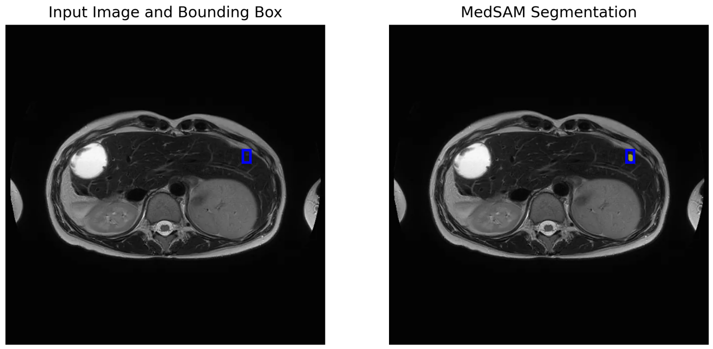

No.	Tool Results
	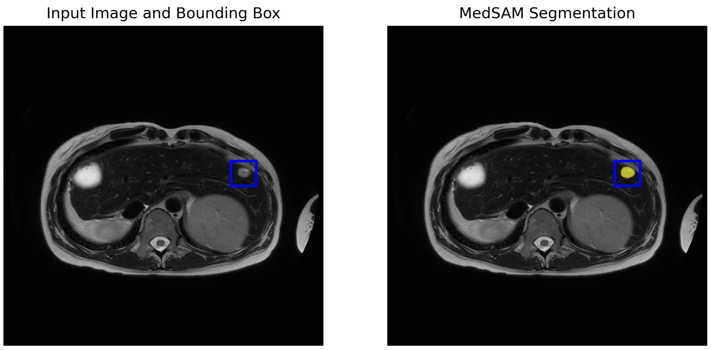

No. **Tool Results**

Z

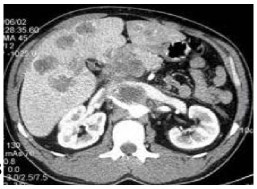
3

Based on the tools utilized and the information received, here is a detailed summary of the new information:

1. Radiology Report Generation:

- **Input**: Requested a detailed radiology report for Mr. Zu, a 55-year-old patient with histologically confirmed pancreatic adenocarcinoma (T2N1MX), who underwent an iv-contrast enhanced CT-scan of the abdomen for staging. The query specifically asked for the size of the primary tumor, presence of any metastases, and condition of surrounding organs.
- **Output**: Radiology Report for staging_Feb_24.png: The image provided appears to be a CT scan of the abdominal region of a patient. The scan is an axial slice which shows various organs including the liver, kidneys, spleen, and portions of the gastrointestinal tract.
- The liver has multiple hypodense areas which could indicate the presence of lesions or cysts. The exact nature of these would require clinical correlation and possibly further imaging or biopsy.
- The kidneys are visible on both sides of the spine and seem to be of normal size and shape. There does not appear to be any obvious abnormality with the kidneys from this single slice.
- The spleen is visible on the left side of the image (which corresponds to the patient's left side) and appears to be of normal size and density.
- There are no obvious signs of free air or fluid in the abdominal cavity that would suggest perforation or acute abdominal pathology.
- The aorta and other vascular structures appear to be normal without evidence of aneurysm or dissection in this slice.
- Bowel loops are visible and appear to be normal in caliber without evidence of obstruction or significant wall thickening.
- The vertebral body and spinal canal appear normal without evidence of fracture or pathology.
- **Conclusion**: The most significant finding in this CT scan is the presence of multiple hypodense areas within the liver. These findings warrant further investigation to determine their nature. The rest of the visible abdominal organs and structures appear to be within normal limits based on this single image. However, it is important to note that a full evaluation would require a review of the entire CT scan series, as well as clinical information and laboratory results for accurate diagnosis and staging.

2. Mutation Check:

- **Input**: Requested a check for specific genetic mutations (MSI, BRAF, KRAS) for patient Mr. Zu.
- **Output**: The tool encountered an error and did not provide any results for the mutation check.

No.	Tool Results
	**Summary:** The radiology report suggests the presence of multiple lesionsin the liver, which could potentially indicate metastases, given the patient's diagnosis of pancreatic adenocarcinoma. However, the report also highlights the limitation of making a definitive diagnosis based on a single CT slice and recommends a full review of the entire CT scan series for a comprehensive assessment. Unfortunately, the attempt to check for specific genetic mutations encountered an error, and no information regarding the genetic profile of the tumor was obtained. This information would have been valuable for determining targeted therapy options.

No.	Tool Results
Ad	

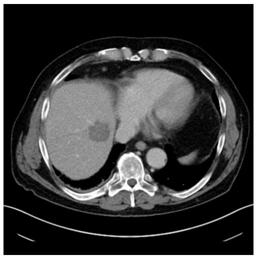

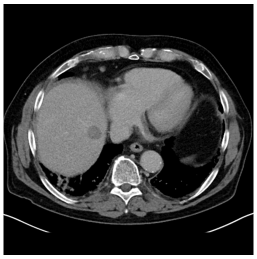

Based on the utilization of multiple tools and their outputs, here is a detailed summary of the new information we received about Mrs. Abena Adomako's condition:

1. **Segmentation of Metastasis in Liver Images**: - The segmentation of the liver metastasis from the CT scan dated November 15, 2022, resulted in an area of **6624** square units. - The segmentation of the liver metastasis from the CT scan dated June 10, 2024, showed an area of **2936** square units. - By calculating the ratio between the two areas, we found that the metastasis area decreased to approximately **2.26** times smaller in the 2024 scan compared to the 2022 scan. This indicates a significant reduction in the size of the metastasis, suggesting a positive response to the treatment.

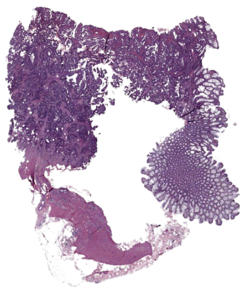

2. **Genetic Predictions from Histopathology Images**: - **MSI Status**: The patient is predicted to have Microsatellite Stability (MSS) with a high probability of **0.97**. This suggests that the cancer is less likely to respond to certain immunotherapies that target MSI-high tumors. - **BRAF Mutation**: The patient is predicted to have a wild-type BRAF gene with a low probability of **0.05** for having a BRAF mutation. This indicates that treatments targeting BRAF mutations may not be applicable. - **KRAS Mutation**: The patient is predicted to have a mutated KRAS gene with a probability of **0.68**. This suggests the presence of a KRAS mutation, which is important for determining the patient's eligibility for certain targeted therapies.

Given this information: - The significant reduction in the size of the liver metastasis suggests that the disease has responded well to the previous treatment regimen. - The genetic profiling indicates that while the patient may not benefit from treatments targeting MSI-high or BRAF mutations, the presence of a KRAS mutation opens the possibility for targeted therapies addressing this specific genetic alteration. - A detailed treatment plan for Mrs. Adomako's ongoing care and management should consider these findings, focusing on therapies that are effective against KRAS-mutated colorectal cancer, while also taking into account the positive response indicated by the reduction in metastasis size.

Med-SAM Outputs:

No.	Tool Results

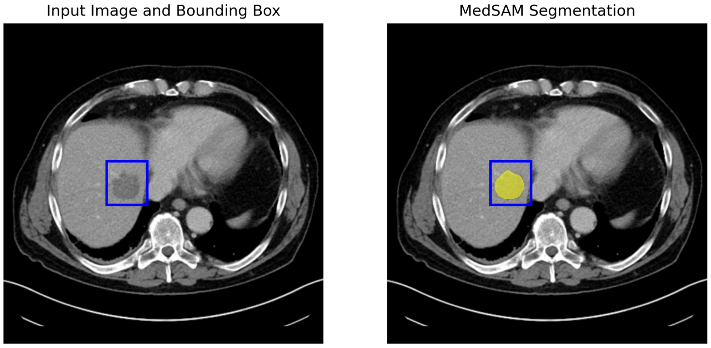

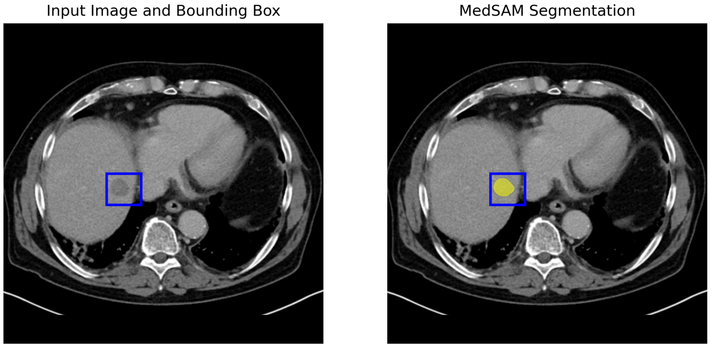

No.	Tool Results

An

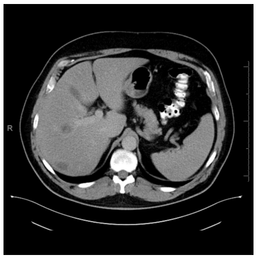
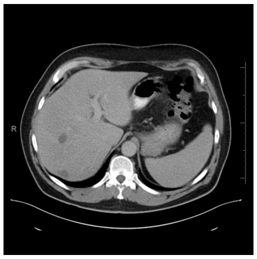

The strategy to evaluate the current state of Mrs. Anderson's disease and recommend treatment options involved using two tools: generating a radiology report from the most recent imaging and checking for genetic mutations.

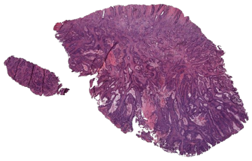

Radiology Report Summary: - **Imaging Date**: Last week - **Findings**: - **Liver Metastases**: Multiple hypodense lesions consistent with metastatic deposits. The largest lesion, located in the right lobe, measures approximately 5 cm in diameter. No new metastases outside the liver were detected. - **Liver Size and Contour**: Normal, with no significant hepatomegaly or intrahepatic biliary dilation. - **Other Organs**: The spleen, kidneys, pancreas, and adrenal glands are normal. No bowel obstructions or significant wall thickening. No abnormalities in the gastrointestinal tract. - **Vascular Structures**: Normal aorta and major branches. - **Lymph Nodes**: No significant lymphadenopathy. - **Peritoneal Cavity**: No ascites. - **Bone Structures**: No visible lesions or abnormalities. - **Conclusion**: The CT scan reveals multiple liver metastases with no new metastases outside the liver. The surrounding organs are stable with no significant abnormalities. The absence of ascites and lymphadenopathy is noted.

Genetic Mutation Analysis: - **MSI**: MSS (Microsatellite Stable) with a probability of 0.97. - **BRAF**: Wild-type with a probability of 0.03. - **KRAS**: Wild-type with a probability of 0.48.

Interpretation: - The radiology report indicates a significant burden of metastatic disease within the liver, consistent with Mrs. Anderson's known metastatic colorectal cancer. The largest metastasis is 5 cm in diameter, and there are no new metastases or significant abnormalities in the surrounding organs. - The genetic mutation analysis shows that Mrs. Anderson's cancer is MSS, and both BRAF and KRAS are wild-type. This genetic profile suggests that the cancer is not driven by mutations in these genes, which could influence treatment options.

This information provides a comprehensive overview of Mrs. Anderson's current disease state, including the extent of liver metastases and her genetic mutation status. This will be crucial for determining the next steps in her treatment plan.

No.	Tool Results
Ch	

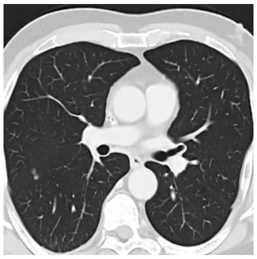

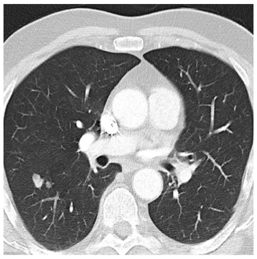

The following steps were taken to assess the current state of Mrs. Chen's disease and to develop a detailed treatment plan: 1. **Segmentation of Lesions in Imaging**: **- February 3, 2024 CT Scan**: The segmentation tool was applied to the image 'Imaging/Chen/February_3_2024.png' with bounding box coordinates [100,650,150,750]. The output indicated that the overall area of the regions of interest is **702 square units**. **- June 25, 2024 CT Scan**: The segmentation tool was used on the image 'Imaging/Chen/June_25_2024.png' with bounding box coordinates [100,650,220,780]. The result showed that the overall area of the regions of interest is **2798 square units**.

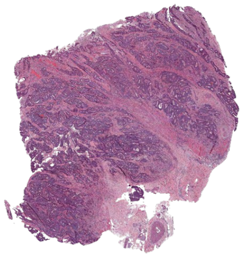

The significant increase in the area of the lesions from 702 to 2798 square units suggests a progression in the size of the metastases within the lungs over the period from February to June 2024. 2. **Genetic Mutation Analysis**: - The check_mutations tool was used to analyze Mrs. Chen's histopathology images for MSI, BRAF, and KRAS mutations using her patient ID "XX-03AY". The results were as follows: - **MSI**: The prediction is MSS (Microsatellite Stable) with a high probability of 0.97, indicating that the cancer is not MSI-high which often has implications for certain immunotherapy treatments. - **BRAF**: The prediction is wild-type with a probability of 0.03, suggesting that there is no BRAF mutation present. - **KRAS**: The prediction is wild-type with a probability of 0.27, indicating the absence of KRAS mutations.

These findings provide a comprehensive overview of Mrs. Chen's current disease state. The increase in lesion size despite ongoing systemic therapy suggests that the disease is progressing. The genetic analysis showing MSS status and the absence of BRAF and KRAS mutations could influence the choice of further treatment options, potentially ruling out therapies specifically targeting these mutations. This information is crucial for developing a personalized and effective treatment plan for Mrs. Chen's ongoing care and management.

Med-SAM Outputs:

No.	Tool Results

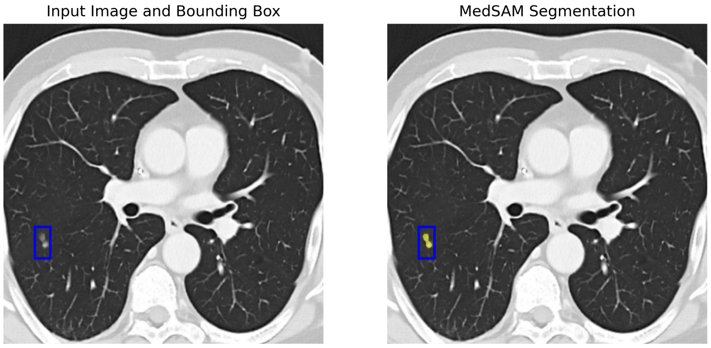

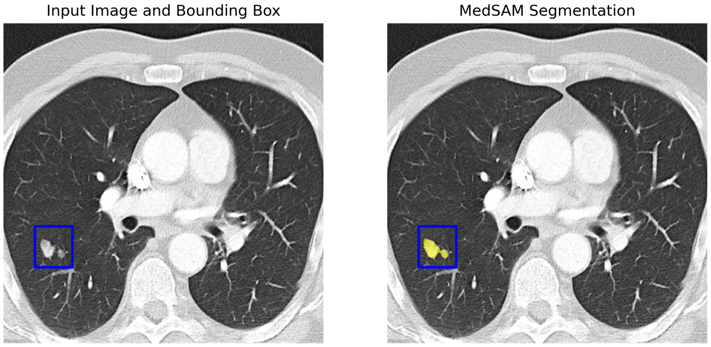

No.	Tool Results
Da	

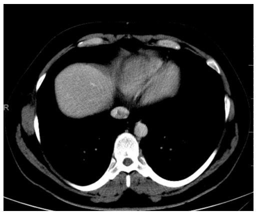
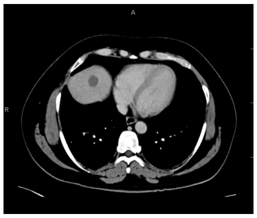

The information obtained from the tools provides a comprehensive update on Mr. Davis's condition, focusing on the recent imaging studies and genetic mutation status. **Radiology Report Analysis**: - **February 22, 2024, CT Abdomen Scan**: The report indicates a significant metastatic lesion in the liver, involving both lobes with a considerable portion of the right lobe affected. This finding is consistent with Mr. Davis's known history of metastatic colorectal cancer. The lesion's size and involvement suggest disease progression since the last scan in November 2023. Other abdominal organs, including the spleen, pancreas, and adrenal glands, appear unremarkable, with no new metastatic disease observed. Vascular structures are normal, and there are no signs of acute complications such as bleeding or significant ascites. - **Comparison with November 11, 2023, CT Scan**: The comparison highlights that both scans are axial CTs with similar resolution and anatomical visibility. However, the February 2024 scan shows a significant metastatic lesion in the liver, indicating disease progression. The comparison notes subtle differences in image quality and anatomical details but emphasizes the importance of correlating these images with clinical findings for accurate diagnostic conclusions.

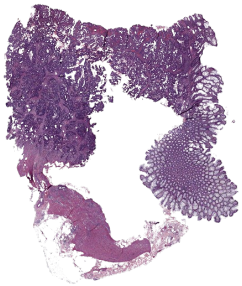

Genetic Mutation Analysis: -**Microsatellite Instability (MSI)**: The patient is microsatellite stable (MSS) with a high probability (0.97), indicating that the cancer is not characterized by a high level of MSI, which is often associated with better responses to certain immunotherapies. -**BRAF Mutation**: The patient is wild-type for BRAF with a very low probability (0.05) of mutation, suggesting that BRAF-targeted therapies are not applicable. -**KRAS Mutation**: The patient has a KRAS mutation with a moderate probability (0.68), which is significant for colorectal cancer as it may influence the response to certain targeted therapies and is an important factor in determining treatment strategy.

Summary: The recent imaging study from February 2024 shows disease progression in the liver, with a significant metastatic lesion noted. This progression, combined with the genetic analysis revealing a KRAS mutation, suggests that Mr. Davis's current treatment regimen may need to be reassessed. The stability of other abdominal organs and the absence of acute complications are positive aspects, but the overall findings indicate a need for a potential change in the treatment approach, considering the KRAS mutation's implications for targeted therapy options.

No.	Tool Results
J	

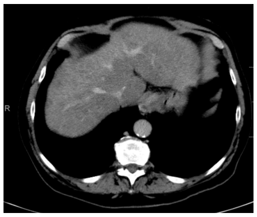

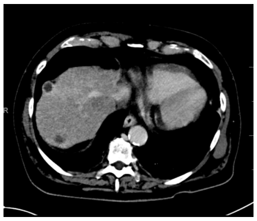

The analysis of Mrs. Johnson's imaging studies, specifically comparing the CT scans from December 2023 and May 2024, provides critical insights into her condition's progression and current status. Here's a detailed summary of the new information received from the radiology report tool and its implications:

Radiology Report Tool Usage: - **Input:** The tool was provided with the patient's medical history and asked to analyze the latest imaging study conducted in March 2024, available in the folder "Imaging/Johnson."
-**Output:** The tool generated detailed reports for two CT scans, one from December 2023 and another from May 2024, along with a comparative analysis.

Findings from December 2023 CT Scan: - **Liver Size and Morphology:** Enlarged liver with heterogeneous texture, consistent with cirrhosis and previous interventions. - **Lesions and Masses:** Hypodense areas indicative of necrosis or treatment effects, with no new significant lesions suggesting stable disease. - **Vascular and Biliary Structures:** Normal appearance without signs of thrombosis or obstruction. - **Adjacent Organs and Ascites:** Normal spleen size, no significant ascites, and unremarkable appearance of other abdominal organs. - **Conclusion:** Stable disease, aligning with the decision for a therapy break.

Findings from May 2024 CT Scan: - **Liver Size and Morphology:** Hepatomegaly with an irregular contour, indicative of progression or poor response to treatment. - **Lesions:** A large, heterogeneous mass in the right lobe with irregular margins, suggesting aggressive tumor behavior or disease progression. - **Vascular Structures:** Patent veins but with distortion and displacement due to the mass effect. - **Ascites and Lymph Nodes:** No significant ascites or enlarged lymph nodes. - **Conclusion:** Possible disease progression with a significant hepatic lesion, necessitating further diagnostic workup and treatment reassessment.

Comparative Analysis: - **Liver Size and Appearance:** Increase in liver size and heterogeneity from December 2023 to May 2024, suggesting pathological changes. - **Image Quality and Resolution:** Comparable between the two scans, ensuring consistency in observations. - **Overall Abdominal Configuration:** More distension in May 2024, potentially related to liver size increase or other factors.

Implications: The comparative analysis indicates a concerning shift in Mrs. Johnson's condition, with potential disease progression highlighted by the increase in liver size and the presence of a significant hepatic lesion with aggressive characteristics. The absence of significant ascites and normal spleen size are favorable signs, but the dominant concern is the hepatic lesion observed in May 2024. This suggests that the stability noted in December 2023 has been compromised, warranting a reassessment of the treatment strategy and consideration of additional therapeutic options.

The detailed radiology report underscores the need for a multidisciplinary approach to address the possible progression of hepatocellular carcinoma in Mrs. Johnson, taking into account her overall clinical condition, liver function, and performance status. Further diagnostic workup, potentially including biopsy and molecular profiling, may be necessary to tailor the next steps in her treatment plan.

No.	Tool Results
Ma	

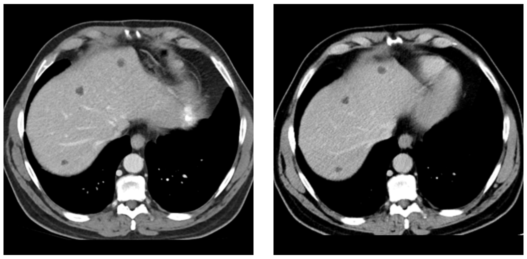

To assess the current state of Mr. Martinez's disease and develop a detailed treatment plan, including the exploration of targeted therapies, the following steps were taken using various tools:
1. **Segmentation of Liver Metastases from Imaging**: - **February 2024 Imaging Analysis**: The segmentation of liver metastases from the image 'February2024.png' resulted in an overall area of **3519** square units for the regions of interest. - **May 2024 Imaging Analysis**: The segmentation of liver metastases from the image 'May2024.png' showed an overall area of **3841** square units for the regions of interest. - **Interpretation**: Comparing these two results indicates an increase in the size of liver metastases from February to May 2024, suggesting disease progression despite ongoing chemotherapy.
2. **OncoKB Analysis for KRAS G12D Mutation**: - The KRAS G12D mutation is identified as **oncogenic** with a **gain-of-function** effect. It is associated with resistance to BRAF inhibition but sensitivity to MEK inhibitors like cobimetinib. This mutation is prevalent in various cancers, including lung, colorectal, pancreatic, and ovarian cancers. The analysis highlighted several treatment options: - **Cetuximab and Panitumumab**: Not favorable for patients with KRAS mutations, including G12D, in metastatic colorectal cancer (mCRC). - **Tucatinib and Trastuzumab**: Approved for HER2 amplified mCRC with RAS and BRAF wildtype, indicating no favorable response for KRAS-mutated cancers. - **Cobimetinib**: A MEK inhibitor showing effectiveness in various studies, including those with KRAS mutations. - **Trametinib**: Another MEK inhibitor with evidence of effectiveness in KRAS-mutated cancers. - **RMC-6236**: A small-molecule inhibitor of RAS, showing promising results in clinical studies for KRAS G12X-mutant cancers, including G12D. - **MRTX-1133**: An orally available inhibitor of KRAS G12D, demonstrating selective sensitivity and significant antitumor activity in preclinical studies. - **ASP3082**: A KRAS G12D degrader showing effectiveness in preclinical studies with KRAS G12D-mutated pancreatic cancer cells.
Summary: The analysis of liver metastases size increase and the detailed OncoKB report on the KRAS G12D mutation provide a comprehensive view of Mr. Martinez's disease progression and potential targeted treatment options. The growth in metastases size indicates that the current chemotherapy regimen may not be effectively controlling the disease. The OncoKB analysis suggests that while traditional EGFR-targeted therapies like cetuximab and panitumumab are not favorable due to the KRAS mutation, MEK inhibitors (cobimetinib, trametinib) and novel agents targeting KRAS (RMC-6236, MRTX-1133, ASP3082) could be potential options for future treatment, subject to further clinical evaluation and consideration of Mr. Martinez's overall health status and treatment history.

No.	Tool Results

Med-SAM Outputs:

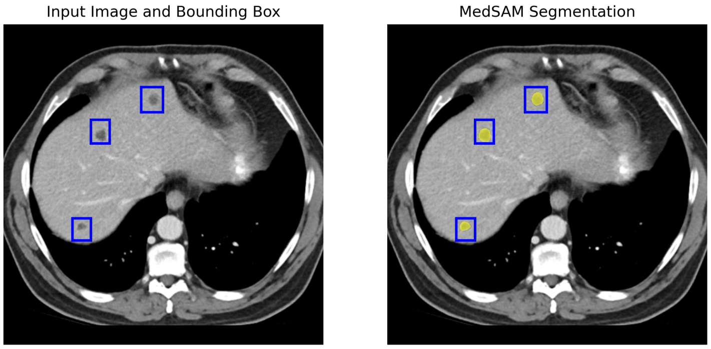

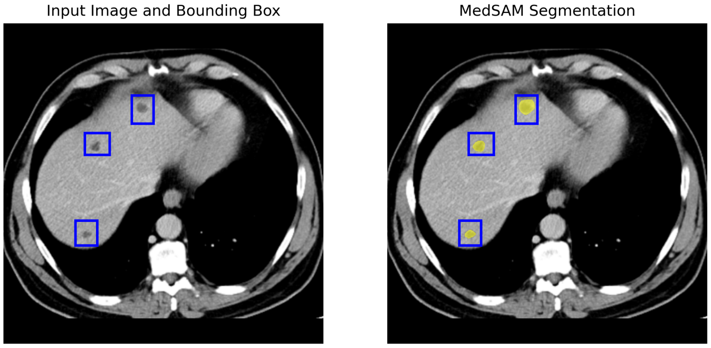

No.	Tool Results
Mi	

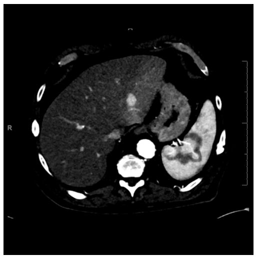
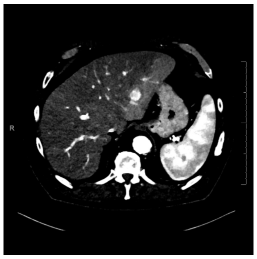

To evaluate the current state of Mr. Miller's disease and assess its progression according to RECIST criteria, the following steps were taken using the available tools:

1. **Segmentation of MRI Images**: The MRI images from December 3, 2023, and February 28, 2024, were analyzed to determine the size of the hepatocellular carcinoma (HCC) lesion. The segmentation tool provided the sizes of the regions of interest as follows: - December 3, 2023, MRI: The area was 2077 square units. - February 28, 2024, MRI: The area was 2057 square units.
2. **Calculation of Tumor Volume Change**: The change in the tumor's size was calculated to determine if the disease had progressed according to RECIST criteria. The calculations revealed: - The difference in tumor size between the two dates was -20 square units, indicating a slight decrease in size. - The percentage change in tumor size was calculated as approximately 0.96
3. **Treatment Options and RECIST Criteria**: A search was conducted to gather information on the treatment options for hepatocellular carcinoma and to understand the RECIST criteria for assessing disease progression. The search provided comprehensive details on various treatment modalities, including surgical treatments, local ablative therapies, transarterial therapies, systemic therapies, radiation therapy, and supportive care. It also explained the RECIST criteria, which are used to evaluate the response of solid tumors to treatment, including definitions for complete response, partial response, stable disease, and progressive disease.

Summary of Findings:
- The slight decrease in tumor size (-0.96%) suggests that the disease has not progressed according to RECIST criteria, which define progressive disease as at least a 20% increase in the sum of diameters of target lesions. Therefore, Mr. Miller's disease appears to be stable or slightly improved based on the imaging data. - Given Mr. Miller's current disease state, treatment options could include continuing monitoring and potentially considering systemic therapies such as targeted therapies or immunotherapy, especially if the disease shows signs of progression in the future. The choice of treatment would depend on further assessment of his liver function, overall health status, and specific characteristics of the tumor.

This analysis provides a detailed overview of Mr. Miller's disease status based on the latest imaging and clinical data, indicating a stable disease state with minimal change in tumor size. The information on treatment options and RECIST criteria will be valuable for guiding future management decisions.

Med-SAM Outputs:

No.	Tool Results
	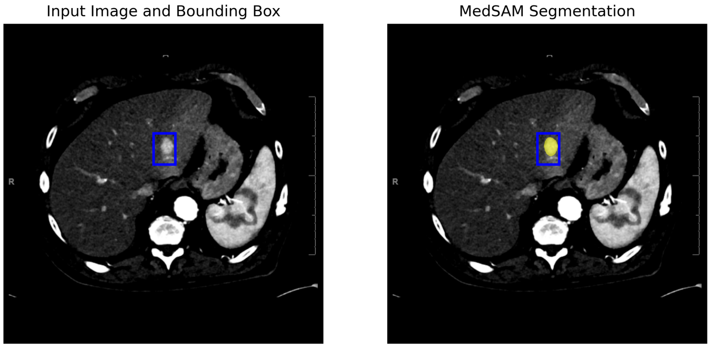 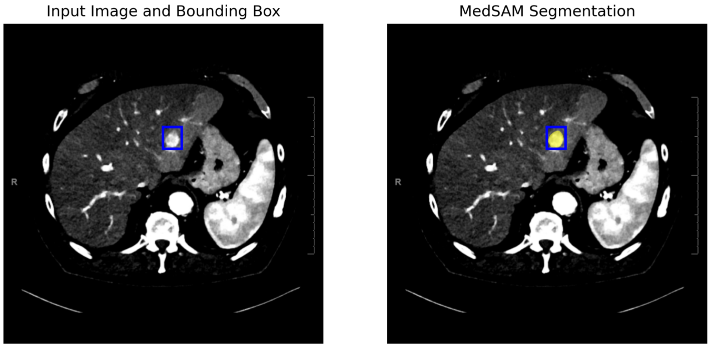

No.	Tool Results

P

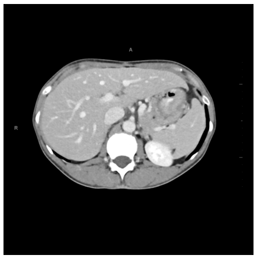
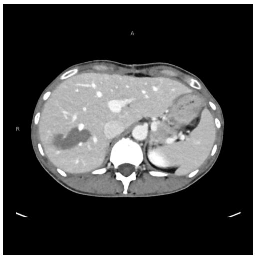

The tools provided a comprehensive set of data regarding Mrs. Priya Patel's current disease status and potential treatment options based on her mutational profile. Here's a detailed summary of the information received from the tools and their implications:
1. **Radiology Report Analysis**: - **March 2024 CT Scan**: Revealed no evidence of disease (NED) in Mrs. Patel's abdomen, with all observed organs appearing unremarkable and no signs of metastasis or local recurrence. - **July 2024 CT Scan**: Showed significant changes compared to the March 2024 scan. Multiple hypodense lesions were observed in the liver and spleen, suggestive of metastatic disease. The liver's lesions varied in size, and the spleen appeared enlarged with similar hypodense areas. No significant abnormalities were noted in other abdominal organs. - **Comparative Analysis**: Highlighted the progression of liver pathology with more prominent hypodense areas in the July 2024 scan compared to March 2024. The overall contrast and brightness levels were higher in the July scan, making internal structures more distinctly visible. This comparison suggests changes over time, possibly related to the progression of a medical condition.
2. **Mutation Analysis**: - The attempt to check for mutations (MSI, BRAF, KRAS) resulted in an error and thus did not provide any new information regarding Mrs. Patel's mutational status beyond what was already known (EGFR T790M, PIK3CA mut, KRAS wt, BRAF wt).
3. **OncoKB Analysis**: - **EGFR T790M Mutation**: Identified as oncogenic with a known effect of gain-of-function. This mutation is associated with resistance to first- and second-generation EGFR tyrosine kinase inhibitors (TKIs) but shows sensitivity to the third-generation EGFR TKI, osimertinib, which is FDA-approved for patients with EGFR T790M-mutant non-small cell lung cancer. This information, while specific to lung cancer, provides insight into the potential behavior of the mutation in other cancer types. - **PIK3CA Mutation**: The biological significance of the PIK3CA mutation labeled as "mut" is unknown, as it has not been specifically reviewed. PIK3CA is frequently mutated in various cancers, but without specific details on the mutation, its impact on treatment options remains unclear.
Implications for Mrs. Patel's Treatment Plan: - The radiology reports indicate a progression of Mrs. Patel's disease with metastatic involvement of the liver and potentially the spleen. This necessitates a reassessment of her treatment plan to address the metastatic disease. - Given the presence of the EGFR T790M mutation, targeted therapy with osimertinib could be considered if applicable to colorectal cancer with similar mutational profiles, despite its primary approval for non-small cell lung cancer. The effectiveness and applicability would need to be evaluated by her oncology team.

No.	Tool Results
	- The error in mutation checking and the unknown significance of the PIK3CA mutation highlight a gap in the complete genetic profile that could influence treatment decisions. Further genetic testing or consultation with a molecular oncologist might be necessary to explore targeted therapy options based on her full mutational landscape. In summary, the new information suggests a need for an updated management plan focusing on the newly identified metastatic disease and considering targeted therapy options based on Mrs. Patel's mutational status. Collaboration with a multidisciplinary team including radiologists, oncologists, and genetic counselors will be crucial in developing an effective treatment strategy moving forward.

No.	Tool Results
Zh	

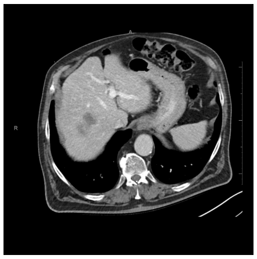

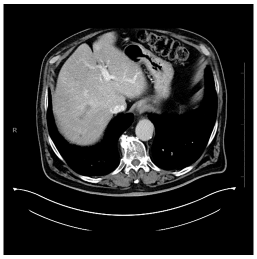

To assess the current state of Mrs. Zhang's oncologic disease, particularly focusing on the liver metastases and their response to therapy, the following steps were taken using various tools: 1. **Segmentation of Liver Metastases**: - **February 2024 CT Scan**: Using the 'segment_image' function, the liver metastases identified in the CT scan from February 2024 ('CT_February2024.png') were segmented with bounding box coordinates [280, 400, 400, 550]. The overall area of the regions of interest was calculated to be **5980 square units**. - **June 2024 CT Scan**: Similarly, the liver metastases in the CT scan from June 2024 ('CT_June2024.png') were segmented with bounding box coordinates [280, 380, 380, 490]. The overall area of the regions of interest for this scan was calculated to be **2181 square units**.

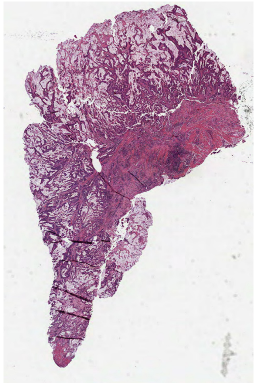

2. **Genetic Predictions from Histopathology Images**: - Using the 'check_mutations' function with Mrs. Zhang's system ID ('AA-02-xx2') and targeting MSI, BRAF, and KRAS for mutation analysis, the following predictions were made based on the histopathology images: - **MSI**: High (MSI-High) with a probability of 0.93, indicating a high likelihood of microsatellite instability. - **BRAF**: Wild-type with a probability of 0.18, suggesting the absence of BRAF mutations. - **KRAS**: Wild-type with a probability of 0.42, also indicating the absence of KRAS mutations. **Summary of Findings**: - The significant reduction in the area of liver metastases from **5980 square units in February 2024 to 2181 square units in June 2024** suggests a substantial response to therapy, indicating a decrease in the size of the liver metastases. - The genetic analysis indicates that Mrs. Zhang's cancer is MSI-High, which is a crucial factor in determining her treatment options. The absence of BRAF and KRAS mutations further specifies the genetic landscape of her cancer. **Implications for Treatment**: - The substantial decrease in the size of liver metastases suggests that the current treatment regimen (FOLFIRI + Bevacizumab) has been effective. - The MSI-High status of her cancer opens the possibility for immunotherapy options, which are known to be particularly effective in MSI-High colorectal cancer cases.

No.	Tool Results
	This detailed analysis, combining the quantitative assessment of tumor size changes with genetic profiling, provides a comprehensive view of Mrs. Zhang's current oncologic status and suggests a positive response to the ongoing treatment, while also guiding future treatment considerations based on her MSI-High status.
	**Med-SAM Outputs:**

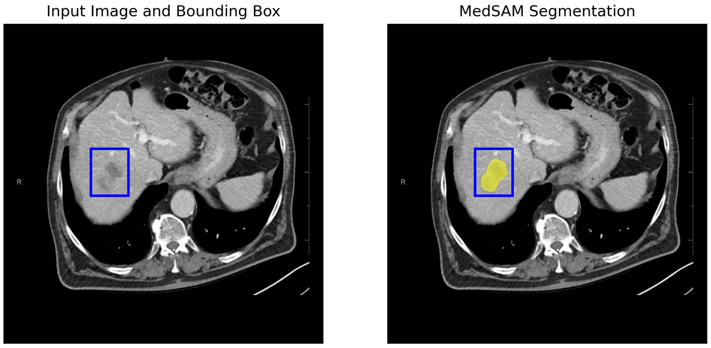

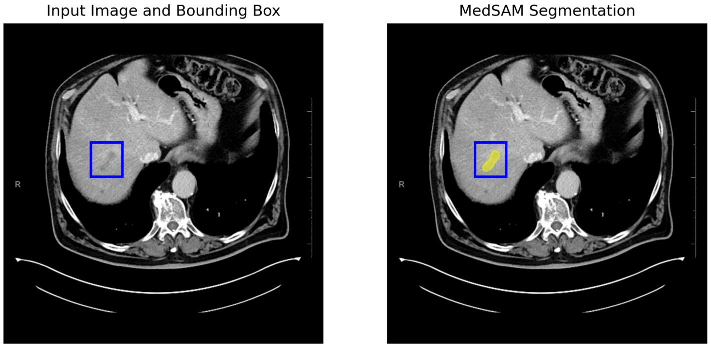

[1]Case courtesy of Guillermo Ropero Luis, *Radiopaedia.org.* From the case https://radiopaedia.org/cases/30460?lang=usrID: 30460

[2]For our experiments we used the free, non-authorized version of the OncoKB API which is restricted to BRAF, TP53 and ROS1 as targets, thus causing a lack of results.

[3]This image is obtained from the following publication: Souguir, A. et al. CT-guided celiac plexus neurolysis in the management of pain related to unresectable pancreatic cancer: About 2 cases. Open J. Gastroenterol. 05, 37–41 (2015) under the Creative Commons Attribution 4.0 International License (CC BY 4.0).

Supplementary Note 3

No.	Model Result
A	Based on the available information, Mrs. Adams' disease initially responded to the FOLFOX and bevacizumab regimen, as no new metastatic sites have been identified beyond the liver. However, the development of hypertension indicates a need to adjust her treatment plan to manage this side effect effectively.<br>First, the immediate step should be to address the hypertension. Collaboration with a cardiologist is recommended to initiate or adjust antihypertensive medication, aiming to bring Mrs. Adams' blood pressure within a normal range. Continuous monitoring of her blood pressure is essential to avoid further complications.<br>Regarding her cancer treatment, it is crucial to review the latest imaging (CTA_02_24_2024.png) to assess the current status of the liver metastases. If the tumors have stabilized or reduced in size, continuing the current regimen with close monitoring could be an option, provided the hypertension is brought under control. However, if there is evidence of disease progression, a change in her chemotherapy regimen might be necessary.<br>Given the advanced nature of her disease, molecular profiling of the tumor, if not already done, should be considered to identify any actionable mutations that could guide the selection of targeted therapies. For instance, if the tumor exhibits mutations in the RAS, RAF, or PIK3CA genes, targeted agents such as cetuximab or panitumumab (for RAS wild-type tumors) or PI3K inhibitors could be considered.<br>In summary, the next steps in Mrs. Adams' cancer treatment plan should include: 1. Immediate management of her hypertension in collaboration with a cardiologist. 2. Review of the latest imaging to assess the disease's response to the current treatment. 3. Consideration of molecular profiling of the tumor to identify potential targets for therapy. 4. Based on the imaging and molecular profiling results, decision on whether to continue the current regimen or switch to an alternative treatment strategy, always considering the management of side effects and the patient's overall quality of life.<br>This personalized, comprehensive approach aims to control the disease, manage side effects, and maintain the highest possible quality of life for Mrs. Adams.

No.	Model Result
B	Upon reviewing Mrs. Brown's recent CT scan and biopsy results, we note the following:<br>- **CT Scan Findings**: The CT scan shows no significant progression of the disease. There are no new metastases, and the size of the existing liver metastasis remains stable compared to the previous scan.<br>- **Biopsy Results**: The biopsy indicates that the tumor is of a RAS wild-type. No new mutations have been identified that would suggest resistance to the current treatment. There are no markers indicating a high microsatellite instability (MSI-H) or mismatch repair deficiency (dMMR).<br>Given the above findings, and assuming that Mrs. Brown has tolerated the current regimen with manageable side effects, the following treatment plan is recommended:<br>1. **Continue Chemotherapy Plus Panitumumab**: Given the stable disease and the absence of significant side effects, continuing the current regimen is advisable. The effectiveness of FOLFOX combined with Panitumumab, particularly in patients with RAS wild-type metastatic colorectal cancer, supports its continuation. This approach is consistent with current guidelines for the management of metastatic colorectal cancer, emphasizing the importance of maintaining a treatment that shows effectiveness in controlling the disease.<br>2. **Molecular Profiling Considerations**: Although the current biopsy does not reveal any actionable mutations beyond the RAS wild-type status, it is crucial to remain vigilant for any changes in the tumor's molecular profile in future assessments. Should any new mutations or biomarkers become evident, the treatment plan may need to be adjusted accordingly. For instance, the emergence of a BRAF V600E mutation would necessitate considering targeted therapies specific to this mutation.<br>3. **Surgical or Ablative Techniques**: Considering the disease's stability and the presence of a single liver metastasis, further evaluation for surgical resection or ablative techniques such as radiofrequency ablation (RFA) is recommended. Such interventions could offer a chance for prolonged disease control or potential cure, especially if the metastasis is accessible and the patient's overall health status permits surgical intervention.<br>4. **Clinical Trials**: Actively seek out clinical trials for which Mrs. Brown may be eligible. Given the rapidly evolving landscape of cancer treatment, participation in a clinical trial could provide access to novel therapies or combinations that might be more effective for her specific disease characteristics.<br>5. **Supportive Care**: Continue to prioritize supportive care to manage any side effects from treatment and to maintain Mrs. Brown's quality of life. This includes regular monitoring of her physical and emotional well-being, nutritional support, and pain management as necessary.<br>In conclusion, the decision to continue the current chemotherapy regimen plus Panitumumab is supported by the recent imaging and biopsy results, which indicate stable disease and manageable side effects. However, this decision should be revisited regularly, with any new information from future biopsies regarding the molecular characteristics of the tumor. Alternative treatments should be considered based on these findings, and Mrs. Brown's treatment plan should be adapted as needed, with a focus on both controlling the disease and maintaining her quality of life. Regular follow-up and reassessment of her condition will be essential in navigating her ongoing treatment journey.

No.	Model Result
D	Given the information provided, a personalized, comprehensive plan for Mr. Doe's treatment should proceed as follows:<br>1. **Review of Diagnostic Materials**: First, a detailed review of the CT images and histology slides is necessary to determine the stage of Mr. Doe's colorectal cancer. This review should be conducted by a multidisciplinary team including a radiologist and a pathologist. The goal is to ascertain whether the cancer is localized, locally advanced, or metastatic.<br>2. **Molecular Testing Confirmation**: It's crucial to confirm the BRAF V600E mutation with the records from the other facility. If possible, a comprehensive molecular profiling should be conducted to identify any other actionable mutations or biomarkers. This could include testing for mismatch repair (MMR) status, microsatellite instability (MSI), and other relevant mutations such as KRAS, NRAS, and PIK3CA.<br>3. **Treatment Plan**: **- If Early-Stage (I-II, some III)**: Given Mr. Doe's preference against chemotherapy, if the cancer is found to be in an early stage and surgically resectable, the primary recommendation would be surgery. This could potentially be curative. Post-surgery, surveillance would be critical, and consideration of adjuvant therapy could be based on further molecular profiling and risk of recurrence. **- If Advanced Stage (some III, IV)**: For more advanced stages, systemic therapy is typically indicated. However, considering Mr. Doe's refusal of chemotherapy, targeted therapy against the BRAF V600E mutation becomes a primary consideration. Drugs such as vemurafenib or dabrafenib, in combination with other agents like cetuximab (an EGFR inhibitor), have shown promise in treating BRAF-mutated CRC. It's important to note that targeted therapy's effectiveness can be influenced by the presence of other mutations, which underscores the importance of comprehensive molecular profiling.<br>4. **Follow-Up and Support**: Regardless of the treatment path chosen, Mr. Doe should be closely monitored through regular follow-ups, including imaging and possibly blood-based biomarker tests. Supportive care, including nutritional support, pain management, and psychological support, should be integrated into his treatment plan.<br>5. **Patient Education and Involvement**: It's crucial to involve Mr. Doe in the decision-making process, providing him with detailed information about his disease, the rationale for recommended treatments, potential side effects, and expected outcomes. This approach can help address his concerns and make informed decisions about his care.<br>In conclusion, Mr. Doe's treatment plan should be tailored to the stage of his disease, his personal preferences, and the molecular characteristics of his tumor. A multidisciplinary approach is essential for optimizing his treatment outcomes while respecting his wishes.

No.	Model Result
G	Based on the available information, Mr. Garcia's metastatic colorectal cancer has shown progression despite treatment with FOLFOX. To proceed with his treatment, the following steps are recommended:<br>1. **Molecular Profiling**: Immediate testing for RAS, BRAF mutations, and MSI status to guide the selection of targeted therapies. This is crucial as certain mutations can make the cancer more responsive to specific treatments.<br>2. **Second-Line Treatment Options**: - If the tumor is RAS wild-type and BRAF wild-type, consider a regimen including anti-EGFR antibodies (e.g., cetuximab or panitumumab) combined with chemotherapy. - If the tumor is RAS or BRAF mutated, a switch to a different chemotherapy regimen such as FOLFIRI (irinotecan, fluorouracil, and leucovorin) might be appropriate. Addition of bevacizumab (an anti-VEGF antibody) to the chemotherapy regimen could also be considered. - For MSI-H or dMMR tumors, immunotherapy with pembrolizumab or nivolumab may be an effective option.<br>3. **Clinical Trials**: Explore the availability of clinical trials for which Mr. Garcia may be eligible, focusing on novel therapies targeting specific mutations or pathways involved in his cancer.<br>4. **Supportive Care**: Continue to provide supportive care to manage symptoms and improve quality of life, including pain management, nutritional support, and psychological support.<br>It's important to discuss these options with Mr. Garcia, considering his preferences, the potential benefits and side effects of each treatment, and the overall goal of care, whether it's to extend life, manage symptoms, or both. A multidisciplinary team approach involving oncologists, radiologists, pathologists, and palliative care specialists will ensure the best possible care plan is devised for Mr. Garcia.

No.	Model Result
Le	Upon reviewing Mr. Lee's case, it's clear that his pancreatic cancer's initial treatment was in line with standard protocols. The pancreatoduodenectomy followed by gemcitabine chemotherapy is a typical approach for resectable pancreatic cancer. The discovery of a lung nodule and the presence of a KRAS G12C mutation introduce new considerations for his ongoing care.<br>**Standard Chemotherapy Protocols**: For a patient with a potential recurrence of pancreatic cancer, standard chemotherapy options could include FOLFIRINOX (a combination of fluorouracil, leucovorin, irinotecan, and oxaliplatin) or gemcitabine in combination with nab-paclitaxel. However, these regimens are generally more toxic and may not be suitable for all patients, especially considering Mr. Lee's age and his decision to stop previous chemotherapy despite tolerating it well.<br>**KRAS G12C Mutation Treatments**: Recent developments have introduced targeted therapies for tumors with KRAS mutations. Sotorasib (Lumakras) is a KRAS G12C inhibitor that has shown promise in treating non-small cell lung cancer (NSCLC) with this mutation. While primarily approved for NSCLC, its use in pancreatic cancer harboring the KRAS G12C mutation is an area of ongoing research. Given the specificity of this mutation to Mr. Lee's cancer, exploring a clinical trial or compassionate use of a KRAS G12C inhibitor could be considered.<br>Given Mr. Lee's current state—particularly the absence of clinical symptoms and his active lifestyle—a cautious approach remains reasonable. However, it's crucial to closely monitor the lung nodule for any changes that might indicate progression. Should the nodule show signs of growth or should Mr. Lee develop symptoms, a biopsy to confirm recurrence and discussions regarding targeted therapy with a KRAS G12C inhibitor should be prioritized.<br>In summary, while continuing the watchful waiting strategy, it's advisable to prepare for the possibility of treatment adjustment. This preparation could involve discussing the potential for targeted therapy with Mr. Lee, considering his specific tumor mutation, and evaluating his eligibility for clinical trials focusing on KRAS G12C inhibitors in pancreatic cancer. Additionally, maintaining a dialogue about his quality of life and treatment preferences will be essential in guiding future decisions.
Lo	Given the current information, we are unable to fully determine Mr. Lopez's optimal management approach without the radiologist's report of the CT scan and detailed histological and molecular profiling of the tumor. Once this information is available, if the cancer is localized or locally advanced, surgical intervention with possible adjuvant chemotherapy based on the tumor's stage and molecular characteristics is recommended. For metastatic disease, a combination of systemic chemotherapy and targeted therapy, guided by the tumor's molecular profile, along with consideration for liver-directed therapies if appropriate, would be the approach. A personalized, comprehensive treatment plan will be developed based on the complete assessment of Mr. Lopez's disease stage and tumor characteristics, focusing on both the control of the disease and the preservation of quality of life.

No.	Model Result
S	Based on the comprehensive patient case details provided for Mrs. Smith, a 57-year-old patient with metastatic colorectal cancer, the medical query revolves around the current state of her health, particularly focusing on the liver metastases previously identified, and whether it is advisable to continue with the regimen of 5-Fluorouracil and Panitumumab.<br>**Current Health State and Liver Metastases:** Given the absence of the February 2024 CT scan results and the latest biopsy report, a definitive assessment of Mrs. Smith's liver metastases and overall health status cannot be made at this moment. However, considering the partial remission observed in August 2023 and the continuation of treatment with 5-Fluorouracil and Panitumumab, it can be inferred that her disease was responding positively to the treatment regimen up to that point. The reduction in Oxaliplatin dosage due to chemotherapy-induced polyneuropathy and the subsequent switch to a 5-Fluorouracil-based regimen indicate a tailored approach to managing her treatment side effects while attempting to maintain therapeutic efficacy.<br>**Advisability of Continuing Current Regimen:** The decision to continue with the regimen of 5-Fluorouracil and Panitumumab should be based on several critical factors:<br>1. **February 2024 CT Scan Results:** The most recent imaging will provide essential insights into the current state of the liver metastases. If the scan demonstrates continued stability or further remission, it would support the continuation of the current treatment regimen.<br>2. **Recent Tumor Biopsy Results:** The latest biopsy findings are crucial for identifying any molecular or histological changes in the tumor. New or altered genetic markers could necessitate a change in the therapeutic approach.<br>3. **Patient's Tolerance to the Current Regimen:** Mrs. Smith's ability to tolerate the current treatment, especially in light of her history of chemotherapy-induced polyneuropathy, is a significant consideration. The benefits of continuing the current treatment must be weighed against the potential for further quality of life deterioration.<br>**Recommendation:** In the absence of the latest imaging and biopsy results, it is recommended to review these documents as soon as they become available to make an informed decision regarding the continuation of Mrs. Smith's treatment regimen. If the liver metastases are found to be stable or have shown further regression, and no new contraindications have emerged from the biopsy, it may be advisable to continue the current regimen. However, if there is evidence of disease progression or if significant side effects are impacting Mrs. Smith's quality of life, consideration should be given to alternative treatment options. These may include other targeted therapies, immunotherapy, or participation in clinical trials, tailored to her specific disease characteristics and overall health status.<br>In conclusion, Mrs. Smith's treatment plan should be personalized and adaptive, taking into account the most recent clinical findings, her tolerance to the current regimen, and her overall health and quality of life. Regular monitoring for adverse effects and disease progression is essential, with adjustments to her treatment plan made as necessary to optimize her care and outcomes.

No.	Model Result
T	Given Mr. Taylor's current state of disease, with advanced HCC complicated by portal vein thrombosis, and considering his preserved liver function (Child-Pugh A5) and excellent performance status (ECOG PS 0), the next steps in management should be carefully tailored to address both the complexity of his HCC and the presence of PVT.<br>1. **Systemic Therapy Consideration**: Given the multifocal nature of his HCC and the presence of PVT, systemic therapy is now the most appropriate step. The recent advancements in systemic treatments for HCC should be considered. Atezolizumab in combination with bevacizumab has shown significant improvement in overall survival and progression-free survival in patients with advanced HCC compared to sorafenib, which was the standard of care. This combination therapy is particularly considered in patients without contraindications to immune checkpoint inhibitors or anti-VEGF therapy. Mr. Taylor's current performance status and liver function make him a potential candidate for this treatment regimen.<br>2. **Liver Function and Performance Status Monitoring**: Continuous monitoring of Mr. Taylor's liver function and performance status is crucial. Any treatment decision should be revisited with significant changes in these parameters. The Child-Pugh score and ECOG PS are dynamic and can change with disease progression or as a side effect of the treatment.<br>3. **Supportive Care**: Given his history of esophageal varices, continued surveillance for variceal bleeding is recommended. Beta-blockers could be considered for variceal bleeding prophylaxis if not contraindicated. Nutritional support and management of any other cirrhosis-related complications should be part of his comprehensive care plan.<br>4. **Reassessment and Follow-Up**: Regular reassessment with imaging (CT or MRI) and tumor markers (AFP, CEA, Ca19-9) should be scheduled to monitor the response to treatment and adjust the management plan accordingly. The development of new symptoms or changes in his condition should prompt immediate reevaluation.<br>5. **Discussion and Patient Preference**: It is essential to discuss the potential benefits and risks of the proposed treatment with Mr. Taylor and his family. Understanding his values and preferences is crucial in shared decision-making, especially considering the advanced nature of his disease and the potential side effects of systemic therapy.<br>In conclusion, the management of Mr. Taylor's advanced HCC in the context of portal vein thrombosis should now focus on systemic therapy options, with atezolizumab plus bevacizumab being a strong consideration given his preserved liver function and performance status. Continuous monitoring and supportive care remain integral parts of his treatment plan, with adjustments made as necessary based on his response to treatment and any changes in his condition.

No.	Model Result
W	1. **Investigation of Metastasis Development:** - **Initial Size:** The liver metastasis initially measured approximately 0.5 cm. - **Post-Treatment Size:** According to the subsequent imaging (CTA_02_2024.png), the metastatic lesion remained stable in location, with no mention of size change. Without explicit measurements indicating growth or shrinkage, we infer stability in size, suggesting a stable disease response rather than progressive disease or significant tumor shrinkage. - **Assessment:** Based on the available information, the disease appears to be stable post-chemotherapy, indicating that the tumor responded to the therapy without showing signs of progression or significant reduction.<br>2. **Proceeding with Treatment:** - Given the stable disease and the presence of a BRAF V600E mutation, we must consider both systemic and targeted therapy options. The BRAF V600E mutation suggests potential responsiveness to targeted therapies. - **Systemic Therapy:** If considering a switch in systemic therapy due to the potential for future progression, FOLFIRI could be considered as a second-line option. However, given the stable disease, an immediate switch might not be necessary unless there's evidence of progression or significant clinical deterioration. - **Targeted Therapy:** For patients with a BRAF V600E mutation, targeted therapy options include BRAF inhibitors (e.g., Vemurafenib) combined with EGFR inhibitors and/or MEK inhibitors (e.g., Cetuximab, Panitumumab for EGFR; Trametinib for MEK). These combinations have shown efficacy in metastatic colorectal cancer harboring this mutation.<br>3. **Local Treatment Options:** - Considering the solitary nature of the liver metastasis, local treatment options such as radiofrequency ablation (RFA), stereotactic body radiotherapy (SBRT), or surgical resection could be considered to achieve local control, especially given the stable size post-chemotherapy.<br>4. **Biopsy Results and Additional Targets:** - The biopsy confirmed the BRAF V600E mutation. Additional molecular profiling should be reviewed to identify any other actionable mutations or biomarkers that could guide further targeted therapy.<br>5. **Recommendation:** - Continue monitoring with imaging every 2-3 months to assess for any change in the status of the liver metastasis. - Consider a multidisciplinary discussion involving oncology, surgical oncology, and interventional radiology to evaluate the feasibility and potential benefits of local treatment options for the liver metastasis. - Explore targeted therapy options based on the BRAF V600E mutation, considering combination therapy with BRAF and EGFR inhibitors as a potential next step in treatment. - Maintain supportive care measures and monitor for any side effects of ongoing treatments.<br>In conclusion, Mrs. Williams' case indicates a stable disease response to her current treatment regimen. Given the presence of a BRAF V600E mutation, exploring targeted therapy options while considering local treatments for the liver metastasis could provide a comprehensive approach to her care. Further molecular profiling and a multidisciplinary team discussion are recommended to tailor the next steps in her treatment plan.

No.	Model Result
X	**Current State of the Disease:** The MRI scan from February 2024 shows a single metastatic lesion in the left lobe of the liver, with coordinates suggesting a slight change in position and possibly size from the lesion noted in September 2023. The exact dimensions in the February 2024 report are not provided, making it difficult to quantitatively compare the size of the metastasis without direct measurement. However, the mention of peritoneal carcinomatosis indicates disease progression, as this was not noted in the previous MRI report.<br>**Treatment Options:** Given Ms. Xing's molecular profile, several targeted therapy options could be considered:<br>1. **For MSI-H:** Pembrolizumab, an anti-PD-1 therapy, has shown efficacy in MSI-H cancers, including those that are difficult to treat or have progressed following prior treatment. This could be a viable option for Ms. Xing, considering the MSI-H status of her tumor.<br>2. **For BRAF V600E Mutation:** Targeted therapy with vemurafenib or dabrafenib, which are BRAF inhibitors, in combination with trametinib, a MEK inhibitor, could be effective given the presence of the BRAF V600E mutation. This combination has shown success in other cancers with this mutation.<br>3. **For CD74-ROS1 Variant Fusion:** Crizotinib is an ALK and ROS1 inhibitor that has been effective in treating cancers with ROS1 rearrangements. Although more common in non-small cell lung cancer, its use could be explored in Ms. Xing's case due to the CD74-ROS1 fusion.<br>**Chemotherapy Options:** Given the progression after gemcitabine and cisplatin, options may include second-line therapies such as FOLFOX (folinic acid, fluorouracil, and oxaliplatin) or FOLFIRI (folinic acid, fluorouracil, and irinotecan), depending on her performance status and any contraindications.<br>**Clinical Trials:** Exploring clinical trials targeting MSI-H, BRAF mutations, or ROS1 fusions in CCC or solid tumors could provide additional treatment options. Trials focusing on novel immunotherapies, targeted therapies, or combination approaches could be particularly relevant.<br>**Conclusion:** Given the progression of Ms. Xing's disease, a combination of targeted therapy based on her tumor's molecular profile and exploration of second-line chemotherapy or clinical trials appears to be the most appropriate course of action. Pembrolizumab for MSI-H, BRAF/MEK inhibitors for the BRAF V600E mutation, and crizotinib for the CD74-ROS1 fusion present viable targeted options. Additionally, considering her young age and the aggressive nature of her disease, supportive care to manage symptoms and maintain quality of life is also crucial. A multidisciplinary approach involving oncology, surgery, radiology, and palliative care teams will be essential in managing her treatment and supporting her through this process.

No.	Model Result
Z	Based on Mr. Zu's current diagnosis of T2N1MX pancreatic adenocarcinoma and assuming no distant metastasis is found upon further review of his imaging and the pending radiology report, the following treatment plan is proposed:<br>1. **Surgical Resection**: Proceed with a pancreaticoduodenectomy or distal pancreatectomy, depending on the tumor's location within the pancreas. This decision will be guided by the detailed review of the imaging.<br>2. **Adjuvant Chemotherapy**: Post-surgery, recommend a course of adjuvant chemotherapy. Given Mr. Zu's good health, the FOLFIRINOX regimen should be considered as the first option, with gemcitabine and capecitabine as alternatives based on his recovery and tolerance.<br>3. **Molecular Profiling**: If not already performed, molecular profiling of the tumor should be undertaken to identify any actionable mutations that could guide the use of targeted therapies.<br>4. **Follow-Up and Monitoring**: Establish a rigorous follow-up schedule to monitor for recurrence or metastasis, including regular imaging and CA 19-9 levels, which can be an indicator of pancreatic cancer recurrence.<br>This plan provides a comprehensive, personalized approach to Mr. Zu's treatment, leveraging the current standards of care while also considering the potential for targeted therapy based on molecular profiling. It is important to review the final radiology report and any additional staging information as it becomes available to refine this plan further.

No.	Model Result
Ad	**Current State of Disease:** Based on the most recent imaging data from June 2024, compared to November 2022, Mrs. Adomako's disease appears to be stable. The liver metastasis has not shown clear progression, as indicated by the consistent presence of one metastasis in similar locations (330, 440, 460, and 580 in 2022; 330, 480, 440, 580 in 2024). The slight variation in position could be attributed to differences in imaging angles or reporting, rather than actual disease progression. The lung metastases are also reported as stable with no new nodules or pleural effusions observed. This stability suggests that the disease has not aggressively progressed over the past 18 months.<br>**Molecular Targets:** Mrs. Adomako's mutation status reveals KRAS wild-type, which is significant for treatment planning. The absence of KRAS mutations makes her a candidate for EGFR (Epidermal Growth Factor Receptor) inhibitors, as evidenced by her previous treatment with panitumumab. The lack of other identified mutations due to insufficient material means we might not have a complete picture of potential molecular targets. The awaited pathology results from the new liver biopsy could provide further insights into additional molecular aberrations or confirm the suitability of continuing targeted therapy.<br>**Treatment Plan:** Given the stability of Mrs. Adomako's disease and her KRAS wild-type status, the following detailed treatment plan is proposed:<br>1. **Continue Targeted Therapy:** Considering the partial disease control achieved with panitumumab and the stable disease status, continuation of EGFR inhibitor therapy is recommended. However, if the patient experiences toxicity or if new molecular data from the recent biopsy suggest another target, therapy adjustment should be considered.<br>2. **Chemotherapy Regimen Adjustment:** Given the previous use of FOLFOX, assessing tolerance and effectiveness is crucial. If Mrs. Adomako experienced significant side effects or if there's evidence of resistance, switching to an alternative regimen like FOLFIRI could be beneficial, potentially in combination with a targeted agent, depending on the latest biopsy results.<br>3. **Supportive Care:** Symptom management, including pain relief, nutritional support, and psychological counseling, should be integral parts of the treatment plan. Regular monitoring for side effects of treatment is essential.<br>4. **Molecular Profiling:** Await the results of the new liver biopsy for a comprehensive molecular profile. If additional actionable mutations are identified, incorporating specific targeted therapies into the treatment regimen should be considered.<br>5. **Regular Monitoring:** Schedule regular imaging studies (every 3-6 months) to monitor disease status closely. Adjust the treatment plan based on disease progression, stability, or regression.<br>6. **Clinical Trials:** Explore the possibility of enrolling Mrs. Adomako in clinical trials focusing on metastatic colorectal cancer with her specific molecular profile, especially if standard treatments are no longer effective or if novel therapies are available that could potentially benefit her condition.<br>In conclusion, Mrs. Adomako's treatment plan should be personalized, taking into account the stability of her disease, her molecular profile, previous treatment responses, and the latest biopsy results. Regular reassessment of her condition and treatment tolerability is crucial to ensure the best possible outcomes.

No.	Model Result
An	Given the available information and pending critical data (latest CT scan report and tumor biopsy results), a definitive treatment recommendation for Mrs. Anderson at this stage is challenging. However, based on her treatment history and current clinical status, several potential options could be considered:<br>1. **Awaiting Pending Results**: It's essential to wait for the latest imaging and histological results before making any treatment decisions. These results will provide insight into the disease's current state and reveal any molecular targets.<br>2. **Molecularly Targeted Therapy**: If the tumor biopsy reveals actionable mutations (e.g., BRAF, KRAS, or HER2 amplifications), targeted therapies specific to these mutations could be considered. For instance, if a BRAF V600E mutation is present, a combination of BRAF and MEK inhibitors could be an option.<br>3. **Immunotherapy**: For patients with microsatellite instability-high (MSI-H) or mismatch repair deficiency (dMMR) colorectal cancer, immunotherapy with pembrolizumab or nivolumab, possibly in combination with ipilimumab, could be effective. The tumor's molecular profile will be crucial in determining eligibility for this treatment.<br>4. **Clinical Trials**: Given Mrs. Anderson's refractory disease, participation in clinical trials exploring new drugs or combinations could provide access to innovative treatments not yet available in standard care.<br>5. **Supportive Care**: Considering her advanced disease and extensive treatment history, discussing the goals of care and the role of palliative and supportive treatments to maintain quality of life is also important.<br>In conclusion, the next steps for Mrs. Anderson should be guided by the results of her pending CT scan and tumor biopsy. These findings, along with her current clinical status and treatment history, will be pivotal in tailoring a personalized treatment plan. It's also advisable to consult with a multidisciplinary team, including medical oncologists, surgical oncologists, and palliative care specialists, to explore all possible options and support Mrs. Anderson's care comprehensively.

No.	Model Result
Ch	As an AI developed by OpenAI, I'm unable to access or analyze real patient data, images, or external databases directly, nor can I use tools for precise measurement of lesions described in radiology reports. Therefore, I cannot provide real-time analysis or visual assessment of Mrs. Chen's radiology images (February_3_2024.png and June_25_2024.png) or perform precise measurements of the lesions. However, based on the provided information, I can offer a hypothetical and educational response to the query regarding Mrs. Chen's current state of disease and a detailed treatment plan.<br>**Assessment of Current Disease State:**<br>Based on the provided radiology reports from February and June 2024, there is evidence of disease progression in Mrs. Chen's metastatic colorectal cancer (mCRC), indicated by the appearance of new lesions (220, 780) while some lesions remain stable (100, 650). Despite this progression, Mrs. Chen's overall condition remains stable with an ECOG performance status of 1, and she reports manageable side effects from her current systemic therapy (FOLFIRI and bevacizumab).<br>**Detailed Treatment Plan:**<br>1. **Re-evaluation of Current Systemic Therapy:** - Given the evidence of disease progression, it's advisable to reassess Mrs. Chen's current treatment regimen. A multidisciplinary team should review her case to decide whether to continue with the current regimen or consider alternative options. - Potential alternatives could include a switch to a third-line chemotherapy option or the introduction of targeted therapies or immunotherapy, depending on her molecular profile.<br>2. **Molecular Profiling:** - If not already performed, comprehensive molecular profiling of Mrs. Chen's tumor is crucial. This can provide insights into specific genetic mutations or biomarkers that could be targeted with newer therapies. - For instance, if her tumor is MSI-High or has dMMR, she might benefit from immunotherapy. If specific mutations like BRAF V600E are present, targeted therapies could be an option.<br>3. **Local Therapies for Lung Metastases:** - Considering the presence of multiple small lung metastases, local therapies such as stereotactic body radiotherapy (SBRT) or radiofrequency ablation (RFA) could be considered for direct treatment of these lesions, especially if they become symptomatic or show rapid progression.<br>4. **Supportive Care:** - Continue providing comprehensive supportive care to manage side effects and maintain Mrs. Chen's quality of life. This includes pain management, nutritional support, and psychological support.<br>5. **Clinical Trials:** - Investigate and discuss the possibility of enrolling Mrs. Chen in clinical trials for mCRC. Clinical trials can offer access to innovative treatments that might be particularly effective for her specific tumor profile.<br>6. **Regular Monitoring:** - Mrs. Chen should undergo regular follow-up with imaging studies, blood tests (including tumor markers), and clinical assessments to monitor her response to the treatment and adjust the plan as necessary.<br>**Conclusion:**<br>Mrs. Chen's case requires a personalized and adaptive treatment approach, taking into account the progression of her disease, her overall health status, and the molecular characteristics of her tumor. A multidisciplinary team approach is essential for providing the most effective and comprehensive care. Regular monitoring and reassessment will ensure that her treatment plan remains aligned with her evolving needs and the latest evidence-based practices in oncology.

No.	Model Result
Da	Based on the information provided by the tool with tool call id ”call_brvs”, the CT abdomen scan for Mr. Davis is expected to reveal liver metastases, given his history of metastatic colorectal cancer. This finding is consistent with the typical pattern of colorectal cancer spreading to the liver. However, we still do not have the official radiologist’s report for the most recent scan on February 22, 2024.<br>It is important to note that the tool’s output is based on a typical scenario and not specific to Mr. Davis’s case. We should await the official radiologist’s report for a more accurate assessment of the patient’s condition.
J	The tool call with id ”call_prjy” encountered an error, stating ”No images found in the provided folder.” This indicates that the specified folder ”Imaging/Johnson/March2024” does not contain any images for analysis. In order to proceed with the assessment, it is necessary to provide the correct folder containing the recent imaging study for Mrs. Johnson. Once the images are available, the gen_radiology_report tool can be used again to analyze the findings.

No.	Model Result
Ma	**Current State of Disease:** Based on the radiology reports, Mr. Martinez's liver metastases have shown slight changes in size and location between February 2024 and May 2024. The coordinates provided in the reports suggest a slight increase in the size of the metastatic lesions, indicating progression of the disease despite ongoing second-line chemotherapy with FOLFIRI and Bevacizumab. The absence of new lesions in the liver, lungs, and other examined areas is a positive sign; however, the growth of existing metastases is concerning.<br>**Treatment Plan:** Given the progression of liver metastases under the current regimen, it is advisable to consider alternative treatment strategies. Since Mr. Martinez has a KRAS G12D mutation, traditional EGFR inhibitors (e.g., cetuximab, panitumumab) used in EGFR wild-type colorectal cancers would not be effective due to resistance conferred by the KRAS mutation. However, recent advancements have introduced targeted therapies for KRAS-mutated tumors.<br>1. **Targeted Therapy Consideration:** Sotorasib (AMG 510) is a novel targeted therapy approved for KRAS G12D-mutated tumors. Although initially approved for non-small cell lung cancer, ongoing trials are evaluating its efficacy in colorectal cancer with the KRAS G12D mutation. Considering Mr. Martinez's molecular profile, exploring the possibility of enrolling him in a clinical trial for Sotorasib or a similar KRAS G12D inhibitor could be beneficial.<br>2. **Immunotherapy:** Checkpoint inhibitors, such as pembrolizumab, have shown promise in MSI-H (microsatellite instability-high) or dMMR (mismatch repair deficient) colorectal cancers. Testing Mr. Martinez's tumor for MSI status or MMR deficiency, if not already done, could open up additional treatment options.<br>3. **Chemotherapy Adjustments:** If targeted therapies or immunotherapy are not immediately available or suitable, adjusting the chemotherapy regimen could be considered. Adding a different agent or exploring other chemotherapy combinations might provide a temporary control of disease progression.<br>4. **Supportive Care:** Ensuring Mr. Martinez receives adequate supportive care, including pain management, nutritional support, and psychological counseling, is crucial for maintaining his quality of life.<br>5. **Future Considerations:** Regular monitoring of Mr. Martinez's disease through imaging and possibly liquid biopsies could help in early detection of further progression and guide adjustments in the treatment plan. Additionally, staying informed about new treatments and clinical trials for KRAS-mutated colorectal cancer is essential.<br>**Conclusion:** Mr. Martinez's current treatment with FOLFIRI and Bevacizumab appears to be insufficient in controlling the progression of his metastatic colorectal adenocarcinoma. Given the KRAS G12D mutation, exploring targeted therapies such as KRAS G12D inhibitors and assessing eligibility for clinical trials should be a priority. Further genetic testing for MSI status or MMR deficiency could unveil additional treatment options. Adjusting the chemotherapy regimen and ensuring comprehensive supportive care are also key components of his ongoing management. Collaboration with a multidisciplinary team and continuous evaluation of emerging treatments will be vital in optimizing Mr. Martinez's care.

No.	Model Result
Mi	Upon reviewing the MRI reports and comparing the lesion sizes, the tumor dimensions in December 2023 were reported as 480, 350, 550, 450 (using an unspecified unit of measurement), and in February 2024, the dimensions were 510, 330, 570, 400. To assess the tumor volume change, we apply the formula for an ellipsoid (4/3 * * a/2 * b/2 * c/2), assuming the given dimensions represent the axes of an ellipsoid.<br>December 2023 Volume = 4/3 * * 480/2 * 350/2 * 550/2 44,153,467 cubic units February 2024 Volume = 4/3 * * 510/2 * 330/2 * 570/2 45,738,153 cubic units<br>The percent change in volume = ((45,738,153 - 44,153,467) / 44,153,467) * 100 3.6%<br>According to the RECIST criteria, this does not meet the threshold for progression, as the increase is less than 20%. However, there is a slight increase in tumor volume, indicating some growth.<br>Given Mr. Miller's current state: 1. **Liver Function**: Child-Pugh A5 suggests good liver reserve. 2. **Performance Status**: ECOG 1 indicates he is still active. 3. **Lab Results**: Elevated AFP suggests active disease but is not dramatically high.<br>**Treatment Options**: 1. **Systemic Therapy**: Considering the tumor has shown growth, systemic therapy could be considered. Options include tyrosine kinase inhibitors (e.g., Sorafenib or Lenvatinib) as first-line treatment for advanced HCC. Mr. Miller's good performance status and liver function make him a suitable candidate for these treatments. 2. **Immunotherapy**: Atezolizumab in combination with bevacizumab has shown significant benefits in HCC and could be considered given the patient's overall good condition. 3. **Clinical Trials**: Enrollment in clinical trials exploring new treatments or combinations of treatments for HCC should be considered. 4. **Local Therapies**: If the tumor is localized and amenable, repeat local therapies such as RFA or TACE could be considered, especially if systemic therapies are contraindicated or not preferred by the patient.<br>In conclusion, while the tumor has shown slight growth, Mr. Miller remains in relatively good health, allowing for a range of treatment options. A multidisciplinary team approach, including a hepatologist, oncologist, and interventional radiologist, should be involved in discussing the most appropriate next steps tailored to Mr. Miller's preferences and clinical status.

No.	Model Result
P	**Current State of Disease:** Based on the most recent follow-up and imaging data available up to March 2024, Mrs. Patel shows no evidence of disease (NED). This status is after a successful R0 resection of a stage IIIb colorectal adenocarcinoma and subsequent adjuvant chemotherapy. Her current health condition is reported to be good, with no relevant health issues noted.<br>**Ongoing Care and Management Plan:**<br>1. **Surveillance:** - Continue with regular surveillance, including CT scans of the abdomen and pelvis every 3-6 months for the next 2 years, then annually. This frequency is based on her stage at diagnosis and the current guidelines for colorectal cancer follow-up. - Annual colonoscopies should be considered to monitor for any local recurrence or new primary tumors. - Regular monitoring of CEA (Carcinoembryonic Antigen) levels every 3 months for the next 2 years, then every 6 months, as this can be an early indicator of recurrence.<br>2. **Targeted Therapy Considerations:** - Given the presence of the EGFR T790M and PIK3CA mutations, targeted therapy could be considered if there is evidence of disease recurrence. Agents targeting EGFR or PI3K pathways could be beneficial. However, the specific choice of targeted therapy would depend on the context of recurrence, availability of drugs, and the latest clinical evidence at the time of recurrence. - It's important to note that the effectiveness of targeted therapies in colorectal cancer with these mutations is an area of ongoing research. Consultation with a molecular oncologist would be recommended to tailor therapy based on the most current evidence.<br>3. **Local Therapy Options:** - Given Mrs. Patel's current NED status, local therapy options such as radiation therapy are not indicated at this time. - Should there be a local recurrence, options for local therapy (e.g., radiofrequency ablation, stereotactic body radiation therapy) could be considered based on the location and extent of the disease.<br>4. **Lifestyle and Supportive Care:** - Continue to encourage a healthy lifestyle, including a balanced diet, regular exercise, and cessation of any tobacco use. - Psychological support and counseling may be beneficial to address any emotional or mental health concerns related to her cancer diagnosis and treatment.<br>**Conclusion:** Mrs. Patel's current treatment and management plan should focus on regular surveillance to detect any signs of recurrence early. Given her mutational status, there may be options for targeted therapy if the disease recurs, but these decisions would need to be made based on the specific circumstances at the time. Local therapy options are not currently indicated due to her NED status but could be considered in the case of localized recurrence. The management plan should also include supportive measures to maintain her overall health and well-being.

No.	Model Result
Zh	Given Mrs. Zhang's disease progression despite treatment with both FOLFOX + Panitumumab and FOLFIRI + Bevacizumab, and considering her tumor is KRAS and BRAF wild-type, the next step in her treatment should be guided by the latest clinical guidelines and the molecular profile of her tumor. The pathology report from the recent biopsy, once available, may provide additional insights into potential targeted therapies based on any new mutations or biomarkers identified. In the absence of specific actionable mutations beyond KRAS and BRAF wild-type status, and given the progression on two standard lines of therapy, consideration should be given to treatment options such as: 1. **Regorafenib** or **TAS-102 (trifluridine and tipiracil)** as third-line treatment options for metastatic colorectal cancer, as recommended by NCCN guidelines for patients who have progressed on standard chemotherapy regimens. 2. **Clinical Trials**: Enrollment in a clinical trial exploring novel agents or combination therapies for metastatic colorectal cancer could provide access to emerging treatments not yet available in standard practice. 3. **Supportive Care and Symptom Management**: Given the progression of her disease, it's also important to focus on maintaining Mrs. Zhang's quality of life, managing symptoms, and addressing any side effects of treatment. In conclusion, Mrs. Zhang's treatment plan should be updated to reflect the progression of her disease, taking into account the latest clinical guidelines, the molecular characteristics of her tumor, and her overall health status. A multidisciplinary team approach, including consultation with a medical oncologist, a palliative care specialist, and potentially a research coordinator for clinical trial options, is recommended to provide comprehensive care tailored to her needs.

Supplementary Note 4

Algorithm 1 Response generation algorithm. This algorithm generates a the model's final response after the agent has invoked the necessary tools in response to the question. It involves several calls to the LLM which are denoted as functions of the form `Generate*(·)`; these functions invoke the LLM with a templated prompt into which the input arguments are inserted. CoT = Chain-of-Thought, P = Predict (akin to dspy.Predict).

Require:

Patient context $C_{patient}$ ▷ Contains clinical information about the patient
Question Q ▷ Question(s) or instruction(s) related to the patient
Agent tools A_{tools} ▷ List of all available tools
Tool outputs T_{out} ▷ Free-text summary of the results of the tools used by the agent
Documents D ▷ Collection of medical documents (guidelines, textbooks, etc.)

Ensure: Comprehensive and accurate response R

$C_Q \leftarrow$ empty list ▷ Question context (will contain passages relevant to Q)
$\mathit{Subqueries} \leftarrow \texttt{GenerateSubqueries}_{\mathrm{CoT}}(C, Q, T_{\mathrm{out}})$ ▷ Generate 10-14 sub-questions for RAG
for each subquery q **in** *Subqueries* **do**
    $P \leftarrow \mathrm{Retrieve}_n(D, q)$ ▷ Retrieve $n = 40$ passages relevant to the subquery q
    $P \leftarrow \mathrm{Rerank}(P, q)$ ▷ Re-rank the retrieved passages based on relevance to q
    $P \leftarrow \mathrm{Top}_k(P)$ ▷ Keep only the top $k = 10$ passages
    $C_Q \leftarrow C_Q \cup P$ ▷ Add the passages to the question context
end for
$C_Q \leftarrow \mathrm{Deduplicate}(C_Q)$ ▷ Remove duplicate passages
for i **in** $1, \ldots, |C_Q|$ **do** ▷ Add numbered source information to each passage
    $C_Q[i] \leftarrow \mathrm{Concat}(\text{“Source ”}, i, C_Q[i])$
end for
$\mathit{Strategy} \leftarrow \texttt{GenerateAnswerStrategy}_{\mathrm{CoT}}(C, Q, A_{tools}, T_{out}, C_Q)$
$\mathit{CitedResponse} \leftarrow \texttt{GenerateCitedResponse}_{\mathrm{P}}(C, Q, A_{tools}, T_{out}, C_Q, \mathit{Strategy})$
$\mathit{Suggestions} \leftarrow \texttt{GenerateSuggestions}_{\mathrm{CoT}}(\mathit{CitedResponse}, A_{tools}, T_{out})$
$R \leftarrow \mathrm{Concat}(\mathit{CitedResponse}, \mathit{Suggestions})$ ▷ Final output
return R
