# Supplementary material for: Development and validation of an autonomous artificial intelligence agent for clinical decision-making in oncology
Source: Nat Cancer. 2025 Jun 6;6(8):1337–49. doi: 10.1038/s43018-025-00991-6 (PMC12380607; doi:10.1038/s43018-025-00991-6)
Supplement: Supplementary file 2 — Reporting Summary [file 43018_2025_991_MOESM2_ESM.pdf]

nature portfolio

Corresponding author(s): Dyke Ferber, Omar S. M. El Nahhas, Georg Wölflein, Isabella C. Wiest, Jan Clusmann, Marie-Elisabeth Leßmann, Sebastian Foersch, Jacqueline Lammert, Maximilian Tschochohei, Dirk Jäger, Manuel Salto-Tellez, Nikolaus Schultz, Daniel Truhn, Jakob Nikolas Kather

Last updated by author(s): April 10, 2025

Reporting Summary

Nature Portfolio wishes to improve the reproducibility of the work that we publish. This form provides structure for consistency and transparency in reporting. For further information on Nature Portfolio policies, see our Editorial Policies and the Editorial Policy Checklist.

Statistics

For all statistical analyses, confirm that the following items are present in the figure legend, table legend, main text, or Methods section.

n/a	Confirmed	
☐	☒	The exact sample size (n) for each experimental group/condition, given as a discrete number and unit of measurement
☐	☒	A statement on whether measurements were taken from distinct samples or whether the same sample was measured repeatedly
☒	☐	The statistical test(s) used AND whether they are one- or two-sided<br>*Only common tests should be described solely by name; describe more complex techniques in the Methods section.*
☒	☐	A description of all covariates tested
☒	☐	A description of any assumptions or corrections, such as tests of normality and adjustment for multiple comparisons
☒	☐	A full description of the statistical parameters including central tendency (e.g. means) or other basic estimates (e.g. regression coefficient) AND variation (e.g. standard deviation) or associated estimates of uncertainty (e.g. confidence intervals)
☒	☐	For null hypothesis testing, the test statistic (e.g. F, t, r) with confidence intervals, effect sizes, degrees of freedom and P value noted<br>*Give P values as exact values whenever suitable.*
☒	☐	For Bayesian analysis, information on the choice of priors and Markov chain Monte Carlo settings
☒	☐	For hierarchical and complex designs, identification of the appropriate level for tests and full reporting of outcomes
☒	☐	Estimates of effect sizes (e.g. Cohen's d, Pearson's r), indicating how they were calculated

Our web collection on statistics for biologists contains articles on many of the points above.

Software and code

Policy information about availability of computer code

Data collection	Imaging data was downloaded manually. Text data download was partially automated using web-tools including the python libraries requests==2.31.0, beautifulsoup4==4.12.2 and selenium==4.16.0. As this work in parts uses proprietary data, we cannot provide open-access to it, but a large collection of documents used in our study is acessible here: https://huggingface.co/datasets/epfl-llm/guidelines.<br>Additional software packages are: PyMuPDF==1.23.8 for text extraction.<br>Codes to reproduce the agent workflow can be found here: https://github.com/Dyke-F/LLM_RAG_Agent.
Data analysis	Data was analysed using Python 3.11. The core packages used are: OpenAI's python API (1.11.0), dspy (2.1.10), llama_index (0.9.43) and Groq (0.9.0). To implement the LLM-Agent, we use a modification of Llama-index OpenAIAgent that can be found here: https://github.com/run-llama/llama_index/blob/bfd9228e0d7ae63cda726982241f52f283cd6dee/llama-index-legacy/llama_index/legacy/agent/legacy/openai_agent.py#L495. Reranking was performed using cohere (4.45). Data was analysed with pandas (2.1.4). Visualisations were performed in Matplotlib (3.8.2).

For manuscripts utilizing custom algorithms or software that are central to the research but not yet described in published literature, software must be made available to editors and reviewers. We strongly encourage code deposition in a community repository (e.g. GitHub). See the Nature Portfolio guidelines for submitting code & software for further information.

Data

Policy information about availability of data

All manuscripts must include a data availability statement. This statement should provide the following information, where applicable:

- Accession codes, unique identifiers, or web links for publicly available datasets
- A description of any restrictions on data availability
- For clinical datasets or third party data, please ensure that the statement adheres to our policy

The study used image data obtained from the TCGA (histology), TCIA (CT imaging) and internal images from the Department of Diagnostic and Interventional Radiology, University Hospital Aachen, Germany. Text data is obtained from the respective sources as indicated in the main manuscript's references. Individual images are sourced from the web and referenced accordingly in the manuscripts supplementary.

Research involving human participants, their data, or biological material

Policy information about studies with human participants or human data. See also policy information about sex, gender (identity/presentation), and sexual orientation and race, ethnicity and racism.

Reporting on sex and gender	We did not perform real-world sex- or gender based analyses in our study. However, in our study we investigated potential biases of our system by randomly assigning different sex, ages and racial/ethnical factors to different patient cases.
Reporting on race, ethnicity, or other socially relevant groupings	We did not examine real-world racial, ethnical or other socially relevant information in our study.<br>However, in our study we investigated potential biases of our system by randomly assigning different sex, ages and racial/ethnical factors to different patient cases.
Population characteristics	We used artifical patient case descriptions, closely mimicking clinical workflows without investigating patient characteristics.
Recruitment	n/a
Ethics oversight	The overall analysis was approved by the Ethics commission of the Medical Faculty of the Technical University Dresden (BO-EK-444102022).

Note that full information on the approval of the study protocol must also be provided in the manuscript.

Field-specific reporting

Please select the one below that is the best fit for your research. If you are not sure, read the appropriate sections before making your selection.

☒ Life sciences ☐ Behavioural & social sciences ☐ Ecological, evolutionary & environmental sciences

For a reference copy of the document with all sections, see nature.com/documents/nr-reporting-summary-flat.pdf

Life sciences study design

All studies must disclose on these points even when the disclosure is negative.

Sample size	Sample size was kept small as this study was considered as a proof-of-concept and required manual human evaluations that are exhaustive already with n=20.
Data exclusions	We did not exclude data from the analysis.
Replication	Data replication is challenging especially due to the usage of a proprietary AI model (GPT-4) to which users have limited access to and that given its guardrails avoiding potentially harmful content, sometimes refuses in answering adequately to medical queries. In these cases we re-ran each sample in a newly instantiated setting until no refusals occured. Additionally, reproducibility might be affected by silent changes that are made to the model and not disclosed to the public by the owners of the model (OpenAI).
Randomization	There was only one evaluation group (AI agent), so we could not perform randomization.
Blinding	Human evaluators performed the evaluations independently. Rating model answers was not blinded, as there was no control group. However, humans were blinded to the models answer when definining the ground truth for required Tool Use and completeness of the model responses.

Reporting for specific materials, systems and methods

We require information from authors about some types of materials, experimental systems and methods used in many studies. Here, indicate whether each material, system or method listed is relevant to your study. If you are not sure if a list item applies to your research, read the appropriate section before selecting a response.

Materials & experimental systems

n/a	Involved in the study
☒	☐ Antibodies
☒	☐ Eukaryotic cell lines
☒	☐ Palaeontology and archaeology
☒	☐ Animals and other organisms
☒	☐ Clinical data
☒	☐ Dual use research of concern
☒	☐ Plants

Methods

n/a	Involved in the study
☒	☐ ChIP-seq
☒	☐ Flow cytometry
☒	☐ MRI-based neuroimaging

Plants

Seed stocks

Report on the source of all seed stocks or other plant material used. If applicable, state the seed stock centre and catalogue number. If plant specimens were collected from the field, describe the collection location, date and sampling procedures.

Novel plant genotypes

Describe the methods by which all novel plant genotypes were produced. This includes those generated by transgenic approaches, gene editing, chemical/radiation-based mutagenesis and hybridization. For transgenic lines, describe the transformation method, the number of independent lines analyzed and the generation upon which experiments were performed. For gene-edited lines, describe the editor used, the endogenous sequence targeted for editing, the targeting guide RNA sequence (if applicable) and how the editor was applied.

Authentication

Describe any authentication procedures for each seed stock used or novel genotype generated. Describe any experiments used to assess the effect of a mutation and, where applicable, how potential secondary effects (e.g. second site T-DNA insertions, mosiacism, off-target gene editing) were examined.
