# Supplementary figures and images for: Development and validation of an autonomous artificial intelligence agent for clinical decision-making in oncology
Source: Nat Cancer. 2025 Jun 6;6(8):1337–49. doi: 10.1038/s43018-025-00991-6 (PMC12380607; doi:10.1038/s43018-025-00991-6)

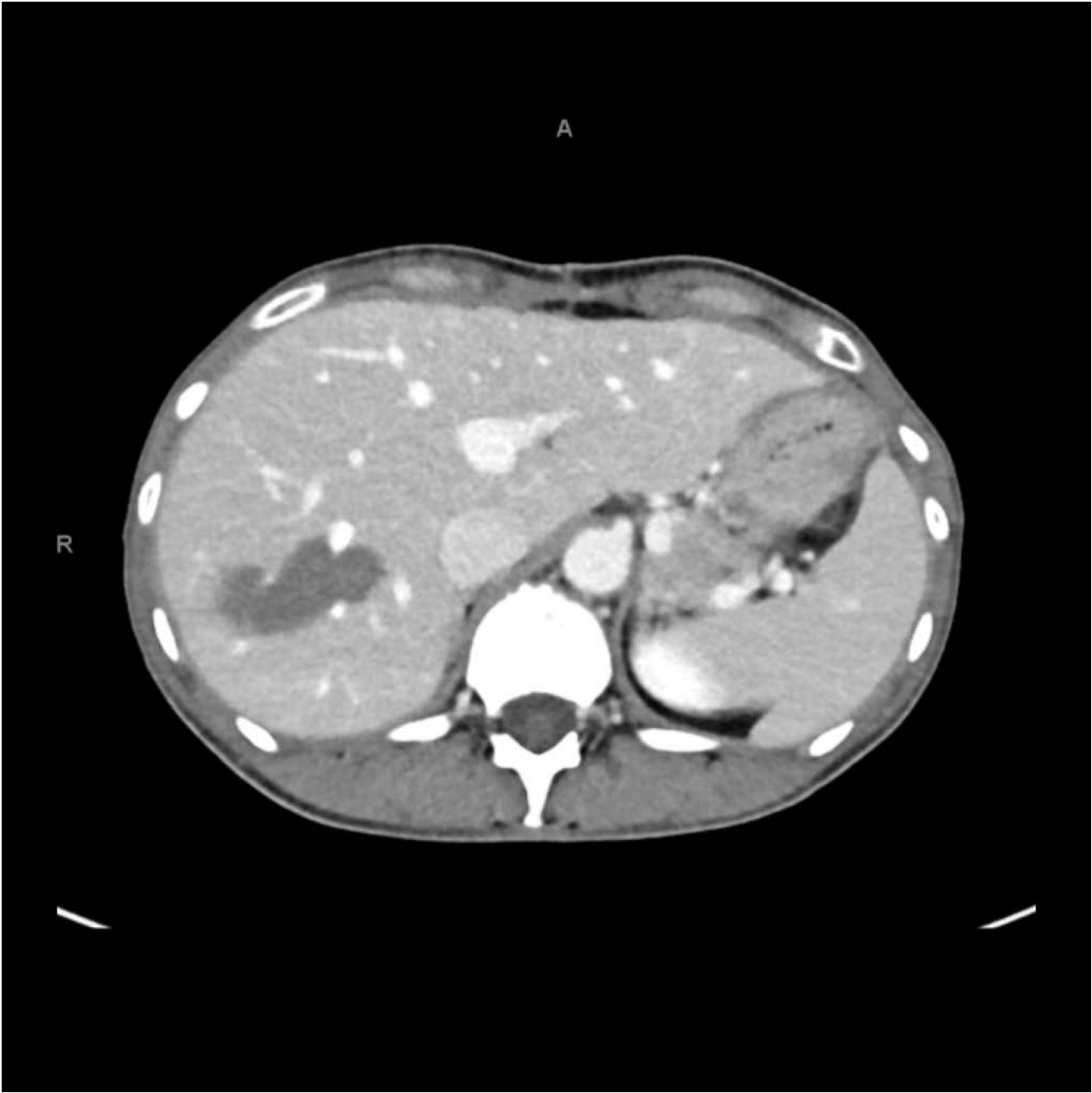

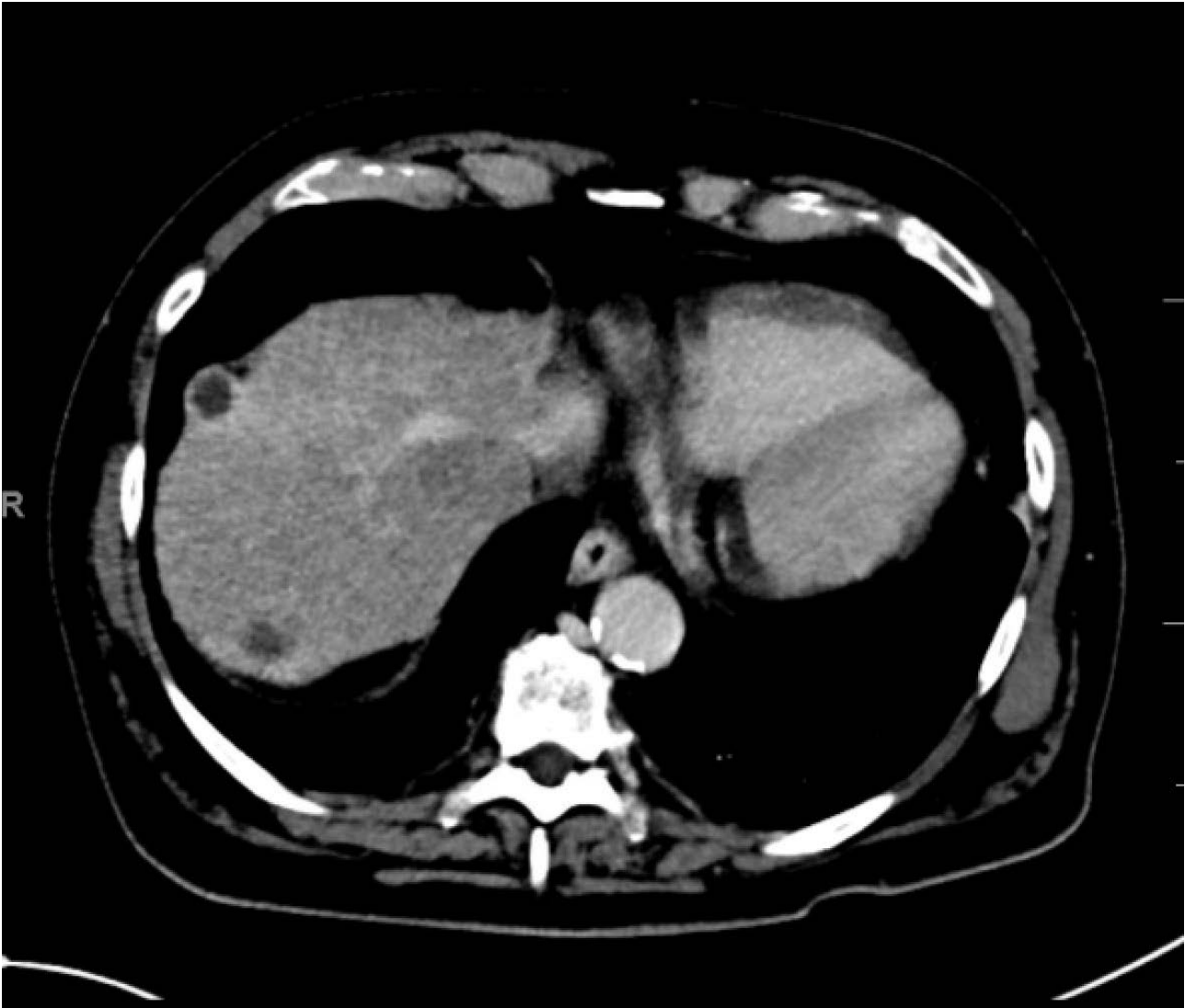

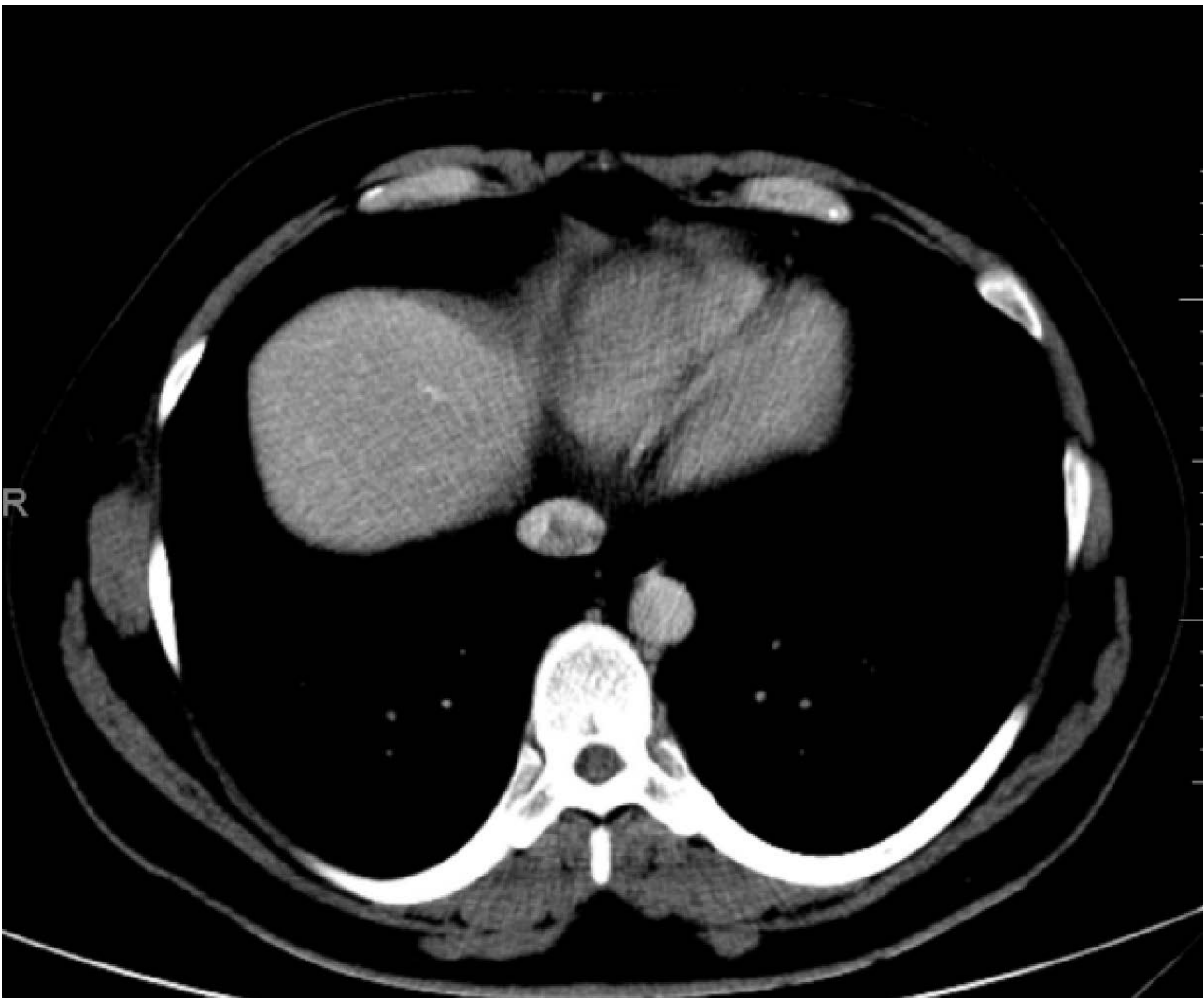

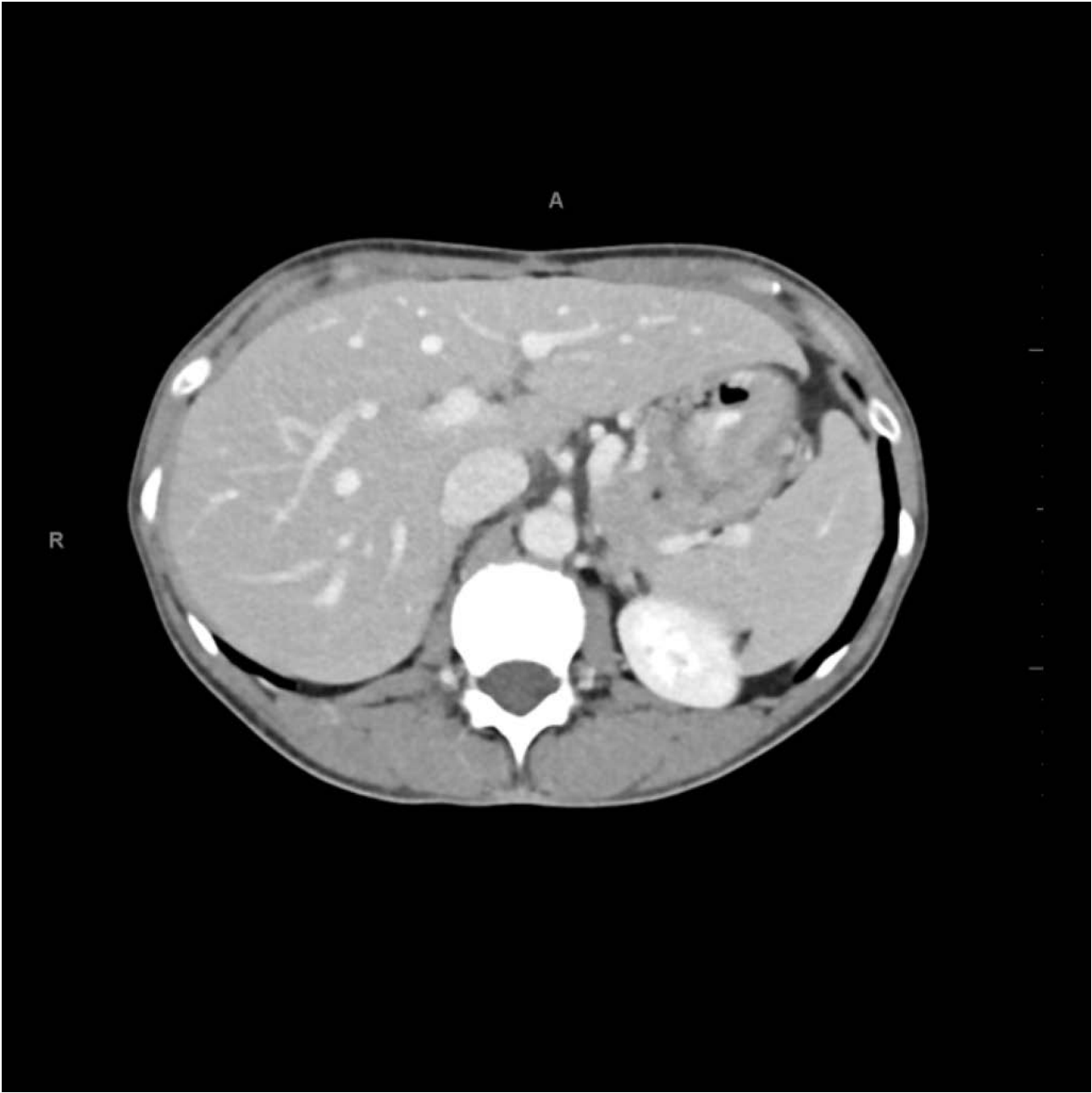

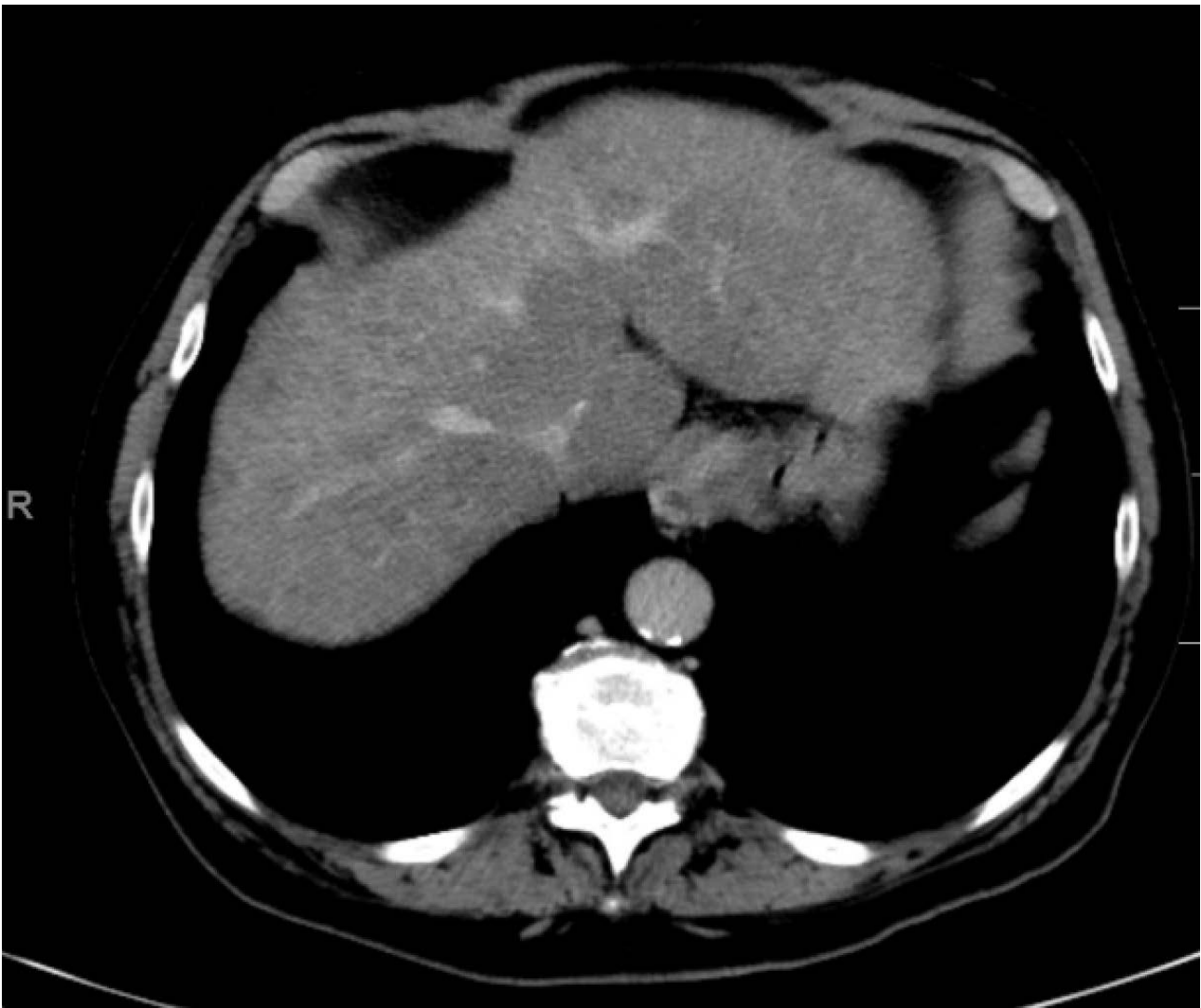

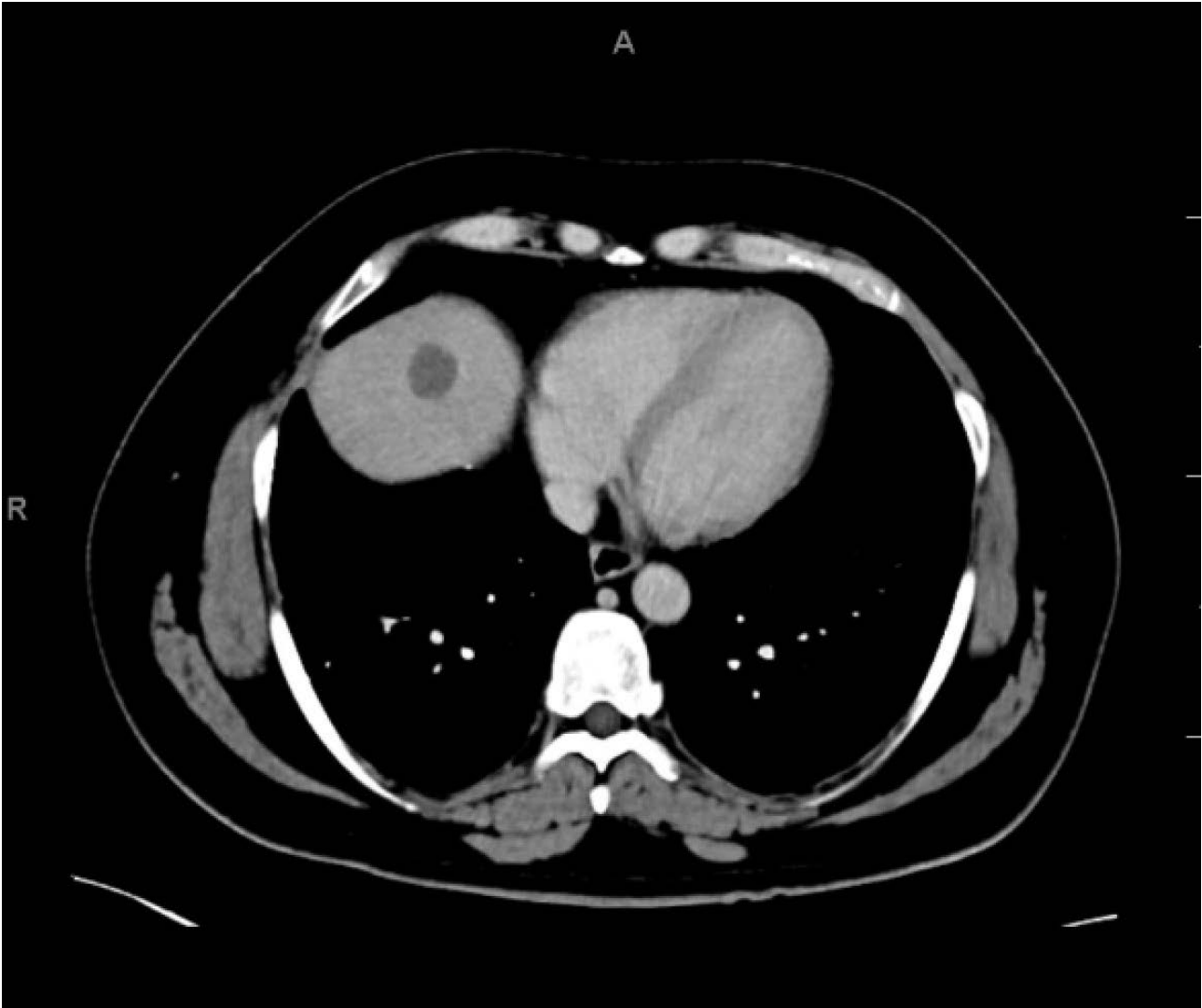

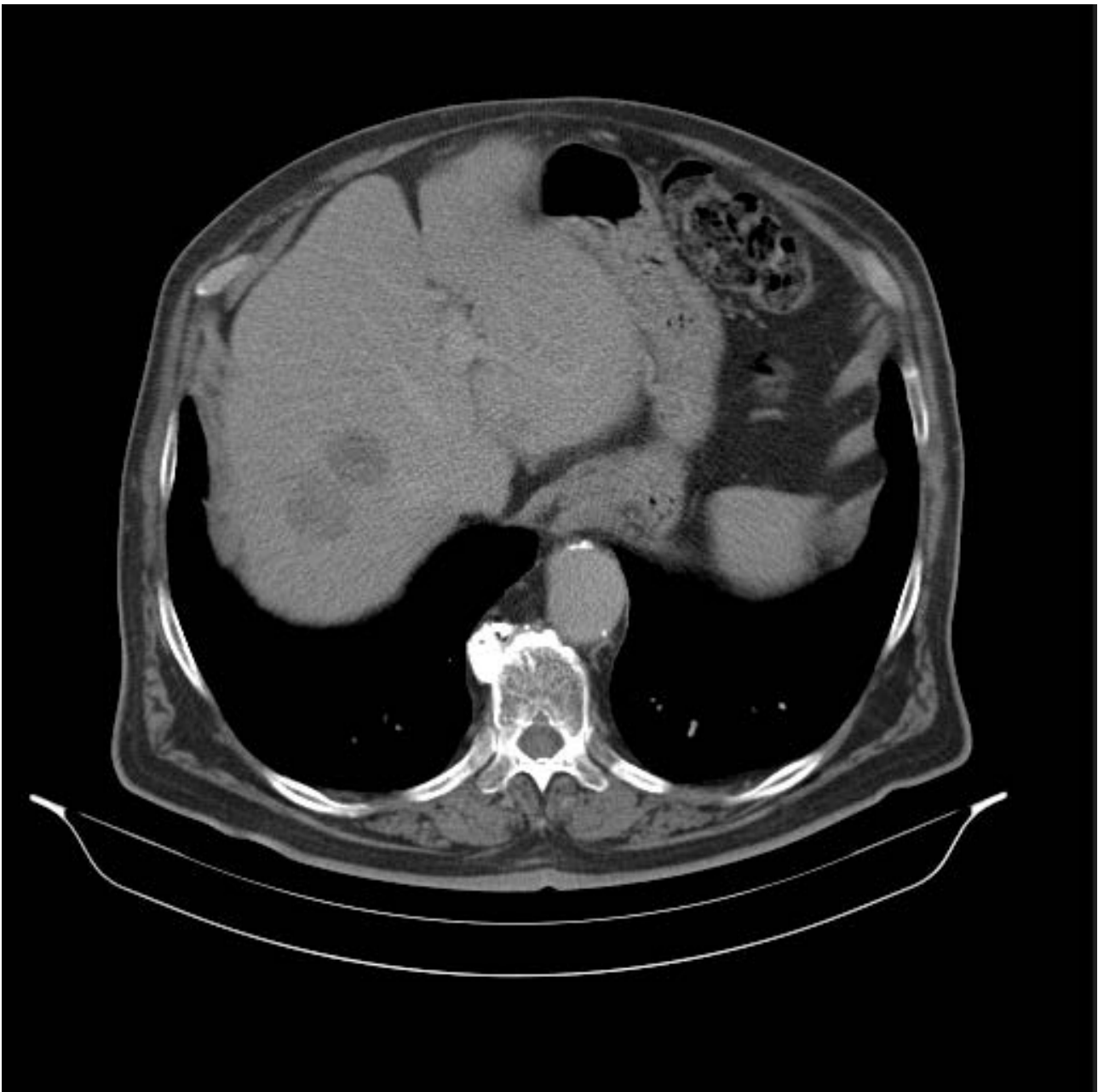

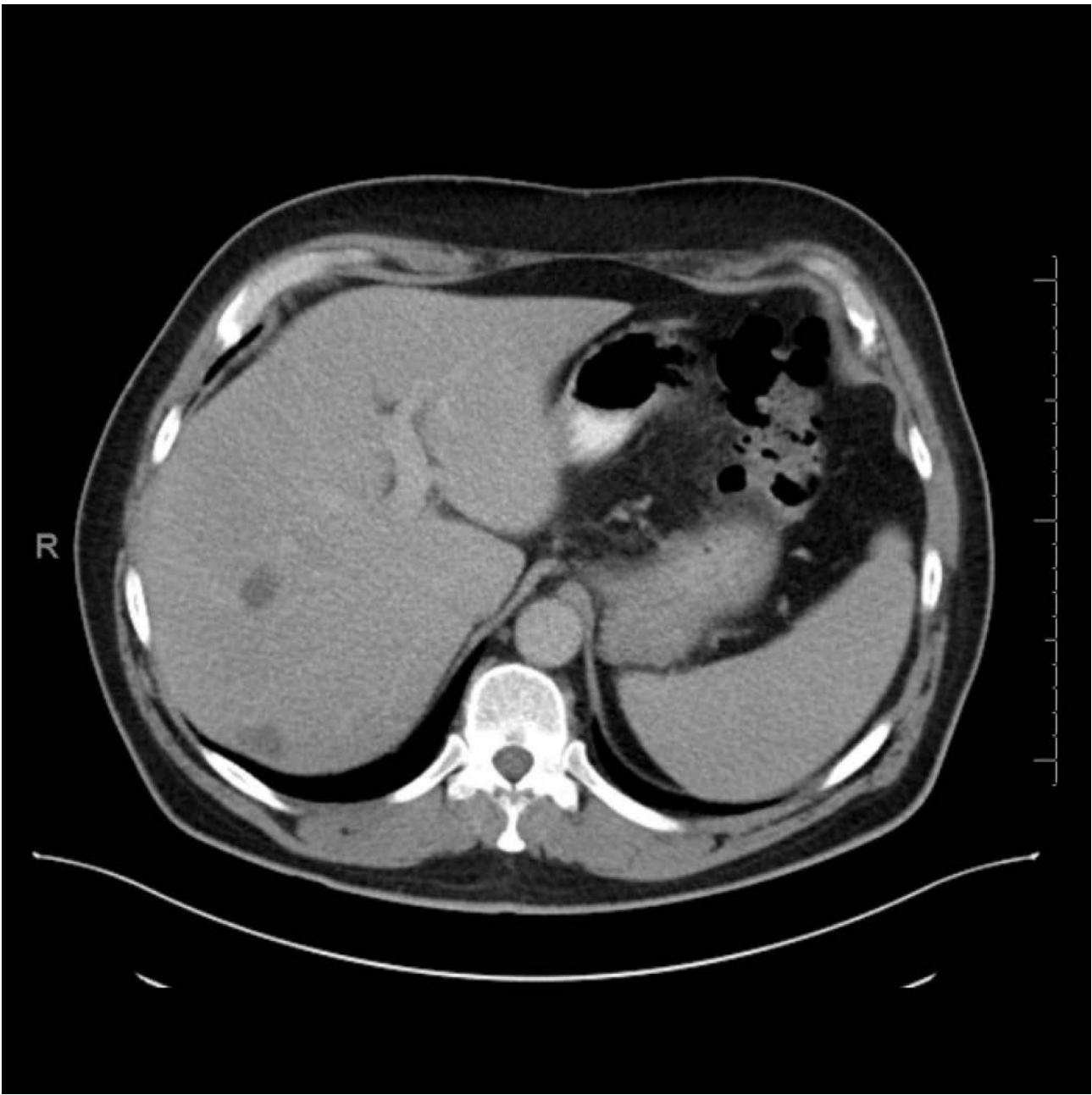

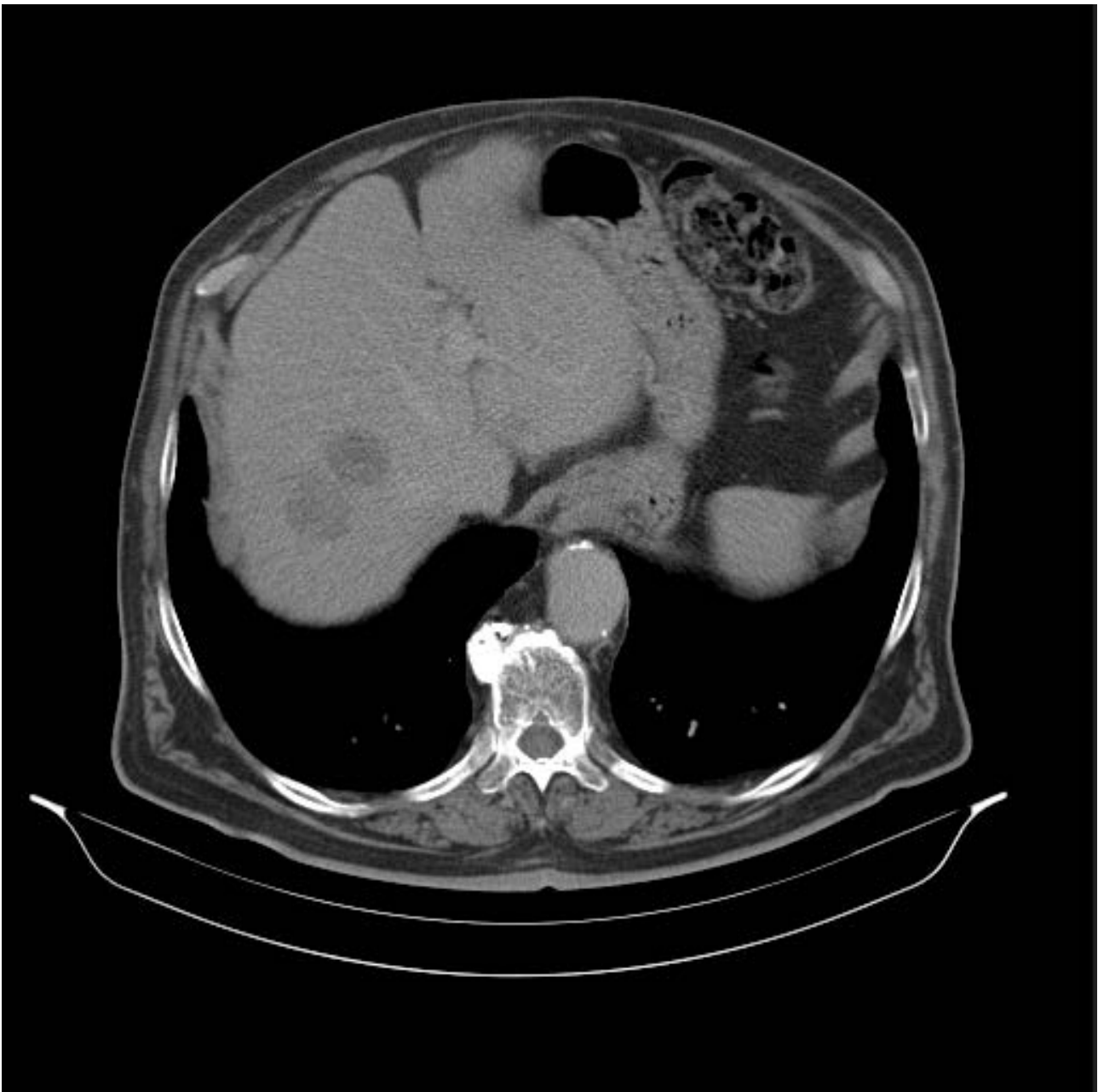

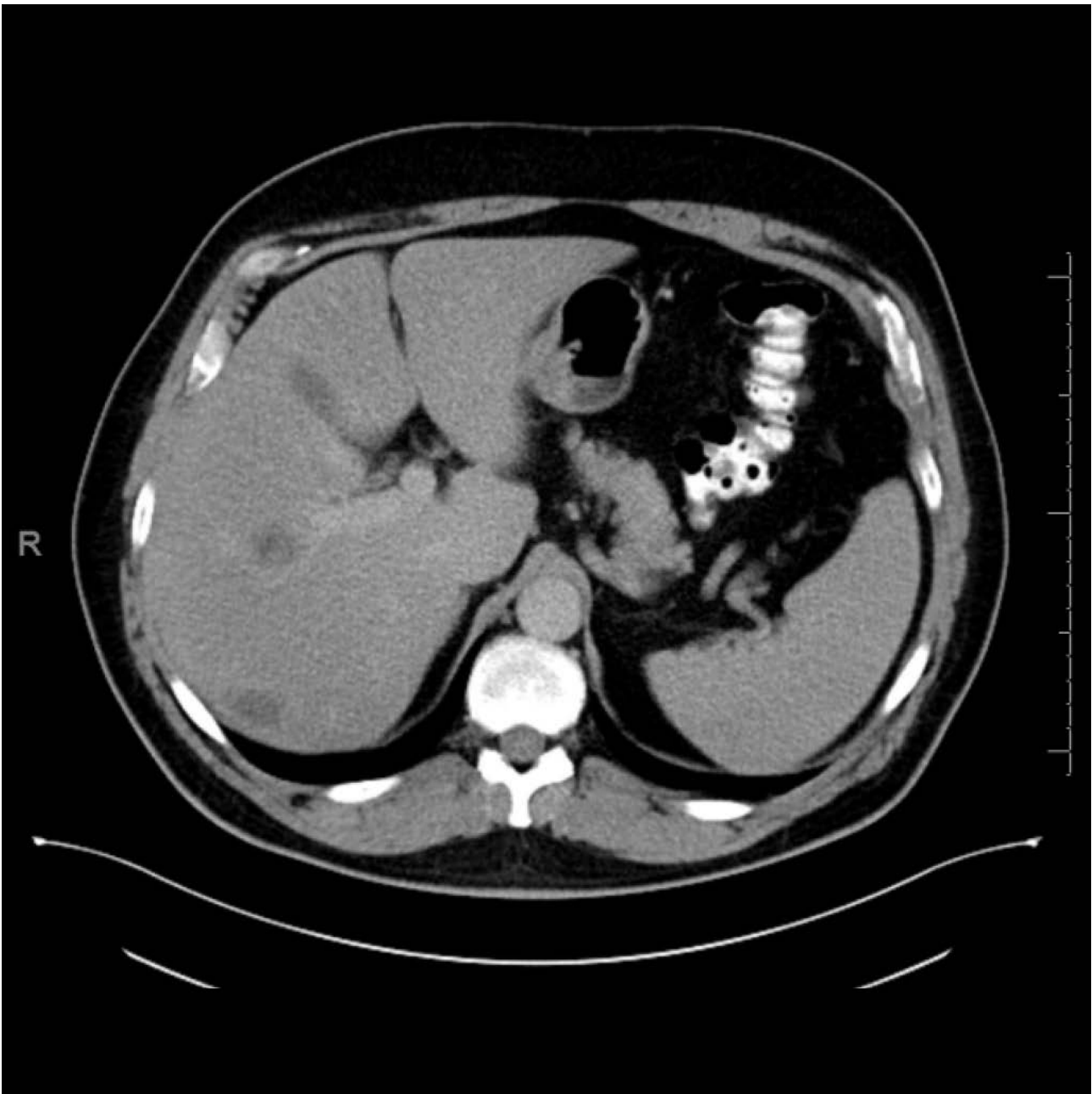

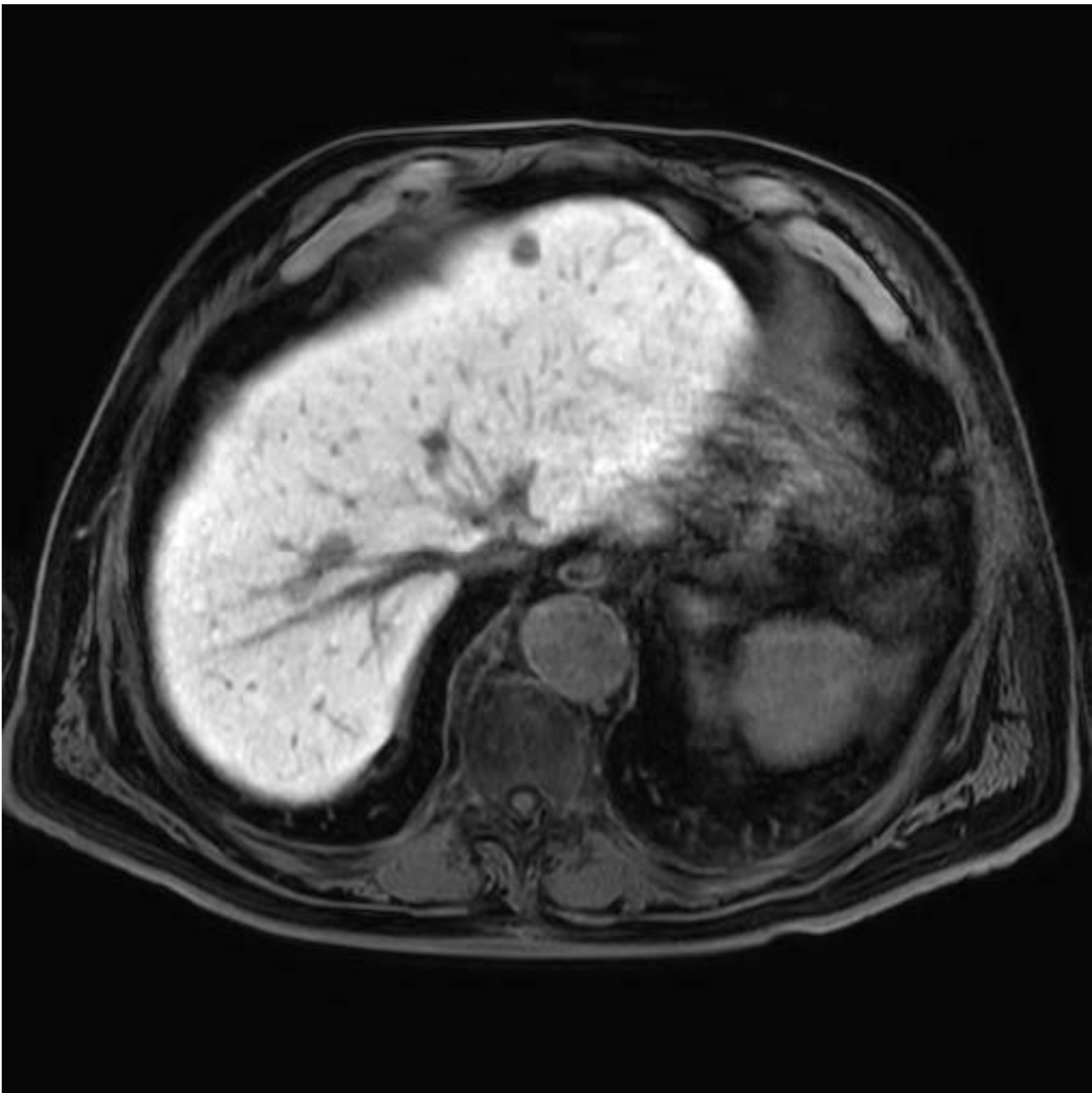

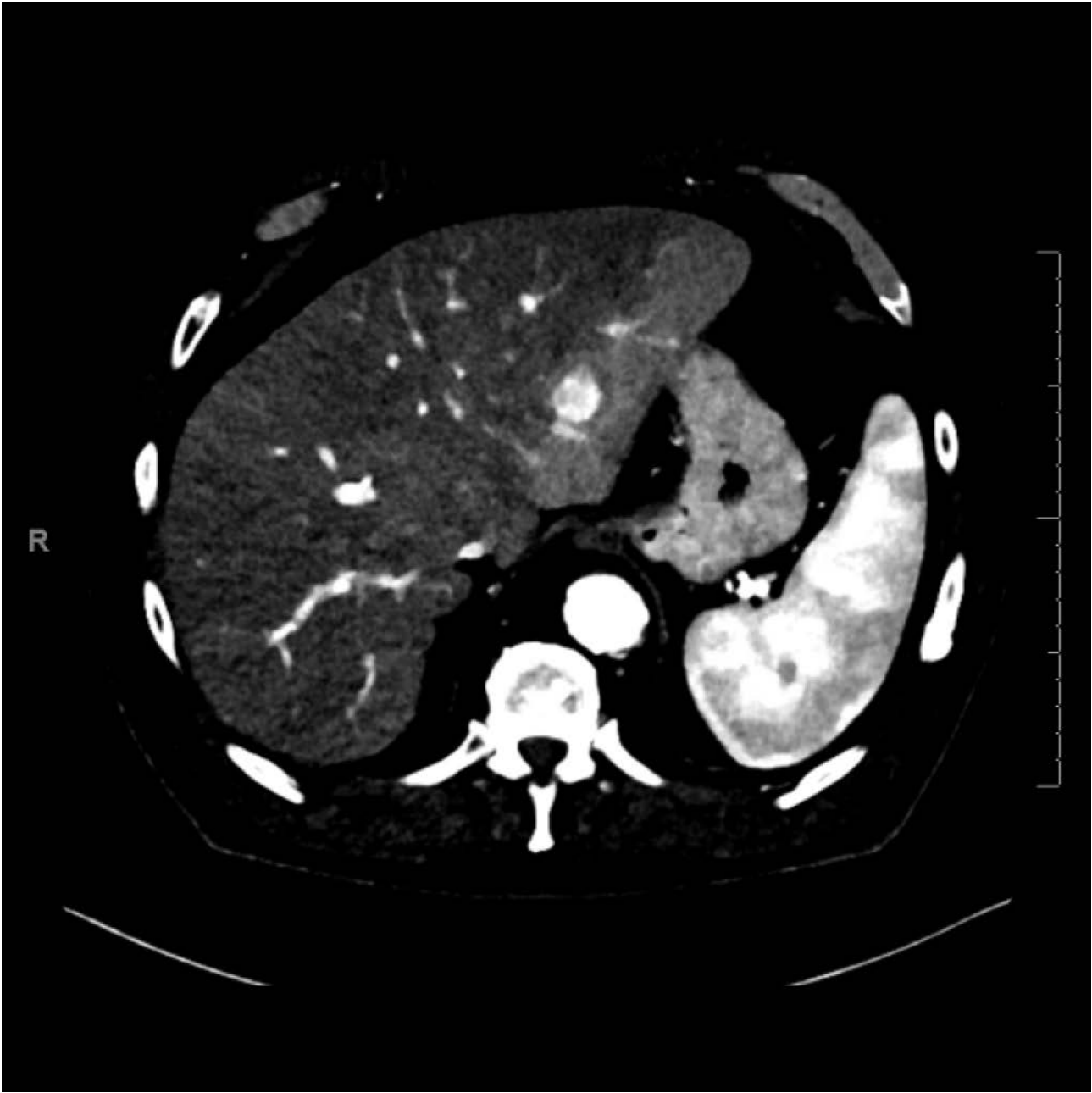

Smith\_2.jpg

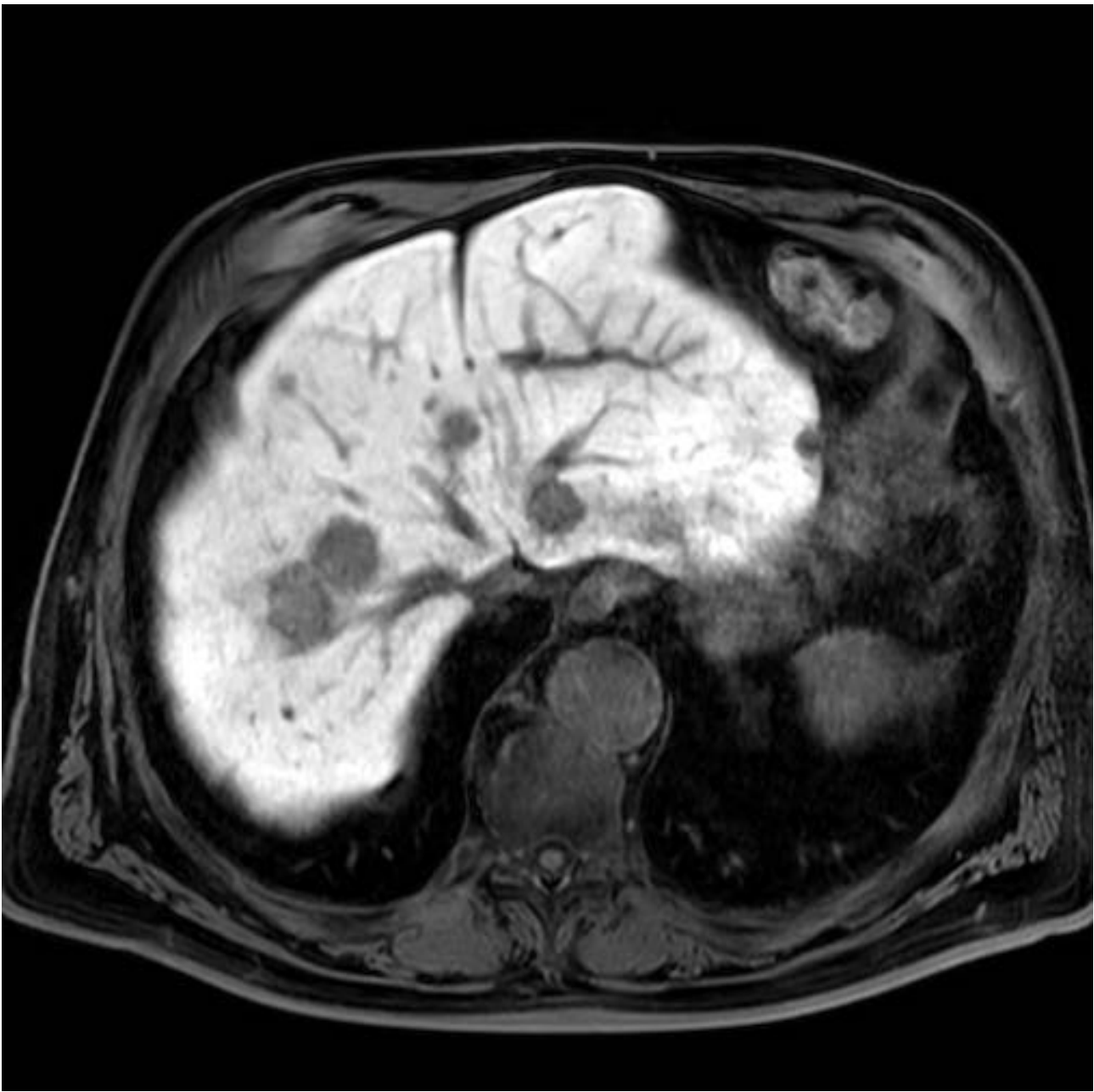

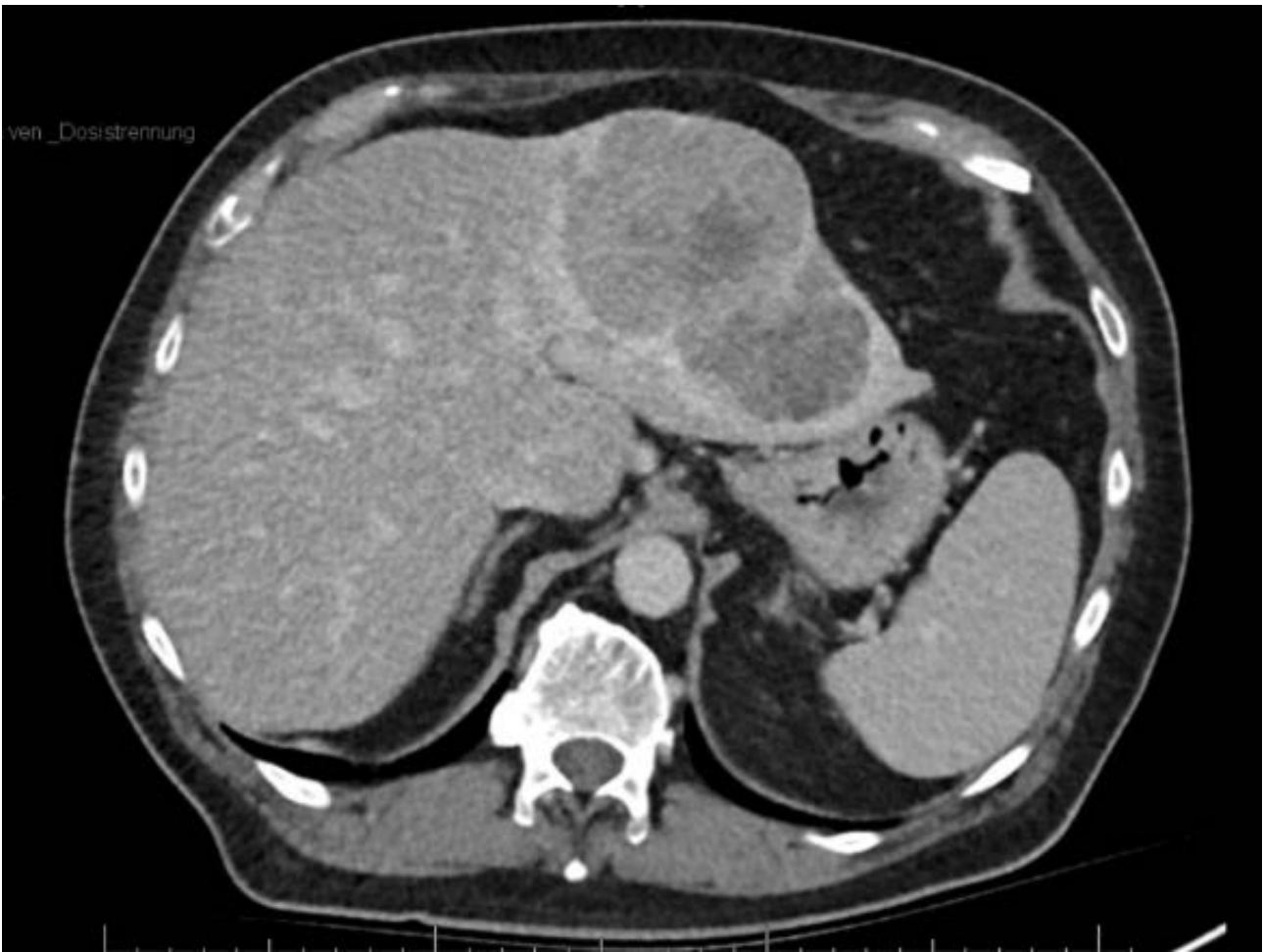

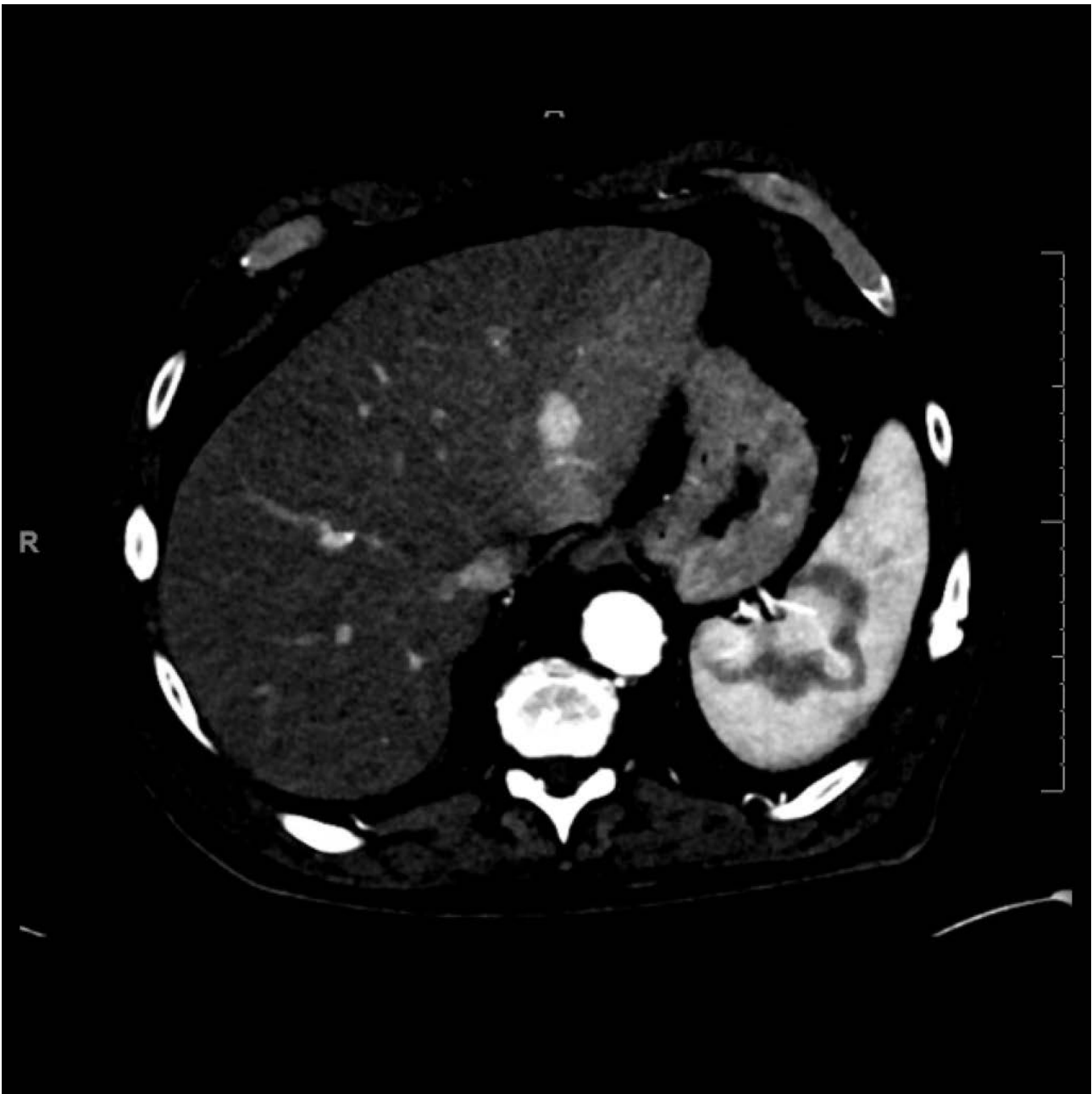

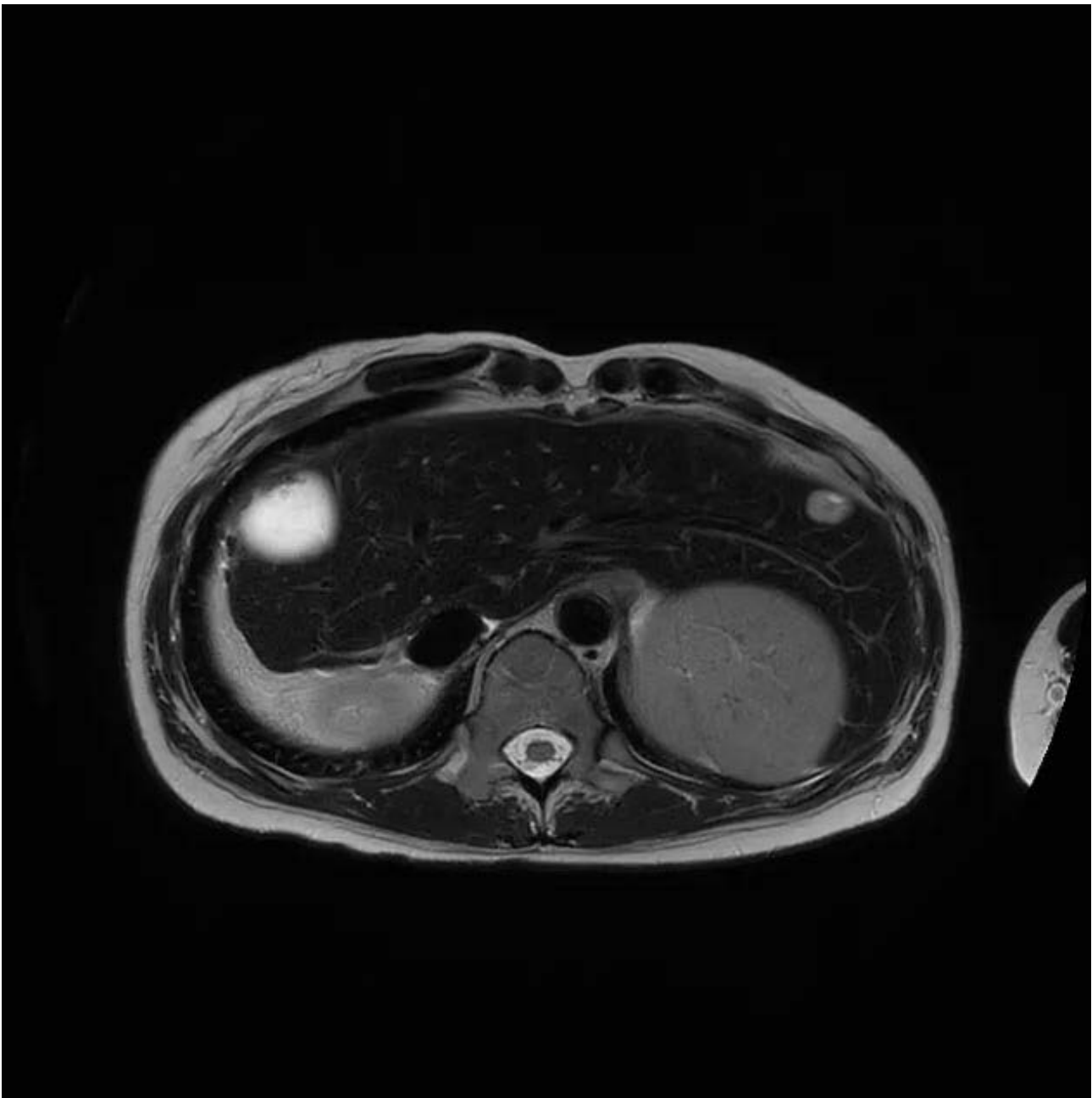

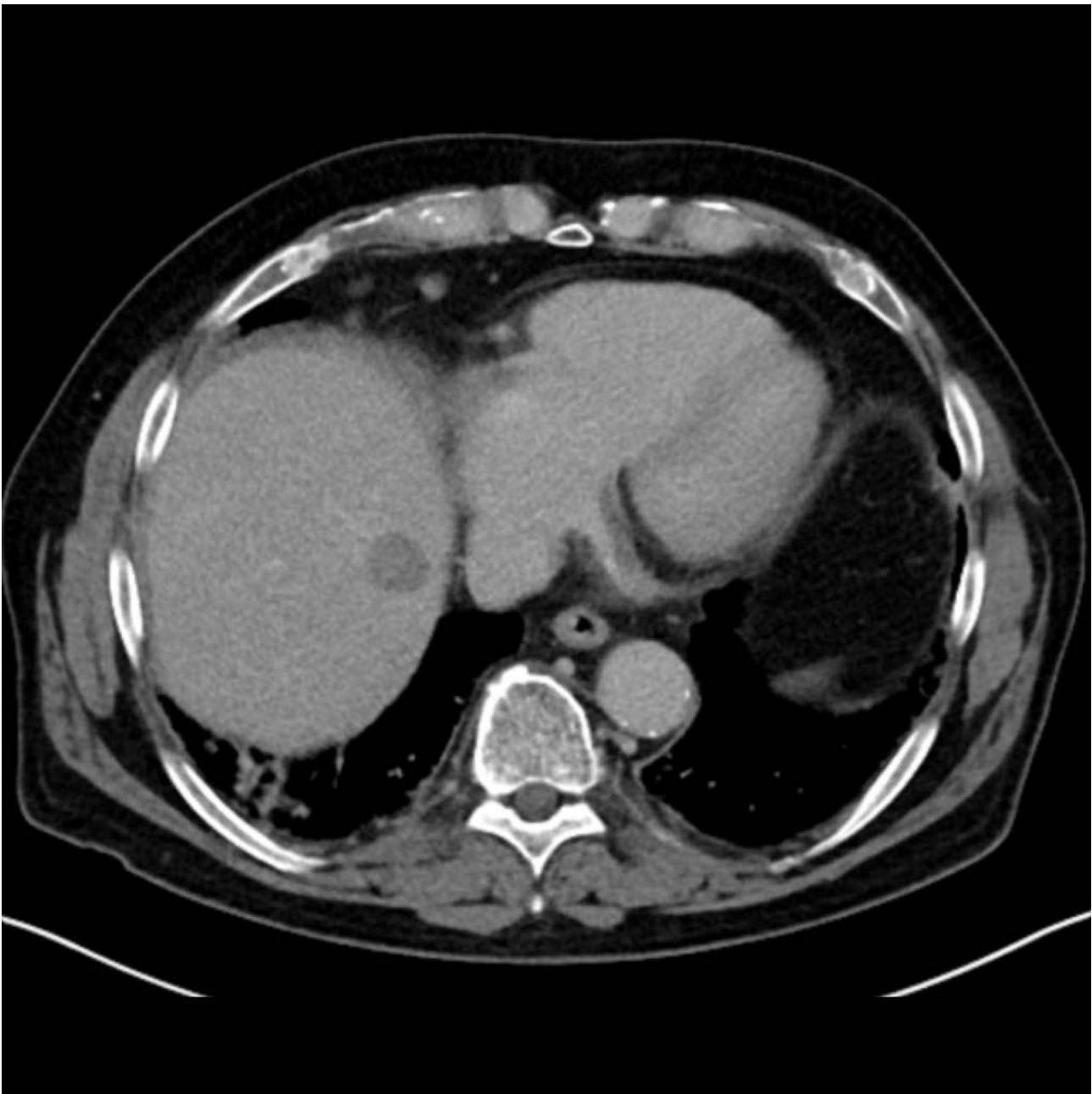

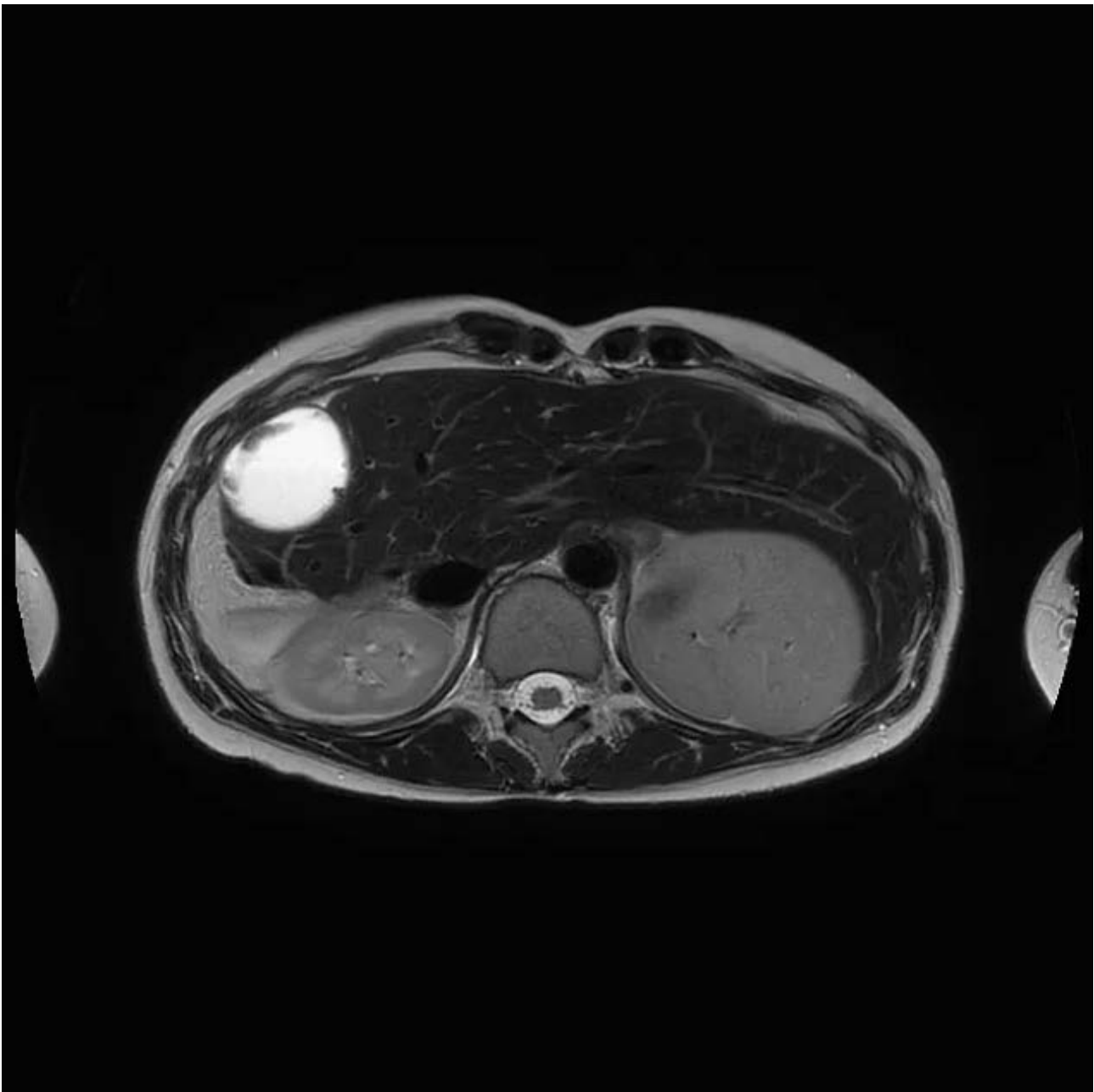

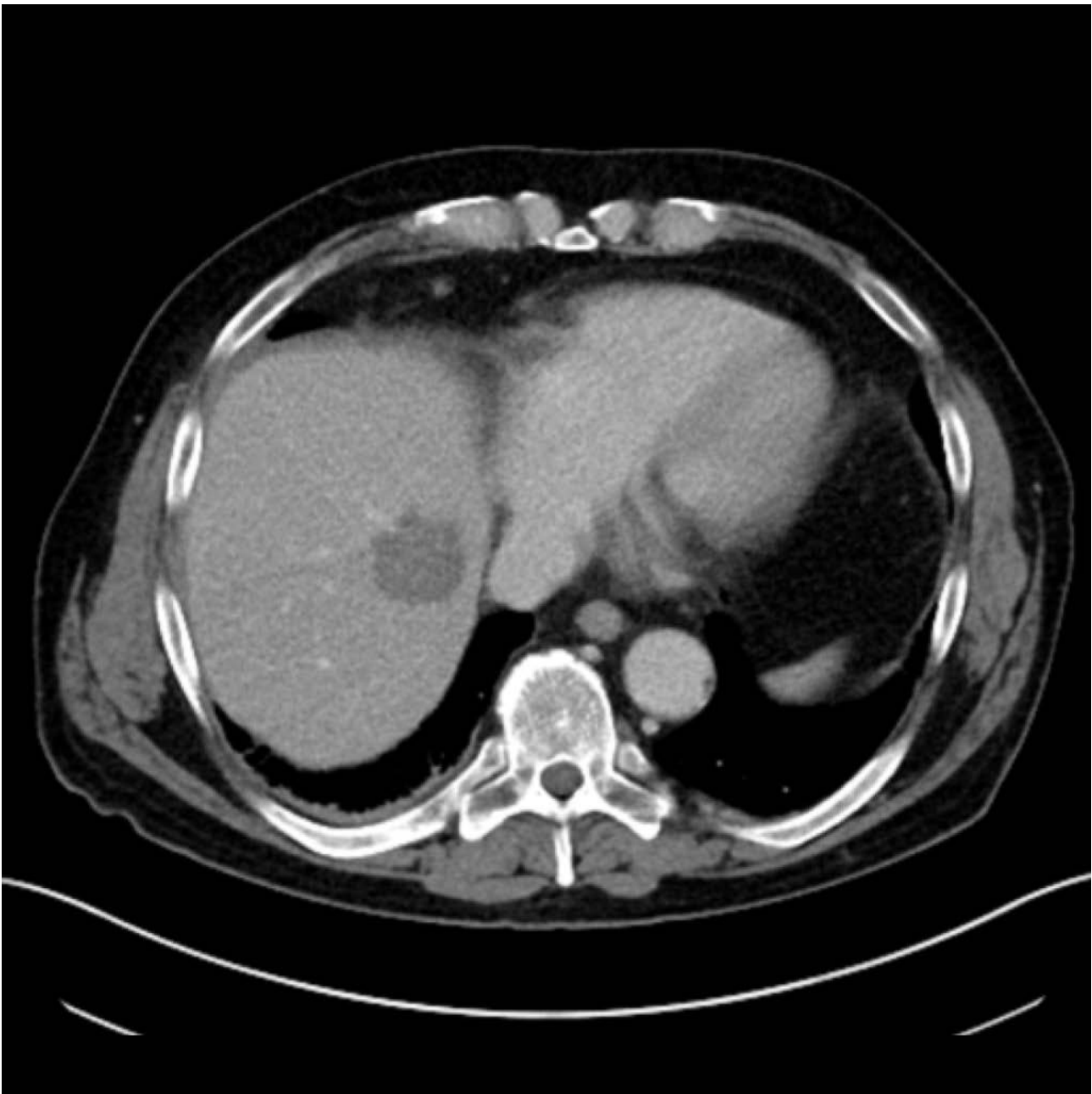

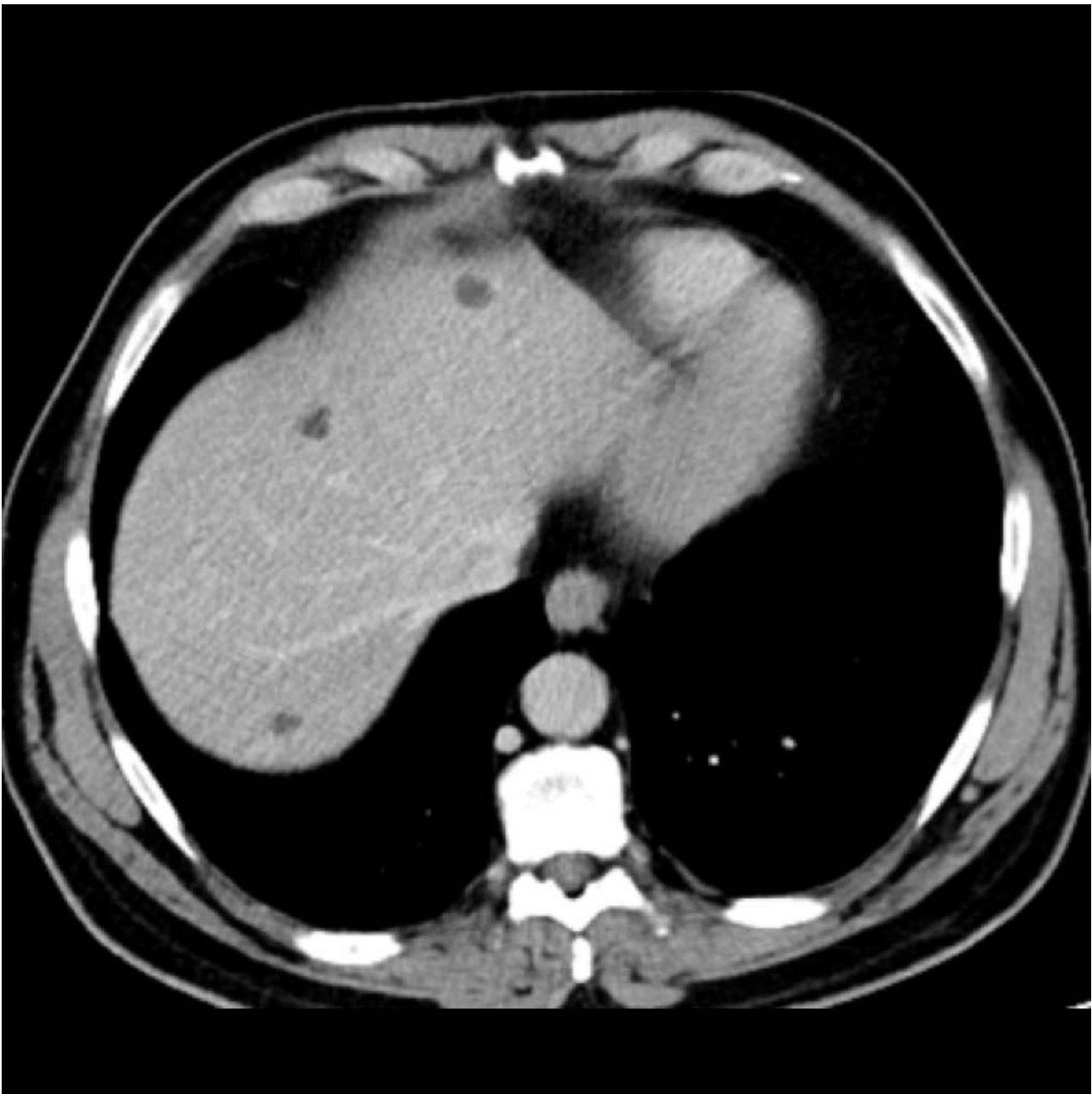

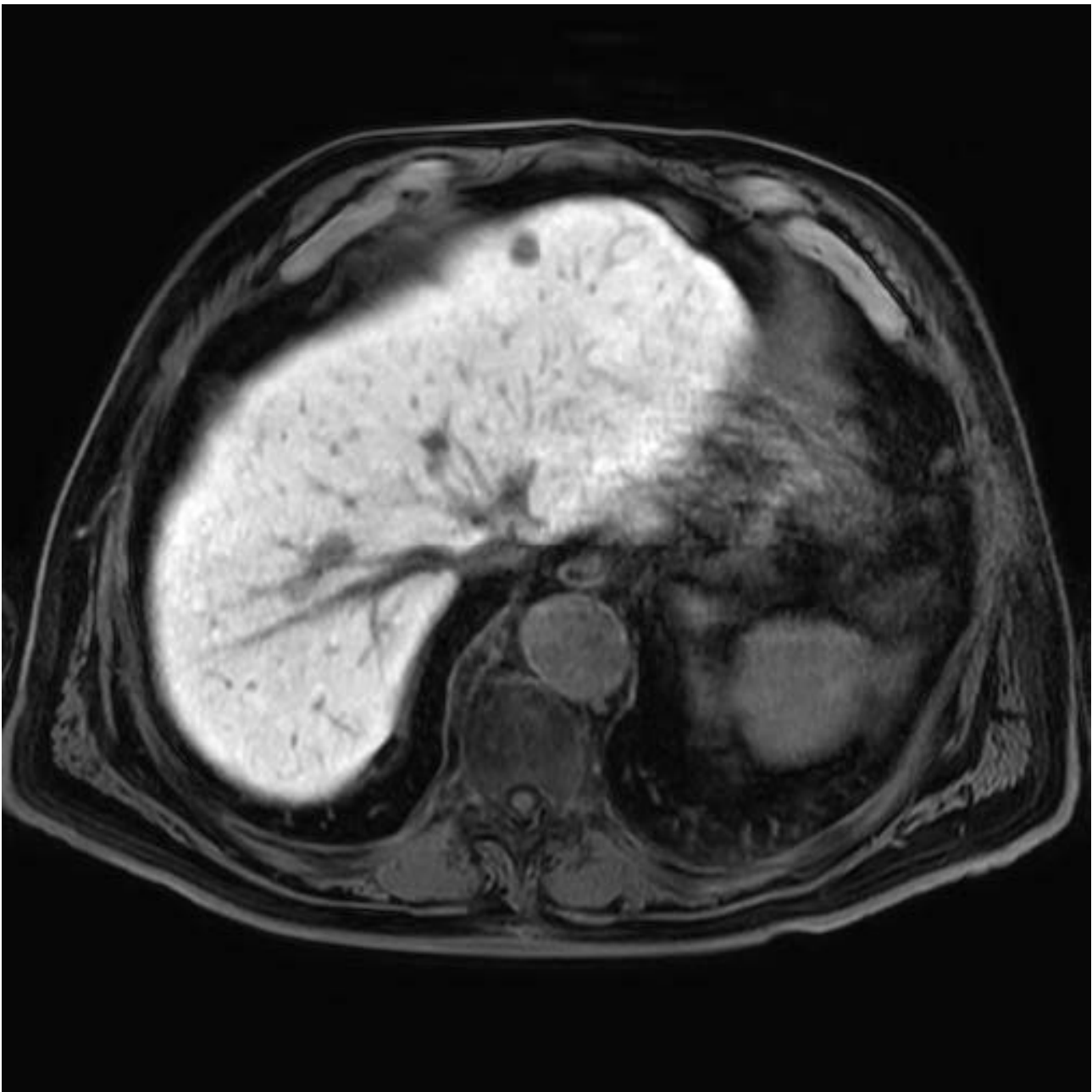

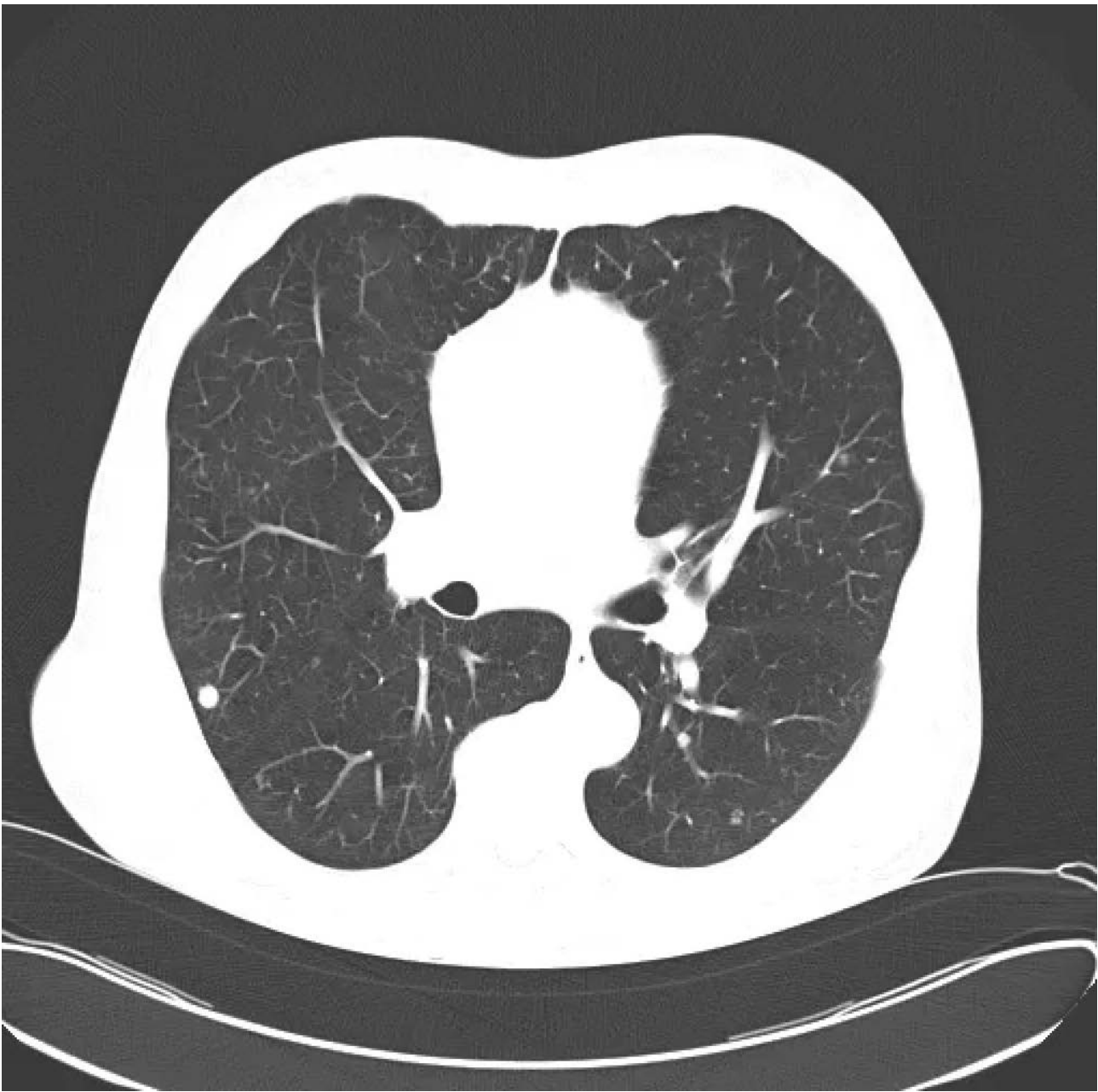

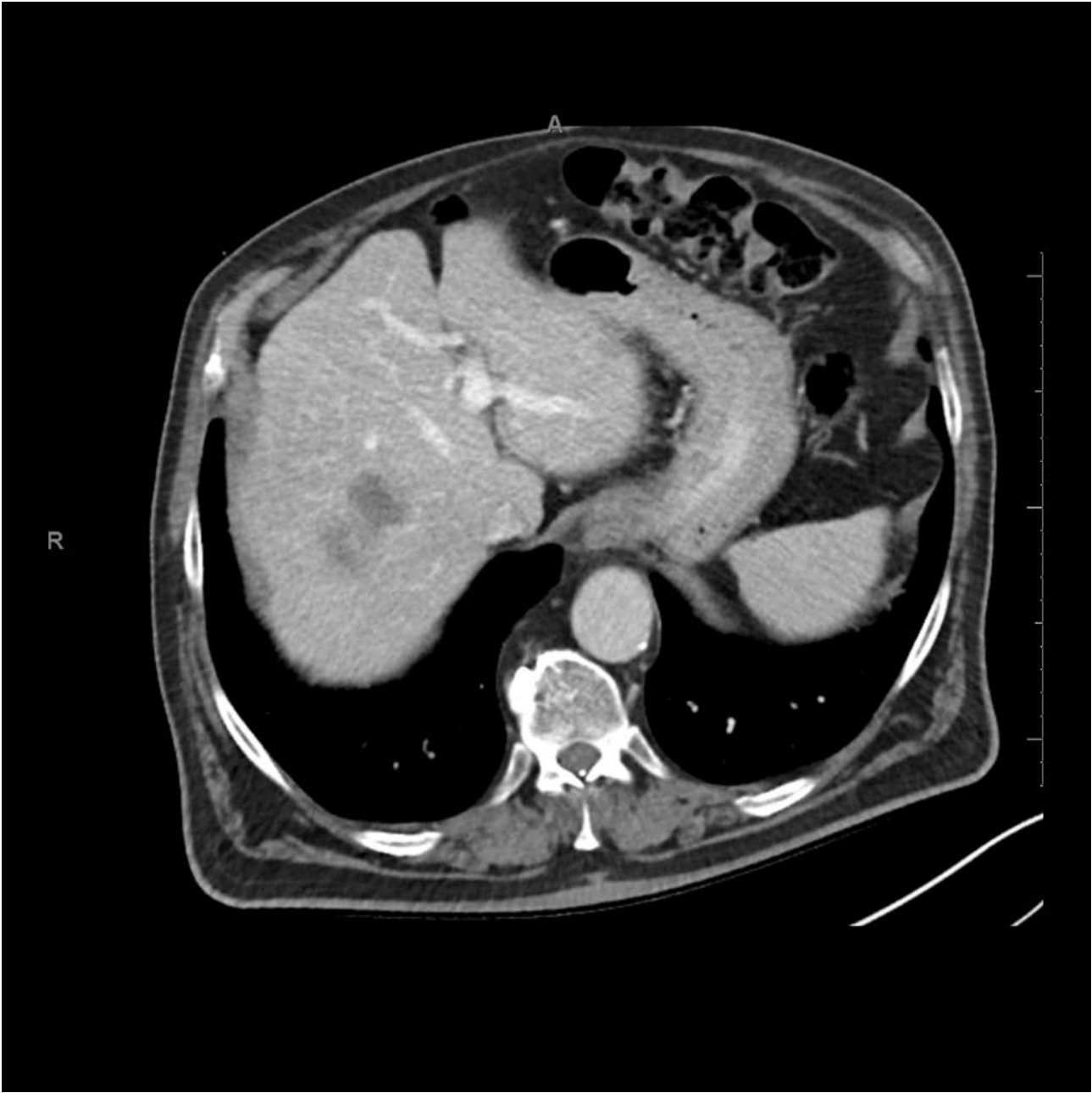

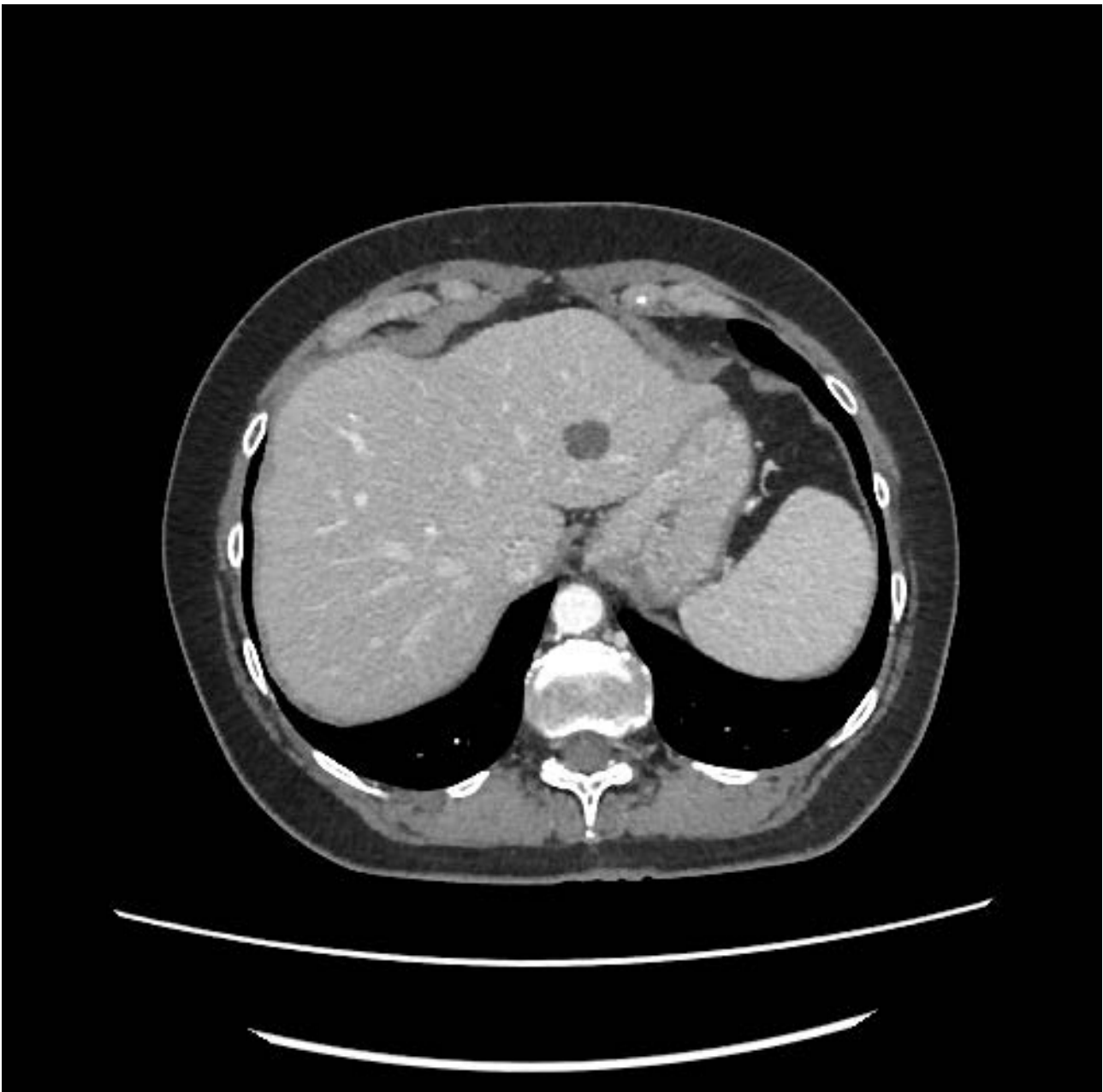

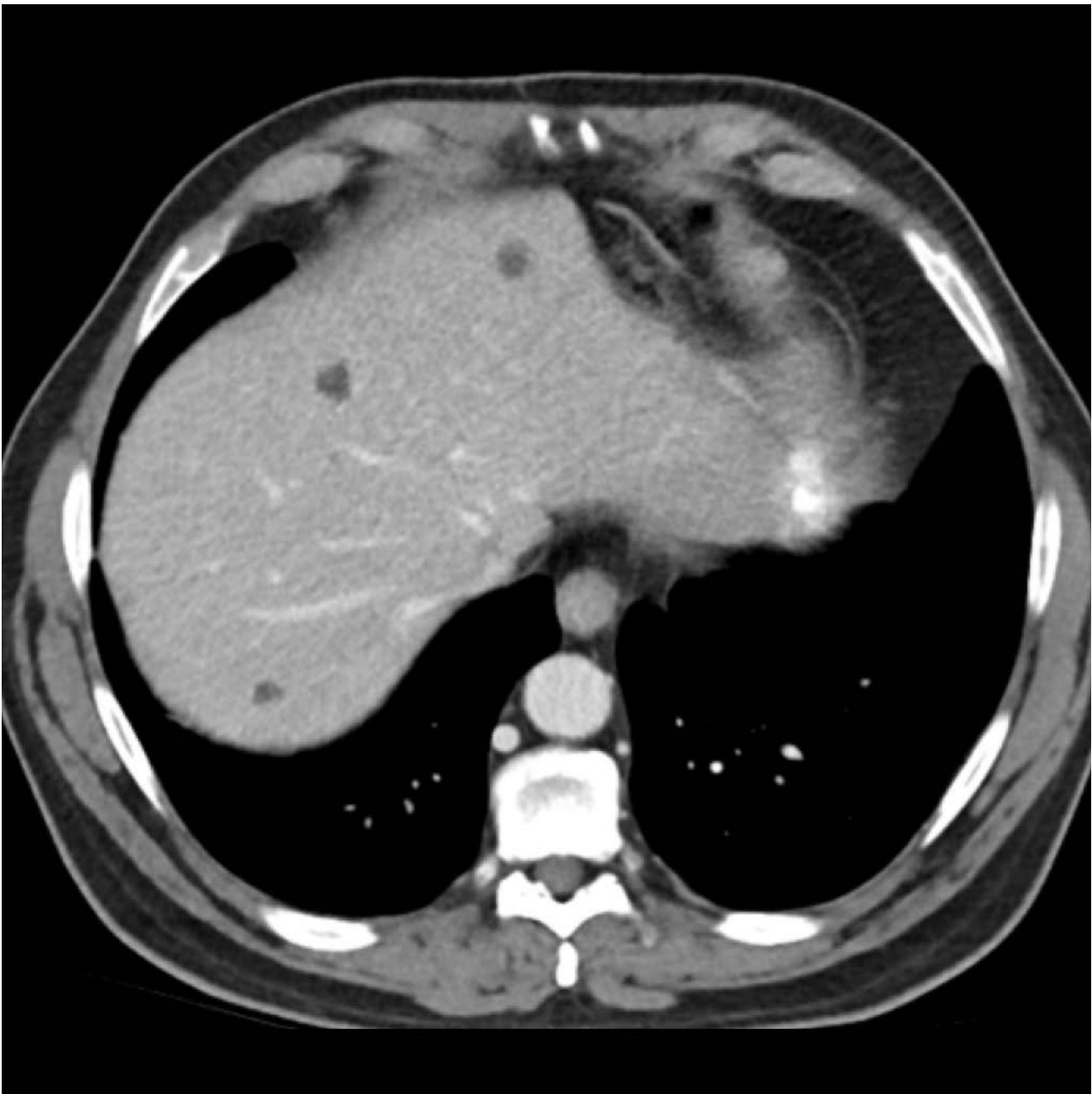

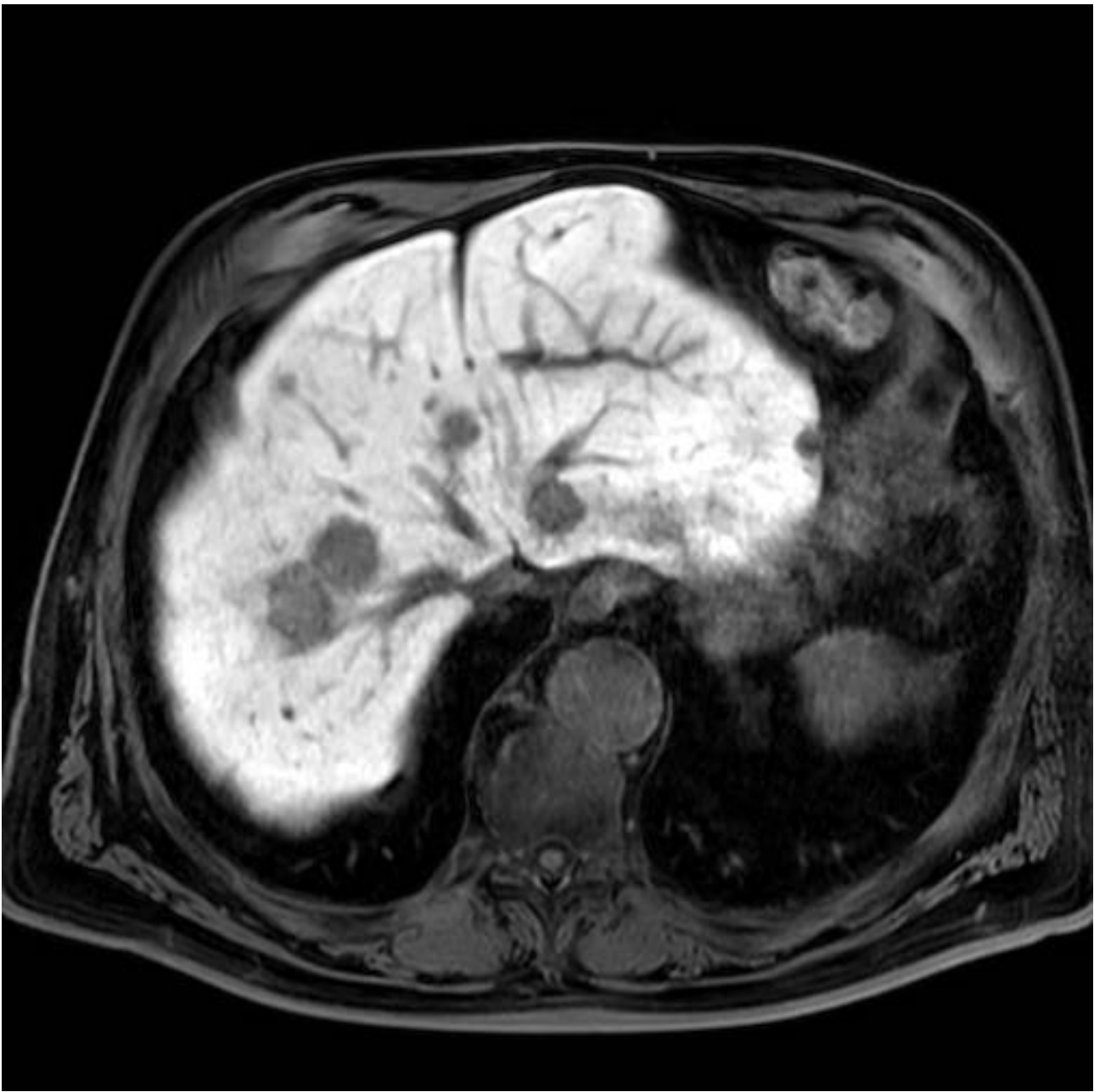

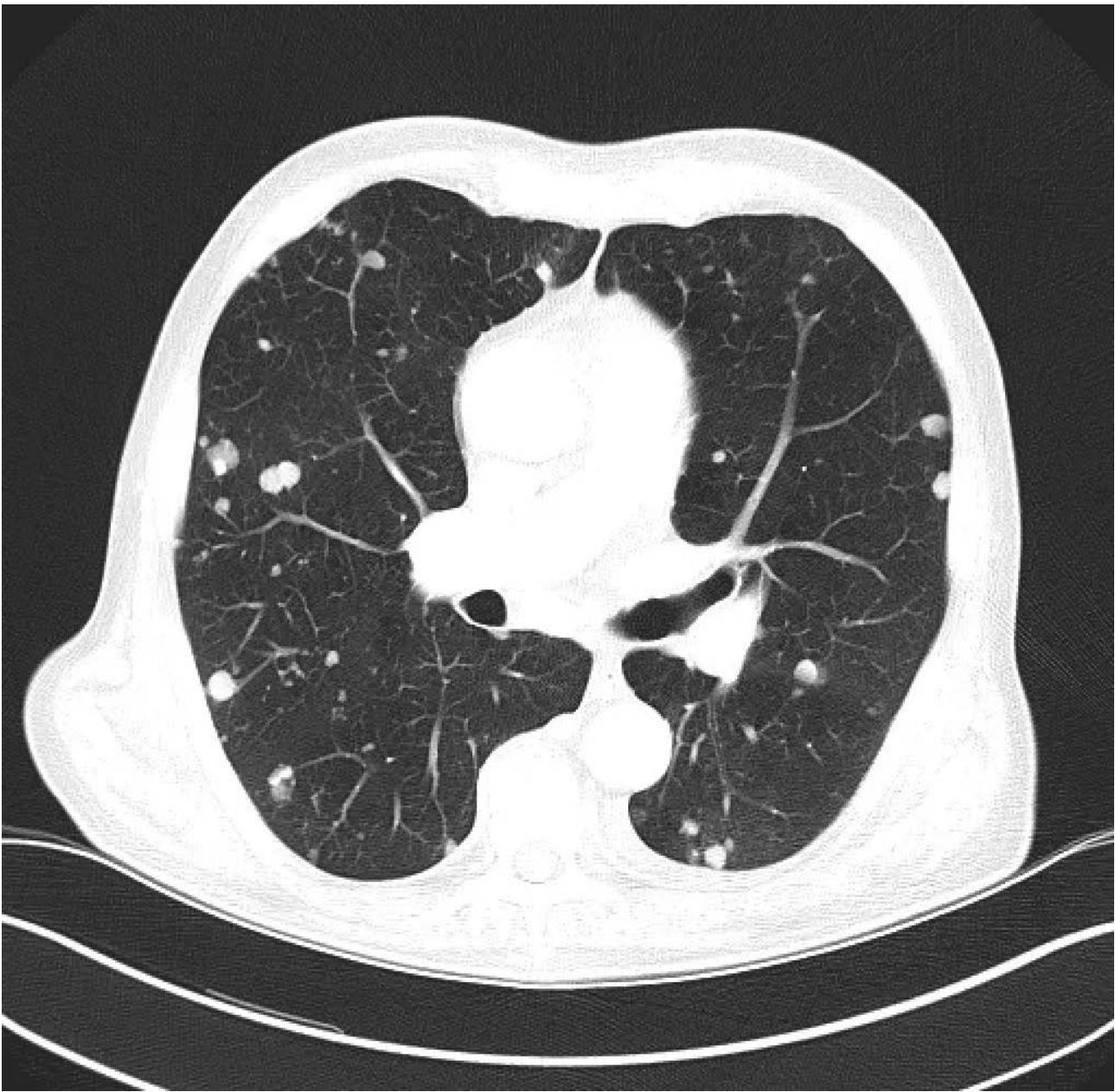

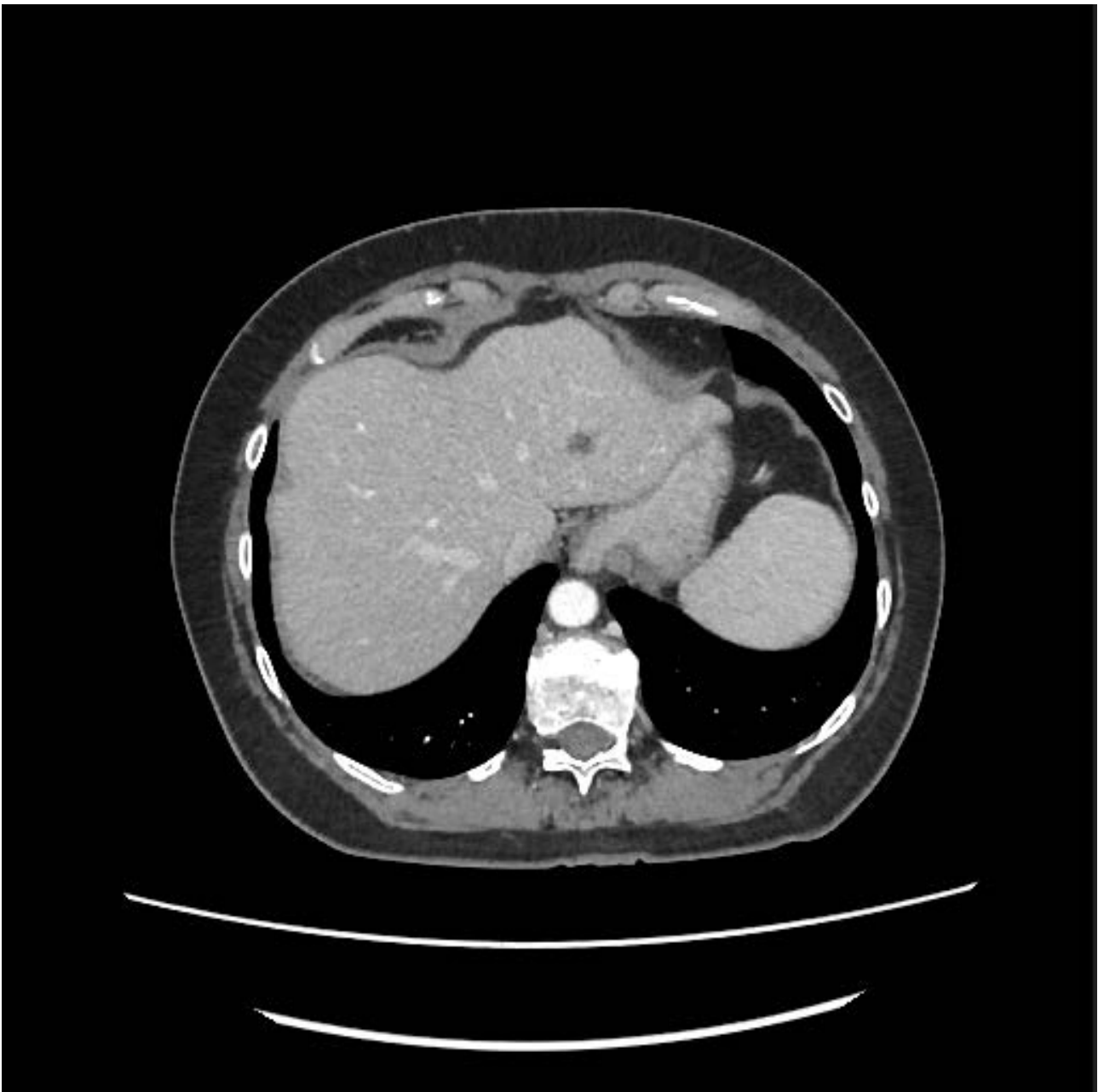

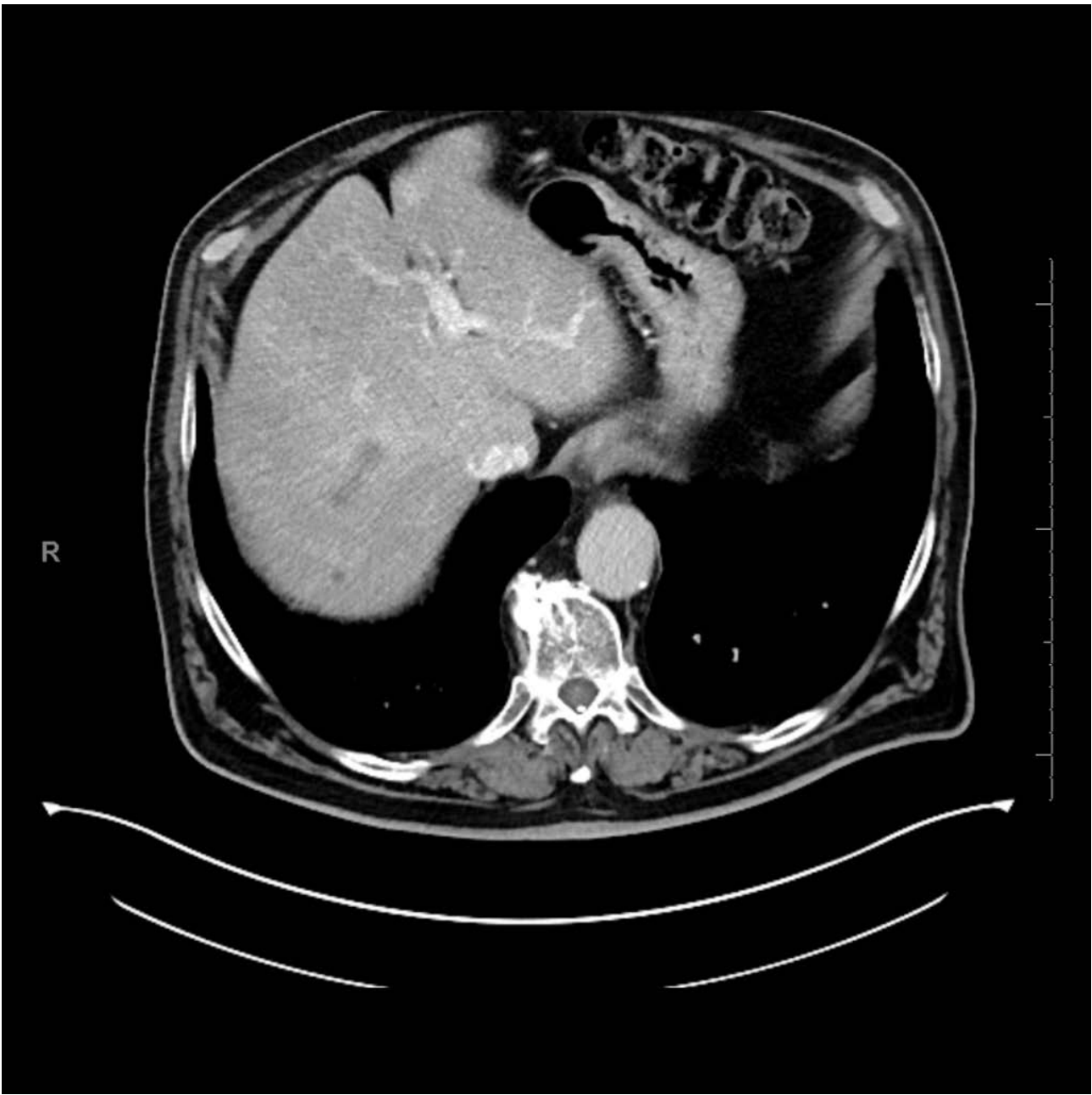

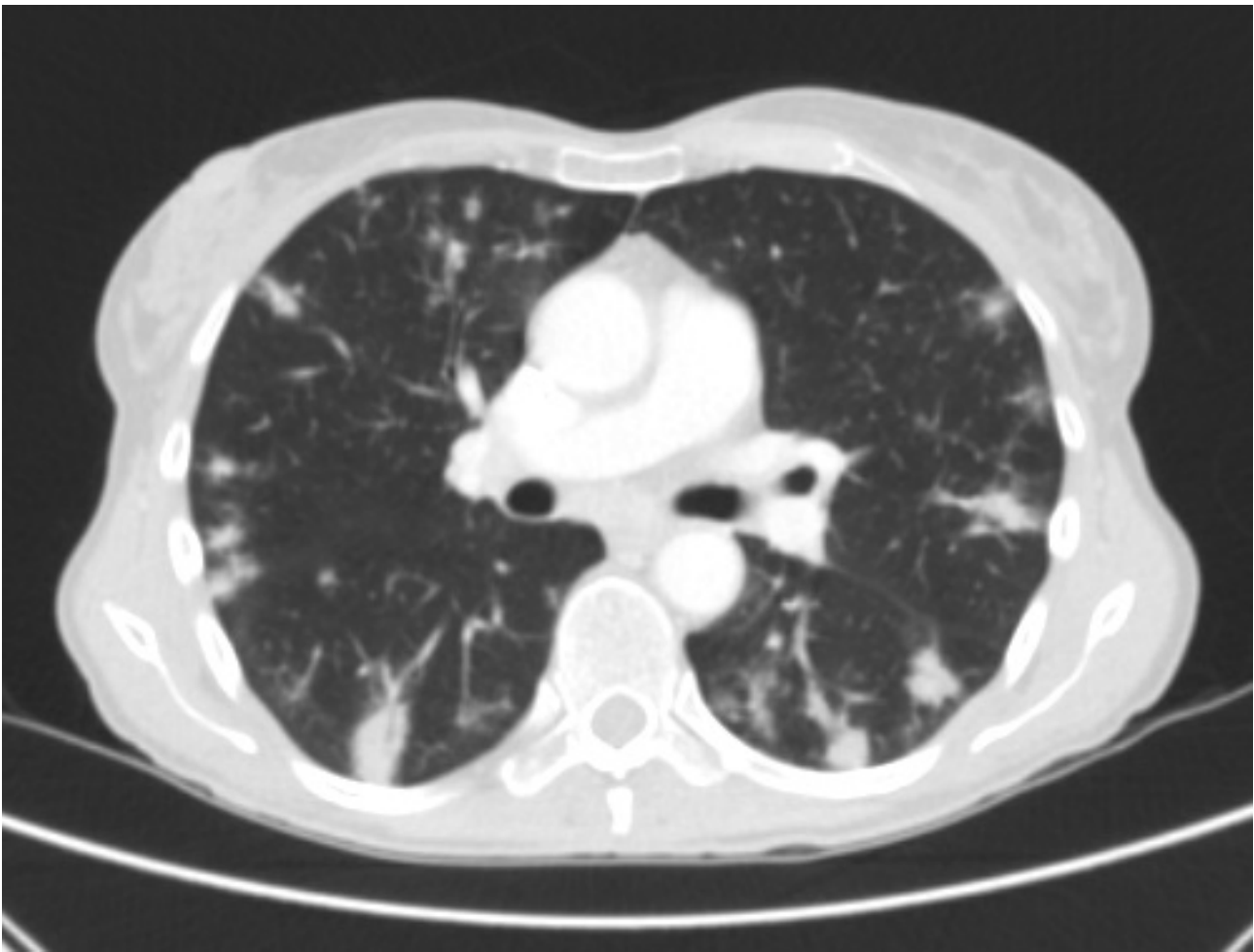

Lopez.jpg

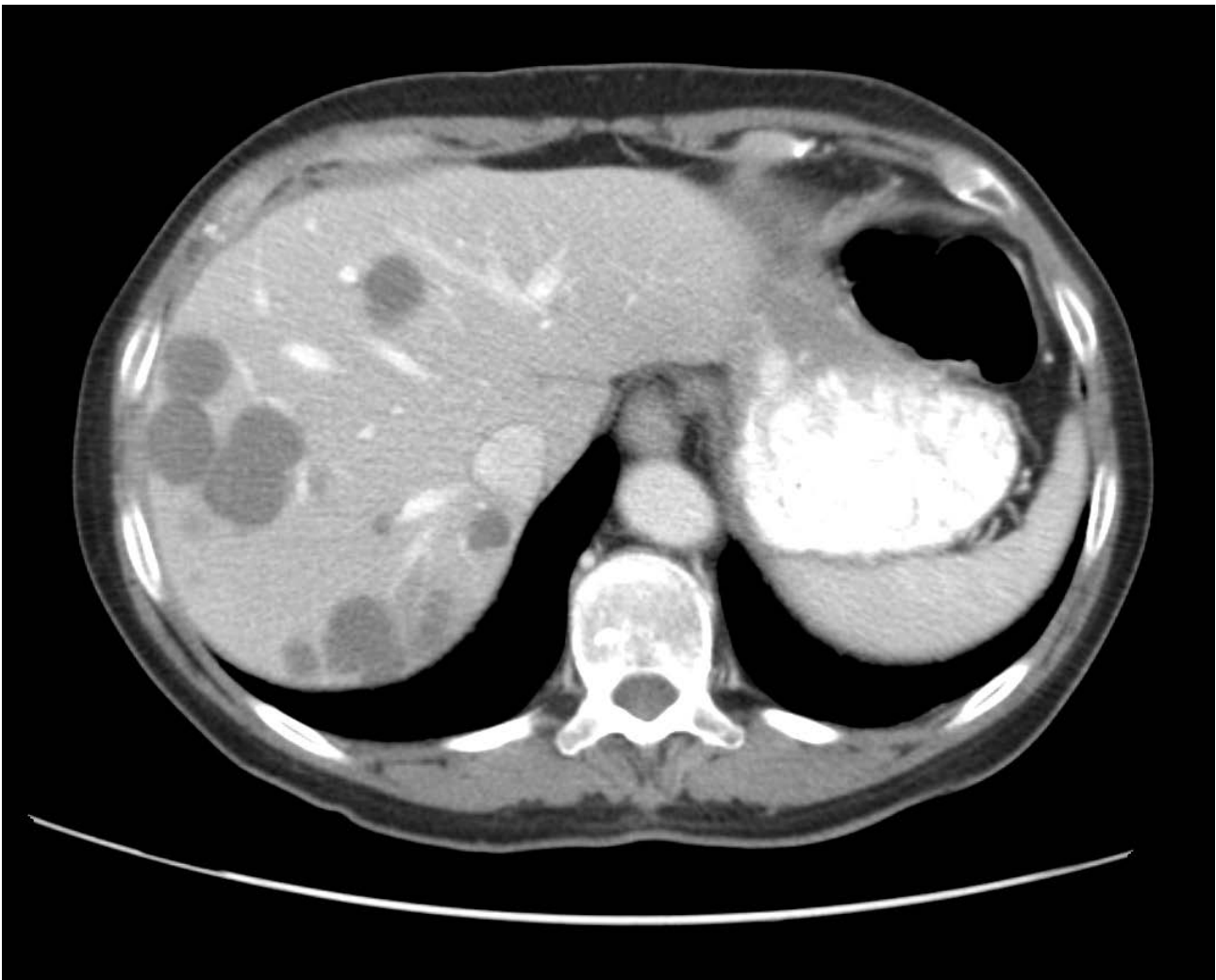

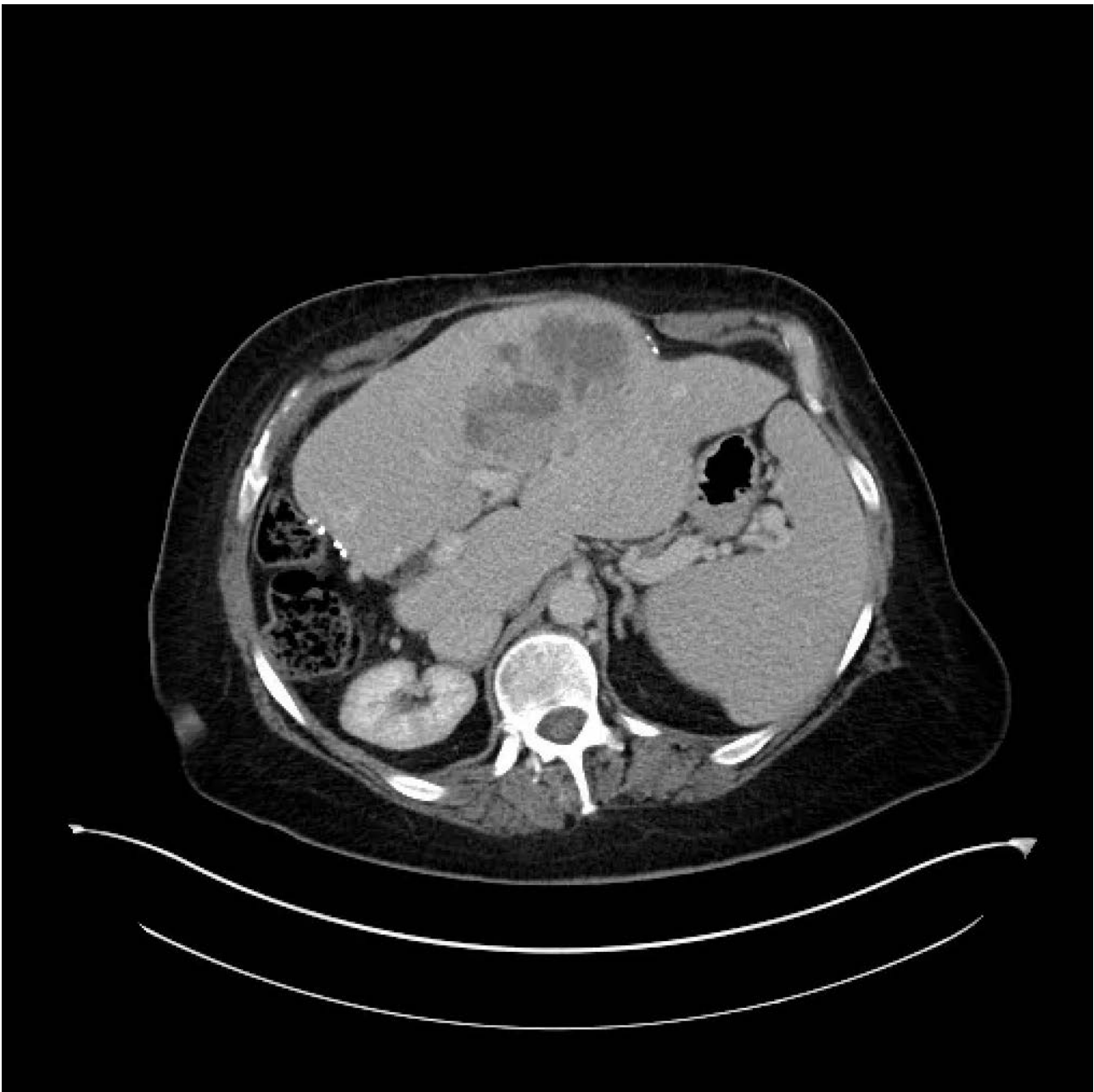

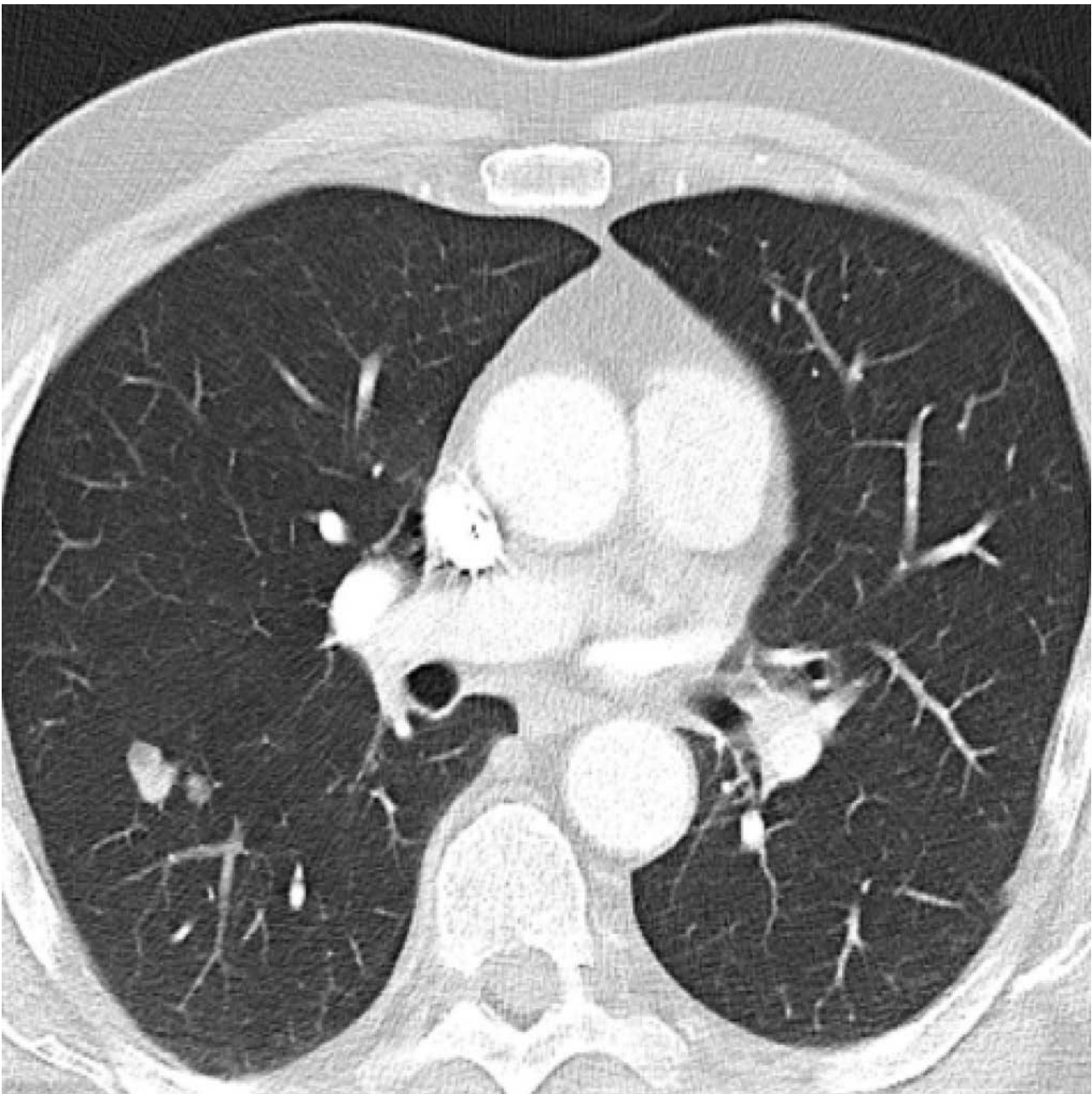

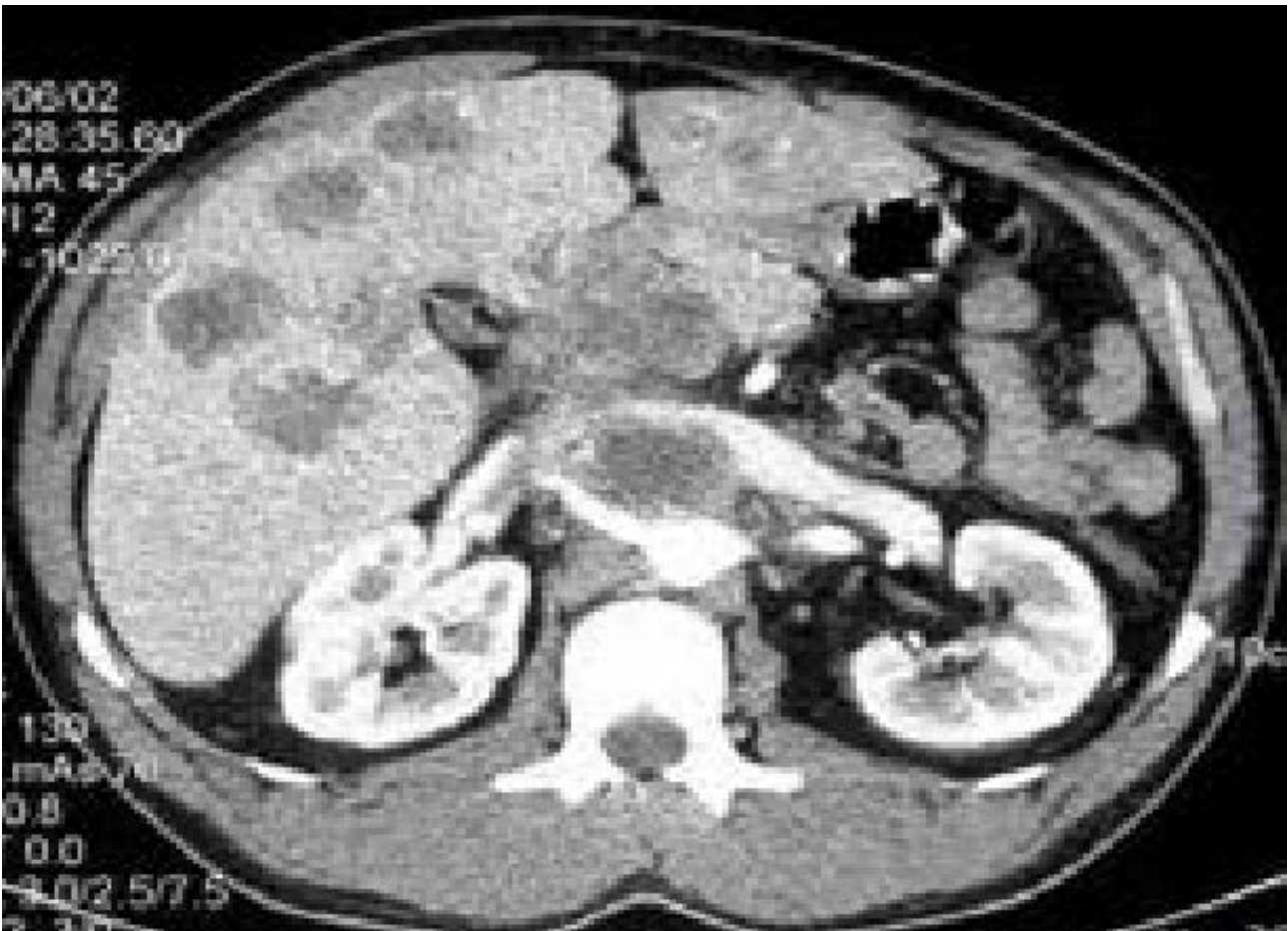

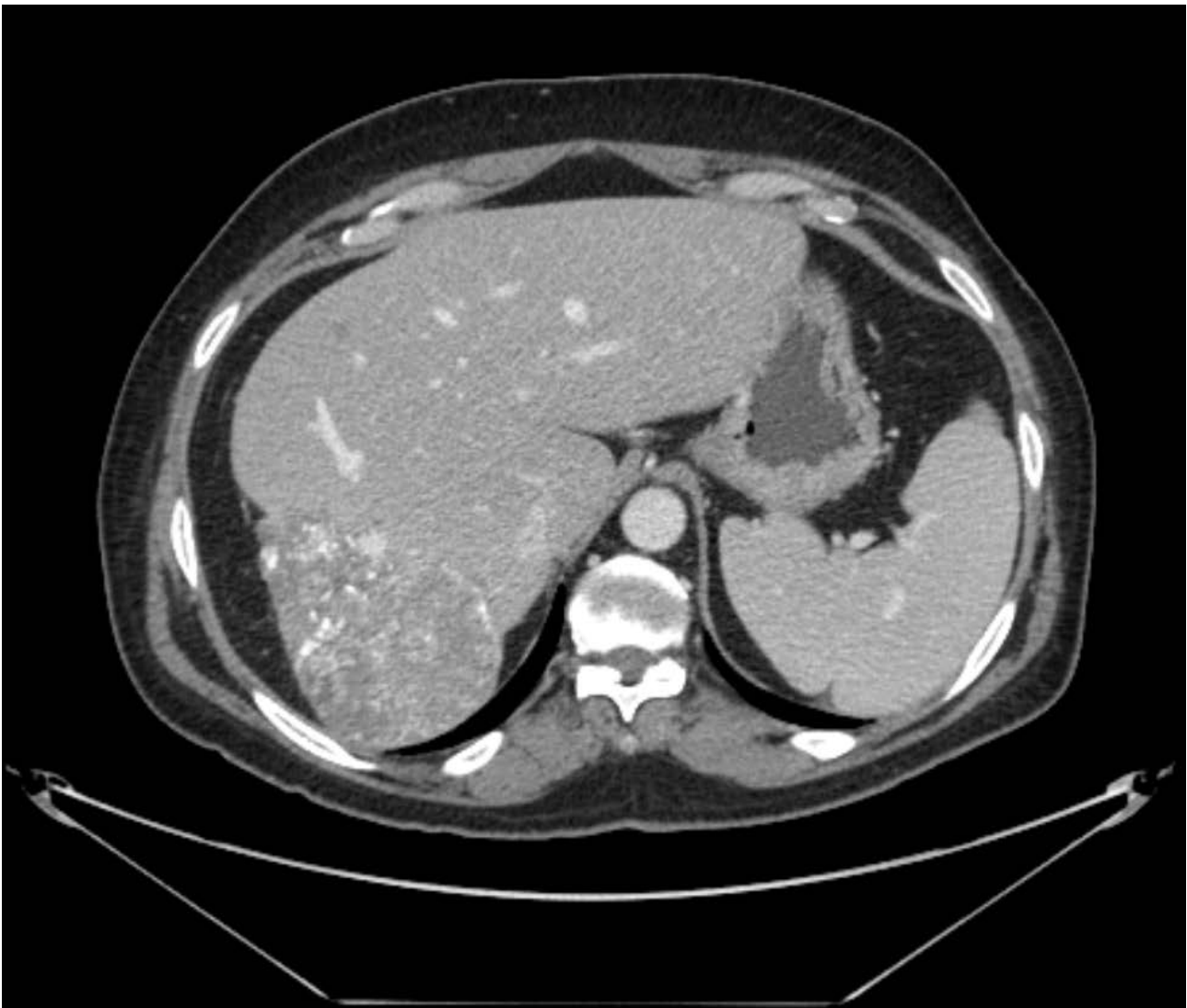

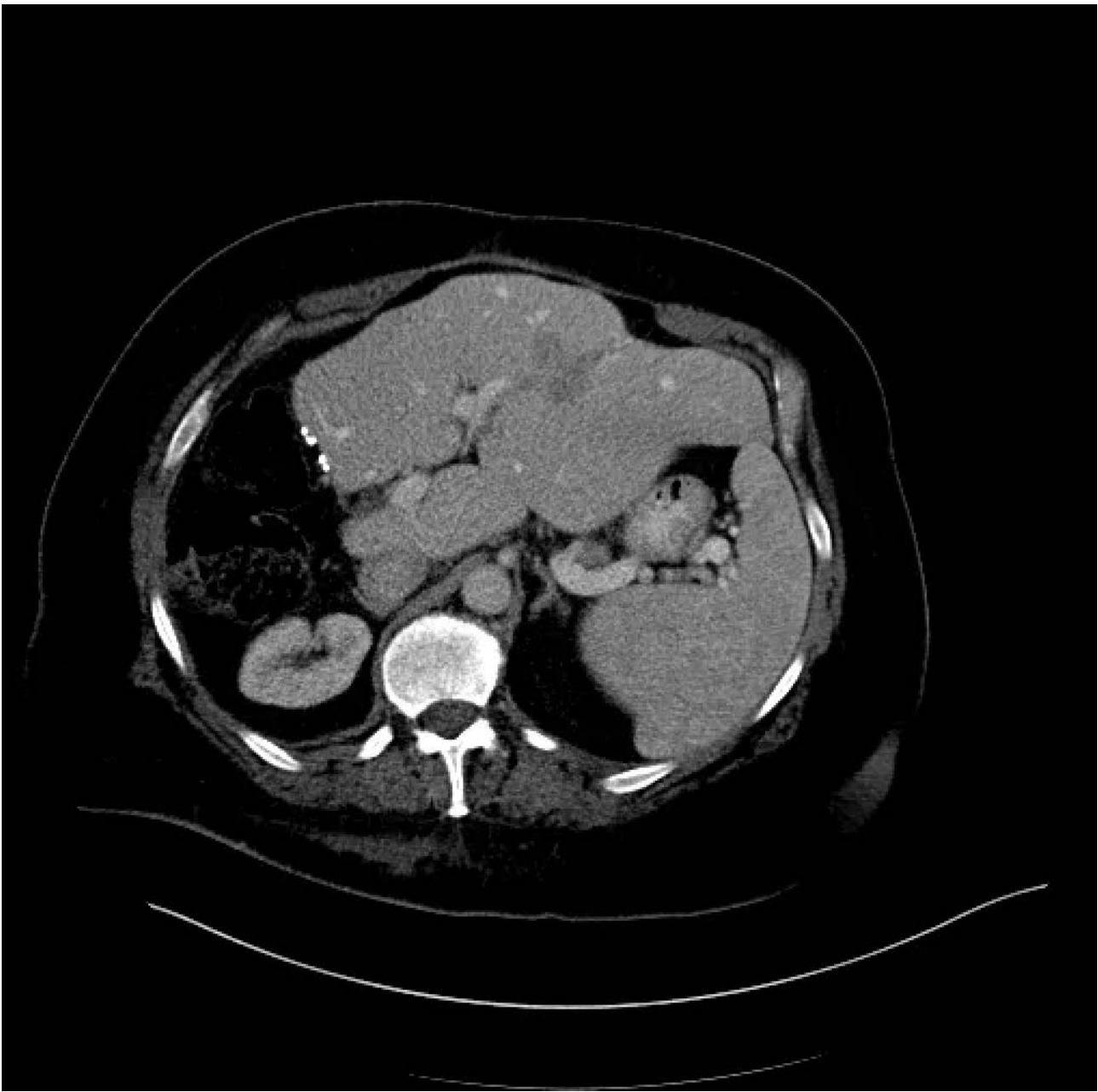

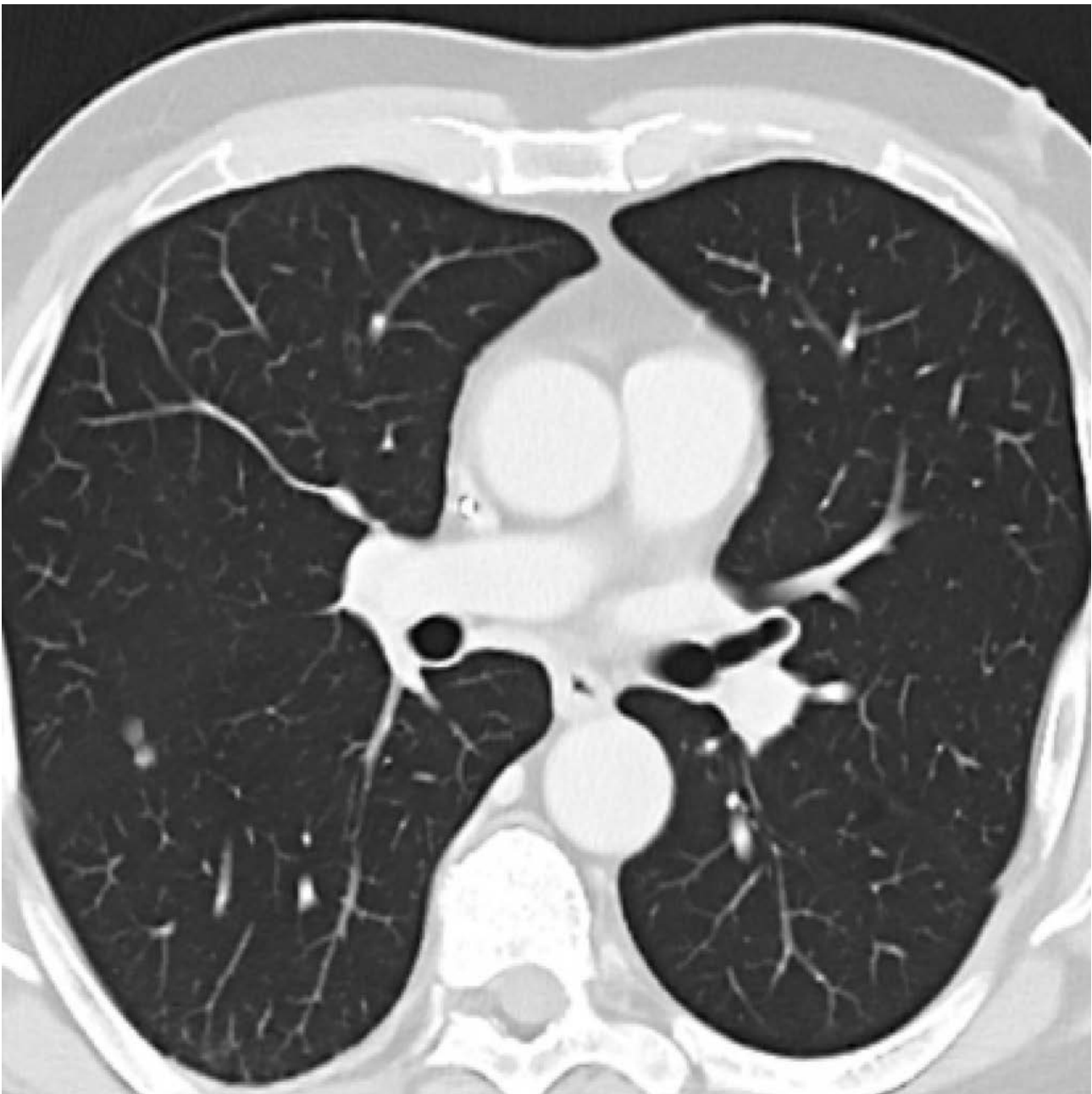

Supplement: Supplementary file 6 — Image source data. [file 43018_2025_991_MOESM6_ESM.pdf]
